# Hybrid intelligent RSM–ANN modeling and optimization of precision turning of CK45 steel for calibration devices

**DOI:** 10.1038/s41598-026-43388-w

**Published:** 2026-04-02

**Authors:** W. M. Farouk, Amer G. Ahmed, Mohammed Gamil, Farah Tamer Nasser, Mohamed Abu-Okail, Mamdouh I. Elamy

**Affiliations:** 1 Mechanical Engineering Department, Faculty of Engineering (Benha), Benha University, Benha, Egypt; 2 Nuclear Metallurgy Department, Atomic Energy Authority, Cairo, Egypt; 3 Department of Mechanical Engineering, Faculty of Engineering-Shoubra, Benha University, Cairo, Egypt; 4 Industrial Engineering Department, College of Engineering, Northern Border University, Arar, Saudi Arabia; 5 Department of Production Engineering and Mechanical Design, Faculty of Engineering, Menoufia University, Shebin El-Kom, Egypt; 6 Korean Egyptian Faculty for Industry and Energy Technology, Beni-Suef Technological University, Beni-Suef, 62521 Egypt; 7 Mechatronics Technology Department, Higher Technological Institut, Beni-Suef, Egypt

**Keywords:** Response Surface Methodology (RSM), Artificial Neural Networks (ANN), Hybrid RSM–ANN frame work, Metal removal rate, (MRR), Tool wear rate (TWR), Arithmetic average surface roughness (Ra), Roundness error (OR), Engineering, Materials science

## Abstract

Precision turning of CK45 steel for calibration devices requires stringent control of surface characteristics, geometrical accuracy, and tool wear. This paper introduces a hybrid intelligent modeling and optimization framework that combines Response Surface Methodology (RSM) with Artificial Neural Networks (ANN) to simultaneously forecast machining efficiency and correlate it with microstructural evolution. Experiments were carried out via cubic boron nitride (CBN) tools under a central composite design, considering spindle speed (N), feed rate (F), depth of cut, (D) and tool nose radius (R) as key process parameters. The effects of these variables on material removal rate (MRR), tool wear rate (TWR), surface roughness parameters (Ra and R_max_), roundness error (OR), and hardness (H) were systematically analyzed. While RSM regression models provided initial predictive capability, their integration with ANN significantly enhanced accuracy, yielding prediction errors below 7% for all responses. Multi-objective optimization identified machining conditions that minimized tool wear (1.23 × 10⁻⁵ g/min) while improving surface quality and dimensional precision. Microstructural analysis further revealed the formation of refined and homogeneous dendritic structures with characteristic sizes ranging from 10.86 to 17.44 μm under optimized conditions. The proposed hybrid intelligent RSM–ANN framework offers a robust and reliable approach for precision turning optimization and enables new insights into the linkage between machining parameters and microstructural characteristics of CK45 steel.

**Supplementary Information:**

The online version contains supplementary material available at 10.1038/s41598-026-43388-w.

## Introduction

CK45 steel is a widely used medium-carbon, non-alloy steel with medium carbon content of 0.45%^1^, renowned for its high tensile strength and machinability^2^. It is extensively employed in precision machinery and calibration components, typically supplied in untreated or normalized form and manufactured through hot rolling and forging. Its mechanical characteristics can be further improved through heat-treatment processes, including quenching and tempering^3^.

However, conventional machining of CK45 presents significant challenges due to high hardness, pronounced work hardening, excessive tool wear, and difficulty achieving high-quality surface finishes. Optimization studies on turning parameters have been performed across various materials, as aluminum, brass, and carburized mild steels, using experimental and statistical techniques as force dynamometry, Taguchi design, ANOVA, and response surface methodology (RSM)^4–8^. These studies highlight the critical impact of spindle speed (N), feed rate (F), depth of cut (D), and tool geometry on material removal rate (MRR), surface roughness (Ra, Rz, Rt), and tool wear rate (TWR)^9–11^. For instance, Bharilya et al.^3^ reported optimal machining conditions for carburized mild steel, aluminum alloys, and brass, demonstrating how F and N significantly affect MRR and surface roughness (SR), with two-factor and squared-term interactions playing important roles. Other investigations revealed similar trends in hot turning Monel-400, EN31, 42CrMo4, and bearing steels, emphasizing the influence of machining parameterizes and workpiece properties on tool wear, SRs, and cutting forces^12–16^.

Advanced machining techniques, including electric pulse-assisted hard turning (EPAHT)^17^, ultrasonic vibration-assisted turning (UVAT)^18^, and cryogenic CO₂ cooling^19^, have been applied to improve surface integrity, reduce roughness, and extend tool life. These methods enhance dynamic recrystallization, reduce work hardening, and maintain minimal surface deviations even under high-speed turning conditions. Additionally, forecast modeling approaches, as artificial neural networks (ANN) and genetic algorithms (GA), have been employed to estimate complex relationships among process parameters and efficiency metrics^20–23^. Integrating these models with multi-objective optimization methods allows for accurate prediction of MRR, TWR, and surface roughness while minimizing experimental inefficiency.

Recent developments have focused on prognostic modeling tools, as ANN and GA, to estimate complex relationships among machining parameters and process efficiency^24^. To reduce trial inefficiency, advanced optimization methods have become widely applied. Abdulridha et al.^21^ integrated Taguchi design with ANN to forecast surface roughness and cutting forces with high precision (MSE < 0.33). Premphet et al.^25^ applied central composite design (CCD) to obtain optimize turning conditions, highlighting V and F as the more effective parameters. Gonzalez et al.^26^ employed a Box–Behnken design to study tool path strategies, identifying specific geometries that provided improved stability but lower performance compared to conventional approaches. The robustness of the hybrid RSM–ANN framework has been recently validated in additive manufacturing contexts. Nasser et al.^27^ utilized this dual-modeling approach for the optimization of FDM-printed biomaterials, concluding that while RSM is effective for determining main effects, ANN offers superior predictive accuracy for highly non-linear responses with deviation less than 6%. Building on this proven methodology, the present study extends the application of this hybrid framework to the subtractive manufacturing domain, specifically investigating the precision turning of CK45 steel.

There is a critical gap in applying predictive frameworks to medium carbon steels (CK45) intended for high-precision calibration devices.

Consequently, this study addresses this necessity by uniquely contrasting Single-Output versus Multi-Output ANN architectures to capture the specific non-linear tool wear behavior of CK45, which differs significantly from the wear mechanisms observed in hardened alloys.

Despite extensive research, a significant knowledge gap persists: prior studies have not comprehensively integrated machining performance metrics with metrologically validated morphological analysis under systematically varied cutting conditions for calibration shafts.

This study addresses this gap by suggesting a novel, measurement-oriented predictive method that uniquely incorporates RSM and ANN with detailed experimental evaluation of both workpiece and tool surface morphology. Key process parameters, N, F, D, and R are quantitatively correlated with measurement-based performance indicators, including MRR, TWR, SR (Ra, R_max_), OR, and H.

The integration of predictive modeling with metrological assessment and uncertainty analysis provides a robust, traceable, and statistically validated approach for precision turning operations. Furthermore, this work offers new insights into the morphological interactions between CK45 and cutting tools of CBN, enhancing understanding of tool–workpiece behavior under optimized conditions. Overall, the study establishes a comprehensive, metrology-driven framework for improving process optimization, surface integrity, and measurement reliability in high-precision manufacturing applications.

## Experimental procedures

### Steps

**Fig. 1:**
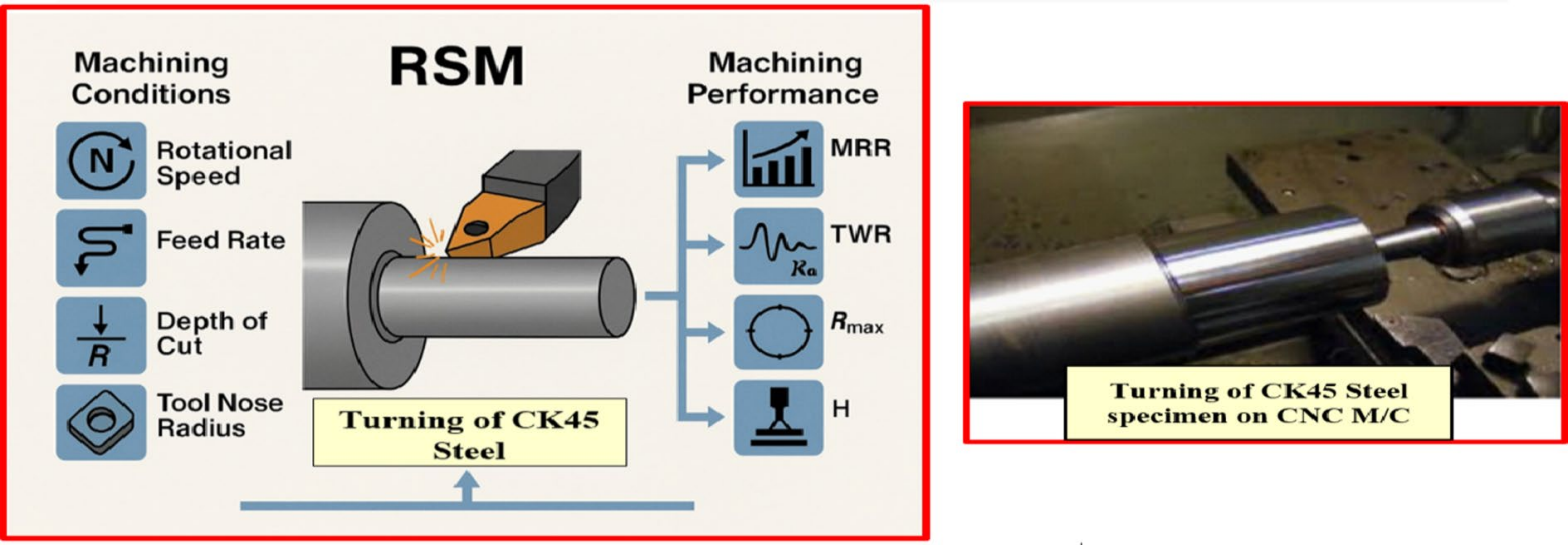
Schematic diagram of turning machining conditions and performance of CK45 by using response surface methodology.

In the current work, a TP was performed on a cylindrical CK45 workpiece with a diameter of 15 mm and a length of 25 mm. The straight turning operation was conducted on a Mori Seiki NL 2500 CNC lathe under dry machining conditions, as shown in Fig. 1, which shows the configuration of CK45 specimens. The MRR was measured, while other performance parameters, including (i) TWR, (ii) R_a_, and (iii) R_max_ were calculated using a tester for SR.

Additionally, OR was assessed with a Mitutoyo roundness testing device. These machining conditions are related to the input variables as N, F, D, and R in TP. The holder for tool that used was the MWLNR 2525 M-0.8 W. CBN has been used as an insert for turning. The listed ISO designation is SNGA12 04 08 T01020. It is installed on the tool holder.

The rake angle is 6, the clearance angle is 6, the cutting edge inclination angle is 6, and the cutting edge angle is 75. Each trial was conducted using a new cutting edge.

Various hardness measurements were performed on a 206 RT hardness tester, from AFFRI, with a preload of 98.07 N and test loads of 1471 N. Wear of the tool was observed by a Mitutoyo tool maker’s microscope, providing an accuracy of 0.001 mm.

SR was assessed using a Mitutoyo SJ-301P handheld equipment, with a length of sample equal to 2.5 cm and a base range of 0.8 mm.

For each workpiece, three measurements were taken and their average was calculated to minimize measurement uncertainty. Prior to the main experimental design, pilot experiments were carried out to ascertain the effective working limits of the cutting conditions and ensure the stability of the machining setup. Schematic diagram of turning machining conditions and performance of CK45 by RSM is depicted in Fig. 1.

The chemical and mechanical characteristics of CK45, it is mainly used in military applications due to its difficulty in manufacturing compared to traditional methods, and are shown in Tables 1 and 2.

Each experiment utilized a CK45 workpiece, with static parameters and operating conditions detailed in Table 3. The selection of values for each factor and machining condition was guided by insights, foundational studies, and hands-on experience.

Before and following each experiment, the specimens were washed with acetone, dried, and weighed on a precision digital electronic balance with an accuracy of 0.1 mg to calculate the MRR and TWR.

These rates were estimated by dividing the weight loss of the specimen and tool insert after machining by the elapsed time.

The purpose of the trial program was to explore the behaviors of machining conditions, N, F, D, and R on MRR, TWR, Ra, R_max_, and H. The program aimed to establish correlations between these factors using RSM. The experiments in the current study were conducted on small-scale specimens to allow precise control of process parameters and detailed microstructural characterization. So, scanning electron microscopy (SEM; Jeol JSM-7600 F), SEM coupled with energy-dispersive spectroscopy (EDS), and elemental mapping were utilized to assess morphological features, detect wear mechanism of single point cutting tools of turning processes, and analyze elemental distribution. Moreover, quantitative analysis of dendritic arm spacing, length, and morphology was performed using Image J software (version 1.53), which allowed automated measurement of dendritic parameters after calibration against known scale bars. To assess scalability to full-size calibration shafts, the optimized parameters obtained from the study were analyzed with consideration of industrial machining conditions. Consequently, the trends and optimal settings identified at small scale-such as peak current, pulse duration, and feed rate can be transferred to larger workpieces, provided that thermal and mechanical loadings are appropriately scaled. The manuscript now includes results and discussion on potential scaling factors, tool-path strategies, and industrial implementation considerations, highlighting the practical applicability of the proposed optimization for full-size manufacturing.

**Table 1:** Elemental composition for CK45 (wt%).

C	Si	Mn	*P*	S	Cr	Mo	Ni
0.4771	0.2105	0.628	0.0109	0.0111	0.1079	0.0236	0.0788
Al	Co	Cu	V	W	Sn	Fe	
0.0073	0.0073	0.1896	0.0035	< 0.0050	0.0103	98.23	

**Table 2:** Mechanical characteristics for CK45.

Tensile Strength, MPa	Yield Strength, MPa	Elongation, %	Impact Charpy, J
590–720	≥ 325	L:≥18	L:≥31

### Experimental plan

**Fig. 2:**
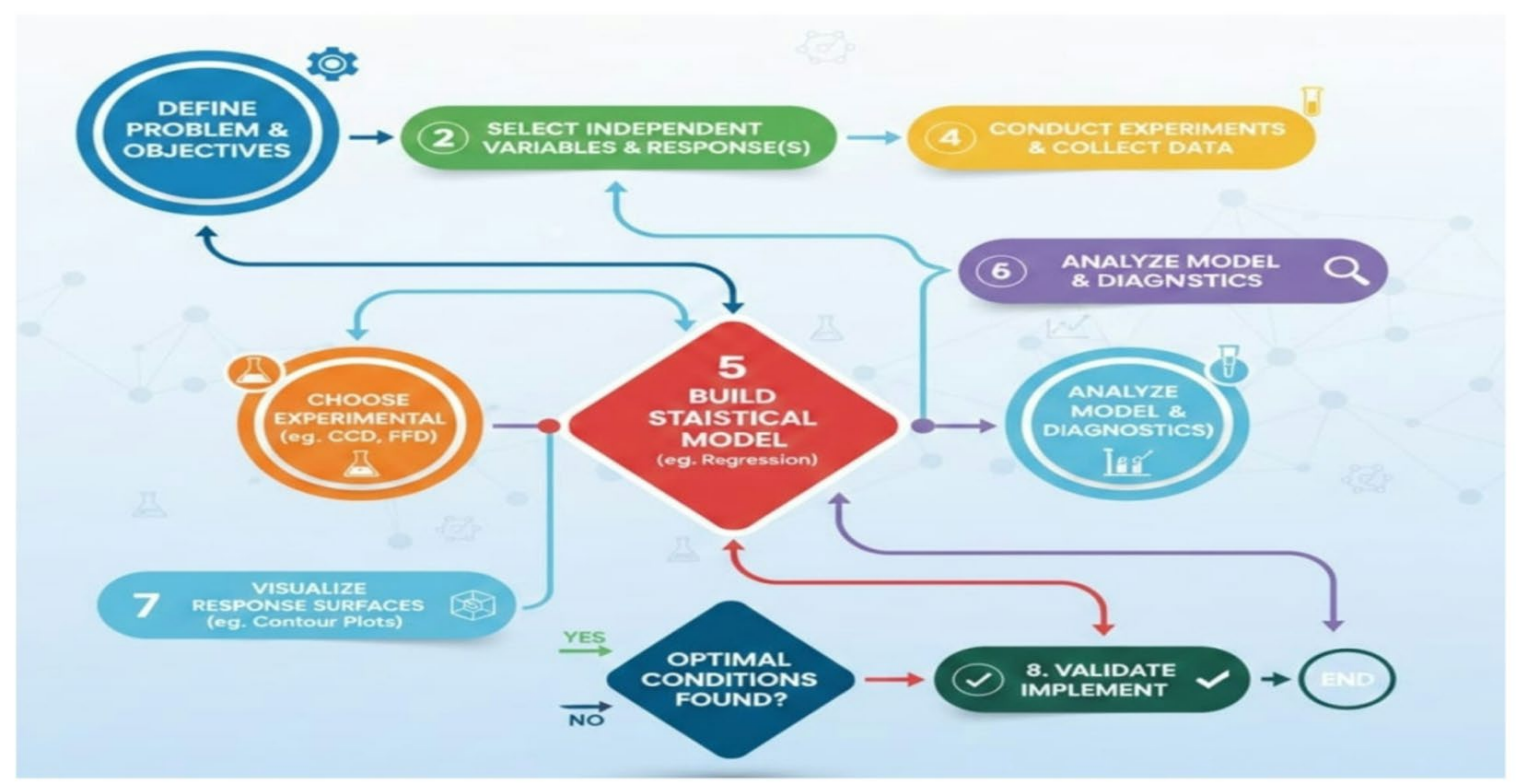
Flow chart of RSM procedure.

To model and optimize the response variables, the RSM was utilized. A sequence of consecutive steps was followed by the RSM method, as Fig. 2 shows. Preliminary experiments were used in this investigation to identify the parameters of N, F, D, and R. The coded and actual values for each operating state are shown in Table 3.

A sequence of studies was conducted using Central Composite Design (CCD) as the foundation for the trial framework. With sixteen points in corner, eight points in axial, and six points in center, the CCD is made up of a 2β fractional factorial design^28^.

The trial’s matrix design, which displays the process’s actual and coded input circumstances throughout (TP), is given in Table 3. The trial design followed these progressive stages:

1. The first experiment’s objective, as shown in Table 4, was to ascertain the effective range of operating conditions.
2. RSM was employed to enhance the trial design.
3. The operating conditions determined by RSM were the basis for the experimental procedures that were conducted.
4. The model was used to analyze the responses that were gathered.

**Table 3:** Coded and actual values of input machining conditions for the TP of CK45.

Input parameters	Symbol	Levels					Responses
		−2	−1	0	+ 1	+ 2	
A: N, (r.p.m)	X_1_	2000	2500	3000	3500	4000	MRR, (gram/min) TWR, (gram/min) R_a_, (µm), R_max_, (µm) OR (µm) H (µm**)**
B: F, (mm/min)	X_2_	100	350	600	850	1100	
C: D, (mm)	X_3_	0.4	0.55	0.7	0.85	1	
D: R, (mm)	X_4_	0.2	0.4	0.6	0.8	1	

### Material removal rate, (MRR)

The samples were weighed prior to and following the TP using a digital electronic balance. MRR was then calculated using the next formula:

1 $$MRR=\frac{\mathrm{W}\mathrm{b}-\mathrm{W}\mathrm{a}}{\mathrm{t}}$$

Where:

MRR: Material removal rate (g/min).

W_b_: Sample weight prior to turning process (g).

W_a_: Sample weight following the turning process (g).

t: Dwelling time of the turning process (min).

### Out of roundness, (OR)

A Roundness Testing Machine was used to calculate the OR for measurement purposes.

### Response surface methodology, (RSM)

RSM is a powerful tool incorporates statistical analysis with mathematical concepts, giving a comprehensive study for addressing problems which multiple independent parameters influence an outcome.

Finding these variables’ ideal values in order to get the required response performance is the main objective of RSM. Fundamentally, regression modeling makes clear the connection between variables and process response through its regression model.

This model is more than just a static representation; it is a prediction tool that makes it possible to estimate system reactions over a variety of process parameter ranges. A notable benefit of RSM is its ability to display the regression model graphically. RSM uses contour plots to map the response against pairs of process parameters in order to visually represent these correlations.

These graphics provide insightful information about how the response varies under different process conditions.

Consequently, it becomes easier to identify the ideal parameter values, which enhances decision-making and optimization techniques. Numerous empirical investigations have proven that RSM is beneficial in a variety of sectors.

To illustrate RSM’s adaptability in intricate industrial processes, Zihyun et al.^29^ combined RSM with ANN to predict NOx removal systems in LNG terminals.

Meanwhile, Al-Taweel and Jawda^30^ presented the effectiveness of RSM to improve process efficiency and quality by applying it to improve the electrochemical turning process. Hassan and Mohammed^30^. investigated the process Parameters optimization depend on RSM. ANOVA for SR and MRR during turning of MWCNTs-Al_2_O_3_/epoxy.

Mansour et al.^32^ exhibited the trials analysis of SR and MRR in turning process using RSM, W.M. Farouk et al.^33^ anticipated the analysis and looked into an experimental trial of the laser micro-turning precision procedure of Armoured 500 T with N_2_ gas accompaniment by RSM. In conclusion, W.M. Farouk et al.^34,35^ studied and used RSM to predict modeling and optimization for tubular distiller operation and pyramidal still performance infused with phase change material and nano material. RSM is useful methodology for predictive modeling and optimization with multiple objectives because of its capacity to represent complicated relationships in a visually understandable style and because of its shown performance in a variety of applications.

**Table 4:** CK45 trial design results, & relating CD of TP.

Exp. No.	X_1_ A: Rotational speed (*N*), (*r*.*p*.m)		X_2_ B: Feed rate (F), (mm/min)		X_3_ C: Depth of cut (D), (mm)		X_4_ D: Tool nose radius (*R*), (mm)		Observed responses						Composite desirability (CD)
	Coded X_1_	Actual *R*	Coded X_2_	Actual F	Coded X_3_	Actual D	Coded X_4_	Actual *R*	MRR (g/min)	TWR (g/min)	Ra (µm)	*R*_max_ (µm)	OR (µm)	H (µm)	
**1**	−1	2500	−1	350	−1	0.55	−1	0.4	0.261422	0.0001265	10.2	42	25	22	0.187784
**2**	1	3500	−1	350	−1	0.55	−1	0.4	0.3344	5.82E-05	4.6	20	20	14	0.632003
**3**	−1	2500	1	850	−1	0.55	−1	0.4	0.352097	8.09E-05	9.2	40	23	15	0.547762
**4**	1	3500	1	850	−1	0.55	−1	0.4	0.34582	2.75E-05	8.5	30	8	19	0.775745
**5**	−1	2500	−1	350	1	0.85	−1	0.4	0.296075	4.7E-05	9.4	39	13	20	0.592731
**6**	1	3500	−1	350	1	0.85	−1	0.4	0.324294	3.98E-05	3.4	10	30	7	0.677311
**7**	−1	2500	1	850	1	0.85	−1	0.4	0.388775	4.66E-05	9	35	19	17	0.781522
**8**	1	3500	1	850	1	0.85	−1	0.4	0.373374	5.43E-05	7.5	10	26	18	0.685146
**9**	−1	2500	−1	350	−1	0.55	1	0.8	0.150728	8.65E-05	7	20	15	16	0.136836
**10**	1	3500	−1	350	−1	0.55	1	0.8	0.395343	2.93E-05	2.5	25	30	17	0.828825
**11**	−1	2500	1	850	−1	0.55	1	0.8	0.2053	6.92E-05	5.5	35	15	10	0.334931
**12**	1	3500	1	850	−1	0.55	1	0.8	0.364555	2.7E-05	6.5	31	18	20	0.795085
**13**	−1	2500	−1	350	1	0.85	1	0.8	0.236476	3.87E-05	8.3	25	6	21	0.470853
**14**	1	3500	−1	350	1	0.85	1	0.8	0.40518	4.26E-05	3	14	40	11	0.810637
**15**	−1	2500	1	850	1	0.85	1	0.8	0.303763	6.67E-05	7.2	35	15	19	0.533074
**16**	1	3500	1	850	1	0.85	1	0.8	0.393779	8.55E-05	6.8	22	35	21	0.596914
**17**	−2	2000	0	600	0	0.7	0	0.6	0.165582	0.0001389	11	55	20	12	0
**18**	2	4000	0	600	0	0.7	0	0.6	0.351007	8.95E-05	5	30	45	10	0.499376
**19**	0	3000	−2	100	0	0.7	0	0.6	0.30577	4.27E-05	5.5	20	22	14	0.614451
**20**	0	3000	2	1100	0	0.7	0	0.6	0.39362	3.99E-05	9	27	16	19	0.776804
**21**	0	3000	0	600	−2	0.4	0	0.6	0.258218	5.55E-05	5	25	17	17	0.464420
**22**	0	3000	0	600	2	1	0	0.6	0.332189	3.45E-05	4.5	19	14	20	0.692073
**23**	0	3000	0	600	0	0.7	−2	0.2	0.4455	3.55E-05	9	20	19	18	0.885976
**24**	0	3000	0	600	0	0.7	2	1	0.399376	2.67E-05	5	18	18	19	0.835958
**25**	0	3000	0	600	0	0.7	0	0.6	0.405431	1.4E-05	4.5	17	16	22	0.919802
**26**	0	3000	0	600	0	0.7	0	0.6	0.408213	9E-06	4.8	20	14	23	0.919802
**27**	0	3000	0	600	0	0.7	0	0.6	0.407243	1E-05	4.6	19	20	25	0.919802
**28**	0	3000	0	600	0	0.7	0	0.6	0.405292	1.1E-05	4.5	18	17	26	0.919802
**29**	0	3000	0	600	0	0.7	0	0.6	0.402015	1.2E-05	4.4	17	19	25	0.919802
**30**	0	3000	0	600	0	0.7	0	0.6	0.401864	1.3E-05	4.5	16	18	27	0.919802

### Artificial neural network (ANN), modeling

**Fig. 3:**
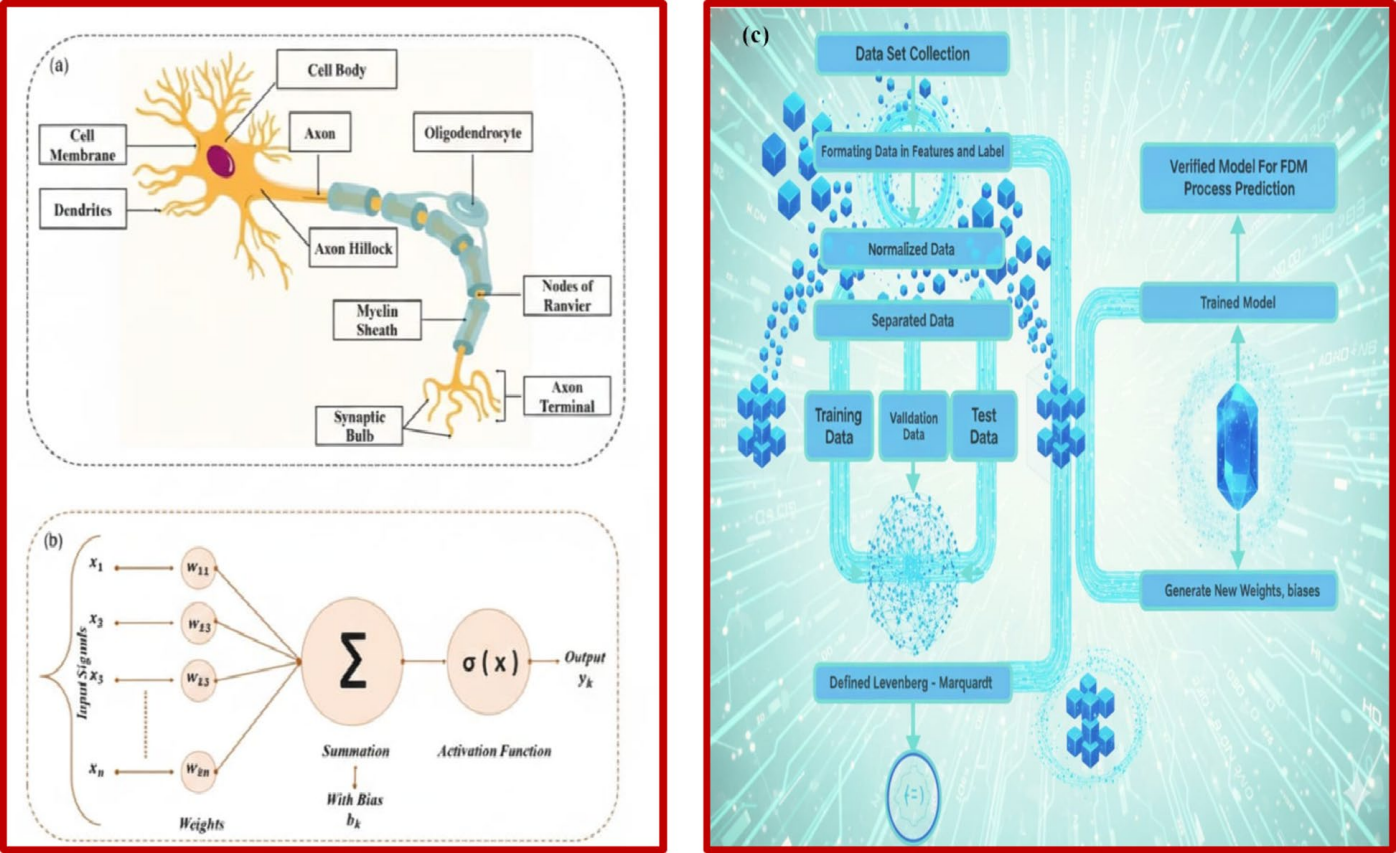
Schematic illustration of (**a**), The fundamental components of an artificial neuron^20^, (**b**) the mathematical representation of ANN^20^, and the overall workflow of the present study for predicting responses of precision turning of CK45 using ANN algorithm.

ANNs are computational models depend on neuronal cells, widely used to forecast machining performance through a multilayer structure comprising input parameters, hidden layers for neuron optimization, and output responses, with weights and biases adjusted to minimize MSE and ensure effective generalization^36^. In this work, the ANN model was developed by MATLAB R2021b, and Fig. 3 illustrates the artificial neuron components, mathematical formulation, and ANN algorithm workflow.

**Fig. 4:**
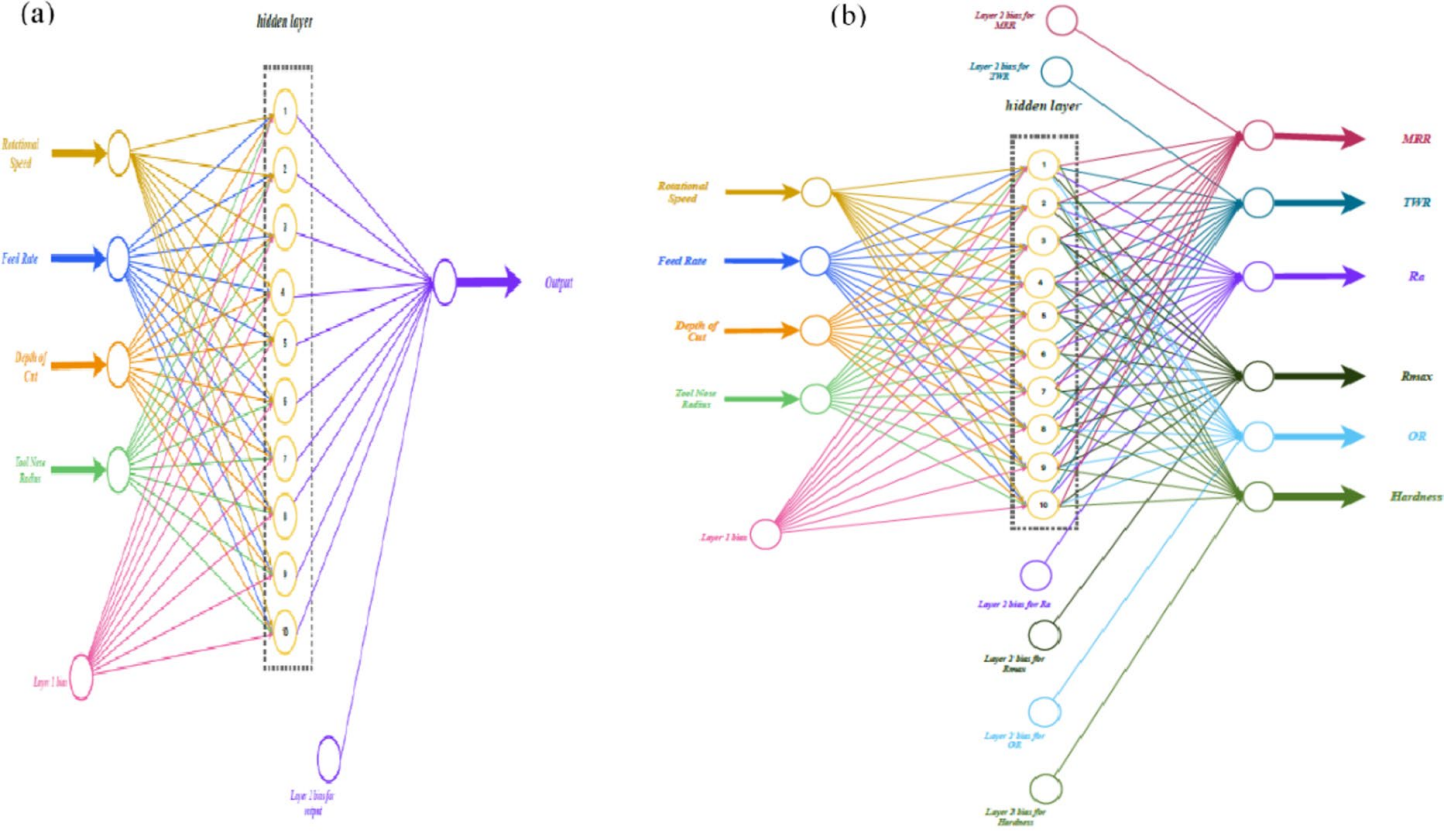
(**a**) The 4–10-1 Single Output ANN (**b**) The 4–10-6 Multi Output ANN.

Six distinct networks were developed, each focused on predicting one output parameter: MRR, TWR, Ra, R_max_, OR, and H. These models were built with a 4–10-1 architecture. The four input variables N, F, D, and R were processed through a hidden layer of 10 neurons, generating a single output parametric for each network, as illustrated in Fig. 4(a).

An incorporated network was constructed to forecast six output parametrices at once. The model applies shared weights and biases in the hidden layer, while separate weights and biases link the hidden layer to each output, as shown in Fig. 4 (b). The purpose of this combined model was to compare its performance with that of individual networks in predicting outcomes.

**Fig. 5:**
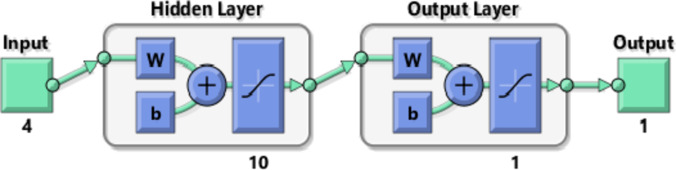
Hidden tier and output tier tansig function representation for a single output.

Neural models were trained through Levenberg–Marquardt (LM) back spread method, with the adaptation learning rule (LEARNGDM) and the hyperbolic tangent sigmoid signal transformation function (TANSIG). The design involved a single hidden layer, as displayed in Fig. 5. The number of neurons in this layer was determined experimentally by trial tuning to balance accuracy and complexity. Architectures with 5 and 8 neurons were also tested but yielded higher Mean Squared Errors compared to the 10-neuron configuration. Too few neurons resulted in under fitting, while too many caused overfitting. A configuration of 10 neurons achieved the most effective generalization on the validation set, delivering accurate predictions without capturing random noise from the training data.

The ANN models were trained using the same experimental dataset consisting of 30 experimental runs, consistent with the RSM design. The experimental results were divided into three groups: training, validation, and testing, with a 70:15:15 distribution. This division is a common and necessary step in machine learning to provide a reliable and unbiased assessment of last model. All input and output variables were normalized prior to training to ensure numerical stability and balanced learning. In the training stage, the training dataset was used to adjust weights and reduce prediction errors. The validation dataset was employed to refine the network structure. Random initialization of weights and biases was employed to enhance generalization and avoid dependence on a specific initialization. The chosen learning parameters for both single- and multi-output networks are presented in Table 5.

**Table 5:** Learning factors selected for ANN.

Learning Factors	Single Output ANN	Multi-Output ANN
Training Function	Train Levenberg-Marquardt (LM)	
Adaption Learning	Function LEARNGD (Gradient descent)	
Performance Function	Mean Square Error	
Network Topology	4–10-1	4–10-6
Transfer Function	TANSIG	
Number of Hidden Layers	1	
Number of Neurons	10	
Training Method	Back-propagation	
Number of Epochs	100	

## Results and discussion

### Parameters optimization, predictive modeling, sensitivity analysis, and model validation were performed using RSM

Regression analysis combined with experimental trials allows one to forecast the intended response as a function of different independent input parameters. When every element is understood, the Yu can be written as:

2 $$\mathrm{Y}\mathrm{u}=f\left(\mathrm{X}\mathrm{1},\mathrm{X}\mathrm{2},\mathrm{X}\mathrm{3},\dots\dots.,\mathrm{X}\mathrm{k}\right)\pm\epsilon$$

where Yu represents the computed answers for X_1_, X_2_, X_3_, and X_4_.For TP, ε represents error, and X_k_ are independent variables of the input circumstances. N, F, D, and R were the four independent variables that were investigated in this study. The relationship amon Yu and independent variables was stated by a quadratic equation that was provided in the current work and is referred to as a second-order polynomial regression formula. The following equation is provided^37^:

3 $${Y}_{u}={b}_{o}+\sum_{i=1}^{n}{b}_{i}{X}_{iu}+\sum_{i=1}^{n}{b}_{ii}{X}_{iu}^{2}+\sum_{j>i}^{n}{b}_{ij}{X}_{iu}{X}_{ju}+\epsilon$$

The coefficients bo, bi, bij, and bii, respectively, reflected the free component, linear part, interaction part, and quadratic part. By looking at the outcomes displayed in Table 6, the equations’ form was established. Using ANOVA, the formula’s sufficiency was evaluated. By using this method, the advanced equations will be deemed reliable if their estimated F magnitude is less than or equal to the required degree of dependability (99.13%, 99.9%, 99.6%, 96.6%, 96.1%, and 95.2% for MRR, TWR, R_a_, R_max_, OR, and H, respectively). Tables 6, 7, 8, 9 and 10, and 11 contain the ANOVA tables for MRR, TWR, Ra, R_max_, OR, and H, respectively.

To test the statistical significance and reliability of the RSM regression models, Analysis of Variance (ANOVA) was carried out for each response variable as following.

The ANOVA results presented in Table 6 indicate that the suggested MRR mathematical model was statistically significant. The F value of 121.46 shows that the equation was affected. “Prob > F” magnitudes less than 0.05 suggested that the equation’s components were affected, whereas values more than 0.1 demonstrated none affected.

Reducing the equation can simplify the model if many of its components remain unchanged. With the exception of a few quadratic and interaction components, including the linear effects of (X_4_^2^) and (X_2_ × _3_), which are included in Table 6, nearly all of the terms were affected. In comparison to the pure error, the improper fit F value of 21.16 showed that the unsuitable fit F is impacted. An F-value that is too high due to poor fit is not appropriate.

The determination coefficient (R^2^ = 0.9913) demonstrates the significance of the model.

**Fig. 6:**
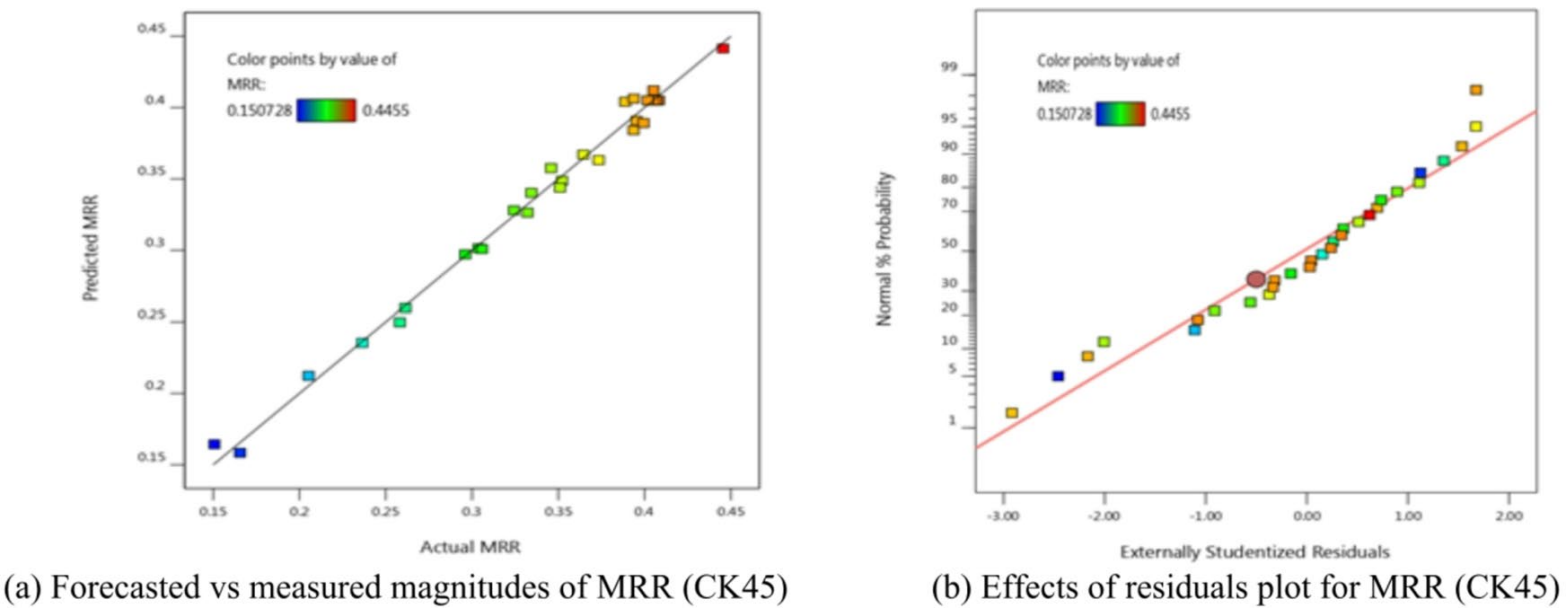
Schematic diagnostics of MRR.

There may be some disparity between the “Adj R^2^” of 0.9831 and the “Pred R^2^” magnitude of 0.9505, which are in reasonable accord. In this case, the accuracy suggested a satisfactory signal based on the (S/*N* = 40.176). This model can be utilized to translate the design space. The graphical analyses of the data are also shown in Fig. 6, where Fig. 6a compares measured and expected values and Fig. 6b shows residuals.

The scattered nature of the data points along the line. The residual graph demonstrated a respectable degree of correlation between the trial and projected magnitudes.

The ANOVA and visual diagnostics curves demonstrated a strong level of agreement between the suggested mathematical model for MRR predictions and the observed data. According to the model’s F-value of 121.46, the model is influenced clearly.

Such a large F-value suggests a mere 0.01% likelihood of being attributed to random noise. Model terms are deemed significant when their P-values fall below 0.0500.

In this case, the significant model terms are X_1_, X_2_, X_3_, X_4_, X_1_X_2_, X_1_X_3_, X_1_X_4_, X_2_X_4_, X_3_X _4_, X_1_^2^, X_2_^2^, and X_3_^2^.

Model terms are considered unimportant if their value exceeds 0.1000. If the model contains numerous non-significant terms, model reduction can enhance its performance. The F-value of 21.16 for the lack of fit indicates that the lack of fit is substantial.

A significant F-value for Lack of Fit could only occur in 0.18% of cases because of noise. The model should fit if there is an affect lack of fit.

The “Adj R^2^” of 0.9831 and the “Pred R^2^” value of 0.9505 are reasonably in agreement; that is, the difference is less than 0.2. The S/N is measured with a suitable precision. It is more desirable to have a S/N ratio larger than 4. A sufficient signal is shown by the ratio (S/*N* = 40.176). This model can be used to navigate the design area.

4 $$\begin{aligned}\mathrm{M}\mathrm{R}\mathrm{R}&=-1.91799+0.000999351\mathrm{X}_1\hspace{0.17em}+\hspace{0.17em}0.000852733\mathrm{X}_2\hspace{0.17em}+\hspace{0.17em}2.20448\mathrm{X}_3-1.30876\mathrm{X}_4-1.43462\mathrm{E}\\&-07\mathrm{X}_1\mathrm{X}_2-\hspace{0.17em}0.000165861\mathrm{X}_1\mathrm{X}_3\hspace{0.17em} +\hspace{0.17em}0.000364419\mathrm{X}_1\mathrm{X}_4\hspace{0.17em}+\hspace{0.17em}0.000119645\mathrm{X}_2\mathrm{X}_3-\hspace{0.17em}0.000205256\mathrm{X}_2\mathrm{X}4\hspace{0.17em}\\&+\hspace{0.17em}0.280194\mathrm{X}_3\mathrm{X}_4-\hspace{0.17em}1.53846\mathrm{E}-07\mathrm{X}_1^2-\hspace{0.17em}2.49783\mathrm{E}-07\mathrm{X}_2^2-\hspace{0.17em}1.2993\mathrm{X}_3^2\hspace{0.17em}+\hspace{0.17em}0.0643586\mathrm{X}_4^2\end{aligned}$$

**Table 6:** ANOVA of MRR for (CK45).

Source	SS	DF	MS	F value	*P* value	(% Contribution) or sensitivity
M	0.1688	14	0.0121	121.46	< 0.0001 (significant)	99.17
X_1_	0.0516	1	0.0516	520.07	< 0.0001	30.31
X_2_	0.0104	1	0.0104	104.65	< 0.0001	6.11
X_3_	0.0088	1	0.0088	88.84	< 0.0001	5.17
X_4_	0.0041	1	0.0041	41.23	< 0.0001	2.41
X_1_^2^	0.0406	1	0.0406	408.86	< 0.0001	23.85
X_2_^2^	0.0067	1	0.0067	67.36	< 0.0001	3.93
X_3_^2^	0.0234	1	0.0234	236.21	< 0.0001	13.74
X_4_^2^	0.0002	1	0.0002	1.83	0.1960 (Not significant)	0.118
X_1_X_2_	0.0051	1	0.0051	51.85	< 0.0001	2.99
X_1_X_3_	0.0025	1	0.0025	24.95	0.0002	1.47
X_1_X_4_	0.0212	1	0.0212	214.11	< 0.0001	12.45
X_2_X_3_	0.0003	1	0.0003	3.25	0.0918 (Not significant)	0.18
X_2_X_4_	0.0017	1	0.0017	16.98	0.0009	0.998
X_3_X_4_	0.0011	1	0.0011	11.39	0.0042	0.65
Residual	0.0015	15	0.0001			
Lack of Fit	0.0015	10	0.0001	21.16	0.0018 (significant)	
Pure Error	0	5	6.874E-06			
Cor Total	0.1702	29				

The TWR is expressed mathematically in Eq. (5). Table 7 displays the ANOVA results. The model’s relevance is conducted by the derived F-value of 1920.53. Furthermore, there was a respectable degree of agreement between the R^2^ = 0.9655 and the adjusted R^2^ = 0.9994 and the projected R^2^ = 0.9989. About S/*N* = 166.797 made it evident that the S/N ratio was suitable.

Use this formula to explore the design space. However, it is clear from the P-values of a number of equation terms that the evidence was not significant (P-value 0.10). Model terms with P-values less than 0.0500 are deemed significant. X_1_, X_2_, X_3_, X_4_, X_1_X _2_, X_1_X _3_, X_1_X_4_, X_2_X _3_, X_2_X _4_, X_3_X_4_, X_1_^2^, X_2_^2^, X_3_^2^, and X_4_^2^ are significant model terms in this case.

**Fig. 7:**
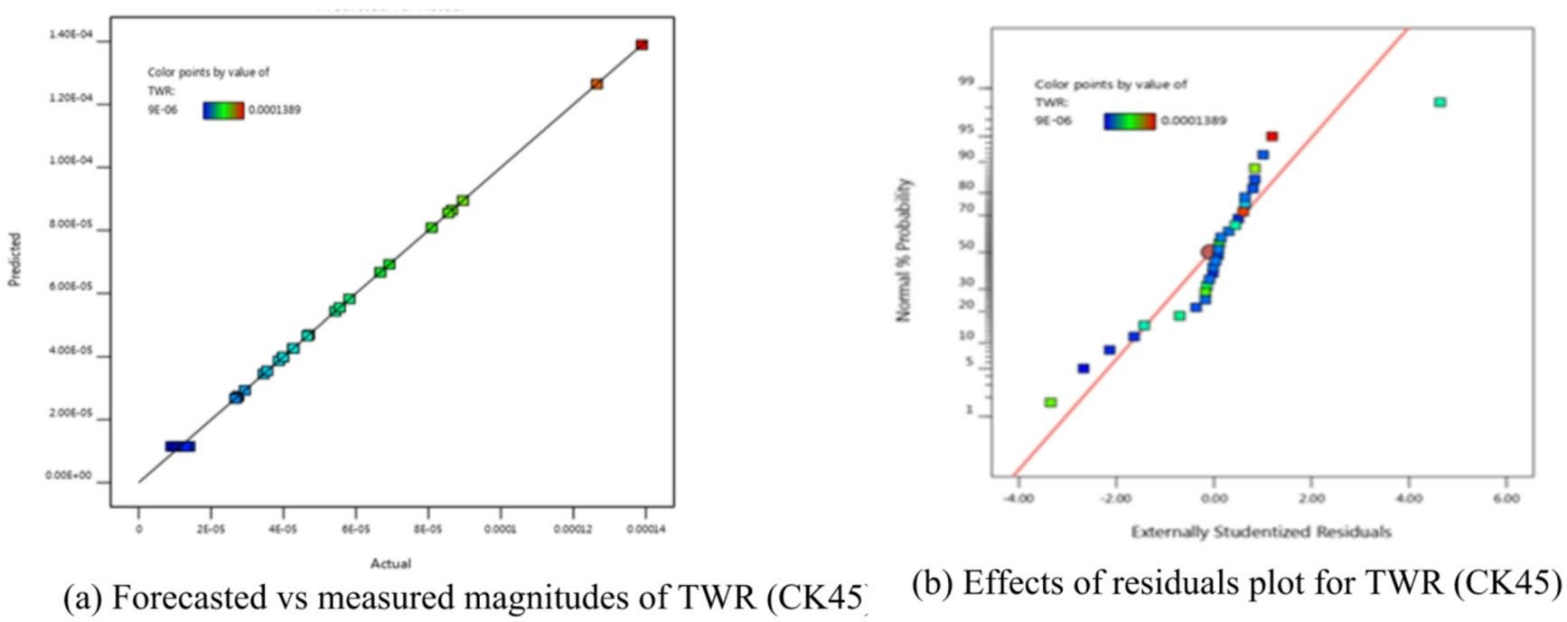
Schematic diagnostics of TWR.

Table 7 included an indication of these terms. The projected and actual results are presented in Fig. 7(a), and the residuals are presented in Fig. 7(b). The results evidently show a strong alignment among the measured results and the estimates produced using TWR mathematical formula.

5 $$\begin{aligned}\mathrm{T}\mathrm{W}\mathrm{R}&=0.002132-8.18006\mathrm{E}-07\mathrm{X}_1-5.31495\mathrm{E}-07\mathrm{X}_2-0.001506\mathrm{X}_3- 0.000512\mathrm{X}_4+2.98500\mathrm{E}-11\mathrm{X}_1\mathrm{X}_2\\&+2.03583\mathrm{E}-07\mathrm{X}_1\mathrm{X}_3+2.78125\mathrm{E}-08\mathrm{X}_1\mathrm{X}_4+3.01500\mathrm{E}-07\mathrm{X}_2\mathrm{X}_3+1.41875\mathrm{E}07\mathrm{X}_2\mathrm{X}_4\\&+0.000264\mathrm{X}_3\mathrm{X}_4+1.02696\mathrm{E}-10\mathrm{X}_1^2+1.19183\mathrm{E}-10\mathrm{X}_2^2+0.000372\mathrm{X}_3^2+0.000122\mathrm{X}_4^2\end{aligned}$$

**Table 7:** ANOVA of TWR for (CK45).

Source	SS	DF	MS	F value	*P* value	(% Contribution) or sensitivity
M	3.138E-08	14	2.242E-09	1920.53	< 0.0001 (significant)	99.93
X_1_	3.668E-09	1	3.668E-09	3142.61	< 0.0001	11.68
X_2_	1.134E-11	1	1.134E-11	9.72	0.0071	0.43
X_3_	6.605E-10	1	6.605E-10	565.86	< 0.0001	2.1035
X_4_	1.166E-10	1	1.166E-10	99.90	< 0.0001	0.371
X_1_^2^	1.808E-08	1	1.808E-08	15490.16	< 0.0001	57.57
X_2_^2^	1.522E-09	1	1.522E-09	1303.95	< 0.0001	4.84
X_3_^2^	1.923E-09	1	1.923E-09	1647.90	< 0.0001	6.12
X_4_^2^	6.583E-10	1	6.583E-10		< 0.0001	2.1
X_1_X_2_	2.228E-10	1	2.228E-10	190.85	< 0.0001	0.71
X_1_X_3_	3.730E-09	1	3.730E-09	3195.91	< 0.0001	11.87
X_1_X_4_	1.238E-10	1	1.238E-10	106.04	< 0.0001	0.39
X_2_X_3_	2.045E-09	1	2.045E-09	1752.36	< 0.0001	6.51
X_2_X_4_	8.051E-10	1	8.051E-10	689.82	< 0.0001	2.56
X_3_X_4_	1.006E-09	1	1.006E-09	862.32	< 0.0001	3.203
Residual	1.751E-11	15	1.167E-12	564.00	< 0.0001	0.056
Lack of Fit	7.500E-15	10	7.500E-16	0.0002	1.0000 (Not significant)	
Pure Error	1.750E-11	5	3.500E-12			
Cor Total	3.140E-08	29				

After undergoing an ANOVA study, the mathematical equation for R_a_, represented by Eq. (6), was shown in Table 8. The equation is appropriate, according to the F-value of 274.20. The R^2^ = 0.9803 is fairly close to the ad.

justed R^2^ = 0.9925 as is the R^2^ = 0.9961. An acceptable signal is indicated by the S/*N* = 59.303. Conversely, P-value analysis revealed that the majority of values are ≤ 0.05, showing the importance of the comparable variables in the equation.

Table 8 indicates that nearly all components were not significant, with the exception of the quadratic terms of R_a_ (X_1_^2^, X_2_^2^, X_4_^2^) and the interaction terms X_1_X_2_, X_1_X_3_, X_1_X_4_, and X_3_X_4_. The linear impacts of X_1_, X_2_, and X_4_ were also not significant.

**Fig. 8:**
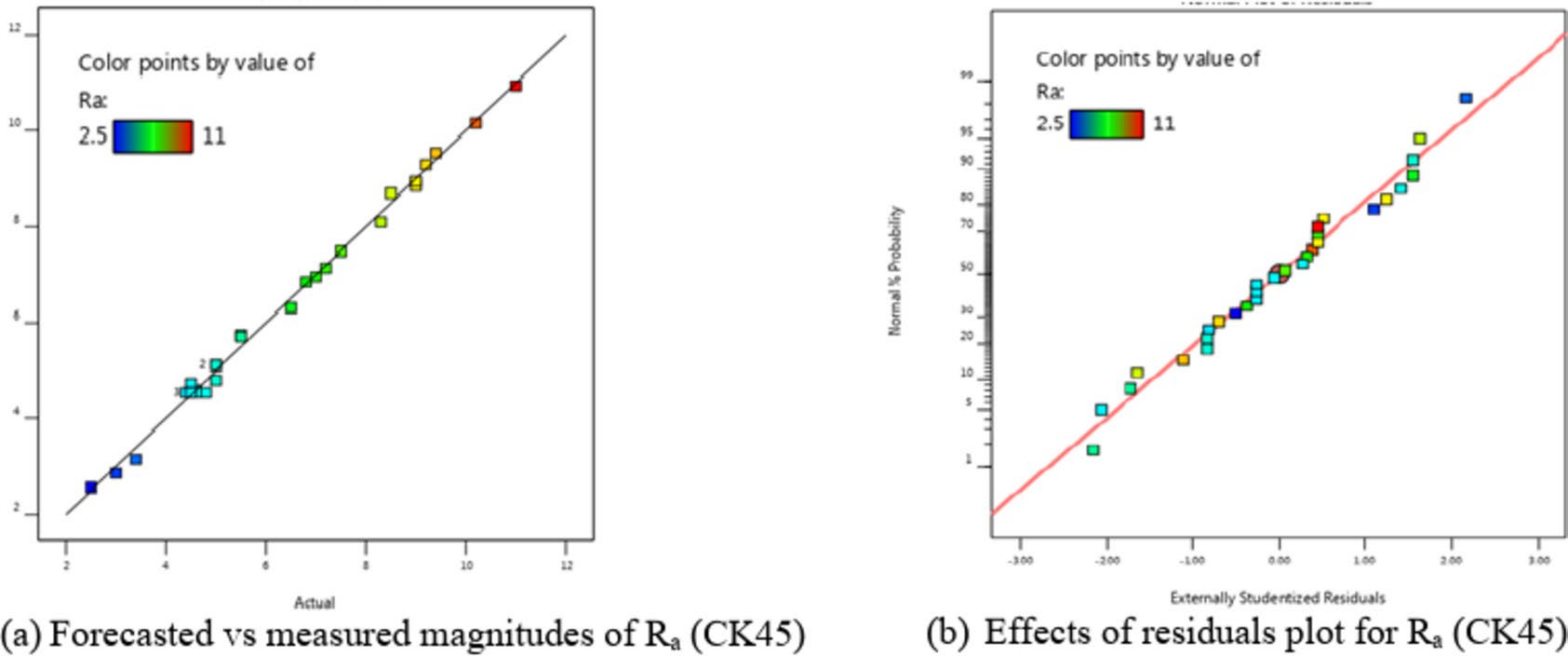
Schematic diagnostics of R_a_.

Figure 8 illustrates the satisfactory findings of the graphic diagnostics for R_a_. In addition, Fig. 8 (a) shows the values that were predicted vs. those that were measured, and Fig. 8 (b) shows the residuals. The data points on the residual graph were dispersed around the line, suggesting a strong correlation between the forecasted and trial values. The ANOVA results and graphic diagnostics clearly show how relevant the proposed mathematical equation is for R_a_.

6 $$\begin{aligned}Ra&= 79.60553 - 0.029448X_1 - 0.039763X_2 - 4.86111X_3 - 41.13750X_4 + 9.9E-06 X_1X_2 \\&- 0.002833X_1X_3 + 0.002875X_1X_4 +0.001667X_2X_3 -0.001750X_2X_4 + 14.58333X_3X_4 \\&+ 3.475E-06X_1^2 + 0.000011X_2^2 + 2.50000X_3^2 + 15.46875X_4^2 \end{aligned}$$

**Table 8:** ANOVA of R_a_ for (CK45).

Source	SS	DF	MS	F value	*P* value	(% Contribution) or sensitivity
M	153.12	14	10.94	274.20	< 0.0001(significant)	99.61
X_1_	51.04	1	51.04	1279.60	< 0.0001	33.20
X_2_	14.73	1	14.73	369.19	< 0.0001	9.58
X_3_	0.0067	1	0.0067	0.1671	0.6885	0.004
X_4_	22.04	1	22.04	552.58	< 0.0001	14.3
X_1_^2^	20.70	1	20.70	518.97	< 0.0001	13.5
X_2_^2^	12.73	1	12.73	319.13	< 0.0001	8.3
X_3_^2^	0.0868	1	0.0868	2.18	0.1609	0.05
X_4_^2^	10.50	1	10.50	263.26	< 0.0001	6.83
X_1_X_2_	24.50	1	24.50	614.27	< 0.0001	15.93
X_1_X_3_	0.7225	1	0.7225	18.11	0.0007	0.47
X_1_X_4_	1.32	1	1.32	33.15	< 0.0001	0.85
X_2_X _3_	0.0625	1	0.0625	1.57	0.2298	0.041
X_2_X_4_	0.1225	1	0.1225	3.07	0.1001	0.079
X_3_X_4_	3.06	1	3.06	76.78	< 0.0001	1.99
Residual	0.5983	15	0.0399			0.39
Lack of Fit	0.5033	10	0.0503	2.65	0.1469 (Not significant)	
Pure Error	0.0950	5	0.0190			
Cor Total	153.72	29				

Equation (7) presents the maximum roughness mathematical model (R_max_), which was evaluated by ANOVA analysis. The outcomes were compiled into Table 9. The proposed equation is significant, as demonstrated by the F-value of 30.98. Additionally, appropriate are the model fitting’s statistical indicators.

There is a reasonable disagreement between the adjusted R^2^ = 0.9354 and the R^2^ = 0.9666 and the expected R^2^ = 0.8226. Moreover, the S/*N* = 24.883 indicates a suitable signal. But several factors in the equation are not significant, as Table 9 makes clear.

**Fig. 9:**
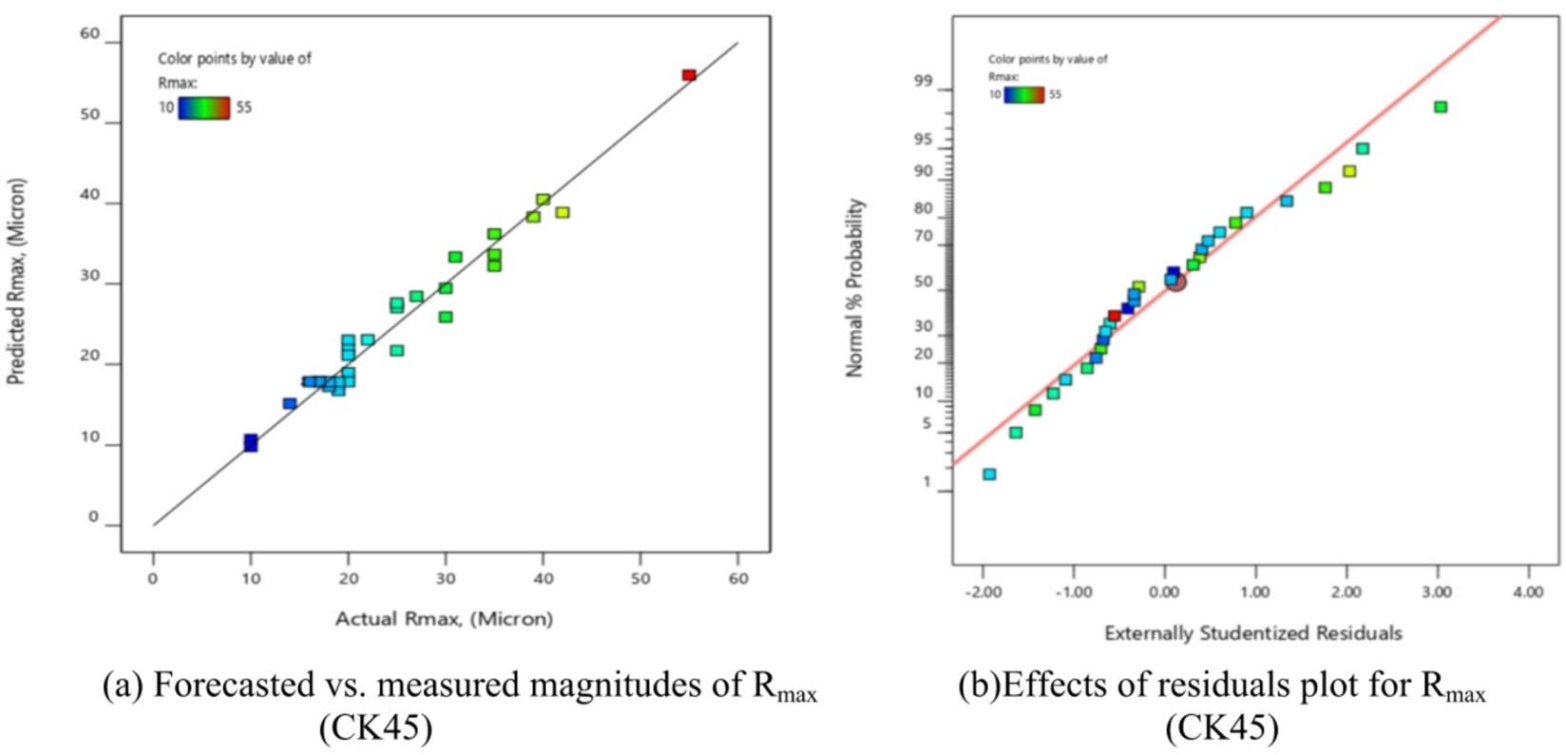
Schematic diagnostics of R_max_.

These consist of the lack of fit, the quadratic term of assistance (X_2_^2^), (X_3_^2^), (X_4_^2^), and the linear effects of X_4_. They also include the impact of the interaction effects (X_1_X_2_), (X_2_X_3_), and (X_3_X_4_). Figure 9 displayed the graphical diagnostics for R_max_.

Plotting the actual measurements vs., the forecasted magnitude and the studentized residuals against the normal % are the two main displays in Fig. 9. Based on these findings, the suggested mathematical model can be used to efficiently explore the design space.

7 $$\begin{aligned}\mathrm{R}\mathrm{m}\mathrm{a}\mathrm{x}&=345.14542-0.160208\mathrm{X}_1-0.034950\mathrm{X}_2+17.63889\mathrm{X}_3-193.02083\mathrm{X}_4+2.5\mathrm{E}-06\mathrm{X}_1\mathrm{X}_2-0.039167\mathrm{X}_1\mathrm{X}_3+0.039375\mathrm{X}_1\mathrm{X}_4\\&-0.025000\mathrm{X}_2\mathrm{X}_3+0.043750\mathrm{X}_2\mathrm{X}_4+47.91667\mathrm{X}_3\mathrm{X}_4+0.000025\mathrm{X}_1^2+0.000024\mathrm{X}_2^2+48.61111\mathrm{X}_3^2+8.59375\mathrm{X}_4^2\end{aligned}$$

**Table 9:** ANOVA of R_max_ for (CK45).

Source	SS	DF	MS	F value	*P* value	(% Contribution) or sensitivity
M	2980.38	14	212.88	30.98	< 0.0001(significant)	96.6
X_1_	1053.37	1	1053.37	153.28	< 0.0001	34.16
X_2_	135.38	1	135.38	19.70	0.0005	4.39
X_3_	176.04	1	176.04	25.62	0.0001	5.71
X_4_	22.04	1	22.04	3.21	0.0935	0.715
X_1_^2^	1060.74	1	1060.74	154.35	< 0.0001	34.4
X_2_^2^	59.17	1	59.17	8.61	0.0103	1.92
X_3_^2^	32.81	1	32.81	4.77	0.0452	1.06
X_4_^2^	3.24	1	3.24	0.4716	0.5027	0.1051
X_1_X_2_	1.56	1	1.56	0.2274	0.6404	0.051
X_1_X_3_	138.06	1	138.06	20.09	0.0004	4.47
X_1_X_4_	248.06	1	248.06	36.10	< 0.0001	8.044
X_2_X_3_	14.06	1	14.06	2.05	0.1731	0.456
X_2_X_4_	76.56	1	76.56	11.14	0.0045	2.483
X_3_X_4_	33.06	1	33.06	4.81	0.0445	1.072
Residual	103.08	15	6.87			3.342
Lack of Fit	92.25	10	9.23	4.26	(Not significant)	
Pure Error	10.83	5	2.17			
Cor Total	3083.47	29				

ANOVA analysis was used to evaluate the mathematical model for OR, illustrated through Eq. (8), and the data are presented in Table 10. The model was suitable, as evidenced by the obtained F-value of 26.10. There was a fair disagreement among the corrected R^2^ value of 0.9238 and the R^2^ of 0.9606. Furthermore, the S/*N* = 22.891 indicated a suitable signal. It was concluded that when the P-values of each term were changed, a few parts of the equation had very little effect.

**Fig. 10:**
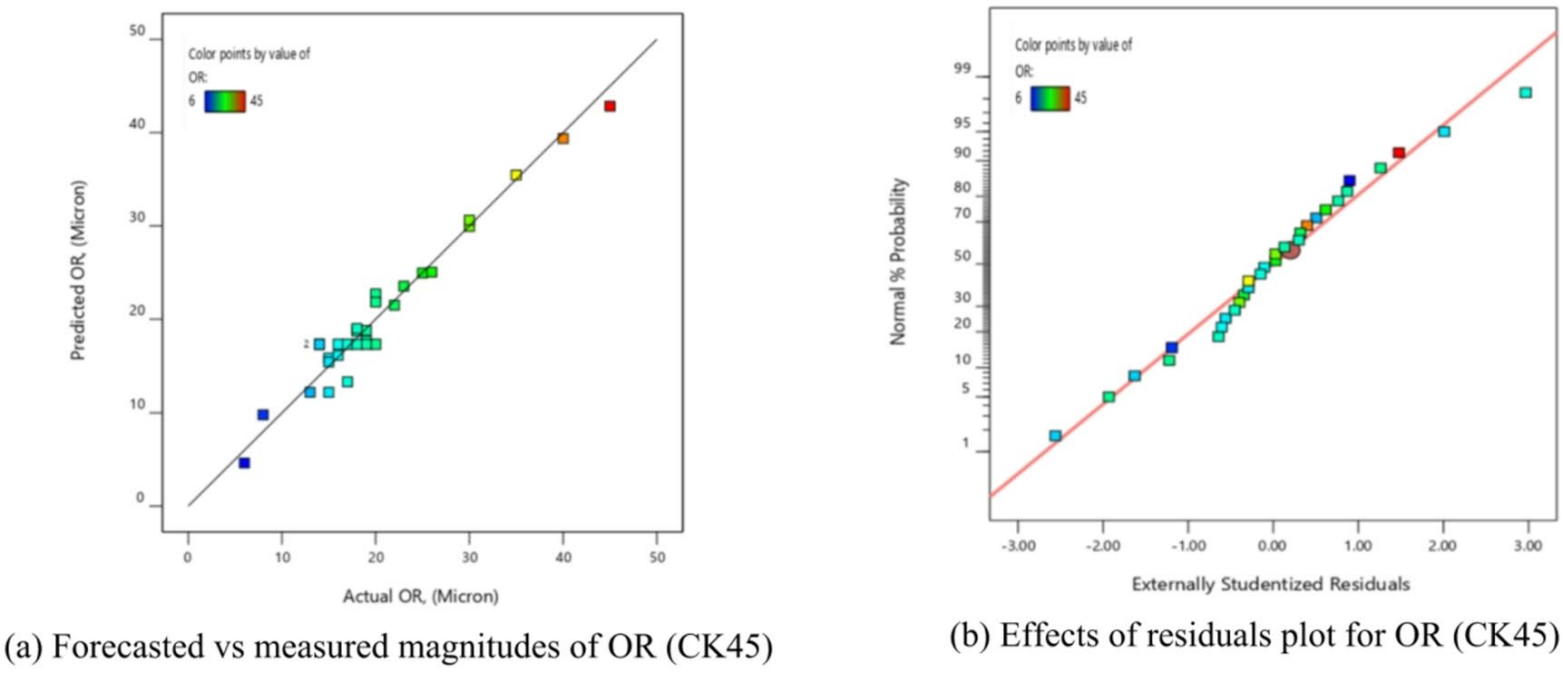
Schematic diagnostics of OR.

Table 10 shows that there is no significant similar among the quadratic form (X_2_^2^, X_3_^2^, and X_4_^2^) influence of X_2_, X_3_, and X_4_ and the interaction effects of (X_2_X_4_) and (X_3_X_4_). The pure error was more notable than the lack of fit, as demonstrated by the lowered Fit F-value of 26.10. The likelihood that noise would result in a drop in this variable’s Fit F-value was 22.891%. It was preferable to have a minimal decline in fitness. Furthermore, the schematic diagnostics for OR are shown in Fig. 10. The actual measurements were shown versus the predicted values for OR in this graph, which also displayed the residual effects versus the forecast values for OR. These results used to explore the design area in an efficient manner.

8 $$\begin{aligned}\mathrm{O}\mathrm{R}\hspace{0.17em}&=\hspace{0.17em}316.438-0.138\mathrm{X}_1+0.016\mathrm{X}_2-201.72\mathrm{X}_3-145.08\mathrm{X}_4-0.000023\mathrm{X}_1\mathrm{X}_2+0.07\mathrm{X}_1\mathrm{X}_3+0.0425\mathrm{X}_1\mathrm{X}_4\\&+0.053\mathrm{X}_2\mathrm{X}_3+0.005\mathrm{X}_2\mathrm{X}_4+12.5\mathrm{X}_3\mathrm{X}_4+0.000015\mathrm{X}_1^2+6\mathrm{E}-06\mathrm{X}_2^2-22.22\mathrm{X}_3^2+6.25\mathrm{X}_4^2\end{aligned}$$

**Table 10:** ANOVA of OR for (CK45).

Source	SS	DF	MS	F value	*P* value	(% Contribution) or sensitivity
M	2035.78	14	145.41	26.10	< 0.0001 (significant)	96.1
X_1_	661.50	1	661.50	118.71	< 0.0001	31.21
X_2_	42.67	1	42.67	7.66	0.0144	2.01
X_3_	24.00	1	24.00	4.31	0.0556	1.132
X_4_	2.67	1	2.67	0.4786	0.4996	0.125
X_1_^2^	385.71	1	385.71	69.22	< 0.0001	18.199
X_2_^2^	3.86	1	3.86	0.6922	0.4185	0.1821
X_3_^2^	6.86	1	6.86	1.23	0.2848	0.32
X_4_^2^	1.71	1	1.71	0.3076	0.5873	0.08
X_1_X_2_	132.25	1	132.25	23.73	0.0002	6.24
X_1_X_3_	400.00	1	400.00	71.78	< 0.0001	18.8
X_1_X_4_	289.00	1	289.00	51.86	< 0.0001	13.6
X_2_X_3_	64.00	1	64.00	11.49	0.0040	3.02
X_2_X_4_	1.00	1	1.00	0.1795	0.6778	0.047
X_3_X_4_	2.25	1	2.25	0.4038	0.5347	0.106
Residual	83.58	15	5.57			3.94
Lack of Fit	60.25	10	6.03	1.29	0.4105 (Not significant)	
Pure Error	23.33	5	4.67			
Cor Total	2119.37	29				

Table 11 presents ANOVA results analysis performed on the mathematical model for H, as represented by Eq. (9). Based on the computed F-value of 21.09, this model is considered significant. An F-value has only a 0.01% chance of arising from noise.

Model terms are clear significant when the P-value is less than 0.05. X_1_, X_2_, X_1_X_2_, X_1_X_3_, X_1_X_4_, X_2_X_3_, X_3_X_4_, X_1_^2^, X_2_^2^, X_3_^2^, and X_4_^2^ are important model terms in this instance. The model terms are deemed un significant if their values exceed 0.1000.

Model reduction might help the model if it has a lot of unnecessary terms. Given the pure error, the F-value for lack of fit, which is 0.50, indicates that the lack of fit is not substantial.

A significant Lack of Fit F-value has an 83.74% likelihood of being due to noise. A negligible mismatch is advantageous. There is less than 0.2 discrepancies among the Adjusted R² of 0.9065 and the forecasted R² of 0.8262, indicating a satisfactory agreement. The S/N ratio is measured with suitable precision. Ideally, the ratio should be higher than 4. A ratio of 16.687 indicates a strong signal, allowing this model to effectively navigate the design area.

**Fig. 11:**
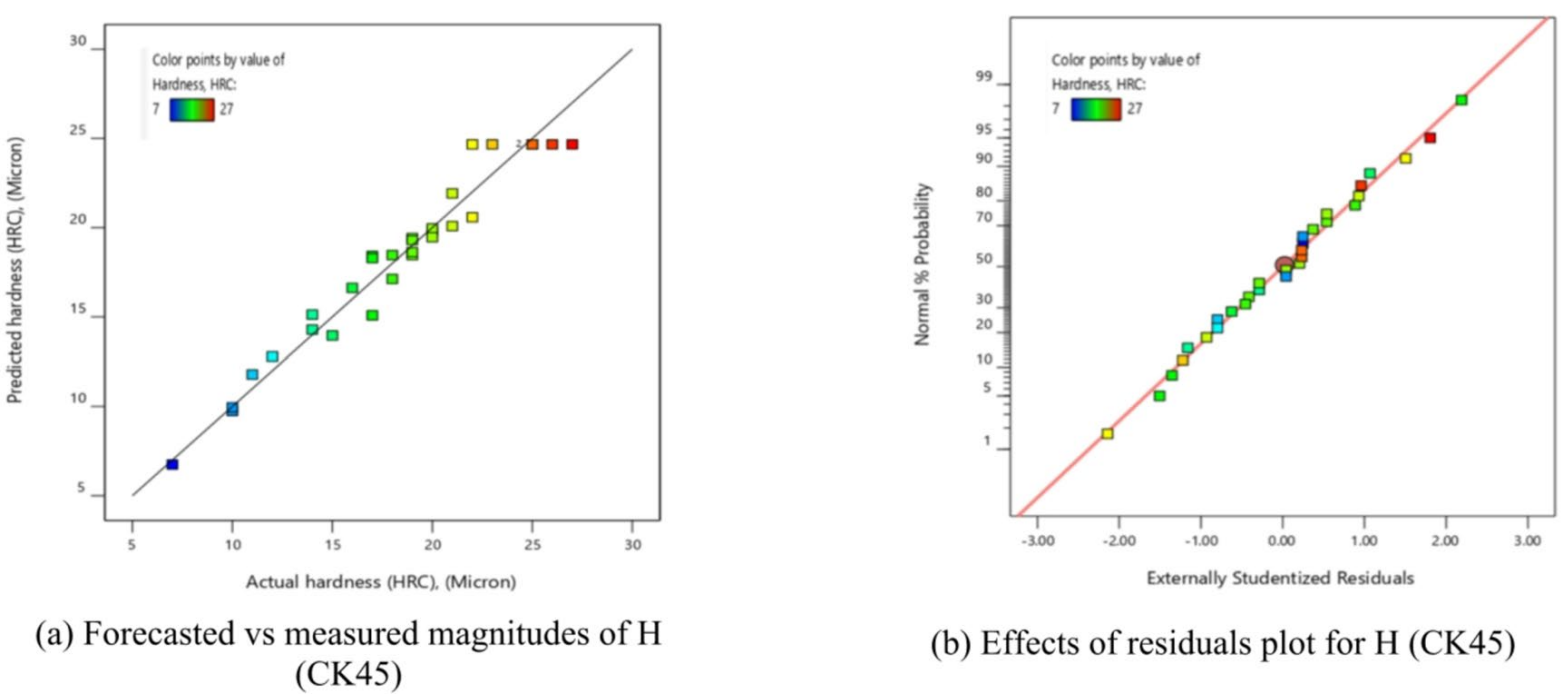
Schematic diagnostics of H.

Additionally, the diagnostic plot for H is presented in Fig. 11, illustrating actual measurements against predicted values for H, as well as residuals versus predicted values.

These findings can be applied to efficiently explore the design space.

9 $$\begin{aligned}Hardness, (H)&= -105.02810+0.072858X_1-0.053350X_2+117.28704X_3-15.1875X_4 +0.000023X_1X_2-0.022500X_1X_3+0.011875X_1X_4\\&+0.035X_2X_3-0.00125X_2X_4+35.41667 X_3X_4 -0.000013 X_1^2-0.000031X_2^2-64.35185 X_3^2-36.19792X_4^2 \end{aligned}$$

**Table 11:** ANOVA of H for (CK45).

Source	SS	DF	MS	F value	*P* value	(% Contribution) or sensitivity
M	680.88	14	48.63	21.09	< 0.0001 (significant)	95.2
X_1_	12.04	1	12.04	5.22	0.0373	1.7
X_2_	18.38	1	18.38	7.97	0.0128	2.56
X_3_	2.04	1	2.04	0.8855	0.3616	0.29
X_4_	1.04	1	1.04	0.4518	0.5117	0.15
X_1_^2^	302.86	1	302.86	131.36	< 0.0001	42.3
X_2_^2^	104.07	1	104.07	45.14	< 0.0001	14.54
X_3_^2^	57.50	1	57.50	24.94	0.0002	8.03
X_4_^2^	57.50	1	57.50	24.94	0.0002	8.03
X_1_ X _2_	138.06	1	138.06	59.88	< 0.0001	19.29
X_1_ X _3_	45.56	1	45.56	19.76	0.0005	6.36
X_1_ X_4_	22.56	1	22.56	9.79	0.0069	3.15
X_2_X_3_	27.56	1	27.56	11.95	0.0035	3.85
X_2_X_4_	0.0625	1	0.0625	0.0271	0.8714	0.0087
X_3_X_4_	18.06	1	18.06	7.83	0.0135	2.52
Residual	34.58	15	2.31			4.833
Lack of Fit	17.25	10	1.73	0.4976	0.8374 (Not significant)	
Pure Error	17.33	5	3.47			
Cor Total	715.47	29				

Using the derived equations, the trailing and predicted data for MRR, TWR, R_a_, R_max_, OR, and H for CK45 are shown in Figs. 6, 7, 8, 9, 10 and 11. The ensuing ANOVA analyses and figures for every response (MRR, TWR, R_a_, R_max_, OR, and H) for CK45 showed that the relationships between the responses and their conditions were clearly depicted by the equations (Eqs. 4, 5, 6, 7, 8 and 9), which had a significant influence. The significant coefficient values for detection (e.g., R^2^ = 0.9913 for MRR, R^2^ = 0.9994 for TWR, R^2^ = 0.9961 for R_a_, R^2^ = 0.9666 for R_max_, R^2^ = 0.9606 for OR, and R^2^ = 0.9517 for H) and the probability values being less than 0.05 confirmed this. Furthermore, residual value analysis was used to assess the advanced equations for MRR, TWR, R_a_, R_max_, OR, and H. The residual values for MRR, TWR, R_a_, R_max_, OR, and H were demonstrated by the curves shown in Figs. (6.b), (7.b), (8.b), (9.b), (10.b), and (11.b), in that order.

The residual values in these graphs (Figs. 6.a, 7.a, 8.a, 9.a, 10.a, and 11.a) were dispersed around a straight-line curve, suggesting a good fit among the forecasted and trial data for the relevant response relationships. Overall, the equations performed satisfactorily and showed no signs of unsuitability, according to the residual graph data for these responses.

These results collectively demonstrate that the developed regression models show strong statistical robustness and are well suited for both predictive analysis and process optimization. To further elucidate the relative importance of the machining parameters, a comprehensive sensitivity analysis was carried out to quantify the influence of the input variables N, F, D, and R on the six performance responses: MRR, TWR, Ra, R_max_, OR, and H.

This analysis provides valuable insights into the dominant parameters governing process performance and surface integrity during precision turning of CK45 workpieces.

The trained ANN model was employed to simulate the output responses by systematically varying one input parameter at a time while maintaining the remaining parameters at their respective median levels. The sensitivity index (SI) corresponding to each input–output relationship was subsequently evaluated using the following equation:

10 $$\mathrm{S}\mathrm{I}=\frac{\mathrm{M}\mathrm{a}\mathrm{x}.\mathrm{Y}\mathrm{i}-\mathrm{M}\mathrm{i}\mathrm{n}.\mathrm{Y}\mathrm{i}}{\mathrm{M}\mathrm{e}\mathrm{a}\mathrm{n}\mathrm{Y}}\times100 [37]$$

Where:

- Yi is the output when parameter iii is varied,
- Y is the overall mean of the response across all conditions.

This method allows for relative comparison of input influences.

**Fig. 12:**
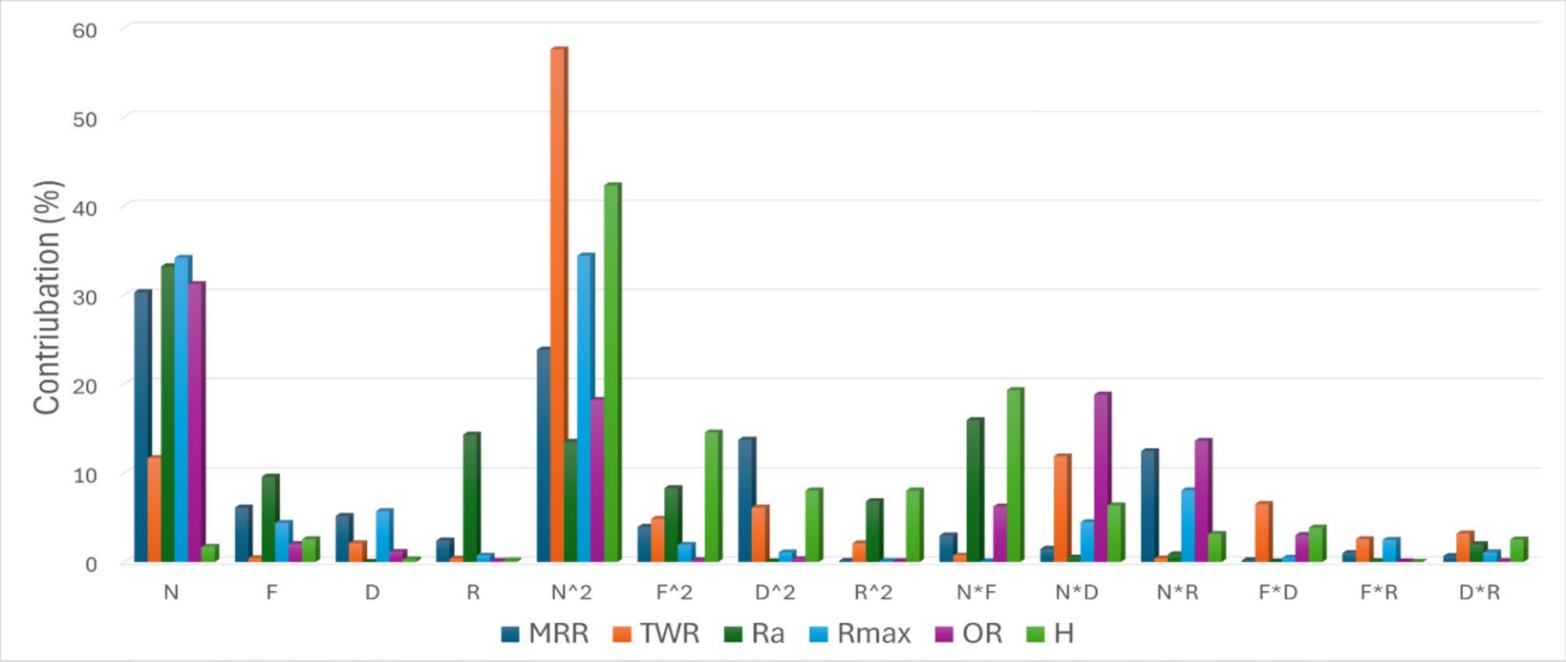
SI or contribution % of machining parameters on the responses of the TP for CK45.

From Fig. 12, TWR and H are mainly influenced by the quadratic term of N, with contributions exceeding 50% and 40%, respectively. N exhibits the largest effect on MRR, Ra, and OR, each with contributions above 30%. R_max_ is nearly equally affected by N and N². Interaction terms involving N (N×F, N×D, N×R) contribute more strongly than other factors. Overall, N is the most dominant input variable across the responses.

### Artificial neural network analysis

**Fig. 13:**
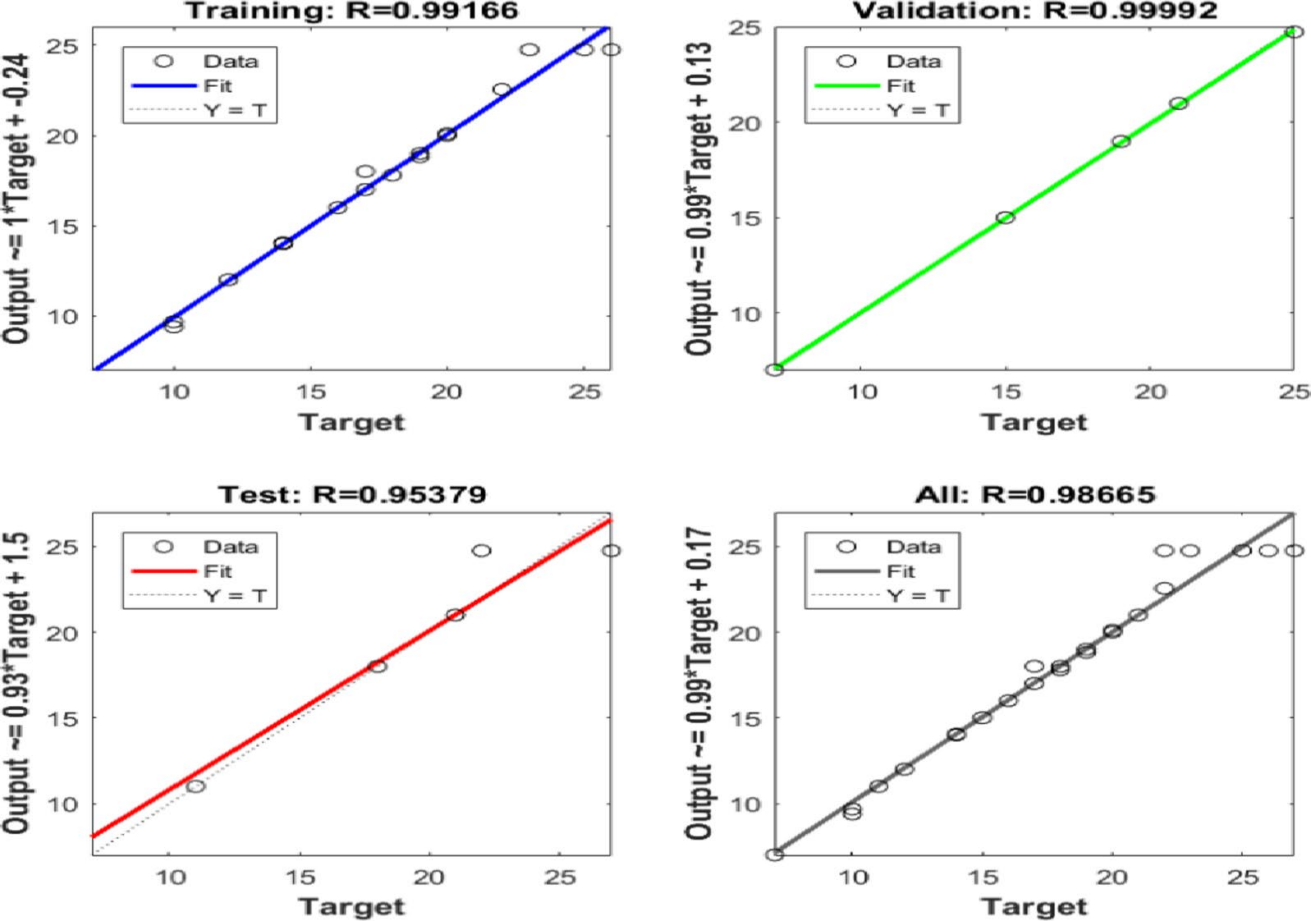
Regression of Training, Verification, Evaluation data sets for H.

**Fig. 14:**
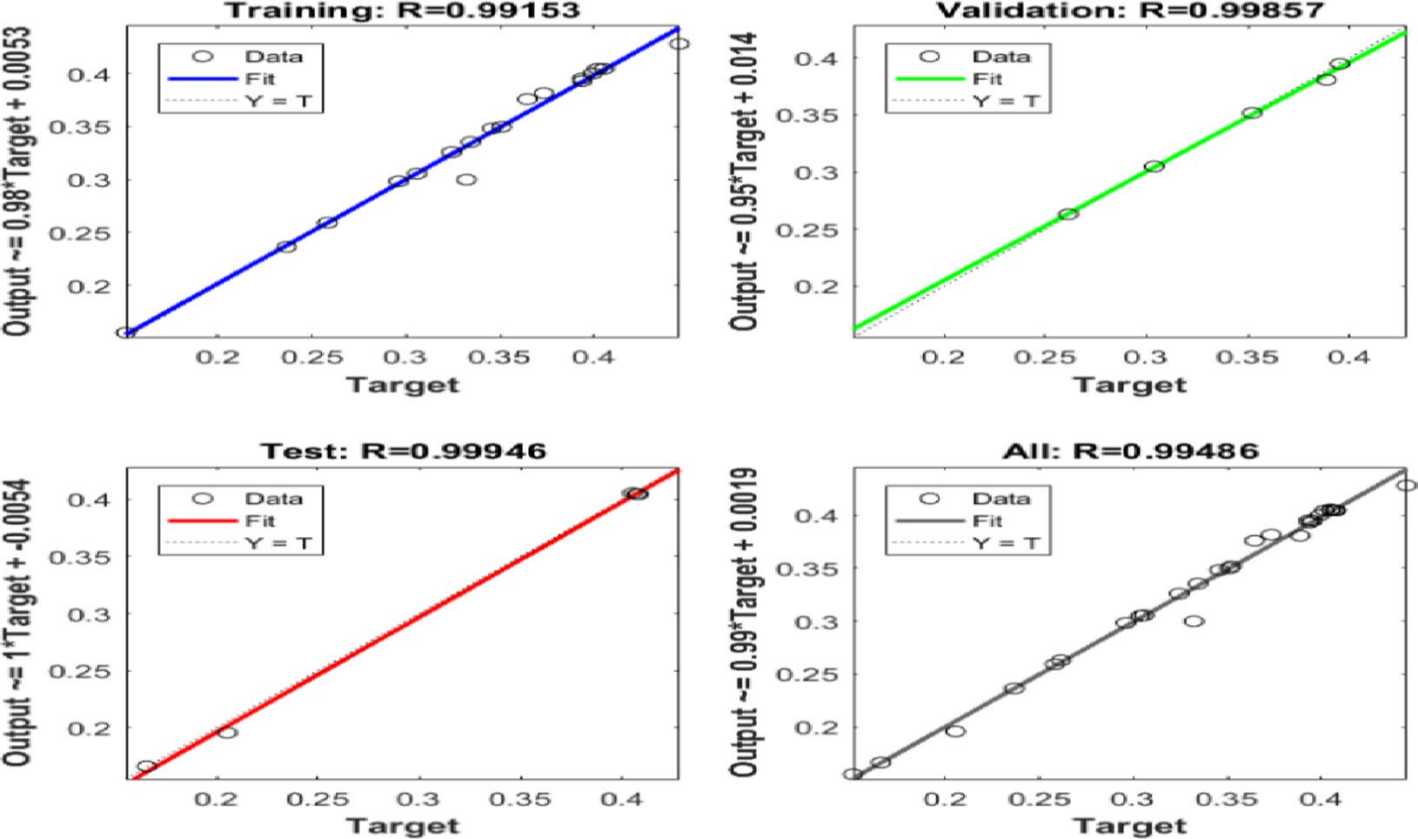
Regression of Training, Verification, Evaluation data sets for MRR.

**Fig. 15:**
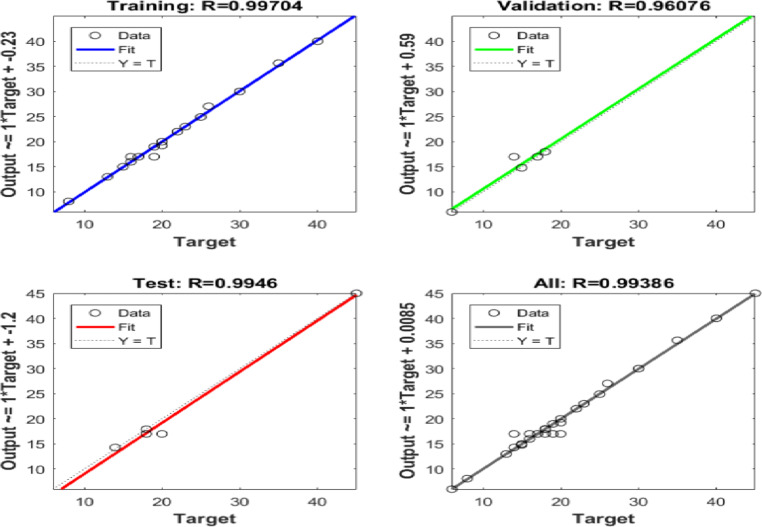
Regression of Training, Verification, Evaluation data sets for OR.

**Fig. 16:**
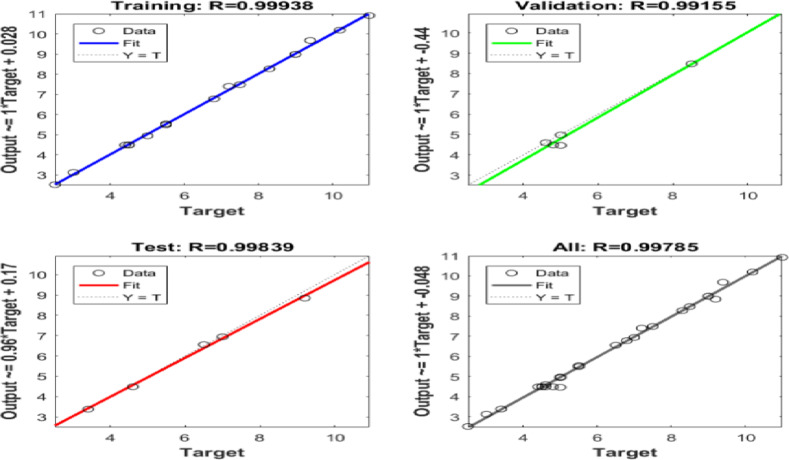
Regression of Training, Verification, Evaluation data sets for Ra.

**Fig. 17:**
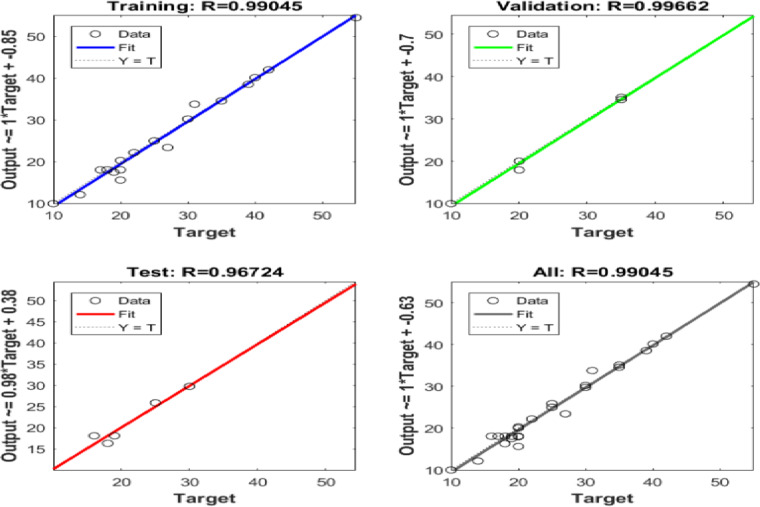
Regression of Training, Verification, Evaluation data sets for R_max_.

**Fig. 18:**
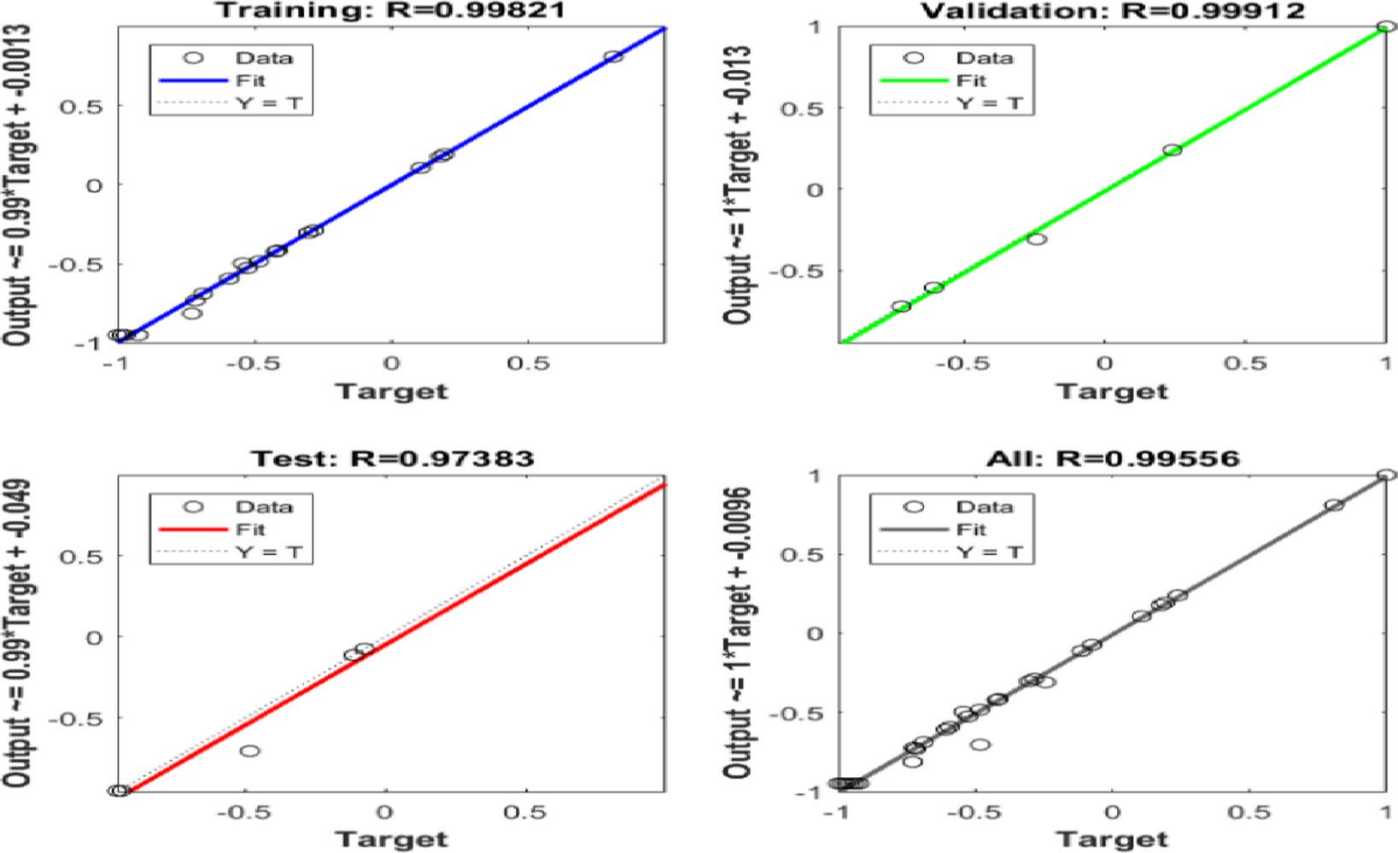
Regression of Training, Verification, Evaluation data sets for TWR.

Six ANN models were created to estimate the six output parameters MRR, TWR, Ra, R_max_, OR, and H using the experimental dataset. The performance of these models is presented in Figures (13 to 18). These plots display the linear relation among predicted and measured magnitudes, represented by straight lines. A clear alignment among the target and forecasted magnitude can be observed. Table 12 lists the regression results for training, validation, and testing of different responses. These magnitudes confirm the strong correlation and consistent reliability of the ANN forecasted across all datasets. The closeness of the fitted line to the Y = T line further validates the precision and stability of the models, highlighting the ANN’s strength in forecasting the output parametric from the input results. It is crucial to note that the Testing Dataset (15%) served as an independent dataset that was strictly excluded from the training phase. The high R-values observed in the ‘Evaluation’ column of Table 12 demonstrate the model’s capability to generalize and accurately predict responses for experimental conditions it has never seen before.

**Table 12:** Regression Values for Single Output ANNs in Training, Validation, and Evaluation.

Response	Training	Validation	Evaluation
H	0.99166	0.99992	0.95379
MRR	0.99153	0.99857	0.99946
OR	0.99704	0.96076	0.9946
Ra	0.99938	0.99155	0.99839
R_max_	0.99045	0.99662	0.96724
TWR	0.99821	0.99912	0.97383

**Fig. 19:**
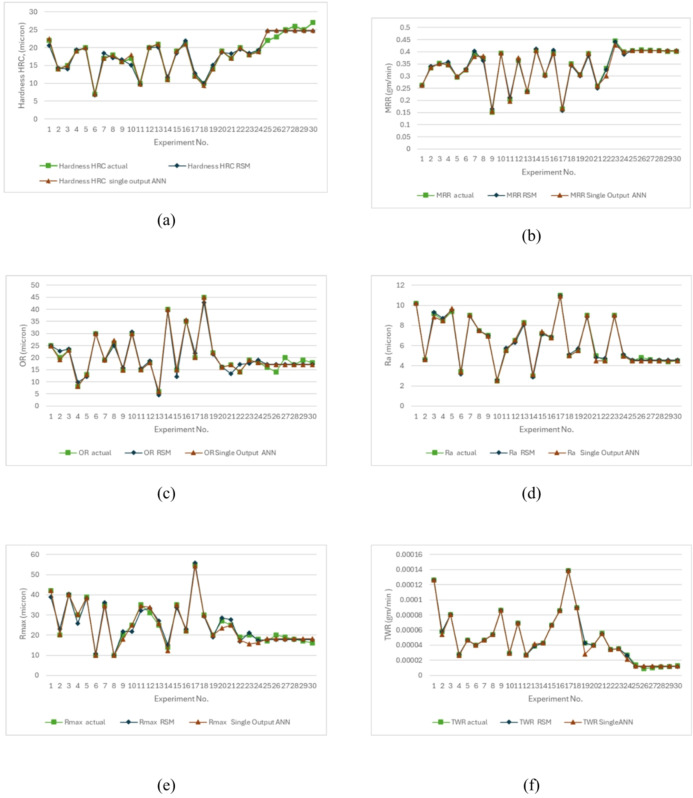
Actual magnitudes Vs Predicted magnitudes by RSM and ANN for (**a**) H (b) MRR(**c**) OR (**d**) Ra (**e**) R_max_ (f) TWR.

The forecasted capability of the regression model (RSM) was compared with that of the ANN model. Both models demonstrated good accuracy, but ANN produced higher correlations and lower errors for most responses than RSM. It proved more suitable for capturing complex characteristic, as illustrated in Fig. 19.

**Fig. 20:**
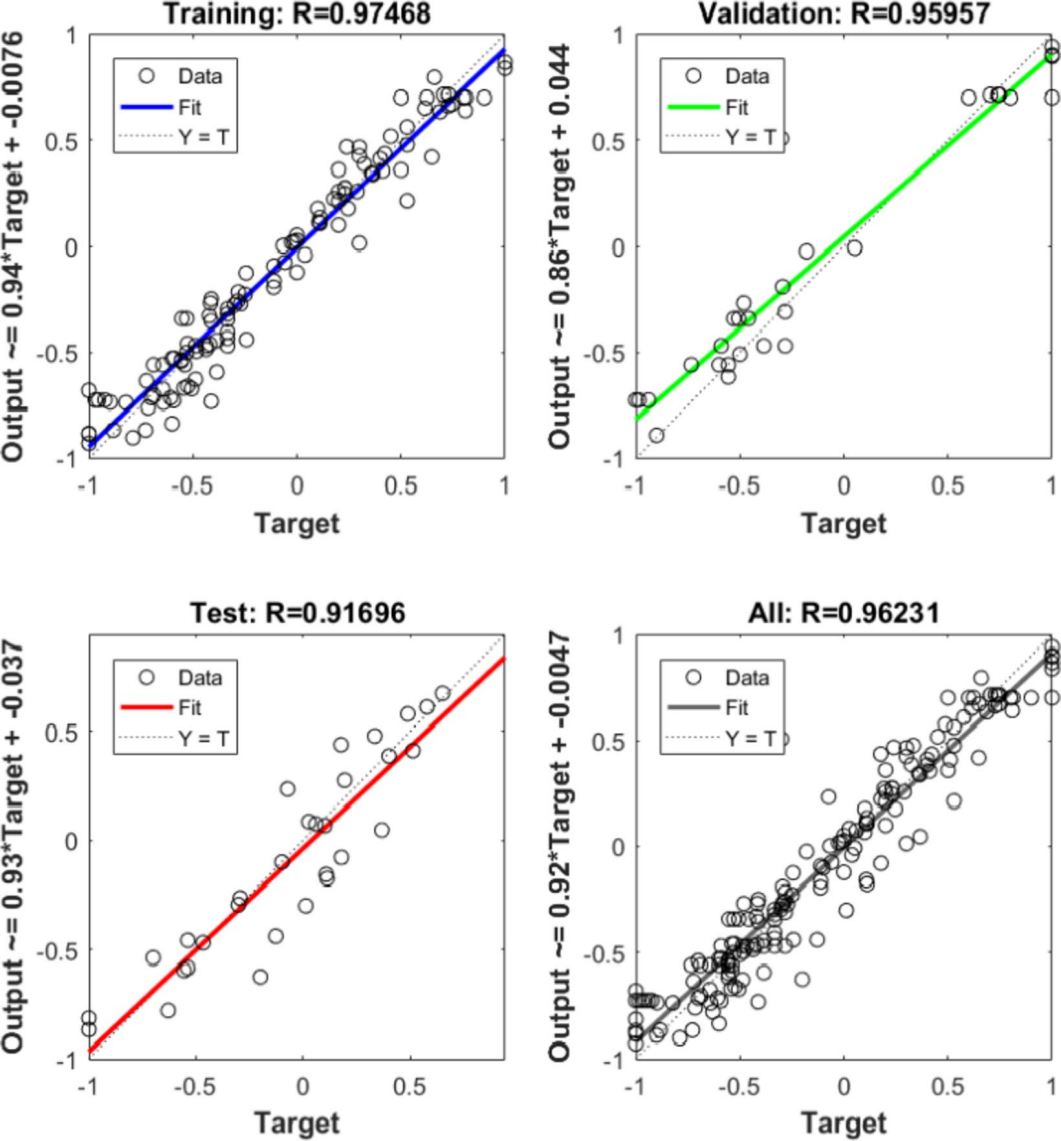
Regression of Training, Validation, Testing Data Sets for Multi-Output ANN.

**Fig. 21:**
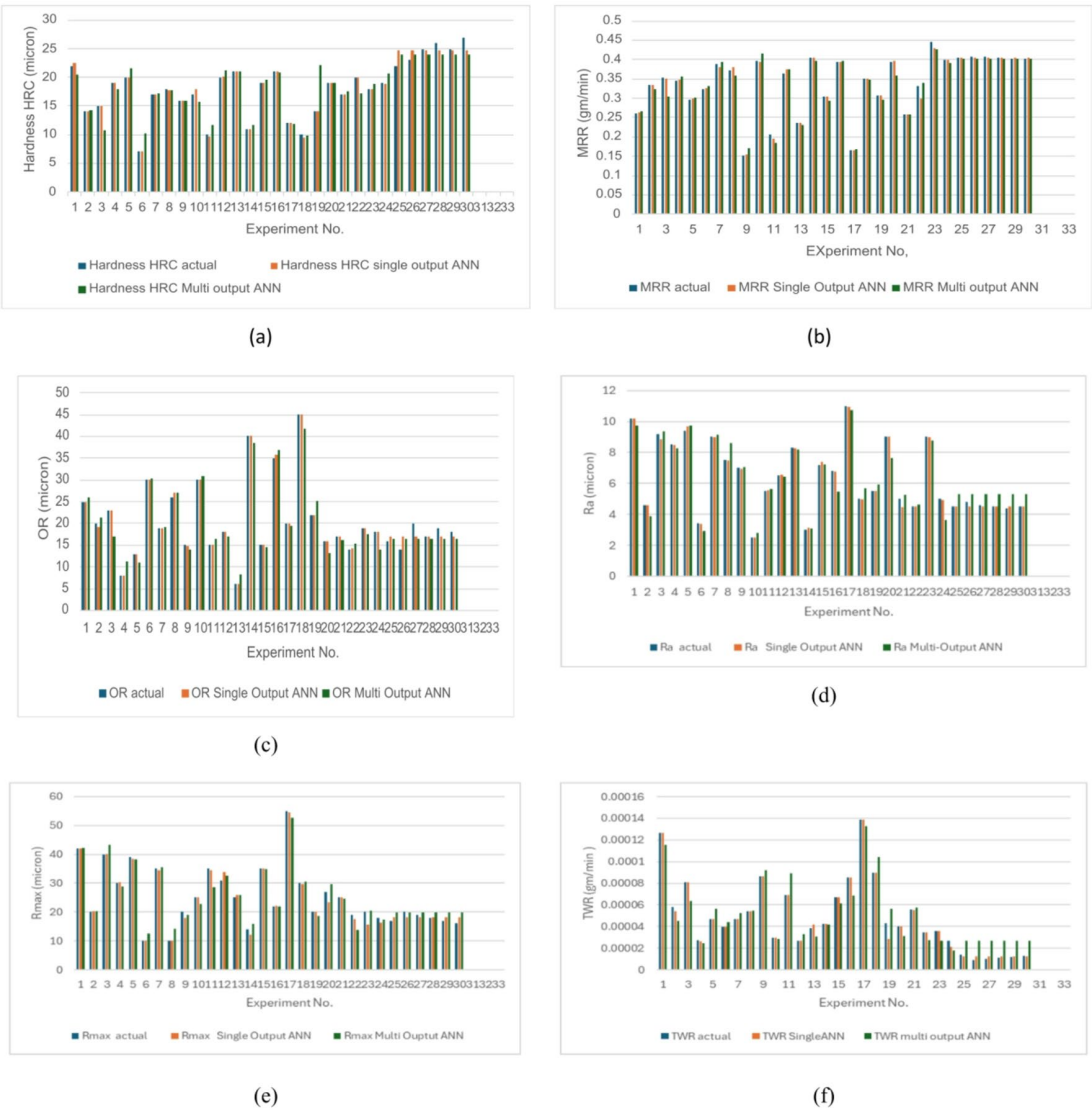
Actual Values Vs Predicted Values Using Single Output ANN and Multi Output ANN (**a**) H (**b**) MRR(**c**) OR (**d**) Ra (**e**) R_max_ (**f**) TWR.

A seventh model was designed to estimate four responses simultaneously by applying shared weights between the input and hidden layers. As presented in Fig. 20, this multi-output ANN achieved high regression values in all stages, reaching 0.97468 for training, 0.95957 for testing, and 0.91696 for validation. In contrast, Figure. 21(f) shows that TWR had very weak correlation with the experimental values, which is linked to its extremely small magnitudes close to zero.

The single-output ANN for the other responses displayed stronger correlation with experimental results compared to the multi-output ANN as shown in figures (21, a to f).

To assess the forecast performance of the three modeling strategies (RSM, multi-output ANN, and single-output ANN), Table 13 provides a comparison using MAPE, MSE, and RMSE, calculated as follows in equations from 11 to 13:

11 $$\mathrm{M}\mathrm{A}\mathrm{P}\mathrm{E}=\frac{1}{\mathrm{n}}\sum_{\mathrm{i}=1}^{\mathrm{n}}\frac{\left|{\mathrm{y}}_{\mathrm{i}}-\widehat{{\mathrm{y}}_{\mathrm{i}}}\right|}{{\mathrm{y}}_{\mathrm{i}}}\mathrm{*}100$$

12 $$\mathrm{M}\mathrm{S}\mathrm{E}=\frac{1}{\mathrm{n}}\sum_{\mathrm{i}=1}^{\mathrm{n}}{\left({\mathrm{y}}_{\mathrm{i}}-\widehat{{\mathrm{y}}_{\mathrm{i}}}\right)}^{2}$$

13 $$\mathrm{R}\mathrm{M}\mathrm{S}\mathrm{E}=\sqrt{\frac{1}{n}\sum_{i=1}^{n}{\left({y}_{i}-\widehat{{y}_{i}}\right)}^{2}}$$

Here, y_i_ denotes the actual magnitude, ŷ_i_ represents the forecasted value, and n refers to the total number of experiments. All models demonstrated strong predictive ability, with MAPE values ≤ 10% across the three approaches, except for TWR in the multi-output ANN, which reached 40.23%. This large error led to rejecting TWR predictions from this model. This resulted from shared weights between inputs and hidden layers. The RSM model was effective in capturing overall patterns for all responses.

The single-output ANN achieved superior consistency and accuracy, maintaining MAPE values below 6% for all six responses. For every response except TWR, the single-output ANN showed lower MAPE than RSM. The multi-output ANN provided a more compact framework by estimating five responses together, though its accuracy was less stable. In summary, the findings emphasize the advantage of single-output ANN when high accuracy is needed, particularly for detecting fine variations in mechanical performance.

**Table 13:** MAPE, MSE, RMSE for ANN Single Output, Multi Output and RSM.

		MAPE (%)	MSE	RMSE
H	single output ANN	1.87	0.64	0.8
	Multi Output ANN	9.01	4.59	2.14
	RSM	4.65	1.53	1.07
MRR	single output ANN	1.25	0.00006	0.0077
	Multi Output ANN	3.19	0.0002	0.014
	RSM	1.91	0.00005	0.007
OR	single output ANN	2.46	0.87	0.93
	Multi Output ANN	10.71	4.9	2.21
	RSM	8.13	2.79	1.67
Ra	single output ANN	1.46	0.023	0.15
	Multi Output ANN	9.12	0.41	0.63
	RSM	2.22	0.02	0.14
R_max_	single output ANN	4.6	2.18	1.48
	Multi Output ANN	9.07	5.74	2.4
	RSM	6.64	3.44	1.85
TWR	single output ANN	5.54	$9.68*{10}^{-12}$	$3.11*{10}^{-6}$
	Multi Output ANN	40.24	$1.24*{10}^{-10}$	$1.12*{10}^{-5}$
	RSM	2.72	$5.84\mathrm{*}{10}^{-13}$	$7.64\mathrm{*}{10}^{-7}$

### Analysis and parametric influences

The procedure included examining the effects of different input operating conditions on process responses in the TP for CK45. These conditions included N (X₁=A) in revolutions per minute, F (X₂=B) in millimeters per minute, D (X₃=C) in millimeters, and R (X₄=D) in millimeters. The measured responses included MRR, TWR, Ra, R_max_, OR, and H.

A parametric analysis was conducted in the experiment to determine how these input parameters influenced the TP responses. Using RSM as a foundation (RSM quadratic equations), two-dimensional response graphs were generated to explore the relationship among the responses and input parameters. These graphs served as a valuable tool for visualizing how changes in input conditions affected the response surface. Additionally, they provided a deeper understanding of how the measured responses were influenced by the turning process parameters.

Interaction points on the graphs represented instances where the influences of independent factors combined in an unexpected manner, significantly impacting response variables.

Determining these interaction points is crucial in RSM, as they play a key role in optimization procedures. Overlooking them can result in inaccurate models and sub optimal outcomes, as demonstrated by the influence of parametrices on different reactions in diverse cases.

For instance, the MRR, TWR, Ra, R_max_, OR, and H responses of CK45 varied based on changes in machining parameters in TP. When no linear relationship exists between the independent factors and response variables, certain non-linearities may appear.

The small adjustments to independent variables can lead to disproportionate changes in the response. Non-linearities in RSM curves manifest as deviations from a straight-line plot or as curved patterns. These deviations can arise due to complex interactions among variables and physical constraints.

Recognizing and determining non-linearities is essential, as they significantly impact the model’s predictive accuracy and the precision of optimization results. If not properly considered, non-linearities can lead to incorrect conclusions or suboptimal process settings.

**Fig. 22:**
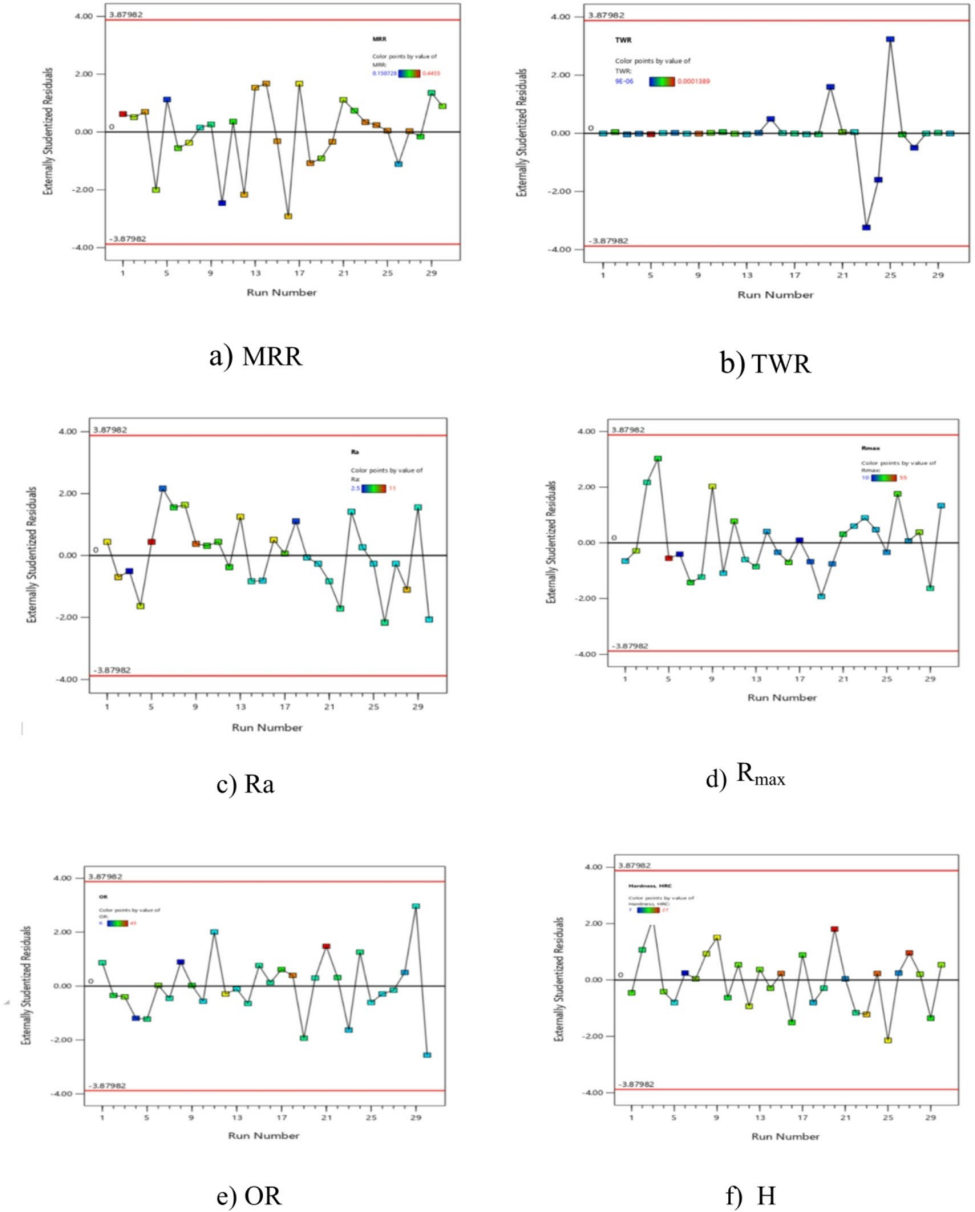
(Studentized residuals versus experimental run order) values of (MRR, TWR, R_a_, R_max_, OR and H) for (CK45).

Plots of studentized residuals vs. experimental run order for MRR, TWR, R_a_, R_max_, OR, and H are displayed in Figs. (22a to 22f), and they were utilized to look into how (TP) factors affected MRR, TWR, R_a_, R_max_, OR, and H during the CK45 experiments. The plots must show a random scatter with no observable patterns in order for this test to be considered valid.

The figures demonstrate that the patterns are in fact random and that there are no clear trends or patterns regarding the upper and lower bounds, suggesting a constant variance.

This provides more evidence of the suitability and dependability of the models that were created for the MRR, TWR, Ra, R_max_, OR, and H in the TP for CK45.

## Response surface analysis

### Behavior of turning process conditions on MRR

**Fig. 23:**
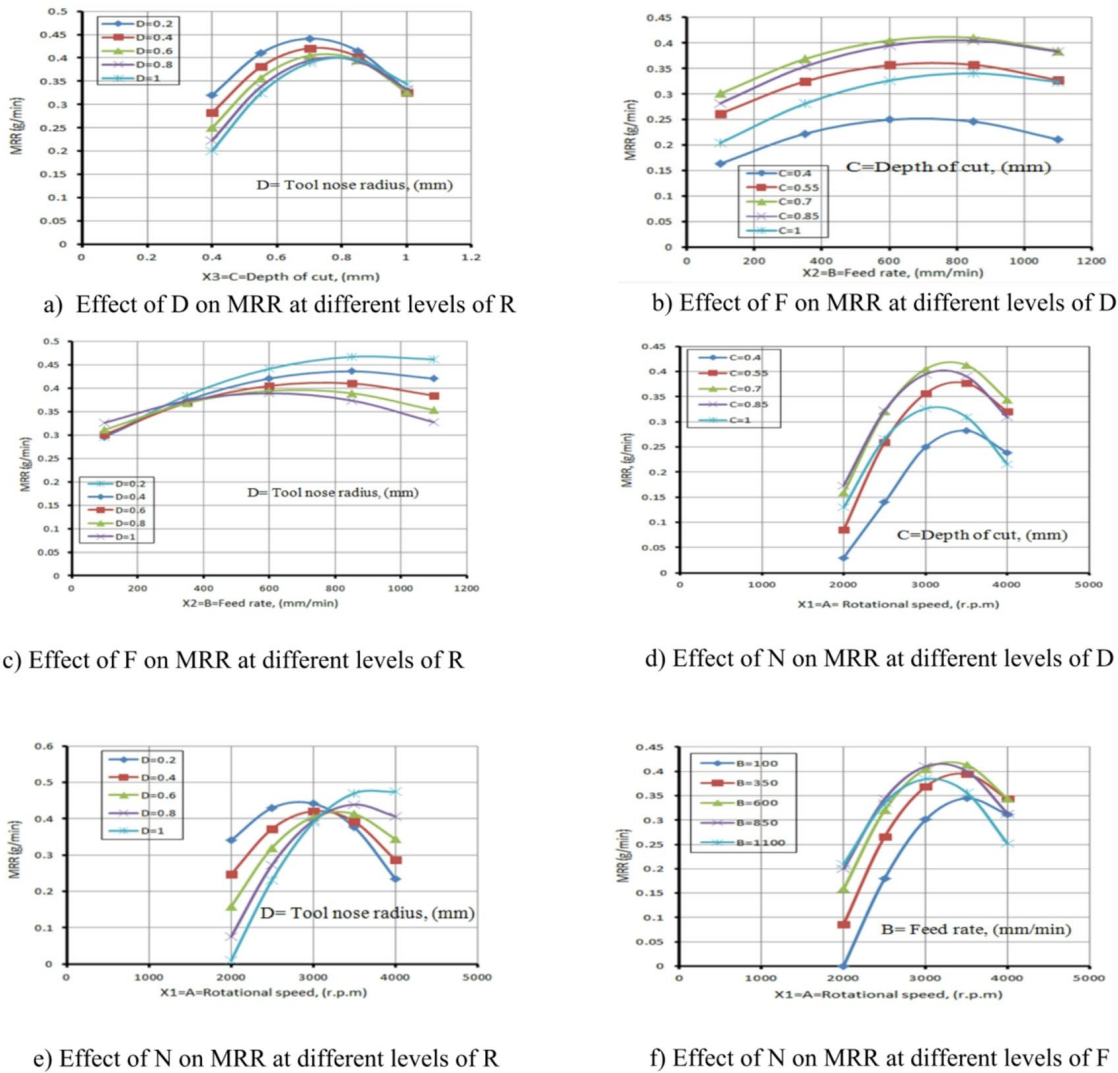
Influence of the different parameters on MRR.

The rate of change in volume determines MRR. As seen in the MRR main influence plot of Fig. 23, the MRR increases as N increases. This is because time plays important role in MRR, and at the same time, the number of turns increases at maximum speed.

MRR also rises as F increases, although it initially appears to increase slowly at lower F values. A similar trend is noted with the D. Since the maximum chip thickness can be removed, increasing D also raises MRR.

The influence on MRR is illustrated in Fig. 23a and f. These figures show that D, R, and F directly affect MRR. The MRR increases when D, F, and N are raised.

Figure 23a illustrates the relationship between the D and the MRR with varying R values. It was found that the MRR increases as D elevates from 0.4 mm to 0.7 mm; however, a further rise to 1 mm results in a decline in MRR.

This reduction is likely due to increased cutting forces leading to tool deflection and vibration, inefficient chip evacuation, elevated thermal loads, and accelerated tool wear all of which negatively impact cutting performance and reduce MRR beyond the optimal depth. This result is agreement with^12–14^. Figure 23b depicts the relationship among the F and the MRR at different values of D. It was found that as the F increases from 100 mm/min to 800 mm/min, the MRR increases due to the higher volume of material engaged by the tool per unit time. Conversely, when the F ranges from 800 mm/min to 1100 mm/min, the MRR begins to decrease.

This reduction is likely attributed to excessive cutting forces and tool vibrations at higher feed rates, which can reduce cutting stability and efficiency. Additionally, the tool may experience accelerated wear and thermal loading, leading to a loss in sharpness and ineffective material removal, ultimately causing a decline in MRR beyond the optimal F. This result is agreement with^38–41^.

Figure 23c illustrates the relationship among the F and the MRR at different tool radius (R) values. It was observed that when the feed rate ranged from 100 mm/min to 380 mm/min, the MRR increased slightly due to the gradual rise in the volume of material removed per unit time, while the variation in tool radius had a minimal effect in this range. However, as the F increased from 400 mm/min to 800 mm/min, the influence of the R became more significant. Larger tool radii likely enhanced the contact area and stability of the cutting process, contributing to improve MRR, whereas smaller radii might have limited the cutting engagement.

A point of intersection among the different R values was noted at F of 380 mm/min, possibly representing a transitional zone where the influence of tool radius begins to emerge.

At a higher F of 1100 mm/min, the effect of the R became more pronounced due to the intensified cutting forces and increased sensitivity of the process to tool geometry, which significantly impacted MRR either positively or negatively depending on the R value. This result is agreement with^12–14^.

Figures 23 (d to f) illustrate the relationship among N and the MRR at different values of D, R, and F. It was observed from Fig. 10d that as the N increases from 2000 r.p.m to 3000 r.p.m, the MRR also increases. This improvement is mainly due to the enhanced cutting action at higher speeds, which promotes efficient material shearing and better chip evacuation, resulting in a higher volume of material removed per unit time. However, as the N increases further from 3000 r.p.m to 4000 r.p.m, the MRR begins to decrease. This decline is likely attributed to the excessive heat generated at higher speeds, which may lead to thermal softening of the tool or workpiece, increased tool wear, and reduced cutting efficiency. Additionally, high-speed cutting can introduce instability and vibration, further compromising the MRR and overall process performance.

In Fig. 23e, it was elucidated that the MRR elevates with increasing N in the range of 2000 r.p.m to 3000 r.p.m with varying R values. This result is agreement with^38–41^.

This increase is attributed to improved cutting efficiency at higher speeds, where enhanced shear action and more effective chip evacuation lead to greater MRR.

However, the opposite effect occurs beyond this range, as the MRR begins to decrease with further increases in N from 3100 r.p.m to 4000 r.p.m.

This decline is likely due to excessive heat generation at high speeds, which may cause thermal softening of the tool or workpiece, accelerated TWR, and reduced cutting performance.

Additionally, increased N can introduce dynamic instability and vibration, especially when combined with specific tool geometries, further diminishing MRR.

Notably, at N of 3100 r.p.m, the MRR reaches 0.43 g/min, which represents the interaction and intersection point of Fig. 10e across the different R values indicating a transitional speed beyond which the influence of R becomes more pronounced and cutting performance begins to deteriorate. This result is agreement with^12–14^.

Figure 23f also shows the same effect and trend as Fig. 22d, depicting the relationship between N and MRR at different F values.

It was noted that the MRR increases with N in the range of 2000 r.p.m to 3000 r.p.m due to improved cutting efficiency, enhanced shear action, and more effective chip evacuation at moderate spindle speeds. However, as N increases further from 3100 r.p.m to 4000 r.p.m, the MRR begins to decline. This result is agreement with^38–41^.

This reduction is attributed to excessive heat generation at higher speeds, which can lead to thermal softening of the workpiece or tool, increased TWR, and reduced cutting performance.

Furthermore, high spindle speeds may cause instability and vibration, especially at certain feed rates, which adversely affect material removal. These combined effects explain the observed trend in both Fig. 10d and f. This result is agreement with^12–14^.

The ANOVA table for MRR is presented in Table 6. Among the parameters, N has the greatest impact on MRR, followed by F, D, and R. The relevance of the process parameters to the response variable is displayed in the ANOVA table.

Table 6 indicates that N and F have a greater impact on MRR than the other factors, which are negligible. If the p-values of the process parameters are less than 0.005 (α = 0.005) at a 99.13% confidence level, they are considered significant; otherwise, they are deemed inconsequential.

ANOVA results (Table 6) and diagnostic plots (Fig. 6) show N and Fas the most significant factors affecting MRR. Main effect plots (Fig. 23) link these trends to physical mechanisms: higher N and F improve shear action and chip evacuation, while excessive values cause tool wear, vibration, and thermal softening, reducing MRR. D and R also influence MRR by optimizing chip thickness and cutting stability. This combined statistical and mechanistic analysis explains the observed variations in MRR.

**Fig. 24:**
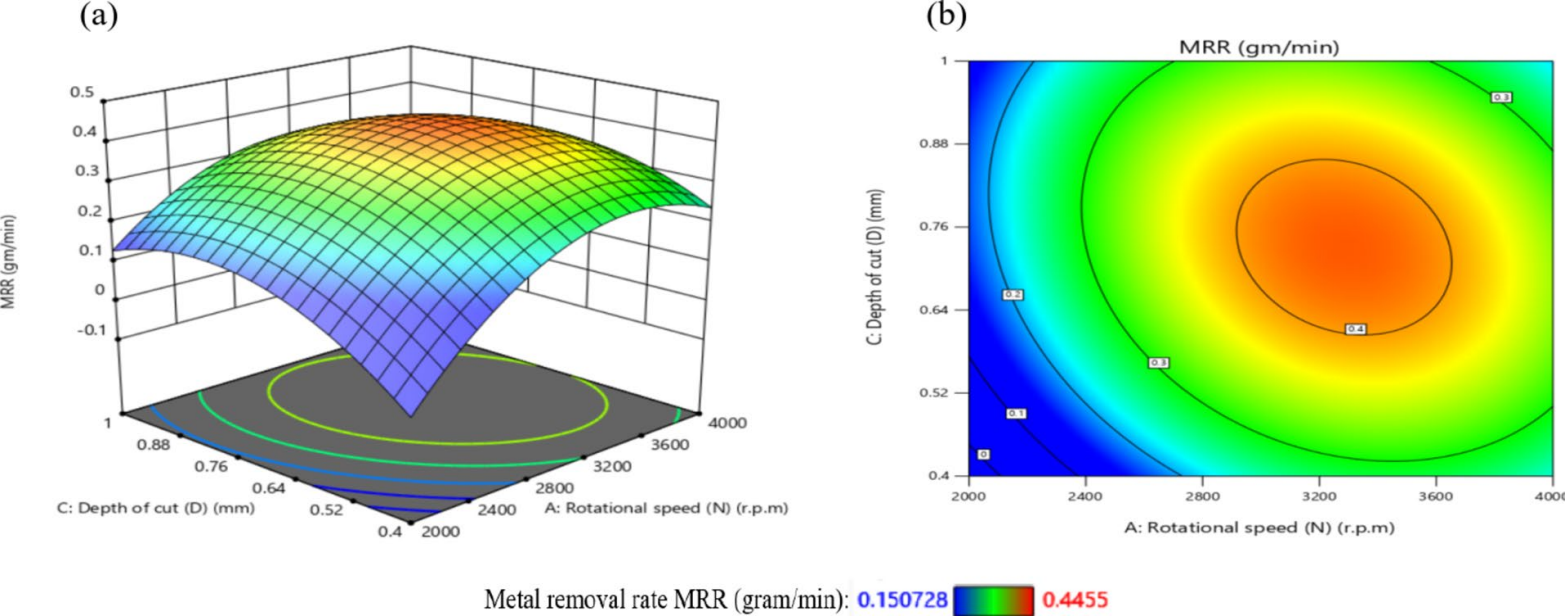
Interaction of N and D on MRR: (**a**) 3D surface; (**b**) contour plot.

The influence of significant interaction terms on the 3D surface and contour plot is examined using response surface analysis, as indicated by the analysis of variance results in Table 6. The impact of the N-D interaction on MRR is depicted in Fig. 24.

According to Fig. 24(a), MRR initially increases and then decreases as N or D increases, while the center values of the other components remain constant. The maximum MRR is 0.445 g per minute when *N* = 3200 r.p.m. and D = 0.76 mm.

The gradient in the direction of N is steeper than that in the direction of D, as shown in Fig. 24(b), suggesting that N has a greater impact on MRR than D. This result is agreement with^38–41^.

The minimum MRR is 0.15 g/min when D = 0.45 mm and *N* = 2200 r.p.m. Based on the interaction analysis, N should be between 3000 r.p.m and 3600 r.p.m, and D should be between 0.60 and 0.80 mm to achieve a maximum MRR of about 0.35 g/min.

**Fig. 25:**
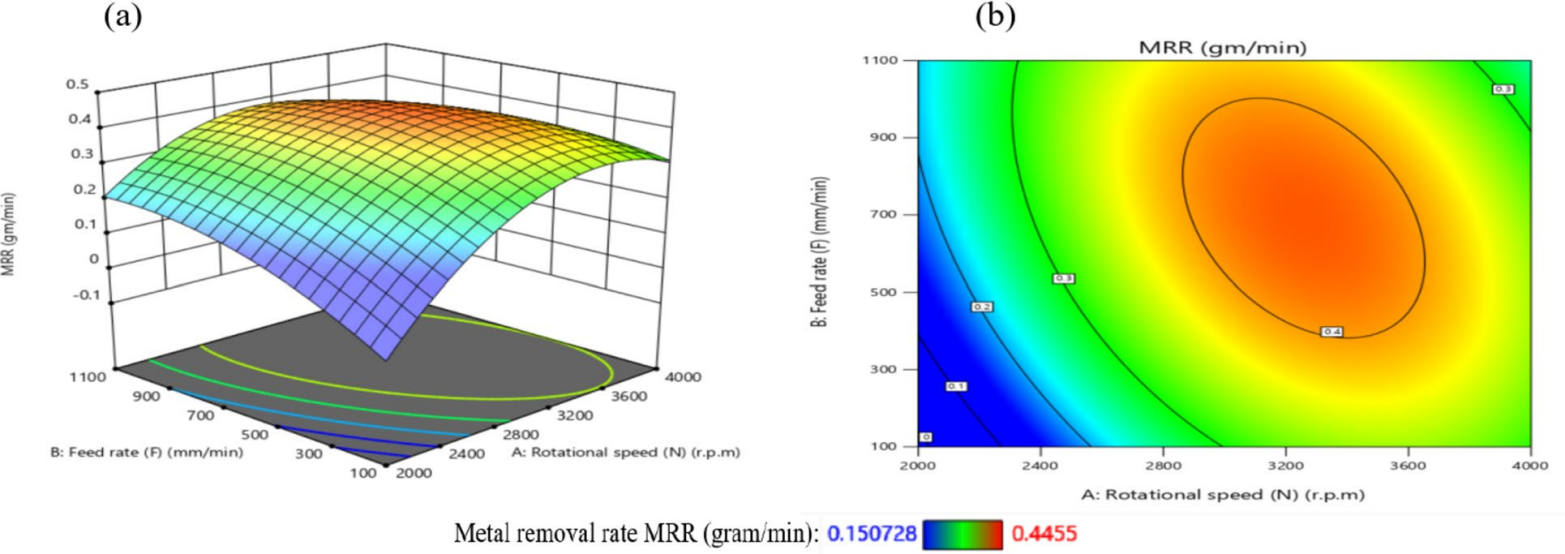
Interaction of N and F on MRR: (**a**) 3D surface; (**b**) contour plot.

The impact of the N-F interaction on MRR is presented in Fig. 25 (a and b). According to Fig. 25(a), When the N ranged from (2000 to 2600) r.p.m and the values of F were among (100 to 700) mm/min, the MRR reached its lowest levels, ranging from (0.1–0.2) g/min. Moreover, when the values of N ranged from 2800 r.p.m to 3800 r.p.m and the values of F were between (350 to 950) mm/min, the MRR reached its highest levels, ranging from (0.35 to 0.4455) g/min, while the center values of the other components remain constant. This result is agreement with^12–14^.

**Fig. 26:**
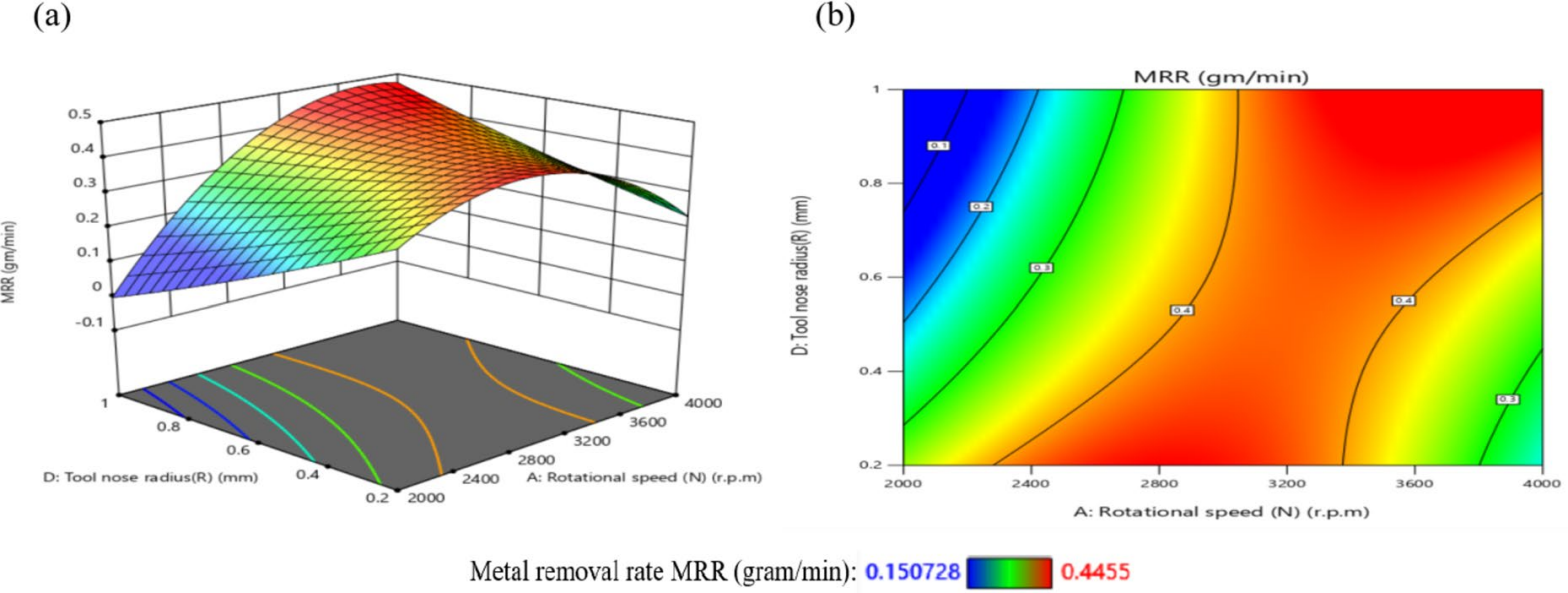
Interaction of N and R on MRR: (**a**) 3D surface; (b) contour plot.

The impact of the N-R interaction on MRR is depicted in Fig. 26. As illustrated in Fig. 26a, when the N ranged from 2000 r.p.m to 2400 r.p.m, the MRR reached its minimum levels. A significant increase in MRR was observed as N increased from 2400 r.p.m to 3600 r.p.m, reaching its peak values while keeping the center values of other components constant. At higher speeds ranging from 3600 r.p.m to 4000 r.p.m, MRR values stabilized at moderate levels.

Additionally, as shown in Fig. 26b, increasing the tool radius (R) from 0.6 mm to 1 mm at N range of 2000 r.p.m to 2400 r.p.m resulted in a reduction in MRR to 0.151 g/min.

Conversely, decreasing R from 0.6 mm to 0.2 mm while increasing N from 2400 r.p.m to 3600 r.p.m led to a notable rise in MRR, attaining a maximum value of 0.445 g/min. This result is agreement with^38–41^.

**Fig. 27:**
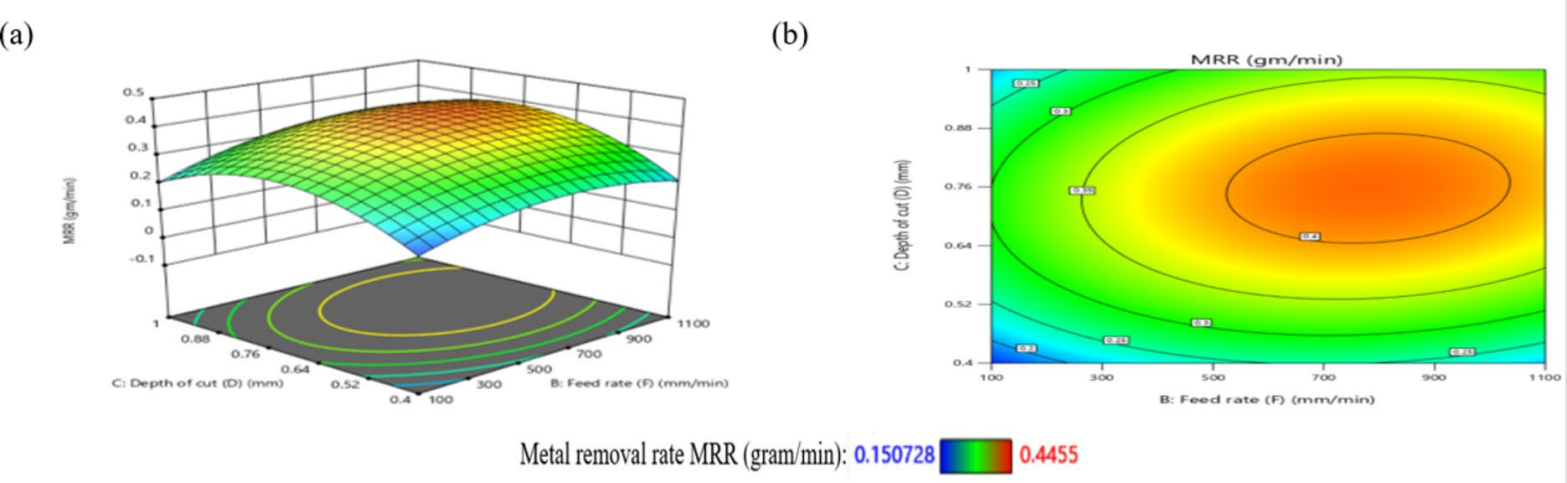
Interaction of F and D on MRR: (**a**) 3D surface; (**b**) contour plot.

The influence of the F-D relationship on MRR is presented in Fig. 27. When the F values range from 100 mm/min to 300 mm/min, MRR arrives its lowest level, at 0.15 g/min, while maintaining the central values of other parameters. However, it elevates and reaches its highest levels when the F ranges from 400 mm/min to 950 mm/min. It obtains moderate levels when the F ranges from 300 mm/min to 400 mm/min, as shown in Fig. 27a.

Meanwhile, when the D reduced from 0.52 mm to 0.4 mm at F of 100 mm/min to 300 mm/min, this led to a reduction in MRR to 0.151 g/min. Conversely, when the D values increased from 0.88 mm to 1 mm along with elevates in F from 100 mm/min to 300 mm/min, the MRR reached 0.25 g/min, as shown in Fig. 27b. Moreover, when the D ranged from 0.64 mm to 0.88 mm and the F ranged from 450 mm/min to 950 mm/min, the MRR reached its maximum magnitude of 0.4455 g/min. This result is agreement with^38^.

### Behavior of TP conditions on TWR

**Fig. 28:**
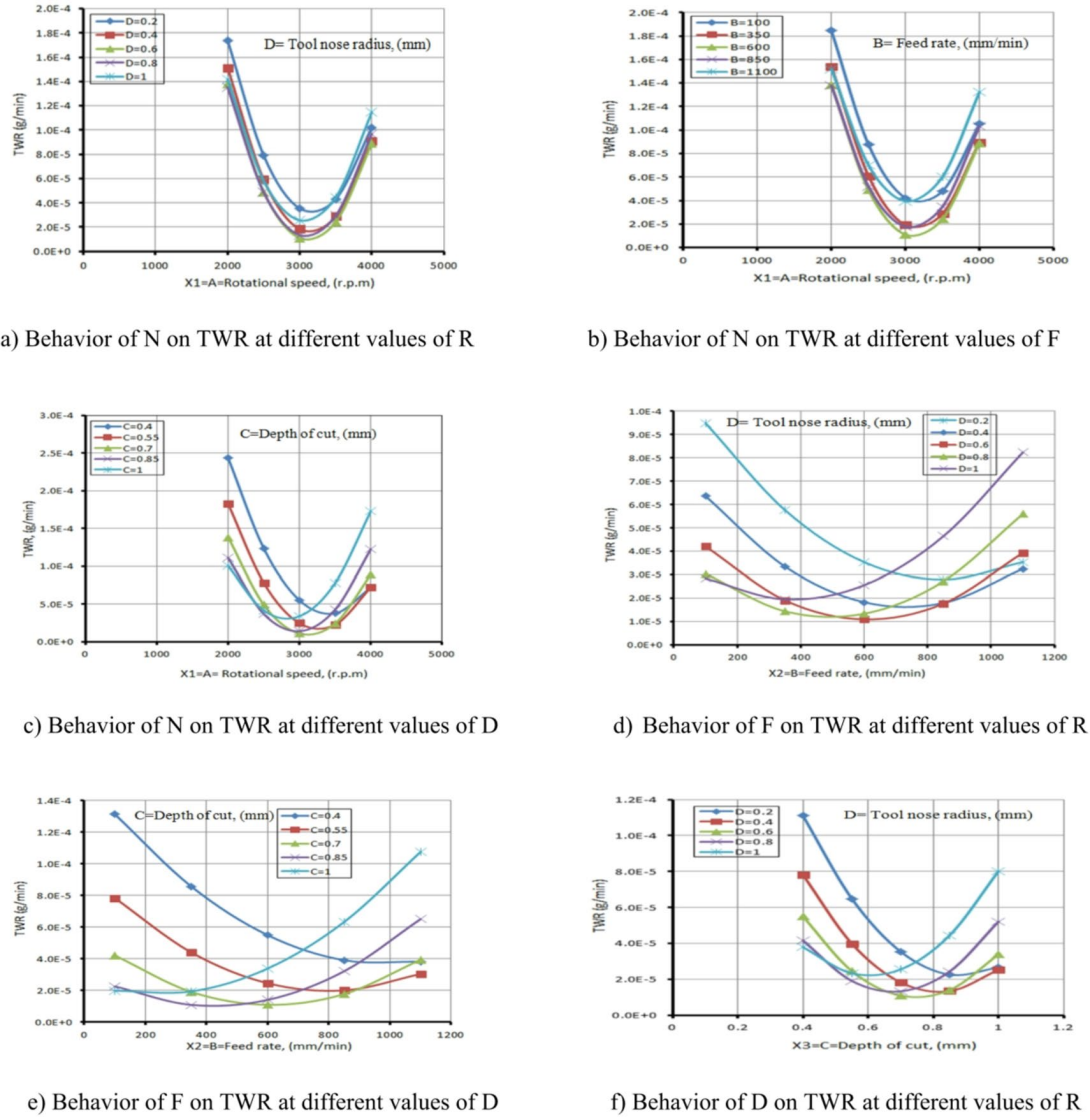
Influence of the various parameters on TWR.

The rate at which volume changes determines TWR. The TWR main effect plot of Fig. 28 shows that TWR increases as N increases. This occurs because the tool material softens at higher speeds due to the elevated temperature at the tool-workpiece interface, promoting diffusional, adhesive, and abrasive wear. Similarly, as F increases, tool wear also rises.

This happens because the cutting force per unit area at the chip-tool contact on the rake face and the work-tool contact on the flank face increases with F. Consequently, tool wear intensifies due to heightened cutting temperatures and mechanical shock.

Likewise, tool wear escalates as D increases. A larger D results in greater contact area, accelerating diffusion-type tool wear as well as adhesive and abrasive wear.

The figures (28a to 28c) illustrate the relationship between N and TWR at various values of R, F, and D. It is noted from Fig. 10a that as the N increases, the TWR decreases within the range of 2000 rpm to 3000 rpm. This result is agreement with^42^.

This reduction is primarily attributed to improved cutting efficiency, enhanced chip evacuation, and reduced friction at moderate N, which collectively lower the thermal and mechanical stresses on the tool.

However, when the N increases beyond 3000 rpm up to 4000 rpm, the TWR begins to rise again. This reversal is likely due to excessive heat generation and increased tool workpiece interaction at high speeds, leading to thermal softening and accelerated wear of the cutting edge. Similar trends are observed in Fig. 28b and c across different F and D, suggesting that the interplay between thermal effects and mechanical loading becomes more dominant at higher cutting speeds, regardless of other machining parameters. This result is agreement with^43^.

Meanwhile, Fig. 28d and e exhibit similar behavior, where the TWR decreases with an increase in F, at various values of R and D, within F range of 100 to 600 mm/min. This decrease in TWR can be explained by the shorter contact time between the tool and the workpiece at moderate F, which decreased heat accumulation and friction at the cutting edge. Additionally, the higher chip removal rates at these feed levels facilitate more efficient chip evacuation and improved cooling, both of which contribute to reducing TWR. However, when the F exceeds 600 mm/min and reaches up to 1100 mm/min, this trend reverses, and the TWR begins to increase. This result is agreement with^42,43^.

This is likely due to the significant rise in cutting forces and mechanical loads on the tool, along with insufficient time for proper heat dissipation and effective chip removal, leading to accelerated wear and deterioration in the tool’s cutting performance.

As for Fig. 28f, which depicts the relationship between D and TWR at different values of R, it exhibits the same behavior as in Fig. 28d and e. The turning point in behavior occurs at D equal to 0.7 mm.

Table 7 presents the ANOVA table for TWR, showing that N has the greatest effect on TWR, then D, F, and R. The relevance of process factors to the response variable is illustrated in the ANOVA table. Table 7 also indicates that N and D have a significantly greater effect on TWR than the other parameters, which are negligible. Process parameters with p-values below 0.005 (α = 0.005) at a 99.94% confidence level are considered significant, while those above 0.005 are deemed inconsequential. This result is agreement with^42^.

ANOVA results (Table 7) and diagnostic plots (Fig. 7) indicate that N and D have the most significant influence on TWR. The main effect plots (Fig. 28) link these trends to physical mechanisms: moderate N and F reduce TWR by improving cutting efficiency, chip evacuation, and reducing thermal and mechanical stresses, while higher N, F, or D increase TWR due to thermal softening, higher cutting forces, and accelerated wear.

R has a lesser effect but influences stability and contact area. This combined statistical and mechanistic interpretation explains the observed variations in TWR and validates the predictive model.

As D increases, the contact area also grows, accelerating diffusion-type tool wear as well as adhesive and abrasive wear.

The ANOVA table for TWR is presented in Table 7, which shows that N has the greatest effect on TWR, then D, F, and R. The significance of process factors on the response variable is demonstrated in the ANOVA table.

Table 7 indicates that N and D have a greater impact on TWR than the other parameters, which are negligible. If the p-values of process parameters are less than 0.005 (α = 0.005) at a 99.94% confidence level, they are considered significant; otherwise, they are deemed inconsequential.

**Fig. 29:**
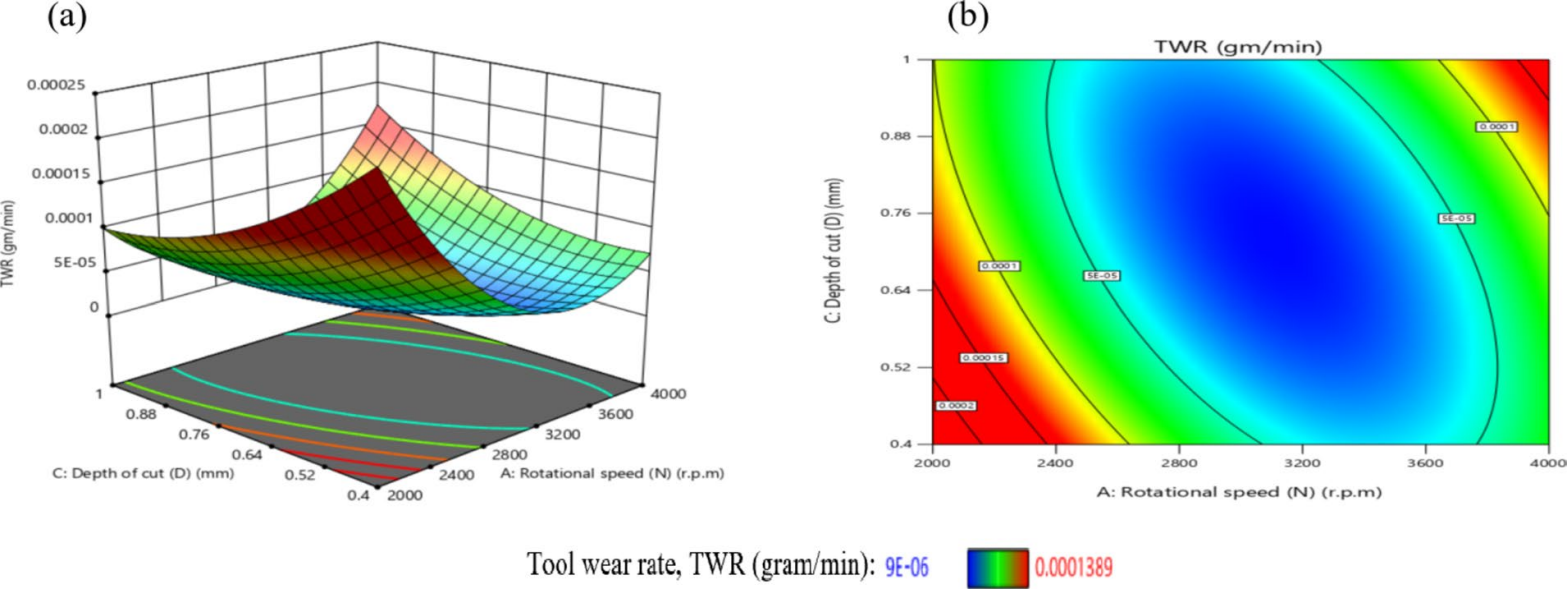
Interaction of N and D on TWR: (**a**) 3D surface; (**b**) contour plot.

The effect of N on TWR is greater than that of D, as shown in Figs. 29(a, and b), where the gradient in the direction of N is steeper than that in the direction of D. The minimum TWR is 9E-06 g per minute when N is 3200 r.p.m. and D is 0.76 mm.

The interaction analysis among the two suggests that D should range between 0.40 mm and 1 mm, while N should be between 3150 r.p.m. and 3850 r.p.m. to achieve a minimum TWR of less than 5E-05 g per minute. This result is agreement with^42^.

**Fig. 30:**
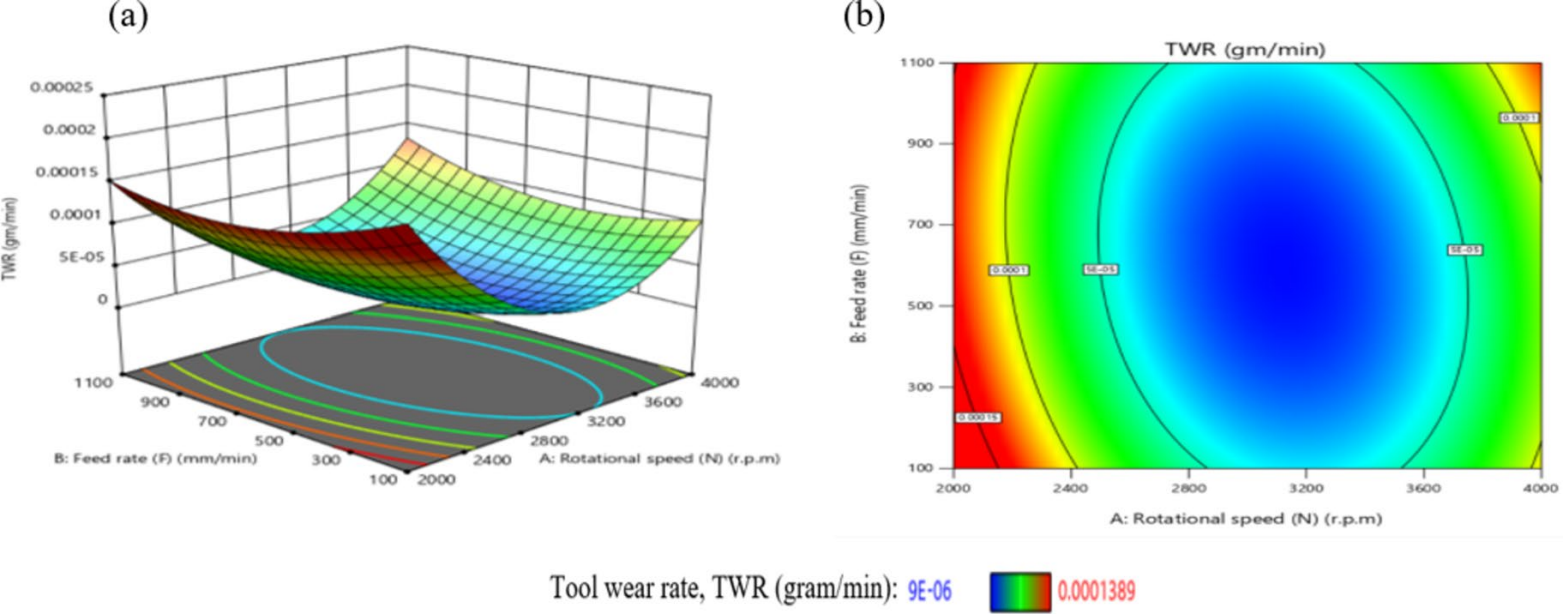
Interaction of N and F on TWR: (a) 3D surface; (b) contour plot.

The influence of the interaction among N and F on TWR is comprehensively presented in Fig. 30. As shown in Fig. 30(a), TWR exhibits a non-linear trend, initially declining and subsequently elevating with the rise of either N or F, while maintaining the remaining parameters constant. The highest TWR value, recorded at 1.85E-04 g/min, occurs at a low F of 100 mm/min and N of 2000 r.p.m. Furthermore, the response surface in Fig. 30(b) reveals a steeper gradient along the N-axis compared to the F-axis, highlighting the more pronounced effect of spindle speed over feed rate on TWR.

The minimum TWR, measured at 1E-05 g per minute, is attained when the F is 600 mm/min and N is 3200 r.p.m.

According to the interaction analysis, maintaining F within the range of 100–1100 mm/min and N between 2800 and 3600 r.p.m. yields optimal conditions for minimizing TWR. Within this operational window, TWR values consistently fall below 5E-05 g/min, indicating a favorable regime for reduced tool degradation. This result is agreement with^43^.

**Fig. 31:**
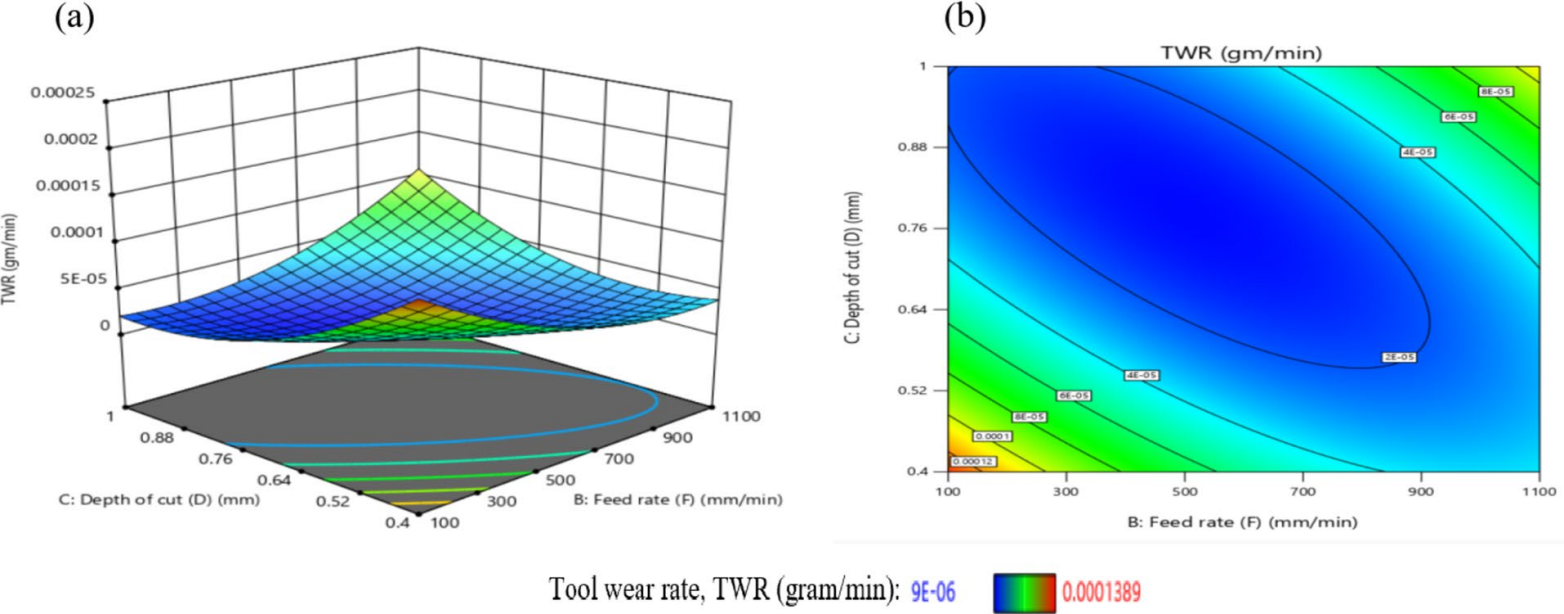
Interaction of F and D on TWR: (**a**) 3D surface; (**b**) contour plot.

The effect of the interaction between F and D on TWR is illustrated in Fig. 31. A moderate interaction is evident at D = 0.7 mm, where changes in F produce noticeable but not dominant variations in TWR. As shown in Fig. 31(a), when other parameters are held constant, increasing F leads to a reduction in TWR at higher depths of cut (D = 0.75 mm and 1.0 mm), while the opposite trend is observed at lower depths (D = 0.4 mm and 0.6 mm), where TWR increases with decreasing F. The maximum TWR, recorded at 1.3E-04 g/min, occurs under the combined conditions of F = 100 mm/min and D = 0.4 mm. Figure 31(b) further demonstrates that the influence of D on TWR is more substantial than that of F, as evidenced by the steeper gradient along the D-axis. The minimum TWR value of 2.0E-05 g per minute is achieved when F is 700 mm/min and D is 0.6 mm. Based on the interaction analysis, maintaining F within 100–900 mm/min and D between 0.60 and 1.00 mm is recommended to achieve TWR values consistently below 2E-05 g per minute, thus promoting enhanced tool life and machining efficiency. This result is agreement with^42^.

**Fig. 32:**
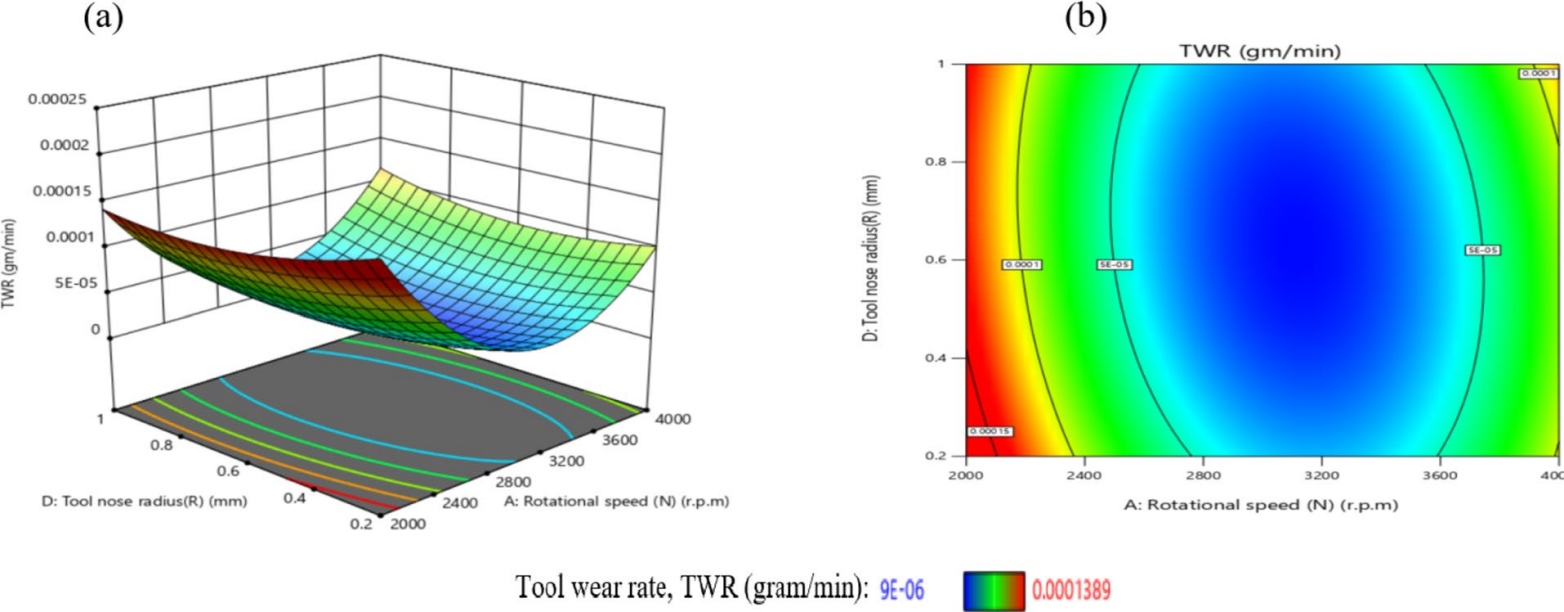
Interaction of N and R on TWR: (**a**) 3D surface; (**b**) contour plot.

The influence of the interaction between N and R on TWR is illustrated in Fig. 32. As depicted in Fig. 32(a), TWR exhibits a non-monotonic trend, initially declining and subsequently increasing with rising values of either N or R, while holding other parameters constant. The highest TWR, recorded at 1.389E-04 g per minute, occurs at a low N of 2000 r.p.m. and a minimal R of 0.2 mm.

Figure 32(b) reveals that the gradient along the N-axis is steeper than that along the R-axis, indicating a stronger influence of N on TWR compared to R. Conversely, the lowest TWR value of 9E-06 g per minute is attained when R is 0.6 mm and N is 3000 r.p.m. Based on the interaction analysis, maintaining N within 2800–3600 r.p.m. and R within 0.2–1.0 mm is recommended to achieve TWR values consistently below 5E-05 g per minute, thereby enhancing tool longevity and machining performance. This result is agreement with^43^.

### Behavior of turning process conditions on R_a_

**Fig. 33:**
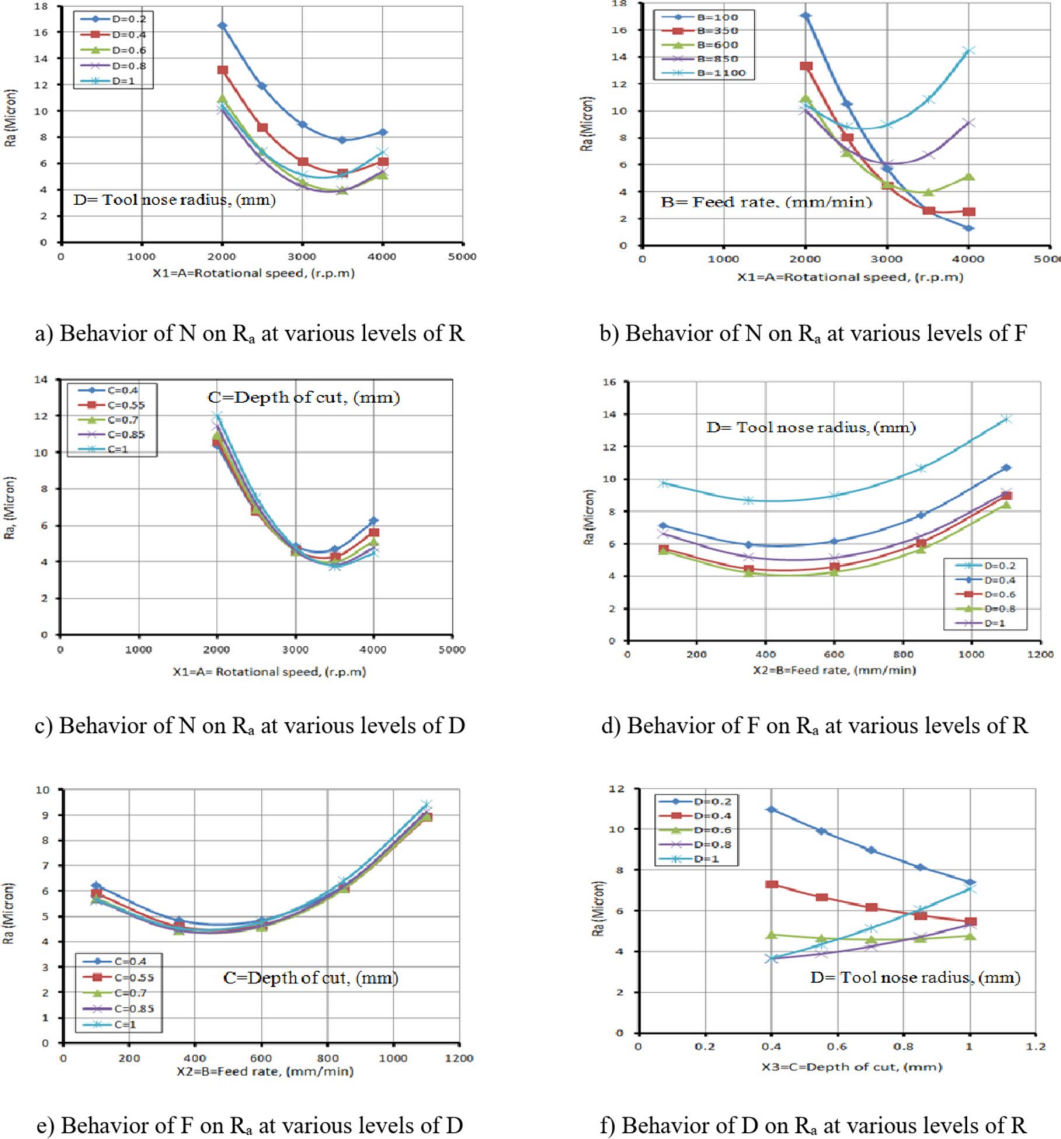
Influence of the various parameters on Ra.

Figures 33(a to f) illustrate the effect of I/P parameters on Ra, showing that as N increases, Ra decreases. This occurs because, as temperature rises and rake frictional stress decreases, cutting forces and the tendency for built-up edge formation reduce at higher cutting speeds. Conversely, as F increases, Ra also increases.

This is because the feed per revolution squared determines the height of the feed mark peaks and the depth of their valleys. Additionally, TWR increases with higher F values. It has also been observed that as D increases, Ra increases due to the rise in cutting forces. As a result, the peaks become wavier, increasing Ra.

Figures 33 (a to c) illustrate the relationship among N and Ra at varying values of R, F, and D. From Fig. 33(a), it is observed that as the N increases from 2000 r.p.m to 3500 r.p.m, the Ra value decreases with changes in R. This behavior can be attributed to enhanced cutting action and reduced built-up edge formation at moderate-to-high spindle speeds, which leads to better surface quality. However, when the spindle speed further increases from 3500 r.p.m to 4000 r.p.m, the Ra value begins to increase.

This is likely due to elevated cutting temperatures and increased tool vibration, both of which negatively impact surface finish. It is also clear from this figure that the variation in R significantly affects Ra, as a larger radius typically leads to a wider contact area and can influence chip formation and surface finish. This result is agreement with^44–46^.

Figure 33(b), which shows the relationship among N and Ra at varying F, demonstrates a similar trend to Figs. 33(a) and 33(c). This similarity stems from the dominant effect of N on cutting temperature and chip formation mechanisms across different F. Furthermore, it is evident that variations in F have a more substantial influence on Ra than R or D. This is due to the direct correlation between F and the height of feed marks higher feed rates leave deeper and more prominent surface irregularities. This result is agreement with^45,46^.

Figure 33(c) presents the relationship between N and Ra at different D. The behavior observed is consistent with that in Fig. 33(a), primarily because the N affects cutting forces and heat generation regardless of the D.

However, it is noted that changes in D have only a slight effect on Ra. This is likely because, within the studied range, the D does not significantly alter the chip formation or surface interaction as long as cutting remains stable. This result is agreement with^44–46^.

Additionally, Figs. 33(d) and 33(e) explore the relationship among F and Ra with variations in R and D, respectively. Among these, the influence of R is more prominent. In both figures, Ra decreases as the F elevates from 100 mm/min to 500 mm/min.

This counterintuitive result can be attributed to a reduction in contact time between tool and workpiece, which reduces frictional heating and tool wear. However, when the F elevates from 500 mm/min to 1100 mm/min, the Ra value rises sharply, exceeding 9 microns. This increase is due to greater chip load and more pronounced feed marks, which contribute to surface roughness. This result is agreement with^46–48^.

Finally, Fig. 33(f) illustrates the relationship between D and Ra at different R. When the R reached 0.2 mm, 0.4 mm, or 0.6 mm, the Ra value decreases with increasing D. This may result from improved chip evacuation and cutting stability under moderate depth engagement. In contrast, when the R is 0.8–1 mm, Ra increases as D increases. This is likely due to the larger contact area causing greater cutting forces and vibrations, which in turn deteriorate the surface finish. This result is agreement with^44–46^.

Table 8 presents the Ra response table. According to this table, N has the greatest effect on Ra, followed by R, F, and D.

The ANOVA data for Ra, shown in Table 8, indicates that N and R play a crucial role in Ra, as their p-values are less than α = 0.005 at a 95% confidence level, whereas D has a minimal influence on Ra since its p-value exceeds α.

The ANOVA results (Table 8) and graphical diagnostics (Fig. 8) confirm the adequacy of the proposed mathematical model for predicting Ra, with N and R identified as the most statistically significant factors. From a physical standpoint, increasing N reduces Ra at moderate levels due to improved shearing action and reduced built-up edge formation, whereas excessive speeds lead to higher Ra because of elevated temperatures and vibration. SRs also increases with F as a result of deeper feed marks, while D exhibits a relatively minor influence within the stable cutting regime. This integrated statistical–physical interpretation clarifies the mechanisms governing surface formation and supports the robustness of the Ra prediction model.

**Fig. 34:**
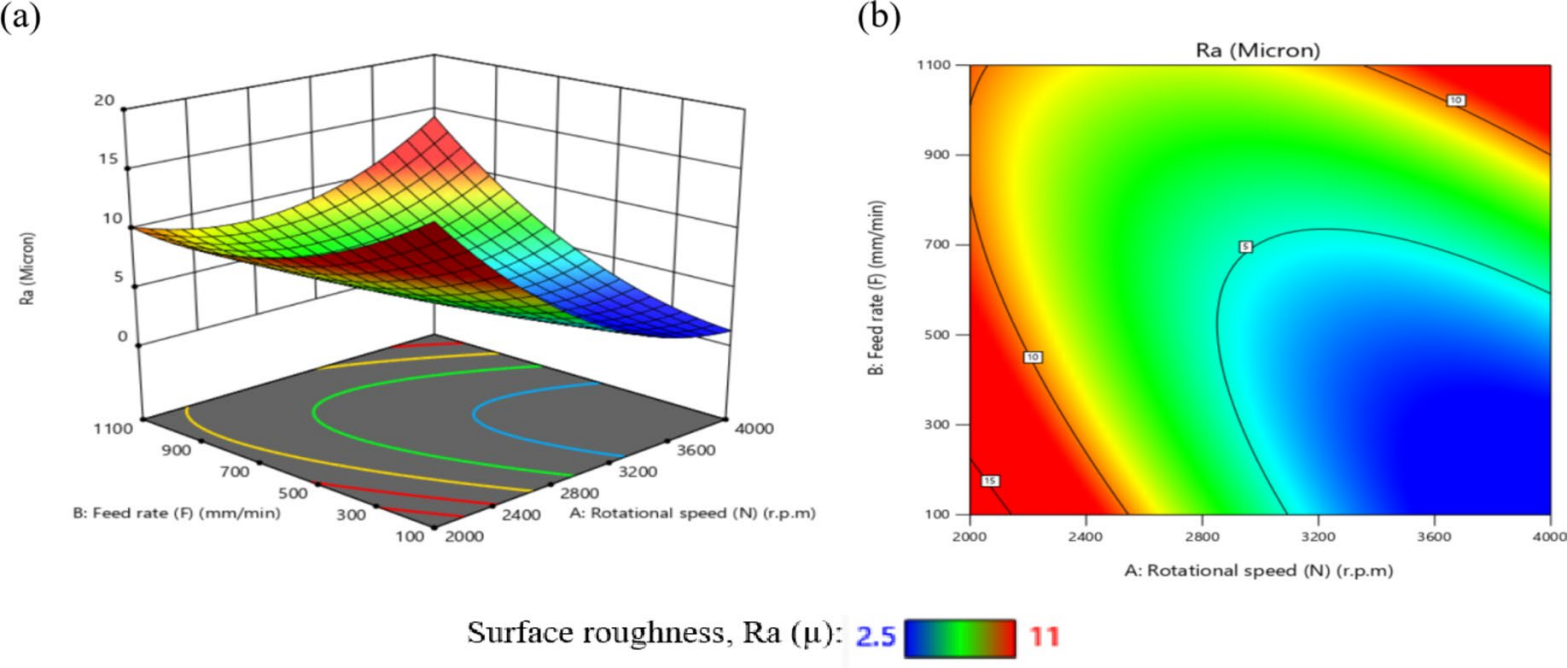
Interaction of N and F on R_a_: (**a**) 3D surface; (**b**) contour plot.

The influence of the interaction between N and F on Ra is presented in Fig. 34. As shown in Fig. 34(a), Ra exhibits reducing trends followed by a slight elevate as either N or F increases, under constant values of other machining parameters. The lowest Ra of 1.5 μm is accomplished at a low F of 100 mm/min and a high N of 4000 r.p.m. In contrast, the maximum Ra value reaches 17 μm under the same F (100 mm/min) but at a reduced N of 2000 r.p.m.

Figure 34(b) illustrates that the surface gradient is steeper along the N-axis than along the F-axis, signifying that N exerts a stronger influence on Ra than F. Interaction analysis recommends operating within a F range of 100–700 mm/min and N range of 3100–4000 r.p.m. to achieve Ra values below 5 μm. This result is agreement with^46–48^.

**Fig. 35:**
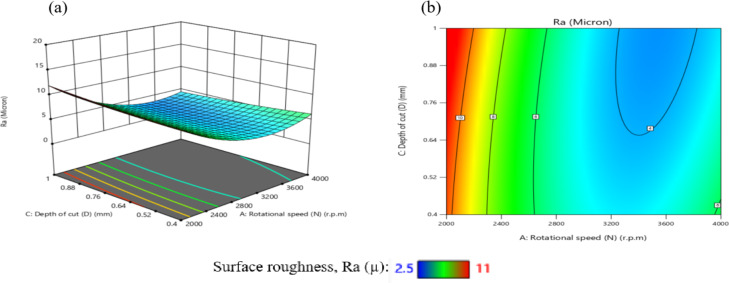
Interaction of N and D on R_a_: (**a**) 3D surface; (**b**) contour plot.

The combined effect of N and D on Ra is demonstrated in Fig. 35. The analysis in Fig. 35(b) shows that N has a more dominant effect on Ra than D, as indicated by the steeper gradient in the direction of N. The highest Ra, measured at 11 μm, occurs when the D is 1 mm and N is 2000 r.p.m. According to the interaction analysis, to maintain Ra below 5 μm, the D should be maintained between 0.64 and 1.00 mm, and N should range from 3200 to 3800 r.p.m. This result is agreement with^44–46^.

**Fig. 36:**
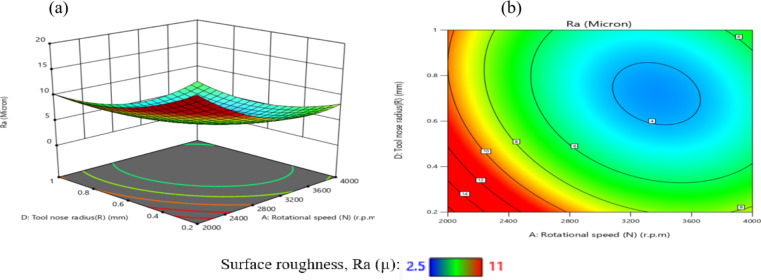
Interaction of N and R on R_a_: (**a**) 3D surface; (**b**) contour plot.

Figure 36 highlights the interaction between R on Ra. As illustrated in Fig. 36(a), Ra initially declines and then experiences a slight increase with rising N or R, assuming other parameters remain constant. The minimum Ra of 5 μm is recorded at *N* = 4000 r.p.m. and *R* = 0.6 mm, whereas the maximum value of 16 μm is observed at *N* = 2000 r.p.m. and *R* = 0.2 mm. The response surface in Fig. 36(b) shows a steeper slope along the N-axis than the R-axis, confirming the greater sensitivity of Ra to N.

Interaction analysis suggests optimal conditions for minimizing Ra below 4 μm are achieved by maintaining N between 3100 and 3700 r.p.m. and R between 0.6 and 0.85 mm. This result is agreement with^45–47^.

**Fig. 37:**
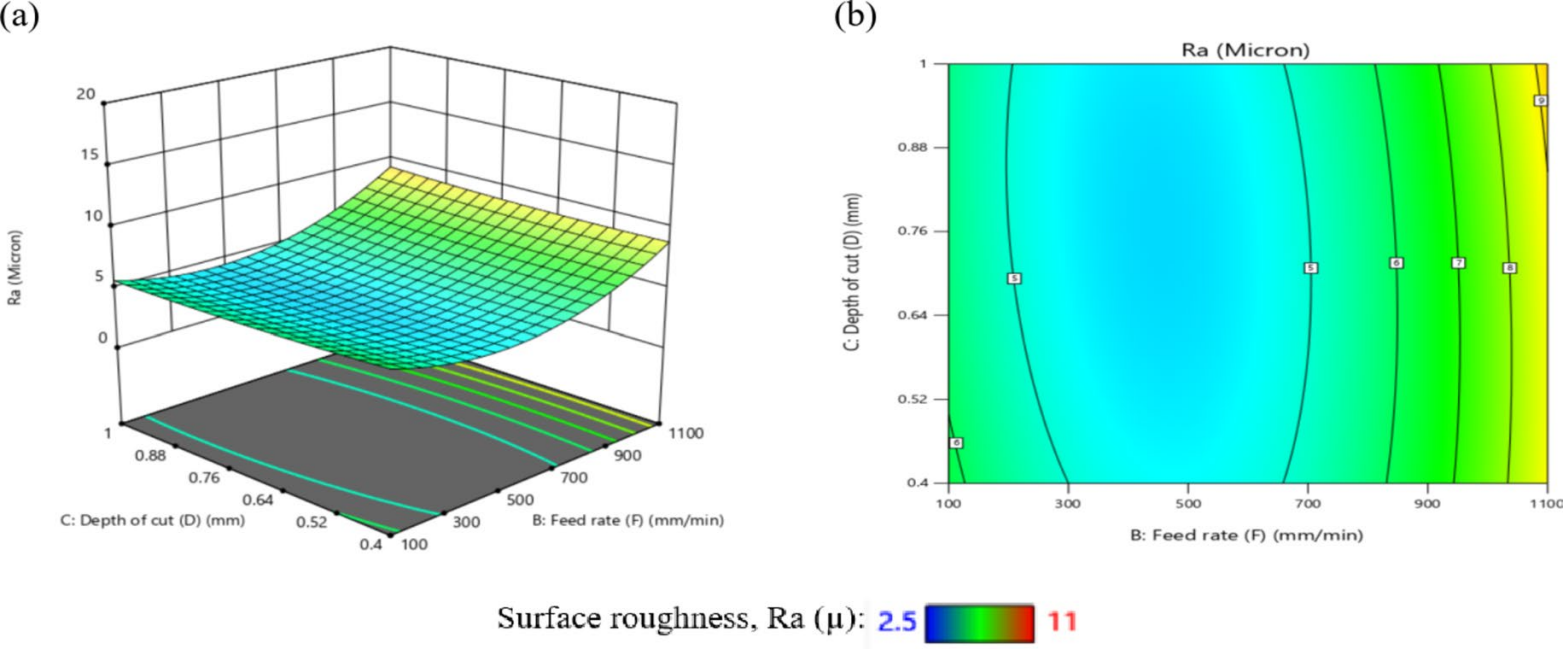
Interaction of F and D on R_a_: (**a**) 3D surface; (**b**) contour plot.

The influence of the F and D interaction on Ra is depicted in Fig. 37. Figure 37(a) reveals that Ra follows a U-shaped trend, decreasing initially and then increasing with higher values of F or D. The minimum Ra of 2.5 μm occurs when D is 0.64 mm and F is 500 mm/min, while the highest Ra of 9.5 μm is recorded at D = 1 mm and F = 1100 mm/min. This result is agreement with^45–48^.

As shown in Fig. 37(b), the gradient is steeper along the F-axis, indicating a stronger effect of feed rate on surface roughness compared to depth of cut. To maintain Ra below 5 μm, the recommended operational window lies within D = 0.4–1.0 mm and F = 300–650 mm/min. This result is agreement with^45–47^.

### Behavior of TP conditions on R_max_

Table 9 presents the response table for R_max_. The table indicates that N has the greatest effect on R_max_, then by D, F, and R. The ANOVA data for R_max_, shown in Table 8, reveals that N, D, and F have a strong influence on R_max_, as their p-values are less than α = 0.005 at a 96.7% confidence level, while R has a negligible effect since its p-value exceeds α.

**Fig. 38:**
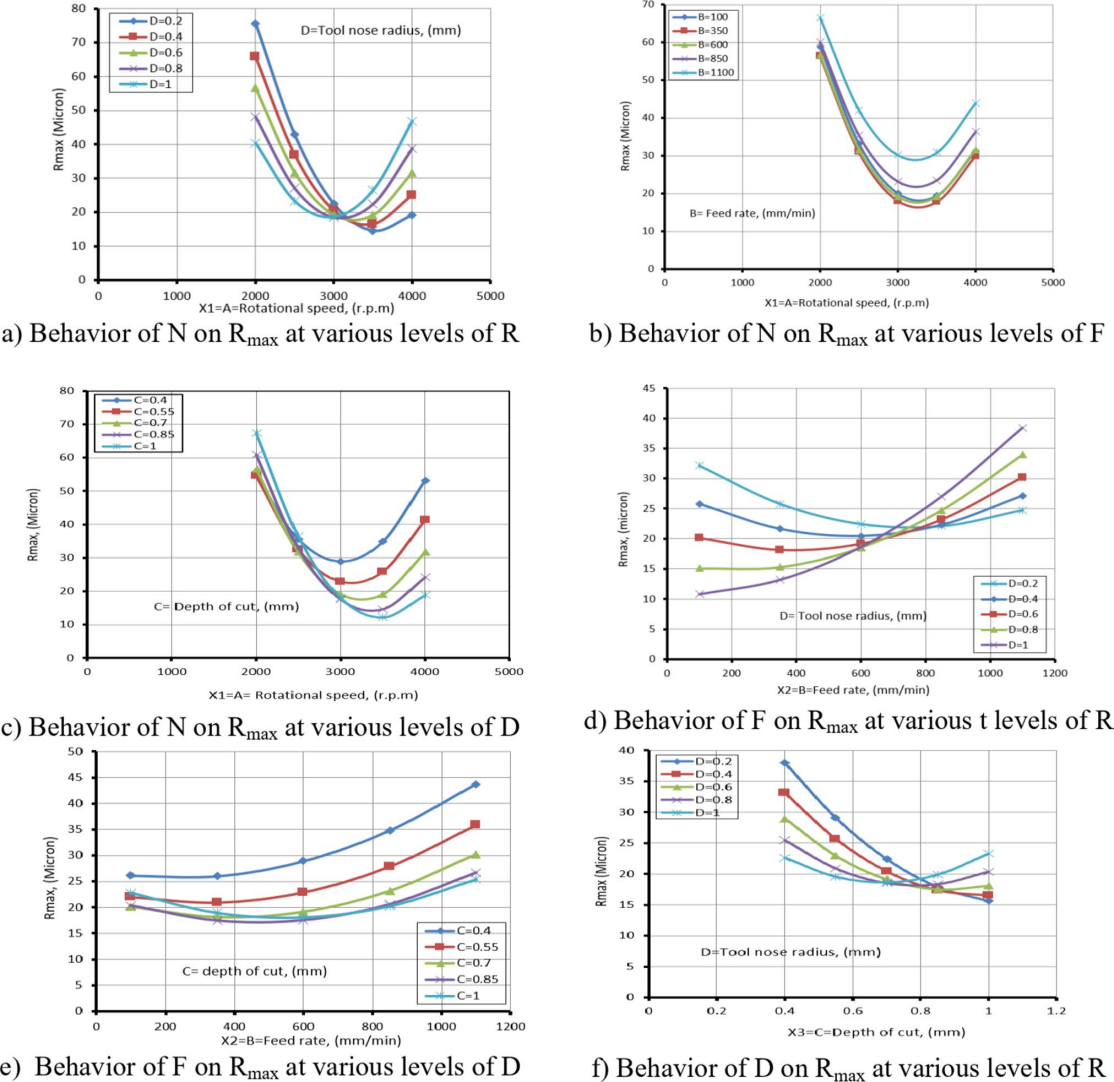
Influence of the various parameters on R_max_.

Figures 38(a–c) illustrate the effect of N on the R_max_ at various values of R, F, and D. In Fig. 38(a), it is observed that increasing the N from 2000 to 3000 rpm which is a significant reduction in R_max_ from 78 microns to 20 microns. This improvement in surface quality is attributed to enhanced cutting conditions at moderate spindle speeds, which reduce the likelihood of built-up edge formation and promote more stable tool–workpiece interaction. This result is agreement with^48,49^.

However, as the N further increases to 4000 rpm, R_max_ begins to rise again, reaching 50 microns. This can be explained by the increase in cutting temperatures and mechanical vibrations, which negatively affect surface integrity. A critical interaction point is observed at N of 3100 rpm, where the minimum R_max_ value of 18 microns is recorded. This suggests that this speed range provides optimal conditions, balancing cutting efficiency, thermal stability, and vibration control.

Figures 38(b) and 38(c), which depict the relationship among N and R_max_ at varying F and D, respectively, reveal a similar trend: R_max_ decreases from 68 microns to 18 microns as the N elevates from 2000 to 3100 rpm. This decline is linked to improved chip formation and thermal behavior under increasing speed. Among the two parameters, D exhibits a more pronounced effect on Rmax than F. This is due to the direct influence of cutting depth on cutting forces and chip removal, which in turn influences surface roughness. This result is agreement with^48,49^.

Figure 38(d) presents the influence of F on R_max_ at different R. As the F increases, R_max_ also increases. A notable interaction point is identified at F of 700 mm/min, where R_max_ is approximately 23 microns. The behavior varies with R, for larger R (0.8 mm and 1 mm), R_max_ increases both before and after the interaction point due to the larger contact area and associated thermal and vibrational effects.

In contrast, for smaller R (0.2 mm, 0.4 mm, and 0.6 mm), R_max_ initially decreases before the interaction point due to enhanced precision and reduced tool engagement. However, it subsequently increases, reaching a peak value of 38 microns after the interaction point, likely due to increased stress and tool wear. This result is agreement with^48,49^.

Figure 38(e) presents the relationship among F and R_max_ at varying D. It shows a consistent increase in R_max_ with increasing F, as higher feed rates result in more pronounced feed marks and surface irregularities. This result is agreement with^48,49^.

Conversely, Fig. 38(f) shows that R_max_ decreases as D increases, particularly when R varies. This behavior can be attributed to improved chip evacuation and cutting stability at higher depths of engagement. A critical interaction point is observed at a D of 0.8 mm and an R_max_ value of 20 microns, indicating an optimal condition where increased D contributes to better surface finish. This result is agreement with^48,49^.

The ANOVA results (Table 9) and diagnostic plots (Fig. 9) confirm the statistical significance and adequacy of the proposed R_max_ model, with N identified as the dominant factor, followed by D and F. Physically, increasing N reduces R_max_ at moderate speeds due to improved chip formation and reduced built-up edge, while excessive speeds increase R_max_ because of thermal effects and vibration. Feed rate increases R_max_ through deeper feed marks and surface irregularities, whereas depth of cut improves surface stability up to an optimal level by enhancing chip evacuation. This combined statistical physical interpretation explains the observed Rmax trends and validates the predictive capability of the model.

**Fig. 39:**
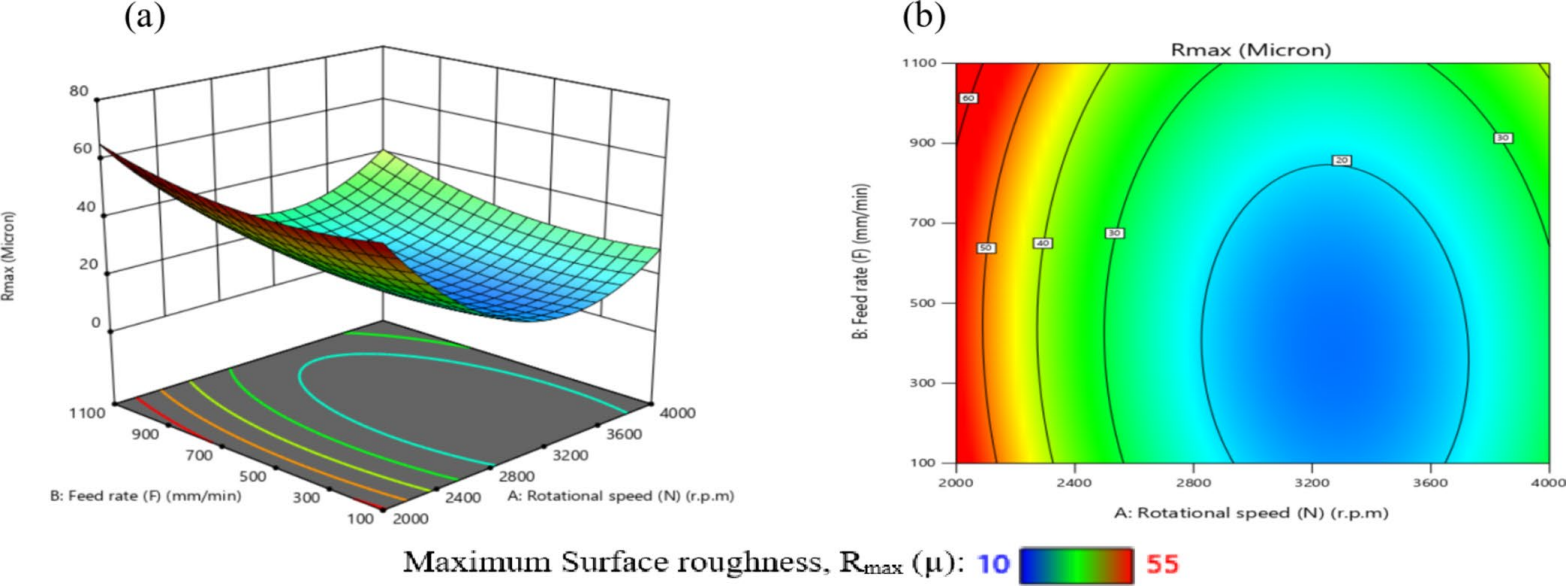
Interaction of N and F on R_max_: (a) 3D surface; (b) contour plot.

The influence of the interaction among N and F on the R_max_ is elucidated in Fig. 39. As presents in Fig. 39(a), R_max_ exhibits a nonlinear behavior initially decreasing and then slightly increasing with rising values of either N or F, under constant values of other parameters. The lowest recorded R_max_ is 10 μm, observed at F = 350 mm/min and *N* = 3200 r.p.m. Notably, the effect of N is more significant than that of F, as demonstrated in Fig. 39(b), where the steeper gradient in the N direction indicates greater sensitivity. In contrast, the highest R_max_ value of 55 μm is observed at F = 1100 mm/min and *N* = 2000 r.p.m. Based on interaction analysis, maintaining F in the range of 100–850 mm/min and N between 3000 and 3700 r.p.m. is recommended to achieve R_max_ values below 20 μm. This result is agreement with^48,49^.

The interaction among N and D and its effect on R_max_ is depicted in Fig. 39. Figure 39(a) reveals a similar trend: R_max_ decreases initially and then slightly increases as either N or D increases, while other conditions are kept constant. The minimum R_max_ of 10 μm is achieved at *N* = 4000 r.p.m. and D = 1 mm. As illustrated in Fig. 39(b), the more pronounced slope along the N axis confirms the dominant influence of N over D. The maximum R_max_, reaching 62 μm, occurs at *N* = 2000 r.p.m. and D = 1 mm. To ensure R_max_ values remain below 25 μm, optimal performance is attained when D is between 0.60 and 1 mm and N is within 2800–4000 r.p.m. This result is agreement with^48,49^.

**Fig. 40:**
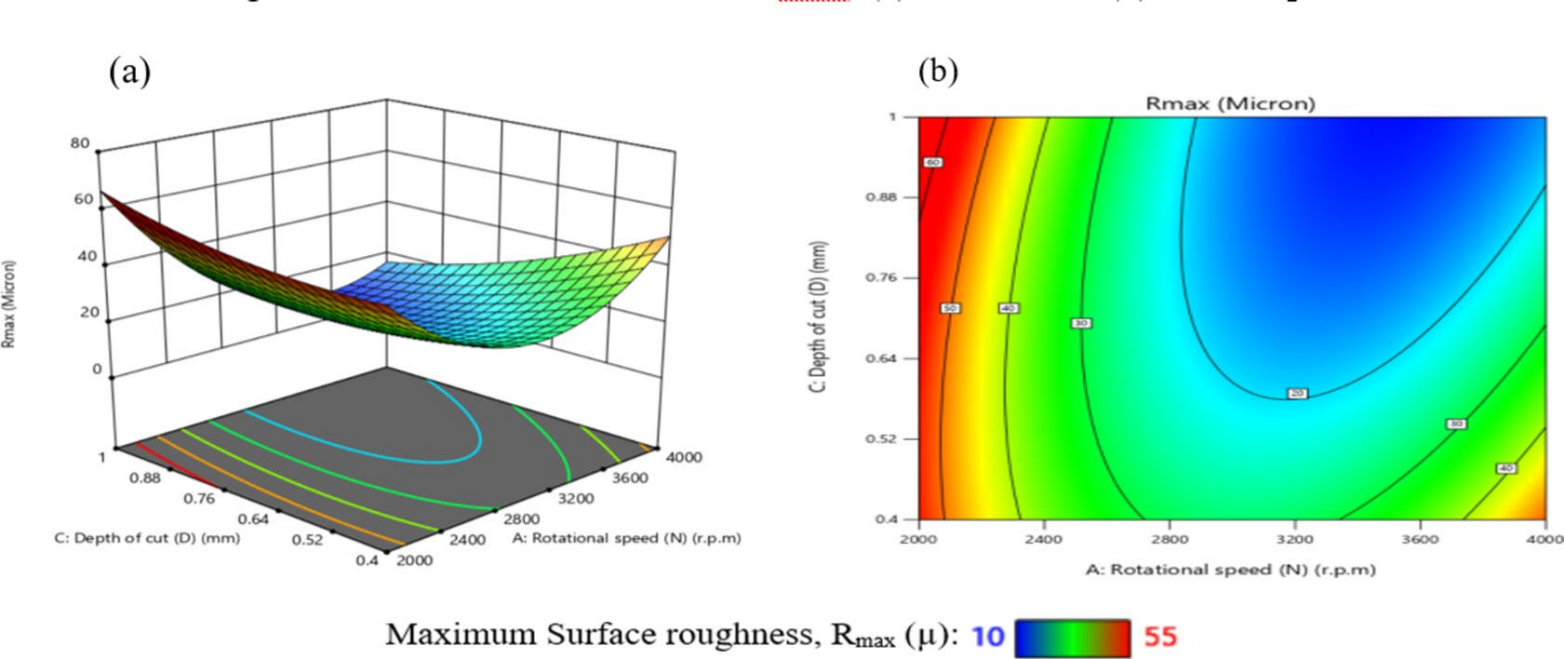
Interaction of N and D on R_max_: (a) 3D surface; (b) contour plot.

Figure 40 presents the effect of the interaction among N and R on R_max_. As observed in Fig. 40(a), the surface roughness initially reduced and then slightly elevates as N or R rises, assuming all other variables are fixed. The optimal condition of R_max_ = 10 μm is recorded at *N* = 4000 r.p.m. and *R* = 0.2 mm. Figure 40(b) again highlights the greater impact of N over R, evidenced by the steeper gradient along the N axis. The R_max_, measured at 78 μm, occurs when *N* = 2000 r.p.m. and *R* = 0.2 mm. To maintain R_max_ below 20 μm, the recommended parameter ranges are *N* = 3000–4000 r.p.m. and *R* = 0.2–1 mm. This result is agreement with^48,49^.

The effect of the N-F interaction on R_max_ is depicted in Fig. 39. Figure 39(a) illustrates that R_max_ initially decreases and then slightly increases as N or F increases when other parameters remain constant. The minimum R_max_ is 10 μm when F is 350 mm/min and N is 3200 r.p.m. The effect of N on R_max_ is greater than that of F, as presented in Fig. 39(b), where the gradient in the direction of N is steeper than in the direction of F. The maximum R_max_ is 55 μm when F is 1100 mm/min and N is 2000 r.p.m. This result is agreement with^48,49^.

Interaction analysis suggests that F should range from 100 mm/min to 850 mm/min, and N should range from 3000 r.p.m. to 3700 r.p.m. to achieve an R_max_ of less than 20 μm.

The N-D interaction effect on R_max_ is illustrated in Fig. 40.

Figure 40(a) shows that R_max_ first decreases and then slightly increases as N or D increases while other parameters remain constant. The minimum R_max_ is 10 μm when N is 4000 r.p.m. and D is 1 mm.

The effect of N on R_max_ is greater than that of D, as noted in Fig. 40(b), where the gradient in the direction of N is steeper than in the direction of D.

The maximum R_max_ is 62 μm when N is 2000 r.p.m. and D is 1 mm. Interaction analysis suggests that D should range from 0.60 mm to 1 mm, and N should range from 2800 r.p.m. to 4000 r.p.m. to achieve an R_max_ lower than 25 μm. This result is agreement with^48,49^.

**Fig. 41:**
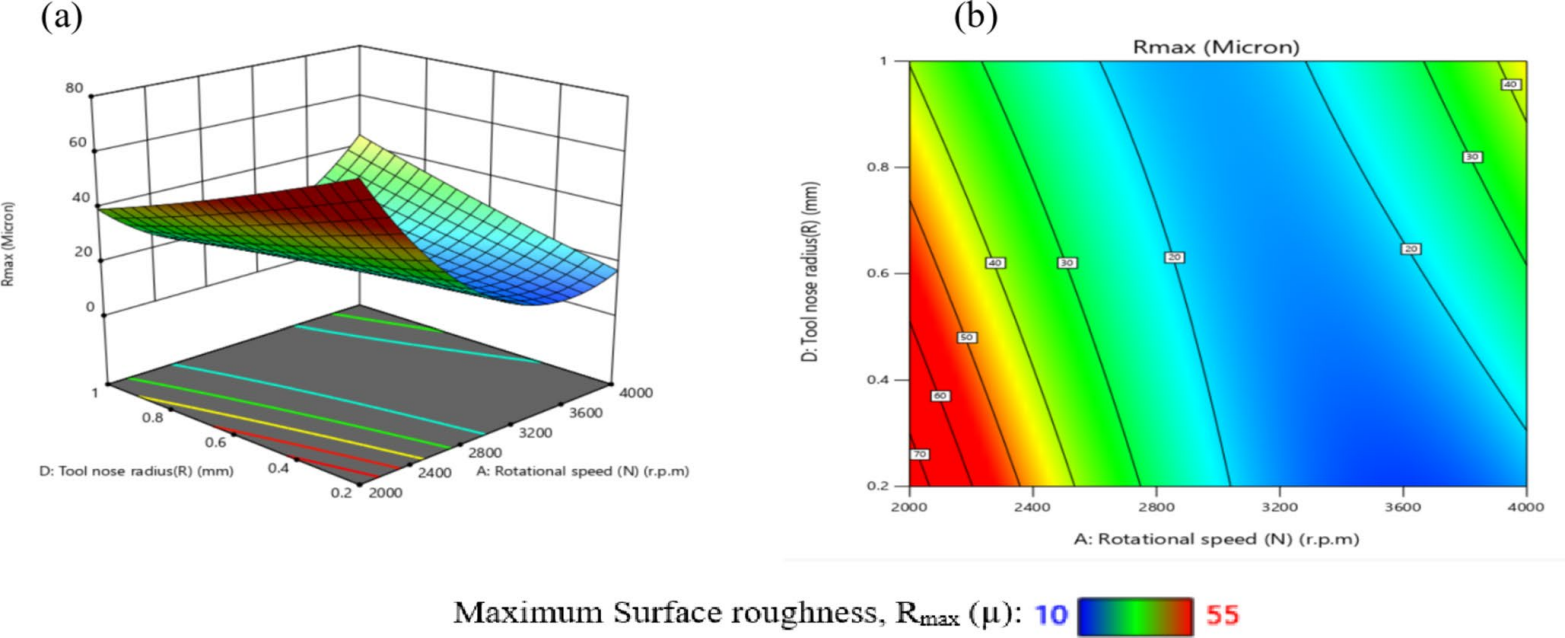
Interaction of N and R on R_max_: (a) 3D surface; (b) contour plot.

The effect of the N-R interaction on R_max_ is depicted in Fig. 41. Figure 41(a) illustrates that R_max_ initially decreases and then slightly increases as N or R increases when other parameters remain constant. The minimum R_max_ is 10 μm when N is 4000 r.p.m. and R is 0.2 mm.

The effect of N on R_max_ is greater than that of R, as shown in Fig. 41(b), where the gradient in the direction of N is steeper than in the direction of R.

**Fig. 42:**
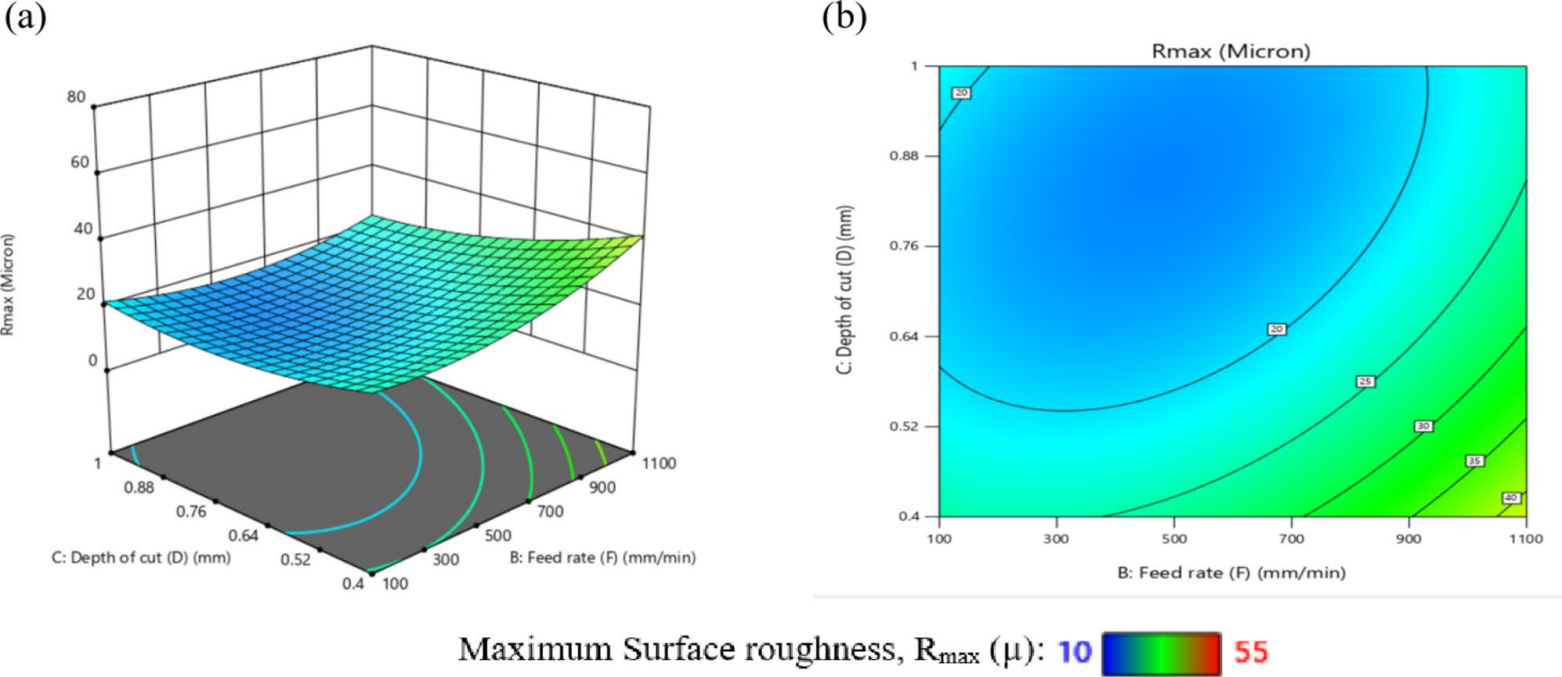
Interaction of F and D on R_max_: (a) 3D surface; (b) contour plot.

The maximum R_max_ is 78 μm when N is 2000 r.p.m. and R is 0.2 mm. Interaction analysis suggests that N should range from 3000 r.p.m. to 4000 r.p.m., and R should range from 0.2 mm to 1 mm to achieve an R_max_ lower than 20 μm. This result is agreement with^48,49^. The combined influence of F and D on R_max_ is presented in Fig. 42. Figure 42(a) indicates that R_max_ decreases initially and then slightly increases with increasing D, while a consistent increase in R_max_ is observed with higher F values. The minimum R_max_ of 10 μm is achieved at D = 0.7 mm and F = 100 mm/min. Figure 42(b) clearly illustrates that F has a stronger effect than D, as reflected by the steeper gradient along the F axis. The maximum R_max_ of 55 μm is associated with D = 0.4 mm and F = 1100 mm/min. To attain R_max_ values below 20 μm, feed rate should be maintained between 100 and 900 mm/min and depth of cut within 0.59–1 mm. This result is agreement with^48,49^.

The F-D interaction effect on R_max_ is shown in Fig. 42. As illustrated in Fig. 42(a), when other parameters remain constant, R_max_ initially decreases and then slightly increases as D increases. Additionally, R_max_ increases with higher F values.

The minimum R_max_ is 10 μm when D is 0.7 mm and F is 100 mm/min. The influence of F on R_max_ is greater than that of D, as seen in Fig. 39(b), where the gradient in the direction of F is greater than in the direction of D. This result is agreement with^48,49^.

The maximum R_max_ is 55 μm when D is 0.4 mm and F is 1100 mm/min. Interaction analysis suggests that F should range from 100 mm/min to 900 mm/min, and D should range from 0.59 mm to 1 mm to achieve an R_max_ lower than 20 μm.

### Behavior of turning process conditions on OR

Table 10 presents the OR response table. It can be inferred from the table that N has a greater impact on OR. The ANOVA data for OR, shown in Table 10, clearly indicates that N has a stronger effect on OR, as its p-value is less than α = 0.005 at a 96% confidence level, whereas D, F, and R have a negligible effect since their p-values are greater than α.

**Fig. 43:**
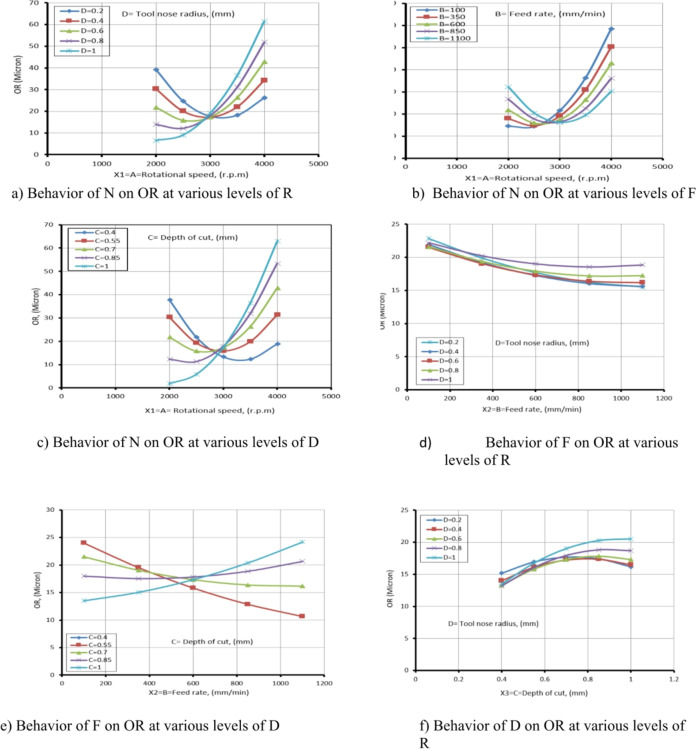
Influence of the various parameters on OR.

Figures 43(a to c) illustrate the relationship among N and OR at varying values of R, F, and D. All three figures demonstrate a consistent trend, where an interaction point occurs at N of approximately 2900 rpm and an OR value of 18 microns. When the N increases from 2000 rpm to 2900 rpm, the OR values decrease despite changes in R, F, and D. This reduction can be attributed to improve cutting stability, improved shear mechanics, and decreased built-up edge formation at moderate speeds. This result is agreement with^50,51^.

However, beyond 2900 rpm, as the N continues to increase up to 4000 rpm, OR values rise significantly, reaching at least 60 microns.

This increase is likely due to thermal softening of the workpiece material, increased vibration, and possible tool wear at higher cutting speeds, which adversely affect surface finish and OR.

Figure 43(d) presents the relationship among F and OR for different R values. An inverse relationship is observed: as the F increases, OR values decrease across all R conditions.

This is likely a result of higher F reduce tool-workpiece contact time, limiting frictional heat generation and smearing effects, thereby enhancing surface finish and OR. This result is agreement with^50–52^.

Figure 43(e) shows the relationship among F and OR at different D. It is evident that increasing the F results in lower OR values at 0.4 mm, 0.55 mm, and 0.7 mm D. This can be attributed to efficient chip evacuation and reduced tool-material interaction time. However, the opposite trend is observed at D of 0.85 mm and 1.0 mm, where OR increases with F. This reversal is likely due to increased cutting forces and tool deflection at deeper cuts, which compromise surface integrity under higher feed conditions. This result is agreement with^50,51^.

Lastly, Fig. 43(f) depicts the relationship among D and OR at varying R values. As the D increases, OR also increases across all R. This can be attributed to higher material removal forces and greater mechanical stress on the cutting edge, which deteriorate the surface finish and OR.

ANOVA results revealed that N is the most statistically significant factor affecting OR, which is consistent with the physical behavior of the cutting process. Moderate cutting speeds enhance machining stability and reduce built-up edge formation and vibrations, leading to lower OR values. Conversely, excessive spindle speeds increase cutting temperature, vibration, and tool wear, which negatively affect SR. The influence of F and D becomes more pronounced at higher levels due to increased cutting forces and tool deflection. This strong agreement between statistical findings and physical interpretation confirms the reliability of the proposed model in describing OR error mechanisms.

**Fig. 44:**
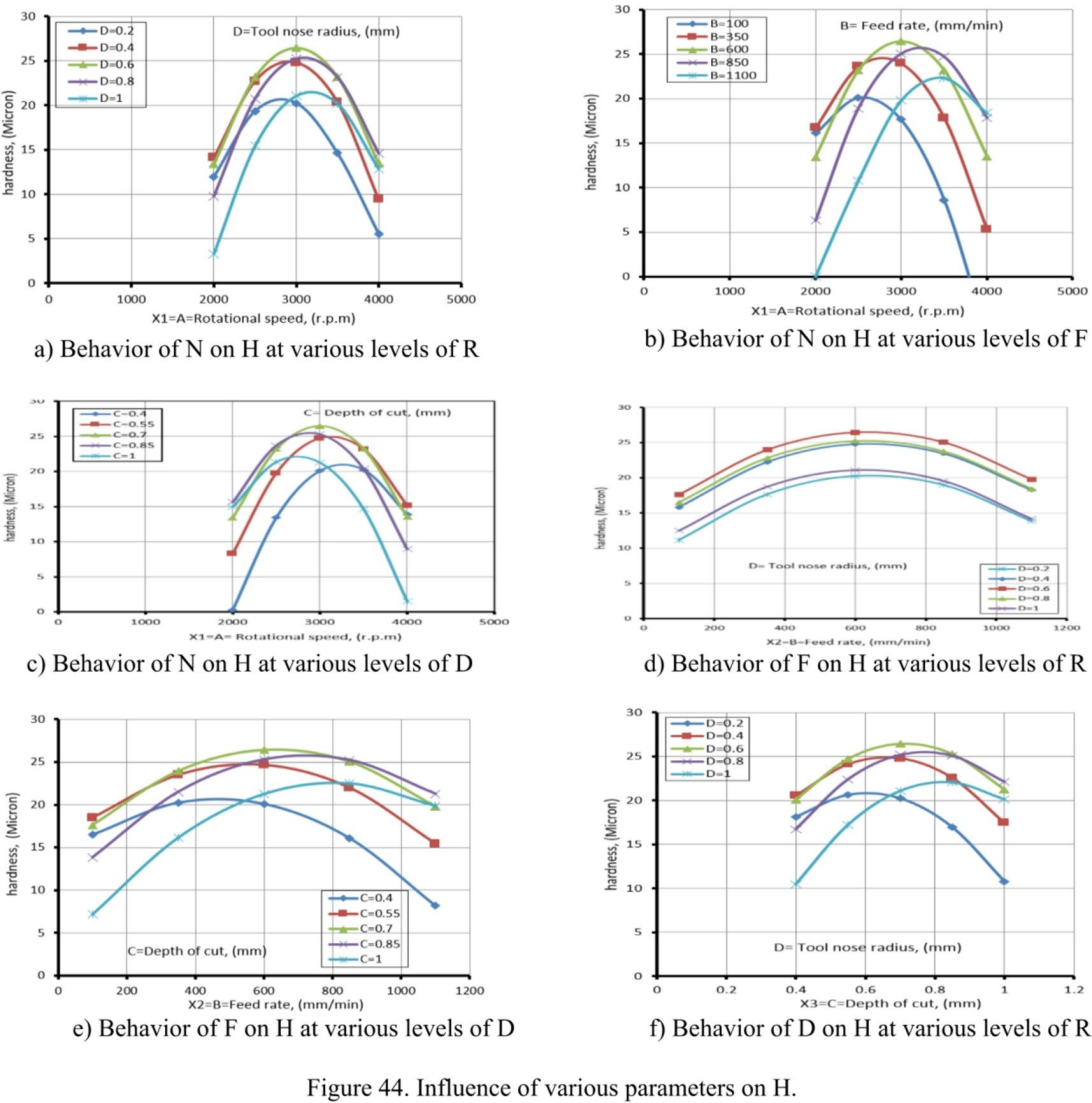
Influence of various parameters on H.

**Fig. 45:**
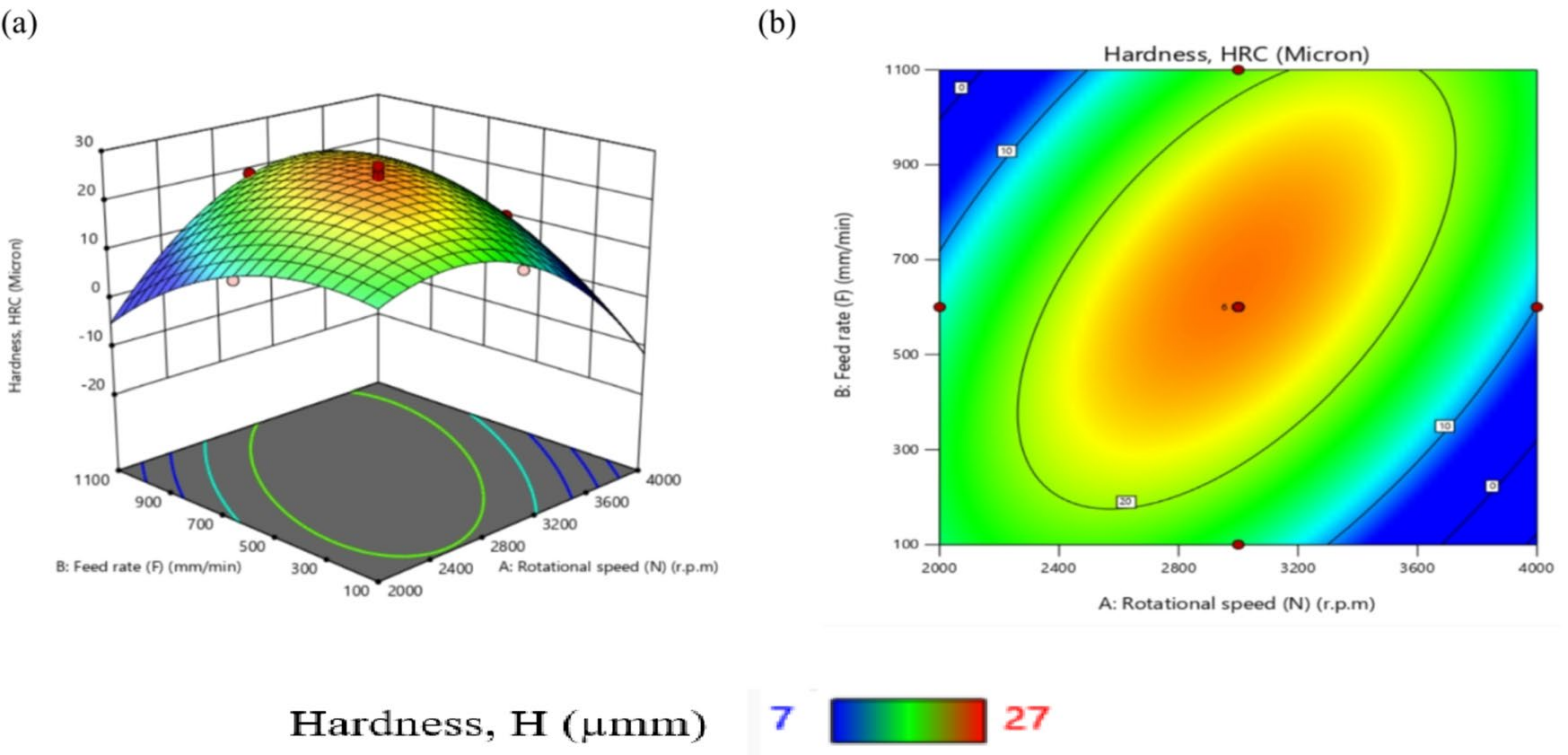
Interaction of N and F on H: (**a**) 3D surface; (**b**) contour plot.

**Fig. 46:**
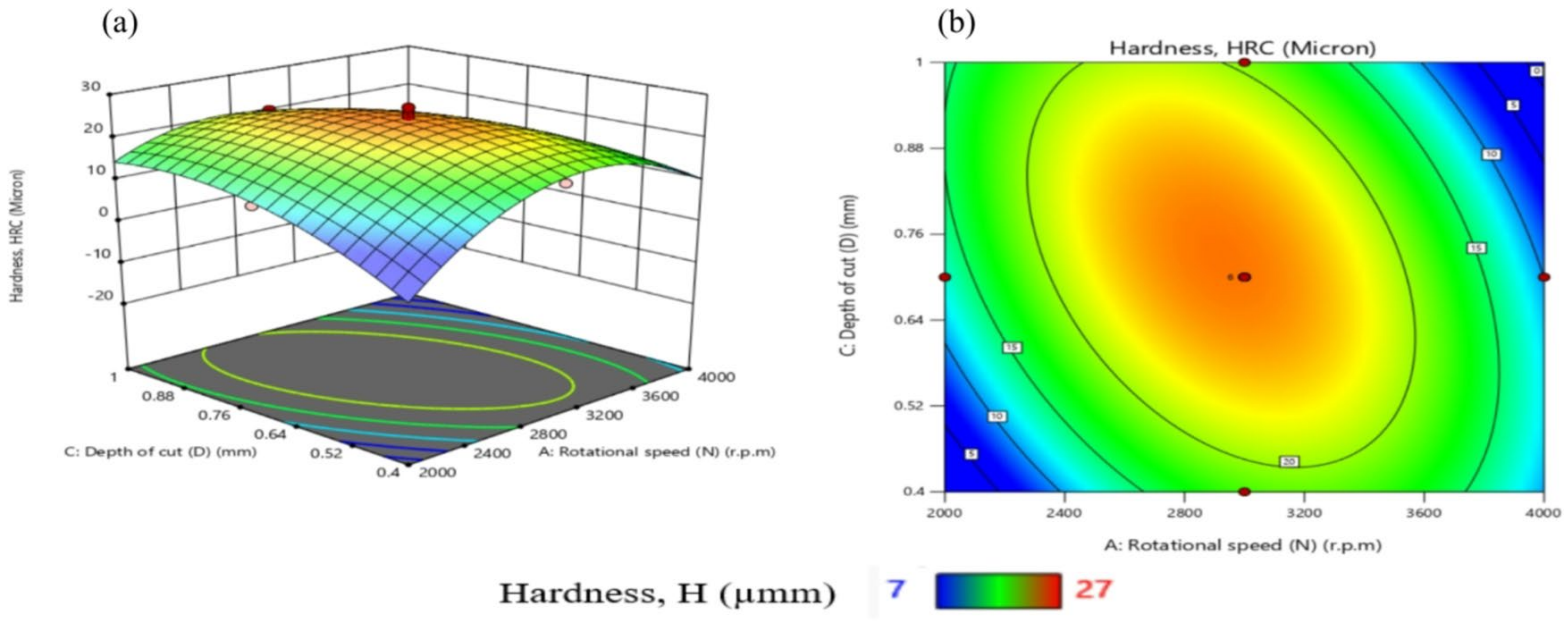
Interaction of N and D on H: (**a**) 3D surface; (**b**) contour plot.

**Fig. 47:**
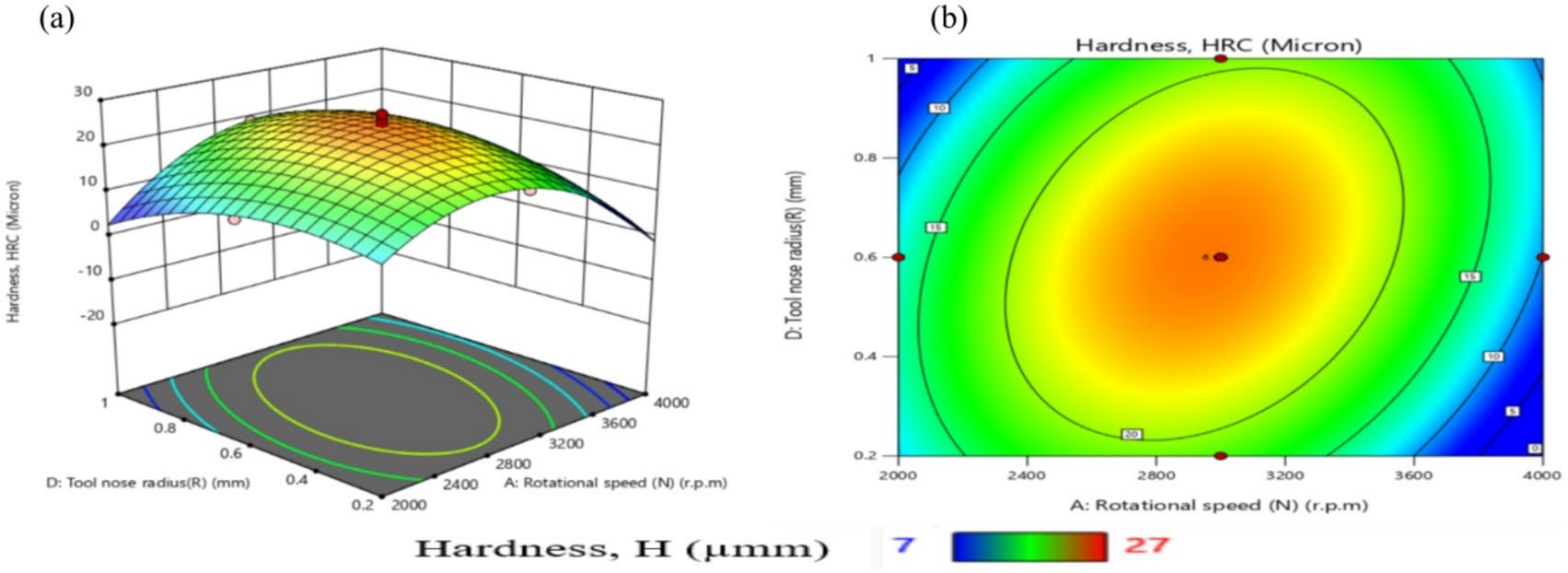
Interaction of N and R on H: (**a**) 3D surface; (**b**) contour plot.

The effect of various parameter interactions on OR is illustrated in Figs. 44, 45, 46 and 47. The N–F interaction (Fig. 44) shows that OR increases significantly as both N and F decrease, with the minimum OR of 6 μm observed at F = 100 mm/min and *N* = 2000 r.p.m. A steeper gradient in the N direction (Fig. 44b) highlights the stronger influence of N over F.

Conversely, the maximum OR of 59 μm occurs at F = 100 mm/min and *N* = 4000 r.p.m. To achieve OR values below 20 μm, the optimal ranges are F = 100–1100 mm/min and *N* = 2000–3000 r.p.m. Regarding the N–D interaction (Fig. 45), OR initially decreases and then slightly increases with rising N at D = 0.4 mm, while at higher D values (0.55–1 mm), OR consistently increases with N. This result is agreement with^50–52^.

The lowest OR of 6 μm is found at D = 1 mm and *N* = 2000 r.p.m., whereas the highest value (63 μm) occurs at D = 1 mm and *N* = 4000 r.p.m. Again, the gradient in the N direction confirms its dominant effect over D, and the optimal ranges for OR < 10 μm are D = 0.88–1 mm and *N* = 2000–2800 r.p.m. For the N–R interaction (Fig. 46), when *R* = 0.2 mm, increasing N reduces OR, but at larger nose radii (*R* = 0.4–1 mm), OR increases with N. The minimum OR of 6 μm is recorded at *R* = 1 mm and *N* = 2000 r.p.m., while the maximum (62 μm) is observed at the same R but at *N* = 4000 r.p.m., with N again showing a stronger influence than R.

To maintain OR < 10 μm, R should range from 0.9 to 1 mm and N from 2000 to 2600 r.p.m. Finally, the F–D interaction (Fig. 47) reveals that OR decreases with increasing F at D = 0.55 and 0.7 mm, but increases with F at D = 0.85 and 1 mm. The minimum OR (6 μm) occurs at D = 1 mm and F = 100 mm/min, whereas the maximum OR (45 μm) is observed at D = 1 mm and F = 1100 mm/min. The influence of F is greater than that of D, and to achieve OR below 10 μm, F should be set between 1000 and 1100 mm/min and D between 0.4 and 0.45 mm. This result is agreement with^50,51^.

### Behavior of turning process conditions on H

Figures 44(a to c) illustrate the impact of input parameters on hardness (H). It is evident that as N increases, so does H. This is because, at higher cutting speeds, the temperature rises, reducing the rake’s frictional stress, which in turn lowers cutting forces and decreases the tendency for built-up edge formation. Similarly, as F increases, H also increases. This is due to the fact that the feed per revolution squared determines the height of the feed marks peaks and the depth of their valleys.

Additionally, H increases with higher F values. It has been observed that increasing D also raises H, as cutting forces increase with D, leading to greater surface waviness and, consequently, higher H. Table 11 presents the response table for H. According to this table, F has the greatest impact on hardness, followed by N, D, and R. The ANOVA data for hardness, shown in Table 11, indicates that F and N have a significant effect on hardness, as their p-values are less than α = 0.005 at a 95.2% confidence level, whereas R has a negligible effect because its p-value exceeds α.

Figures 44(a to c) present the relationship between N and H at various R, F, and D. It is observed that as the N increases from 2000 rpm to 3000 rpm, the H improves significantly, rising from approximately 1 micron to 25 microns.

This trend is attributed to enhanced work hardening mechanisms occurring at moderate cutting speeds, where optimal thermal and mechanical loading promotes localized plastic deformation and surface densification. This result is agreement with^53–55^.

However, when the N further increases from 3000 rpm to 4000 rpm, the H values begin to decline, reaching below 5 microns across all tool configurations.

This reversal in trend is primarily due to excessive heat generation, which leads to thermal softening of the machined surface, microstructural alterations, and potential loss of surface integrity. This result is agreement with^55,56^.

Figure 44(d) illustrates the relationship between F and H at different R. When the F increases from 100 mm/min to 600 mm/min, the H increases from 10 microns to a maximum of 25 microns. This behavior can be explained by higher strain rates and more intensive mechanical interaction at the tool–workpiece interface, which induces strain hardening and improves the near-surface mechanical properties. This result is agreement with^53^.

Nevertheless, increasing the F further from 600 mm/min to 1100 mm/min leads to a decrease in H, with values dropping to a minimum of 14 microns. This reduction can be attributed to decreased contact time, reduced effective cutting engagement, and dynamic instabilities during high-speed feed conditions, which limit energy transfer and hinder work hardening. This result is agreement with^54^.

Figure 44(e) shows the effect of F on H at different D. A steady increase in F from 100 mm/min to 600 mm/min results in an increase in H, reaching up to 25 microns, particularly at D equal to 0.4 mm, 0.55 mm, and 0.7 mm.

This is due to optimal cutting forces that enhance material compaction and plastic deformation without inducing thermal softening. A similar effect is noted at greater depths (0.85 mm and 1 mm), with H increasing as the F reaches 700 mm/min.

This suggests that up to a certain feed threshold, material deformation outweighs softening effects, resulting in improved H even at higher material removal volumes. This result is agreement with^54–56^.

Finally, Fig. 44(f) depicts the relationship among D and H at various R. It is evident that as the D increases from 0.4 mm to 0.6 mm, H increases to a peak value of 25 microns, especially for R of 0.2 mm and 0.4 mm. This is likely due to more intense compressive stresses and effective plastic deformation at moderate depths.

However, a further increase in D from 0.6 mm to 1 mm leads to a reduction in H, reaching values as low as 11 microns. A similar trend is observed across other curves for larger R (0.6 mm, 0.8 mm, and 1 mm), particularly around D of 0.9 mm. This decline can be explained by increased cutting temperature, reduction in tool stability, and the onset of subsurface damage, all of which contribute to diminished H. This result is agreement with^56^.

The ANOVA results revealed that F and N are the most statistically significant factors affecting H, which is consistent with the underlying physical cutting mechanisms. Moderate values of N and F promote strain hardening due to optimal thermo-mechanical loading, leading to increased surface hardness. In contrast, excessive increases in N or F generate excessive heat and dynamic instability, resulting in thermal softening and a reduction in hardness. D exhibits a nonlinear influence, with maximum hardness occurring at intermediate values due to a balance between cutting forces and plastic deformation. This strong agreement between statistical findings and physical interpretation confirms the reliability of the proposed model for predicting surface hardness.

**Fig. 48:**
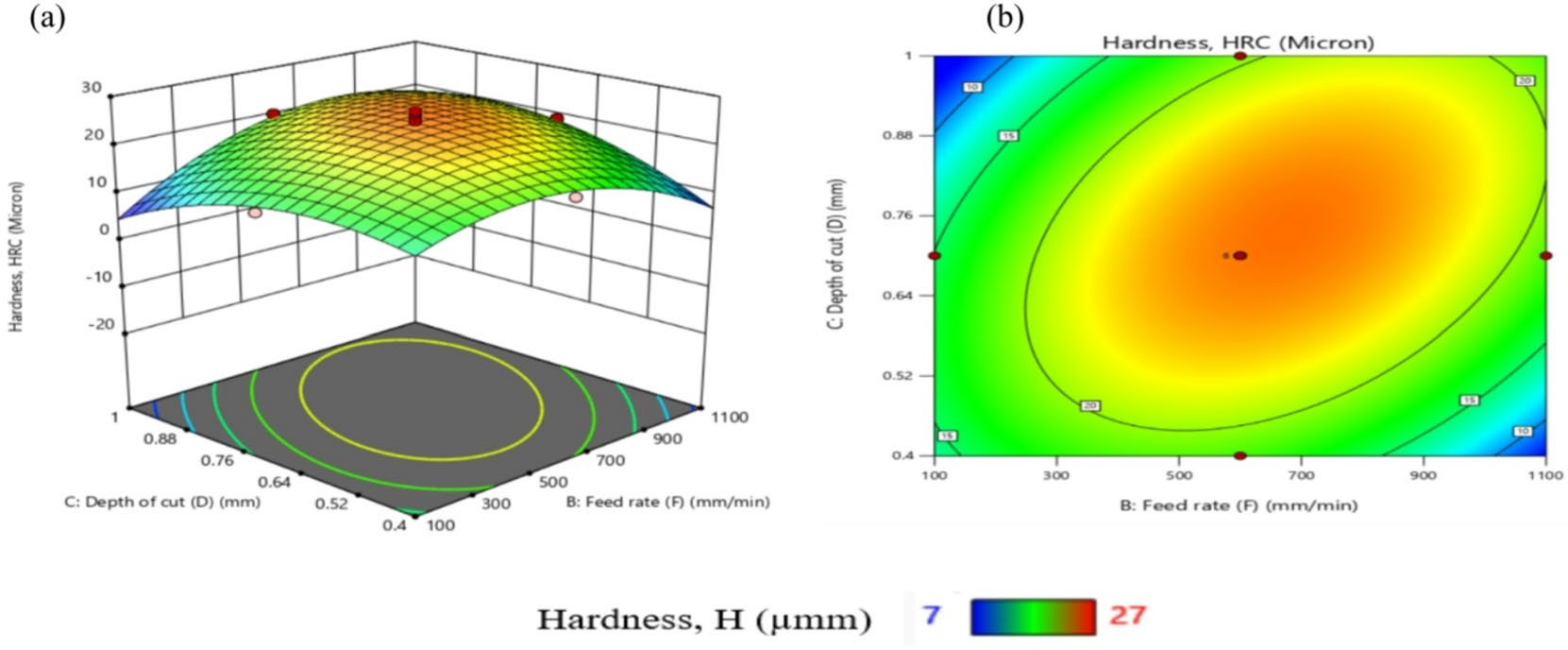
Interaction of F and D on H: (**a**) 3D surface; (**b**) contour plot.

Figures)45 to 48 (present 3D surface plots illustrating the interactive effects of different parameters on H. As shown in Figs. 45(a and b), 46(a and b), 47(a and b) and 48(a and b) the maximum H was reached at F = 600 mm/min with *N* = 3000 r.p.m., or with D = 0.7 mm and *R* = 0.6 mm. However, as F, N, D, and R increased, H increased. This result is agreement with^53^.

The effect of various cutting conditions on the intended responses can be analyzed using ANOVA, as shown in Table 11. A 95.2% confidence level was used to evaluate the selected experimental design. Table 11 presents the ANOVA results for each surface hardness variable examined in this study H, including the P-values, which indicate each parameter’s significance, and the percentage contribution of each factor.

Parameters with a significance level below 0.05 (5%) have a substantial effect on the quality attributes. With a contribution of 2.56%, F is the most significant factor influencing H. The next most influential parameters are N and D, contributing 1.7% and 0.29%, respectively. R has the least effect on H, with a contribution of 0.15%.

## Process multi-response optimization

Six different criteria were considered in this investigation: MRR, TWR, Ra, R_max_, OR, and H. The primary objective is to identify the optimal combination of condition levels that increases MRR and H while decreasing TWR, Ra, R_max_, and OR. However, due to potential conflicts among these criteria and their dependence on TP conditions and user preferences, achieving a multi-objective optimal decision that satisfies all competing goals can be challenging.

The multiple response method, also known as desirability, is a modern technique frequently used to address industrial challenges involving multiple quality attributes^31–35^.

A dimensionless value (d_i_), called desirability, is derived from each evaluated response using the optimization function, represented as D(X).

This method includes a goal function with a D(X) range from 0 to 1. A value of 0 indicates that one or more responses are outside the acceptable limits, while a value close to 1 represents the best-case scenario.

Composite Desirability (CD), which is the weighted geometric mean of individual desirability values, is used to assess the overall performance of the responses. The factor settings that yield the highest total desirability are considered the optimal parameter values.

Optimization is achieved using the simultaneous goal function, which calculates the geometric mean of all transformed responses. Individual optimizations are combined to obtain a CD^35^. The primary objectives are to maximize the CD and determine the optimal input variable settings. The evaluation of individual optimization depends on whether, given the study’s requirements, it is preferable to maximize a specific response.

14 $${d}_{i}=\left\{\begin{array}{cc}0&i<{L}_{i}\\\frac{i-{L}_{i}}{{T}_{i}-{L}_{i}}\times{r}_{i}&{L}_{i}<i<{T}_{i}\\1&i>{T}_{i}\end{array}\right\}$$

When minimizing a certain response is the goal, the individual optimization is evaluated as follows:

15 $${d}_{i}=\left\{\begin{array}{cc}0&i>{H}_{i}\\\frac{{H}_{i}-i}{{H}_{i}-{T}_{i}}\times{r}_{i}&{T}_{i}<i<{H}_{i}\\1&i<{T}_{i}\end{array}\right\}$$

When the goal is to target a certain response, the individual optimization is ascertained as follows:

16 $${d}_{i}=\left\{\begin{array}{cc}\frac{i-{L}_{i}}{{T}_{i}-{L}_{i}}\times{r}_{i}&{L}_{i}<i<{T}_{i}\\\frac{{H}_{i}-i}{{H}_{i}-{T}_{i}}\times{r}_{i}&{T}_{i}<i<{H}_{i}\\0&{H}_{i}<i<{L}_{i}\end{array}\right\}$$

Where:

- i represents the expected value of the i^th^ response.
- Ti denotes the desired outcome for the i^th^ response.
- L_i_ represents the least suitable value for the i^th^ response.
- Hi represents the most suitable value for the i^th^ response.
- d_i_ represents the desirability (D(X)) for the i^th^ response.
- CD represents the overall optimization.
- r_i_ represents the weight of the optimization function for the i^th^ response.
- w_i_ denotes the significance of the i^th^ response.
- w represents the total of all w_i_ values.
- The CD is evaluated as follows when each response is given the same weight:
  17 $$\mathrm{C}\mathrm{D}=(\mathrm{d}\mathrm{1}\mathrm{d}\mathrm{2}\dots\mathrm{d}\mathrm{n})1/\mathrm{n}=\left[\right(\mathrm{d}\mathrm{i}\mathrm{w}\mathrm{i}\left)\right]1/\mathrm{W}$$

The weight w_i_, which satisfies the criterion 0 < w_i_ < 1, and the sum of all weights w₁ + w₂ + w₃ + … + wₙ = 1, are considered to evaluate the potential differences in the importance of various responses, where n represents the number of responses. Table 14 presents the goals, constraints on the input conditions, weights for each condition, and their relative significance. Table 14 displays the results of ten distinct combinations of operating conditions, yielding high CD values ranging from 0.1504 to 0.8322. Additionally, the anticipated results for certain pairings are shown. The optimal values of the operational variables, as determined in Table 15, are presented in Table 16, along with the corresponding minimum and maximum constraints.

**Table 14:** Input TP conditions and responses limitations.

Name	Objective	Li	Hi	Lower W	Upper W	Importance
N, (r.p.m)	Is in range	2000	4000	1	1	1
F, (mm/min)	Is in range	100	1100	1	1	1
D, (mm)	Is in range	0.4	1	1	1	1
R, (mm)	Is in range	0.2	1	1	1	1
MRR, (gram/min)	Max	0.150728	0.4455	1	1	1
TWR, (gram/min)	Min	9E-06	0.0001389	1	1	1
Ra, (µm)	Min	2.5	11	1	1	1
R_max_, (µm)	Min	10	55	1	1	1
OR, (µm)	Min	6	45	1	1	1
H, (µm)	Max	7	27	1	1	1

**Table 15:** High desirability for TP combinations in machining CK45.

Number	Process parameters				Forecast responses						Desirability
	(*N*), (*r*.*p*.m)	(F), (mm/min)	(D), (mm)	(*R*), (mm)	MRR (g/min)	TWR (g/min)	Ra, (µm)	*R*_max,_ (µm)	OR, (µm)	H, (µm)	
1	3000	600	0.7	0.6	0.41	0	4.6	17.8	17.3	24.6	0.83
2	2265.5	1011.4	0.72	0.41	0.375	0.0001	10.7	51.2	28.4	8.4	0.17
3	2301.87	1100	0.87	0.56	0.325	0.0001	9.91	49.9	22.35	9.1	0.23
4	3639.8	407.7	0.90	0.62	0.35	0.0001	2.35	12.99	41.8	7.5	0.30
5	2262.8	987.16	0.68	0.40	0.371	0.0001	10.78	51.5	29.4	8.21	0.1504
6	3203.17	1100	1	0.39	0.3163	0.0001	10.52	11.87	25.41	14.34	0.35
7	3408.28	322.85	0.65	0.20	0.3461	0.0001	6.93	19.84	21.8	9.1	0.45
8	3477.79	1100	0.48	1	0.259	0.0001	10.33	53.2	13.2	8.9	0.197
9	3062.7	643.71	0.62	1	0.38	0	4.73	19.3	18.97	17.84	0.7234
10	3000	600	0.7	0.60	0.41	0	4.55	17.83	17.33	24.66	0.8322

**Table 16:** Constraints on parameters and optimal parameters for specimens machined using TP for CK45.

Parameter	limitations of parameters	Optimum parameters
N, (r.p.m)	2000–4000	3000
F, (mm/min)	100–1100	600
D, (mm)	0.4–1	0.70
R, (mm)	0.2–1	0.60

Using the derived equations (Eq. 8) and the CD optimization method, this study simultaneously optimized six responses: MRR, TWR, Ra, R_max_, OR, and H. Answer optimization requires that the achieved solution meets the overall objectives for each response. The optimal TP values for CK45 steel, along with their constraints, are presented in Table 17.

Table 16 displays the parameter terms, responses, targets, and weights for each condition for workpieces machined using rough TP. To determine the optimal solution conditions, the input thermal conditions were analyzed within a trial range to maximize MRR and H, while minimizing TWR, Ra, R_max_, and OR. In addition to the numerical values for the ideal temperature conditions, the tables also present the expected optimal response values under these TP settings.

**Fig. 49:**
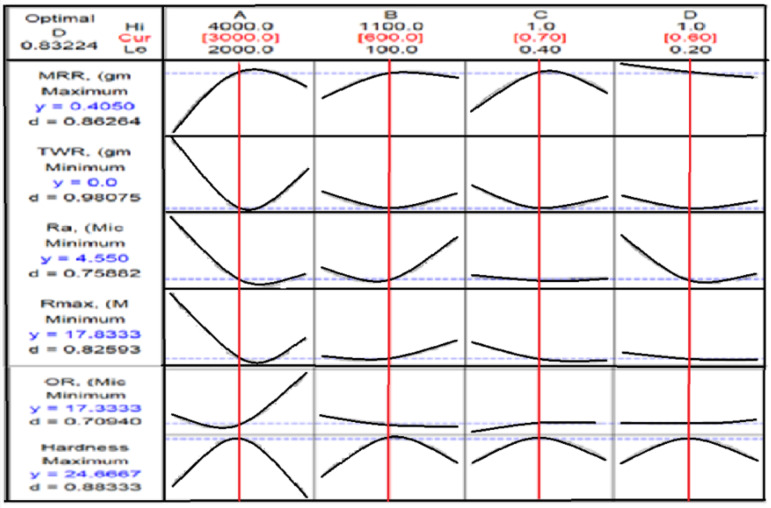
Multi-objective optimization outcomes for turning CK45.

Figure 49 illustrates the optimized curves for the six responses (MRR, TWR, Ra, R_max_, OR, and H) as well as the optimization results for workpieces machined using rough TP.

The response magnitudes are represented by the horizontal dotted lines within the cells, while the ideal thermal parametric settings are indicated by the remaining vertical lines inside the cells. The CD values for CK45 steel workpieces machined using TP were found to be 0.83224.

**Table 17:** Expected optimal responses for workpieces machined with turning parameters for CK45.

Response	Objective	Expected optimum responses
MRR, (gram/min)	Maximize	0.40501
TWR, (gram/min)	Minimize	1.15E-05
Ra, (µm)	Minimize	4.55
R_max,_ (µm)	Minimize	17.8333
OR, (µm)	Minimize	17.3333
H, (µm)	Maximize	24.6667

**Fig. 50:**
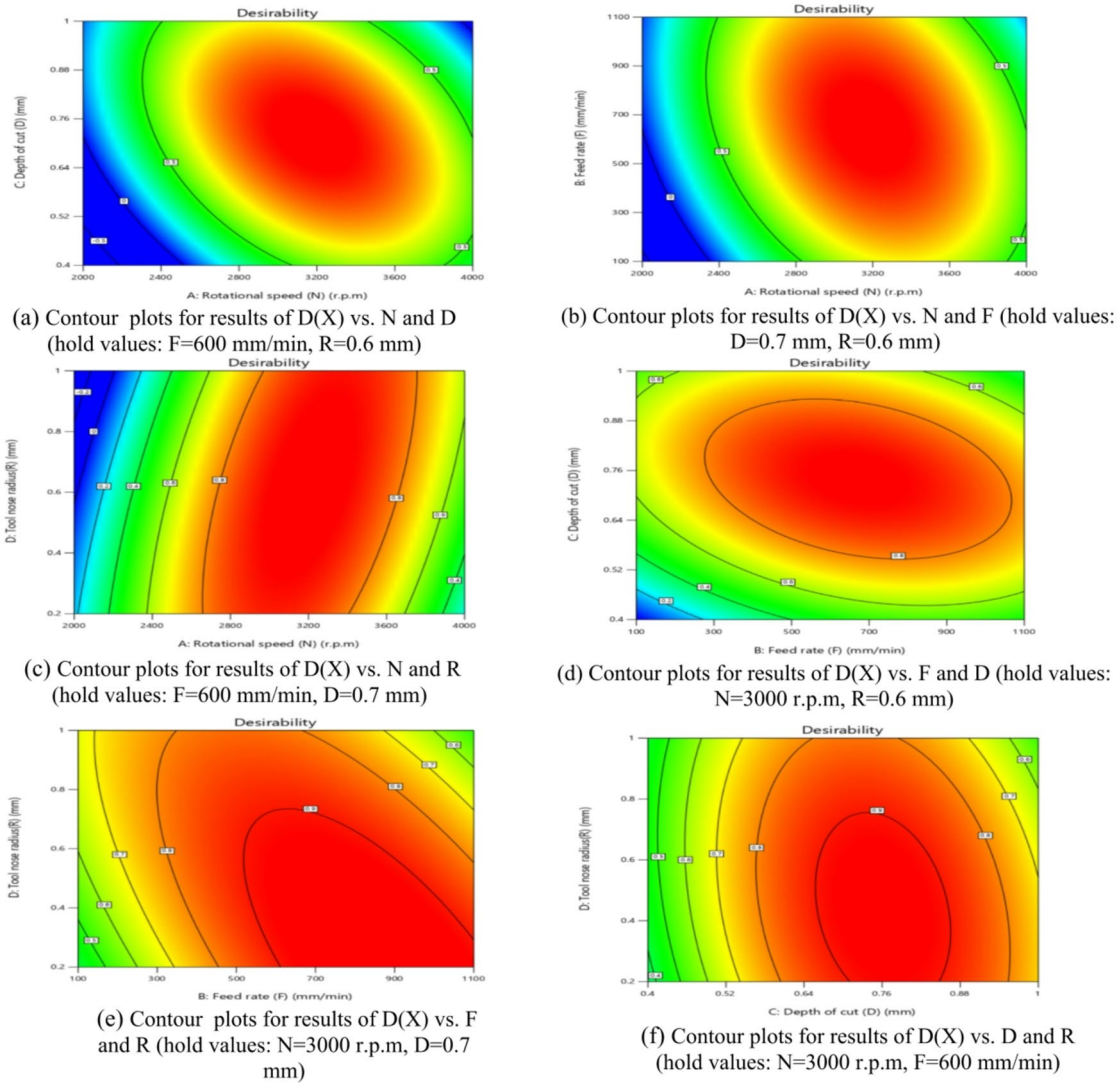
(a, b, c, d, e, f) Contour plots of the D(X) function for CK45 workpieces machined using TP parameters.

As shown in Fig. 50, desirability functions and contour plots were created to illustrate the impact and sensitivity of the results (a, b, c, d, e, f). The optimal solutions were found in the regions closer to the center of the plots in Fig. 54 (a, b, c, d). In these regions, the CD values exceeded 0.5, 0.5, 0.8, and 0.8, respectively, and increased as one approached the center points of the contour plots. Furthermore, the CD values for both Fig. 50 (e and f) were higher than 0.9, indicating that the most favorable zones were located in the middle of the graphs.

## Confirmation experiments

The final stage involves predicting and confirming the TP’s efficiency using the optimal conditions after selecting the TP settings within the ideal range. Trials are conducted using the previously determined optimal input parameter settings for MRR, TWR, Ra, R_max_, OR, and H to evaluate the TP while considering the ideal response magnitudes. Since this optimal parameter combination (*N* = 3000 rpm, F = 600 mm/min, D = 0.7 mm, *R* = 0.6 mm) was not included in the original training matrix, this experiment serves as a confirmatory physical validation using an independent dataset, thereby substantiating the practical robustness and predictive reliability of the proposed hybrid model.

According to the trial findings, the observed values for MRR, TWR, Ra, R_max_, OR, and H are 0.41 g/min, 1.23 × 10⁻⁵ g/min, 4.73 μm, 18.1 μm, 17.8 μm, and 24.9 μm, respectively.

Table 15 presents the experimental verification of the necessary equation for MRR, TWR, Ra, R_max_, OR, and H under optimal parametric conditions during the TP of CK45 steel. It also includes the anticipated optimal responses, experimental trial results, and error analysis. The errors between the predicted and trial magnitudes for MRR, TWR, Ra, R_max_, OR, and H range from 1.9%, 6.5%, 6.5%, 1.6%, 2.8%, and 1.2%, respectively. The values in Table 16 demonstrate that the calculated errors are minimal. These minor discrepancies indicate the high reproducibility of the study outcomes. and the error was calculated according to the following equation:

18 $$\mathbf{E}\mathbf{r}\mathbf{r}\mathbf{o}\mathbf{r}\mathbf{\%}=\left|\frac{\mathbf{E}\mathbf{x}\mathbf{p}\mathbf{e}\mathbf{c}\mathbf{t}\mathbf{e}\mathbf{d}\mathbf{o}\mathbf{p}\mathbf{t}\mathbf{i}\mathbf{m}\mathbf{i}\mathbf{u}\mathbf{m}\mathbf{r}\mathbf{e}\mathbf{s}\mathbf{p}\mathbf{o}\mathbf{n}\mathbf{s}\mathbf{e}-\mathbf{A}\mathbf{c}\mathbf{t}\mathbf{u}\mathbf{a}\mathbf{l}\mathbf{o}\mathbf{p}\mathbf{t}\mathbf{i}\mathbf{m}\mathbf{i}\mathbf{u}\mathbf{m}\mathbf{r}\mathbf{e}\mathbf{s}\mathbf{p}\mathbf{o}\mathbf{n}\mathbf{s}\mathbf{e}}{\mathbf{E}\mathbf{x}\mathbf{p}\mathbf{e}\mathbf{c}\mathbf{t}\mathbf{e}\mathbf{d}\mathbf{o}\mathbf{p}\mathbf{t}\mathbf{i}\mathbf{m}\mathbf{i}\mathbf{u}\mathbf{m}\mathbf{r}\mathbf{e}\mathbf{s}\mathbf{p}\mathbf{o}\mathbf{n}\mathbf{s}\mathbf{e}}\right|\mathbf{*}100$$

By empirically modeling all machining responses and verifying the selected optimal cutting parameters, it is possible to determine the predicted range of all machining responses as well as the range of parameters.

**Fig. 51:**
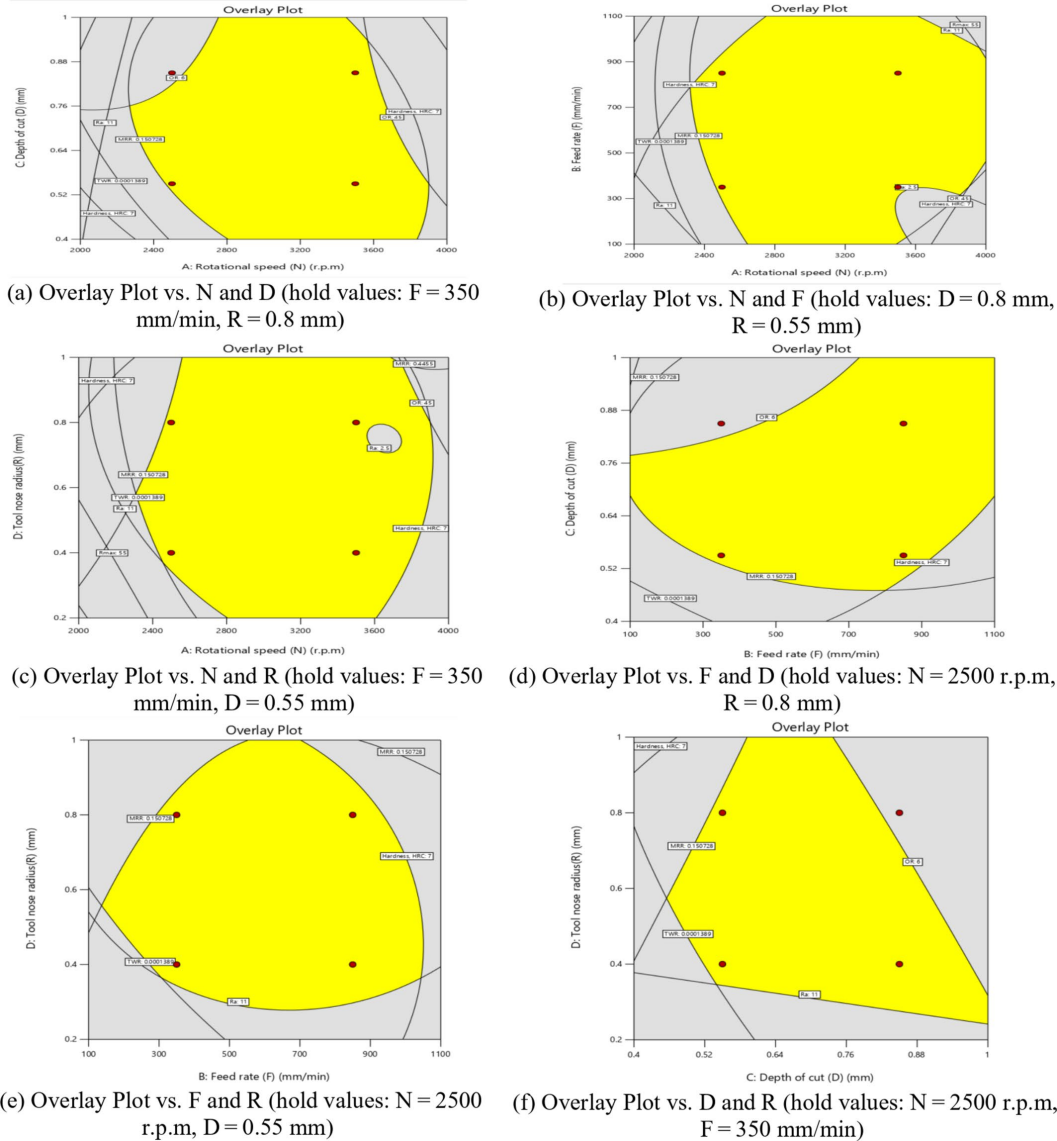
(a, b, c, d, e, f) Overlay Plot for multi-response for results Ck45 workpieces turned by rough (TP).

A relatively simple method for optimizing multiple responses with a limited number of process variables is to overlay the contour plots for each response^35^. The new response range was chosen from the confirmation report illustrated in Table 18, using the contours of 0.151 ≤ MRR ≤ 0.446, 9 × 10⁻⁶ ≤ TWR ≤ 0.00014, 2.5 ≤ Ra ≤ 11, 10 ≤ R_max_ ≤ 55, 6 ≤ OR ≤ 45, and 7 ≤ H ≤ 27. Figure 51 illustrates an overlay plot of the six response criteria (MRR, TWR, Ra, R_max_, OR, and H) situated in the yellow (light) zone within the grey region (a, b, c, d, e, f).

**Table 18:** Evaluation of the required models under optimal conditions for CK45 workpieces machined with turning parameters (TP).

Combination of controlling factors		Response	Expected optimum responses	Observed responses	Error (%)
N, (r.p.m)	3000	MRR, (gram/min)	0.41	0.413	1.9
F, (mm/min)	600	TWR, (gram/min)	1.15E-05	1.23 E-05	6.5
D, (mm)	0.70	Ra, (µm)	4.55	4.7	3.8
R, (mm)	0.60	R_max_, (µm)	17.8	18.1	1.6
		OR, (µm)	17.3	17.8	2.8
		H, (µm)	24.7	24.9	1.2

## Microstructural analyses

**Fig. 52:**
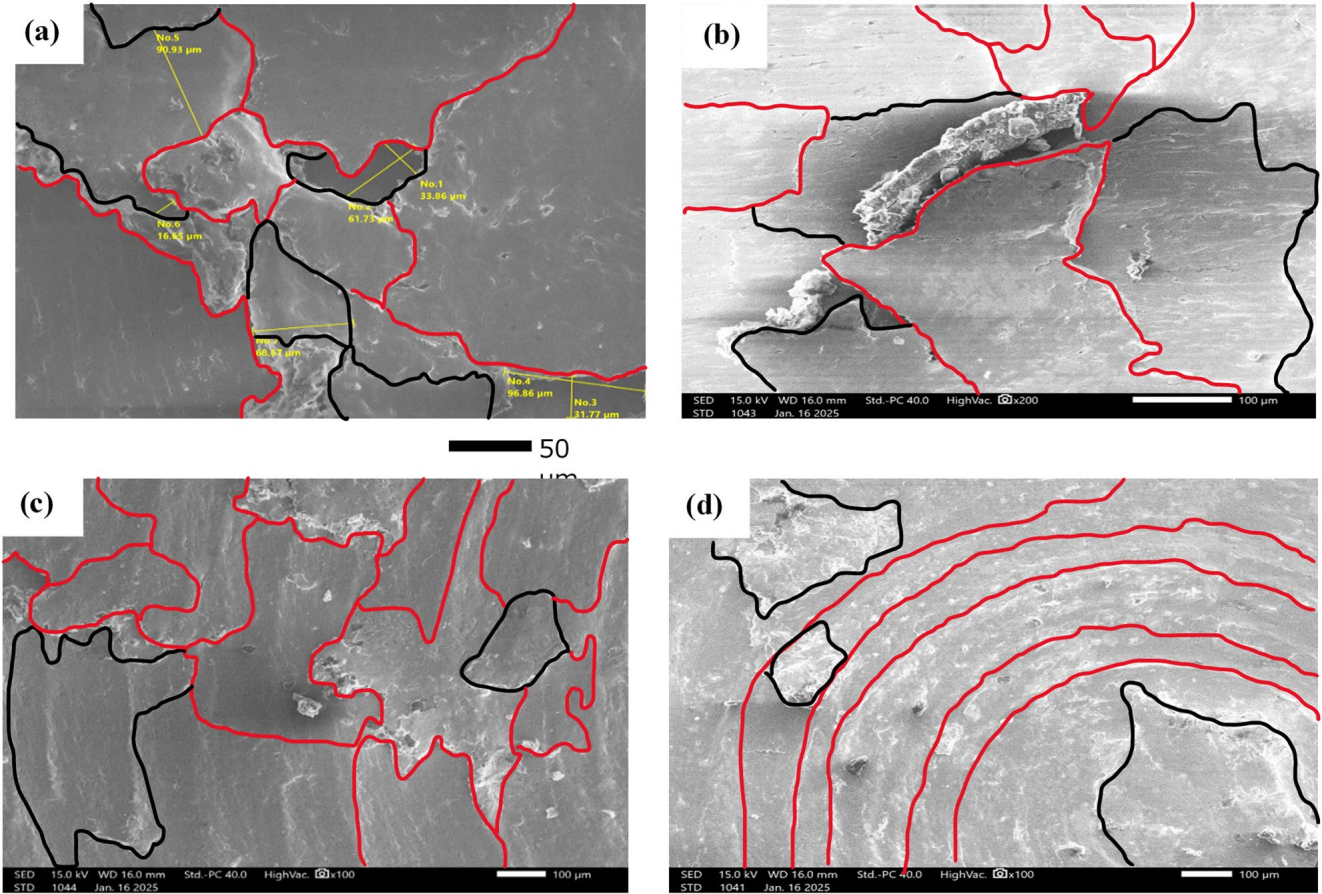
SEM photos of different positions (a) first position, (b) second position, (c) third position, and (d) fourth position of CK45 workpiece at bad (TP) machining conditions: *N*= 2000 r.p.m, F= 600 mm/min, D= 0.70 mm and *R*= 0.6 mm.

In Experiment 17, after the turning process of CK45 under machining conditions of *N* = 2000 r.p.m, F = 600 mm/min, D = 0.70 mm, and *R* = 0.6 mm, the surface morphology revealed a diverse range of irregular geometric formations. Notably, irregular dendritic structure-like structures of varying sizes, ranging from 90.93 μm to 16.65 μm, were observed (Fig. 52a–c), along with large semi-spherical (semicircular) formations (Fig. 52d). These irregularities suggest instability in the cutting process, potentially influenced by factors such as chip formation dynamics, tool-workpiece interactions, and thermal effects. The present of observation corresponds with the results reported by Zou, B et al^57^., and Ranganathan, et al^23^..

Further analysis of machining performance metrics confirmed that these instabilities contributed to poor surface quality and process inefficiencies. The MRR was recorded at 0.165582 g/min, while the TWR was measured at 0.0001389 g/min. Additionally, the Ra was alarmingly high at 11 μm, with R_max_ reaching 55 μm. Other critical parameters, such as OR at 20 μm and H at 12 μm, further indicated suboptimal machining conditions. The composite desirability score of 0 reflects an overall poor response in terms of machining efficiency and surface integrity, emphasizing the need for process optimization to enhance surface quality and tool performance while minimizing defects. This observation is agreement with^12–14^.

A key factor influencing machining efficiency is the rate of change in volume, which directly determines MRR. As shown in the MRR main effect plot (Fig. 23), MRR increases as N rises.

This is because machining time plays a crucial role in material removal, and with an increasing number of turns at maximum speed, the removal rate improves. Additionally, MRR increases as F rises, although at lower F values, the growth appears gradual. A similar trend is observed for depth of cut; since greater chip thickness can be removed, increasing D also enhances MRR. This observation is agreement with^38–41^.

The influence of key parameters on MRR is illustrated in Fig. 23a and f, showing that D, R, and F directly affect MRR. When D, F, and N are increased, MRR follows an upward trend.

The ANOVA table for MRR, presented in Table 6, further highlights the significance of these parameters, with N having the greatest impact, followed by F, D, and R. The table also reveals that while N and F significantly influence MRR, the effects of other factors are negligible.

If the p-values of the process parameters are less than 0.005 (α = 0.005) at a 99.13% confidence level, they are considered statistically significant; otherwise, they are deemed inconsequential.

These findings suggest that the machining instabilities observed in Experiment 17 as evidenced by surface irregularities and poor performance metrics are closely linked to process parameters. Optimizing N and F could significantly enhance machining efficiency while addressing these inefficiencies and surface quality concerns.

Implementing better parameter control strategies may lead to improved tool performance, reduced defects, and overall machining effectiveness. This observation is agreement with^58,61^.

**Fig. 53:**
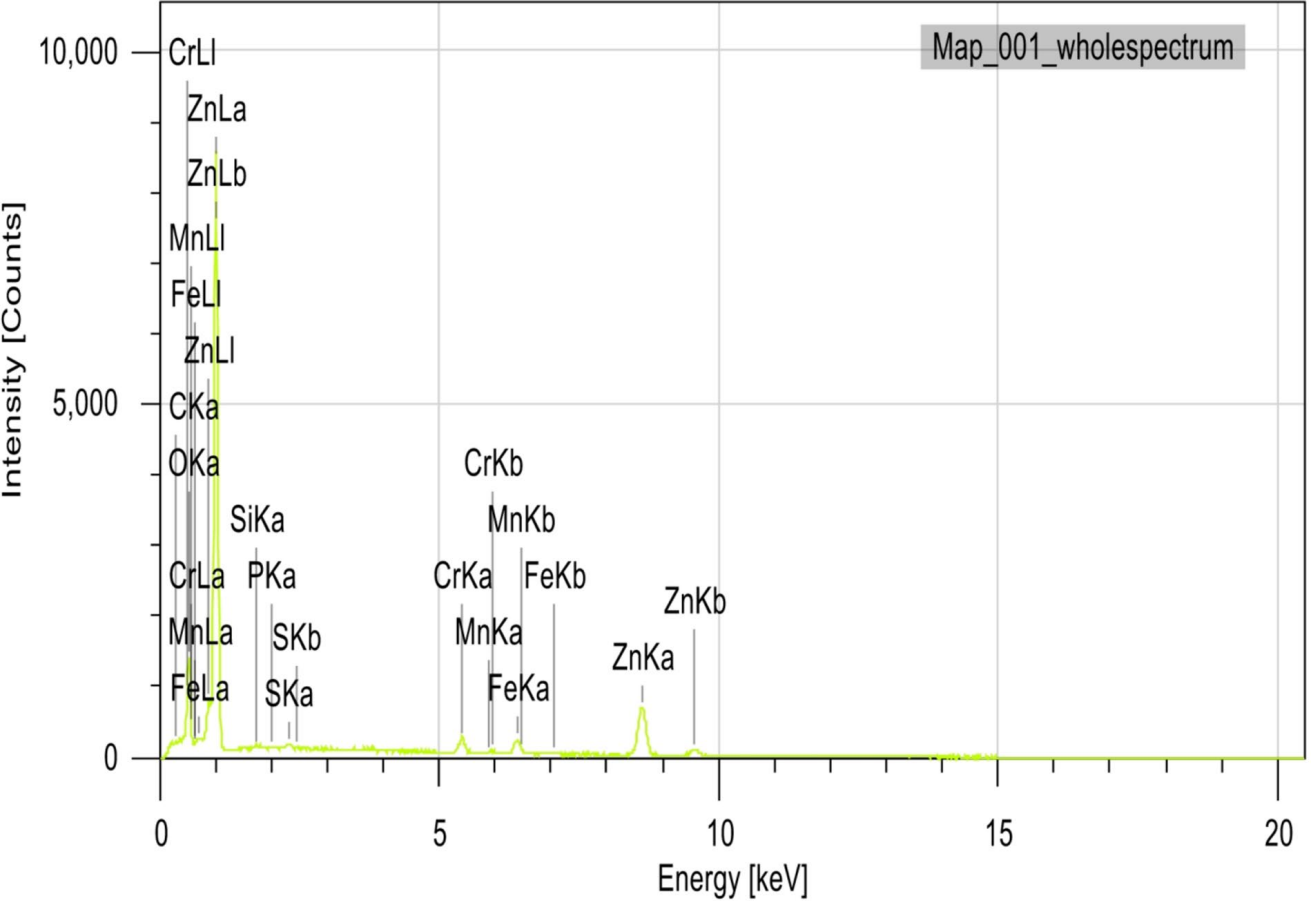
EDS analysis of fourth position of CK45 workpiece at bad (TP) machining conditions: *N* = 2000 r.p.m, F = 600 mm/min, D = 0.70 mm and *R* = 0.6 mm.

Figure 53 reveals the EDS analysis of the fourth position of the CK45 steel workpiece under unfavorable machining conditions (*N* = 2000 r.p.m, F = 600 mm/min, D = 0.70 mm, and *R* = 0.6 mm), providing valuable insights into its elemental composition. The EDS analysis reveals the presence of Fe, Mn, C, Si, Cr, and P, confirming their expected appearance in the chemical composition of the CK45 workpiece, as detailed in Table 1. Interestingly, the EDS analysis of the CK45 workpiece reveals the presence of Zn, originating from the coating layer of the single-point cutting tool insert. This residual Zn transfer contributes to high surface roughness for CK45 workpiece, highlighting its significant impact on machining performance.

**Fig. 54:**
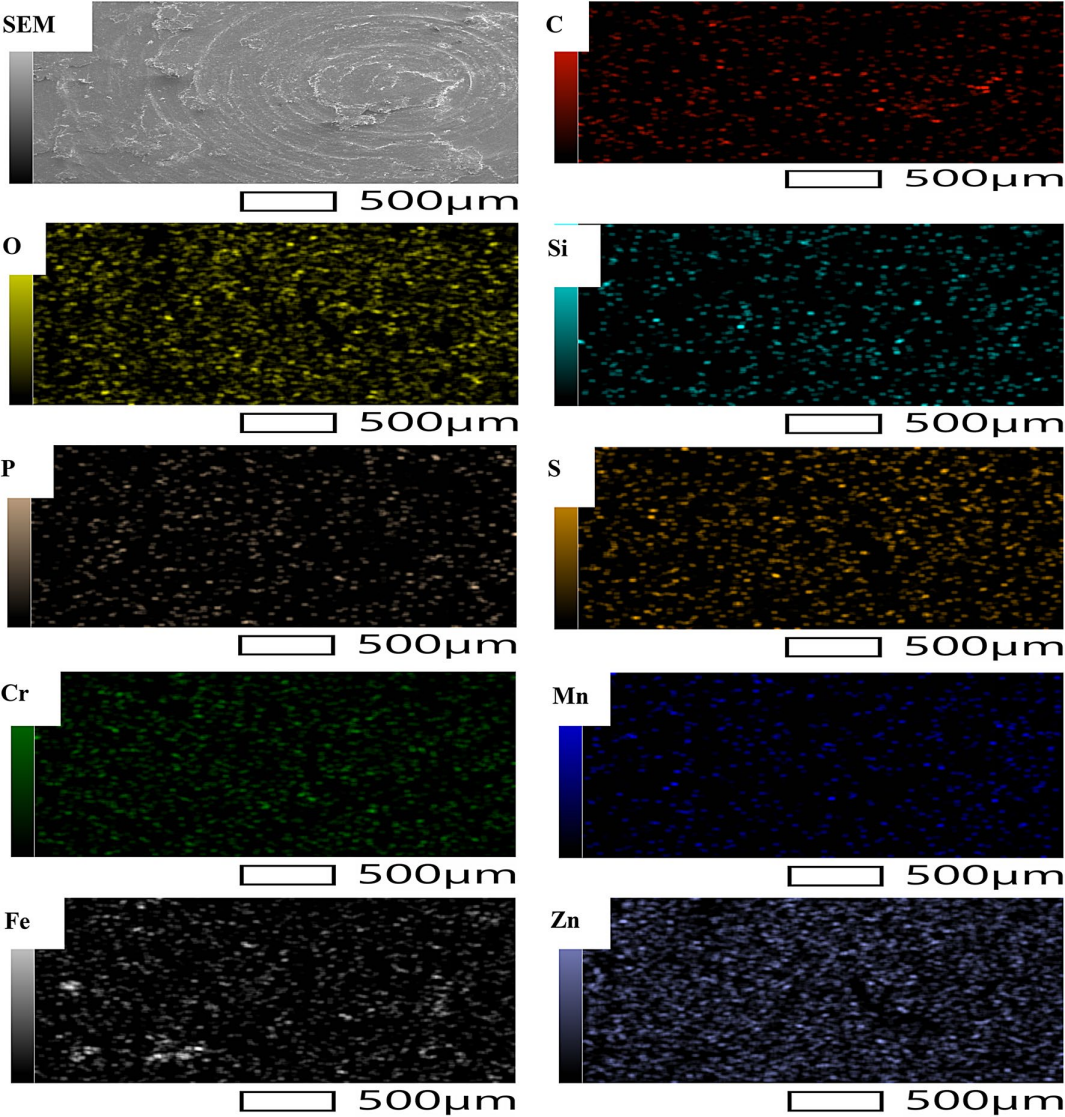
Map analysis of fourth position of CK45 workpiece at bad (TP) machining conditions: *N* = 2000 r.p.m, F = 600 mm/min, D = 0.70 mm and *R* = 0.6 mm.

Figure 54 presents the map analysis of the fourth position of the CK45 steel workpiece under unfavorable TP machining conditions: *N* = 2000 r.p.m, F = 600 mm/min, D = 0.70 mm, and *R* = 0.6 mm. According to Fig. 58, the distribution of C, O, Si, P, S, Cr, Mn, Fe, and Zn appears uneven and non-uniform. This can be attributed to several factors, including (i) unsuitable machining conditions, (ii) the formation of large semi-spherical structures and irregular dendritic formations, and (iii) high surface roughness.

**Fig. 55:**
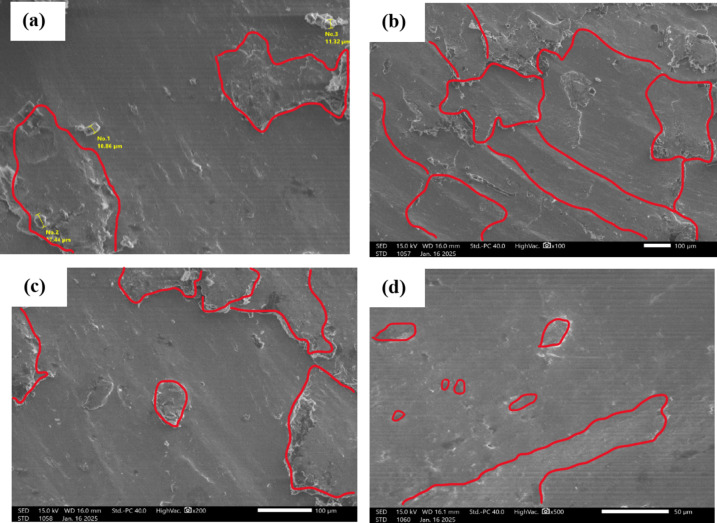
SEM photos of different positions (**a**) first position, (**b**) second position, (**c**) third position, and (**d**) fourth position of CK45 workpiece at optimum (TP) machining conditions: *N* = 3000 r.p.m, F = 600 mm/min, D = 0.70 mm and *R* = 0.6 mm.

Figure 55 showcases the results of the optimum turning experiment on CK45, conducted under machining conditions of *N* = 3000 r.p.m, F = 600 mm/min, D = 0.70 mm, and *R* = 0.6 mm. In contrast to the sample from Experiment No. 17 and other trials within the same experimental design, the surface morphology here exhibited a more refined and consistent array of regular geometric formations.

Most notably, dendritic structures of uniform size ranging from 10.86 μm to 17.44 μm were clearly visible (Fig. 55a–d), along with delicate, fine-scale geometrical patterns (Fig. 55d). The present of observation better than the results reported by panda et al^11^.,. These features indicate a stable cutting process, likely driven by the optimized machining parameters specifically the high rotational speed, moderate feed rate, and shallow depth of cut which contributed to the absence of surface cracks, voids, and craters. This observation is agreement with^38,41^.

A thorough analysis of the machining performance metrics revealed that the stability of the process played a pivotal role in achieving both exceptional surface quality and operational efficiency. The MRR consistently reached 0.40501 g/min, while the TWR remained impressively low at 1.15E-05 g/min, indicating minimal tool degradation. The surface integrity was further affirmed by a notably low Ra of 4.55 μm, coupled with a modest R_max_ of 17.83 μm. Additional parameters, such as the OR of 17.33 μm and H of 24.66 μm, provided deeper insight into the precision of the machining process under these specific conditions. Most significantly, the composite desirability score of 0.832, as shown in Fig. 49, highlights an optimized balance between machining efficiency and surface finish. These findings emphasize the crucial need for continuous refinement of process parameters to ensure superior tool performance and minimize the occurrence of surface defects. This observation is agreement with^12,14^.

**Fig. 56:**
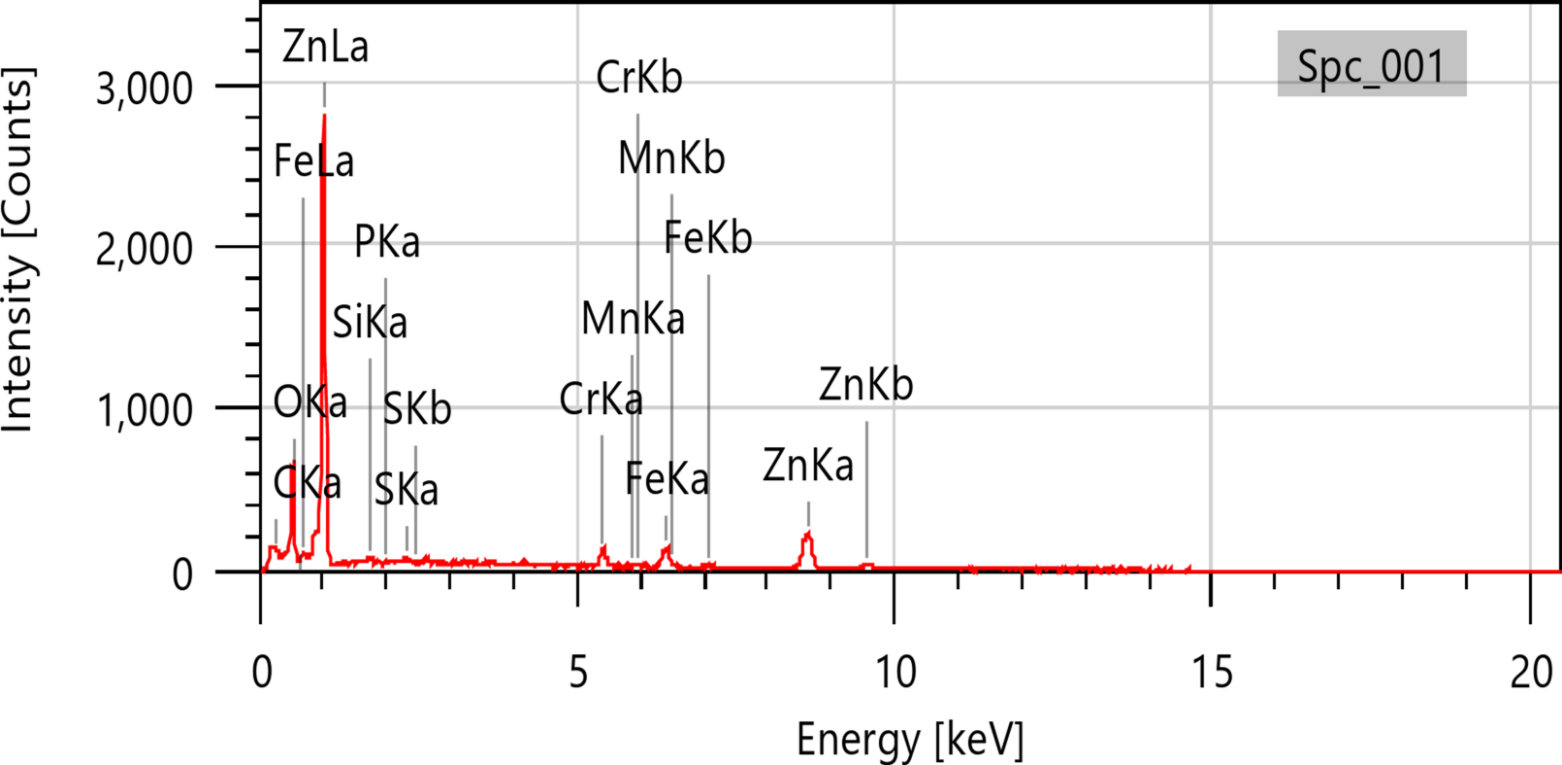
EDS analysis of second position of CK45 workpiece at (TP) optimum machining conditions at *N* = 3000 r.p.m, F = 600 mm/min, D = 0.70 mm and *R* = 0.6 mm.

Figure 56 presents the EDS analysis conducted at the fourth position of the CK45 steel workpiece, which was machined under optimal conditions (*N* = 3000 r.p.m, F = 600 mm/min, D = 0.70 mm, and *R* = 0.6 mm), highlighting the surface’s elemental composition. The analysis confirmed the presence of essential elements Fe, Mn, C, Si, Cr, P, and Zn all of which are consistent with the expected chemical composition of CK45, as shown in Table 1. It is noted that the presence of a small amount of Zn causes to reduce surface roughness and high dimensional accuracy of the workpiece.

**Fig. 57:**
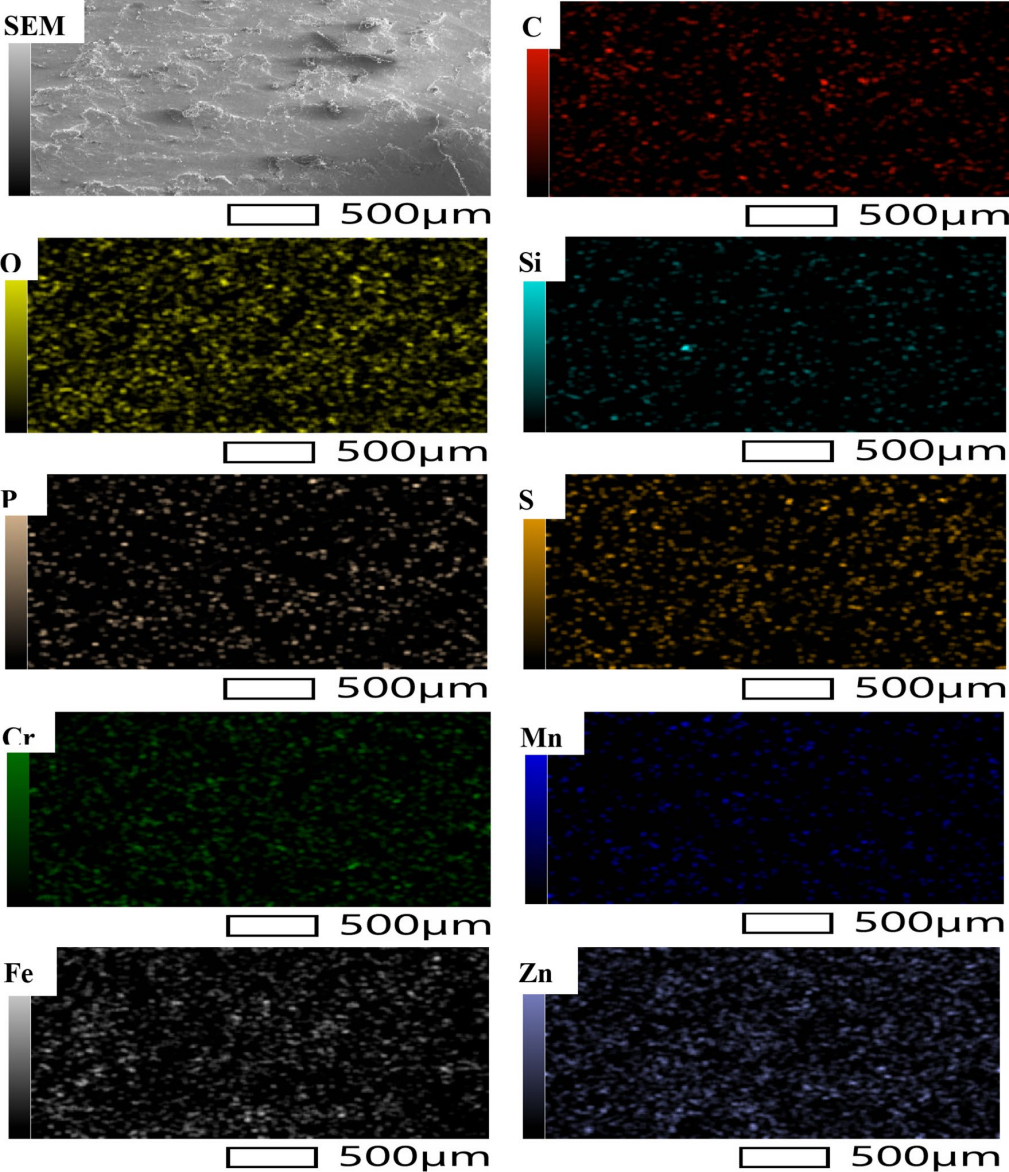
Map analysis of second position of CK45 workpiece at optimum (TP) machining conditions at *N* = 3000 r.p.m, F = 600 mm/min, D = 0.70 mm and *R* = 0.6 mm.

Figure 57 illustrates the map analysis of the CK45 steel workpiece in its second position, processed under favorable (TP) machining parameters: *N* = 3000 r.p.m, F = 600 mm/min, D = 0.70 mm, and *R* = 0.6 mm. The figure shows a uniform and consistent distribution of elements C, O, Si, P, S, Cr, Mn, Fe, and Zn. This uniformity can be attributed to several factors: (i) optimal machining conditions, (ii) the presence of well-defined geometric and dendritic structures, and (iii) low surface roughness.

**Fig. 58:**
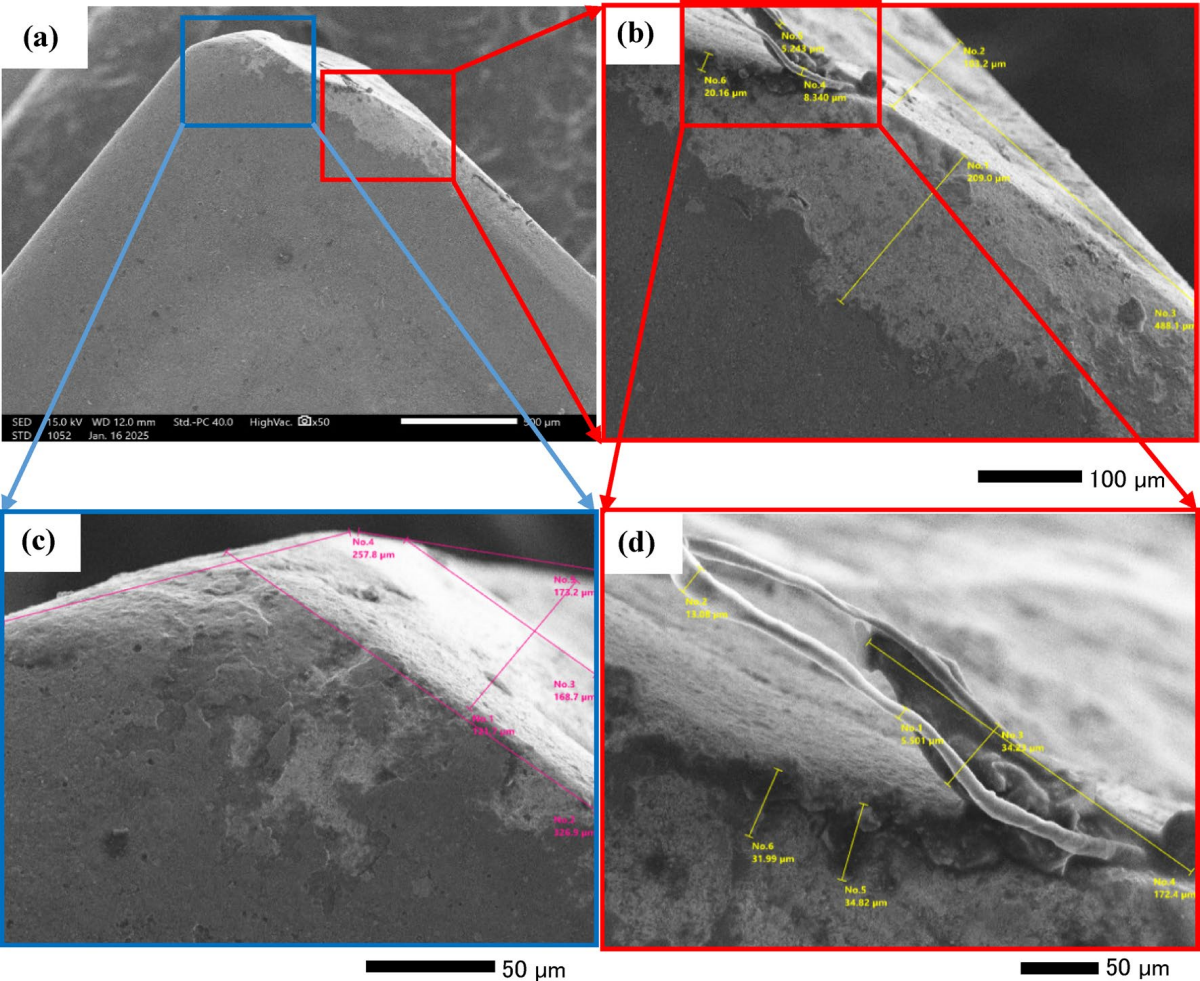
contrasts sharply with the results in Fig. 49, showcasing SEM imagery of the CBN insert taken under optimal (TP) machining conditions for a CK45 steel workpiece.

Figure 58 presents a series of SEM images captured at various locations on the CBN insert under unfavorable (TP) machining conditions applied to a CK45 steel workpiece, in experiment 17 with parameters set at N of 2000 r.p.m, F of 600 mm/min, D of 0.70 mm, and R of 0.6 mm. The microstructural analysis reveals notable wear phenomena: micro-agglomerates and adhered chips from workpiece were observed along the flank and cutting edge of the insert, measuring up to 172.4 microns in size, while micro-grooves with an average width of 34.23 microns were also identified (Figs. 58b, d). Interestingly, the nose of the insert exhibited distinct engraving patterns, likely a result of progressive wear. This degradation is further supported by a measured wear rate of approximately 0.0001389 g/min, as illustrated in Fig. 58a and c. The present of observation corresponds with the results reported by Wang et al^50^., and Panda, et al^11^.,. These observations highlight the adverse effects of suboptimal machining parameters on tool integrity and surface interaction.

Figure 58. SEM photos of different positions (a) first position, (b) second position, (c) third position, and (d) fourth position of (CBN) insert at bad TP machining conditions for CK45 workpiece at *N* = 2000 r.p.m, F = 600 mm/min, D = 0.70 mm and *R* = 0.6 mm.

This experiment, conducted with ideal parameters N of 3000 r.p.m, F of 600 mm/min, D of 0.70 mm, and R of 0.6 mm highlights the benefits of precision tuning in machining operations.

**Fig. 59:**
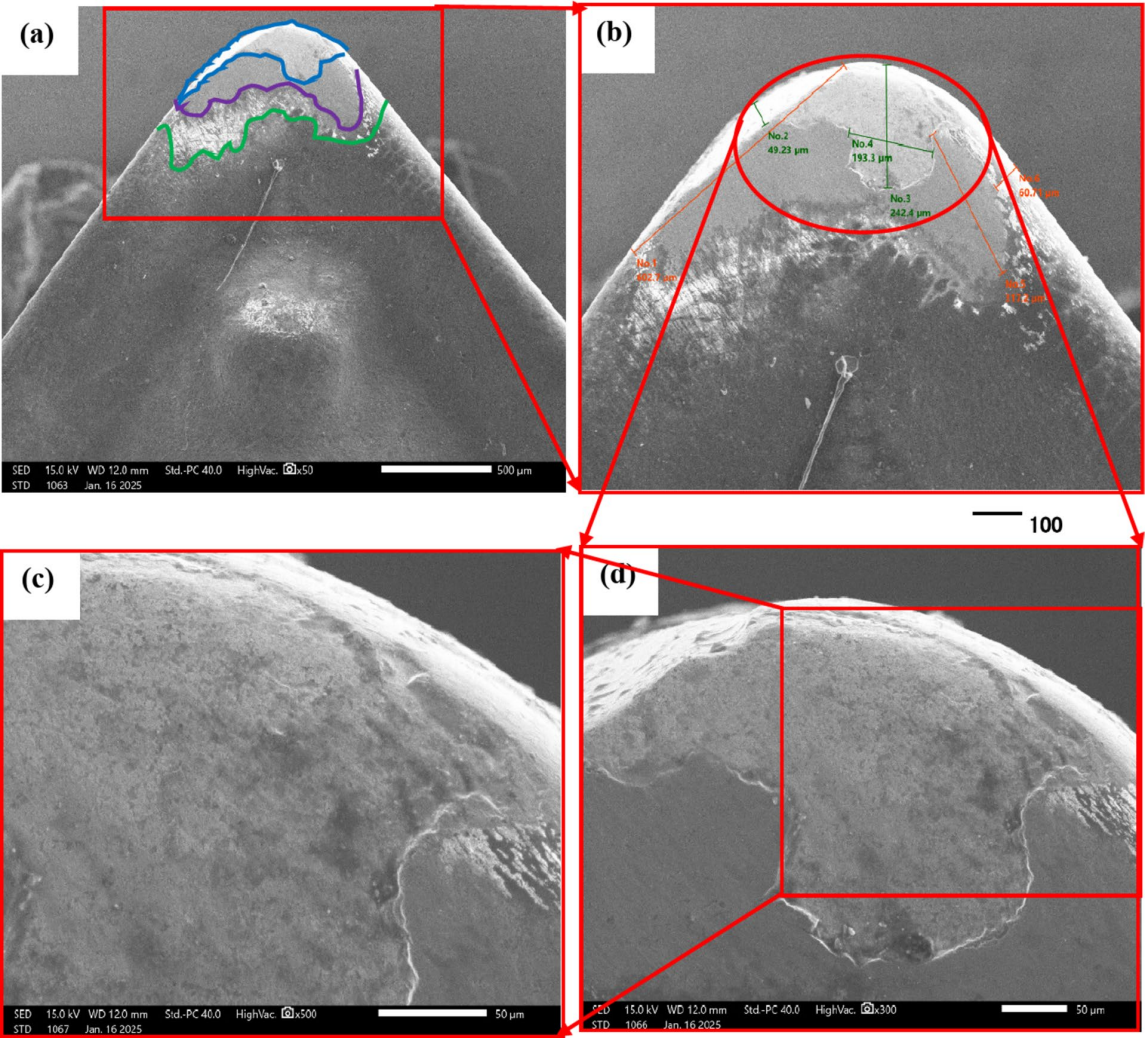
SEM photos of different positions (a) first position, (b) second position, (c) third position, and (d) fourth position of (CBN) insert at optimum (TP) machining conditions for CK45 steel workpiece at *N* = 3000 r.p.m, F = 600 mm/min, D = 0.70 mm and *R* = 0.6 mm.

Under these favorable conditions, tool wear was minimal. SEM images (Figs. 59a–d) reveal smooth, clean edges with no significant micro-agglomerates or chip adhesion, the largest detected particle measuring only 49.23 microns. Though micro-grooves were observed, their dimensions 242.4 microns in length and 193.3 microns in width indicate controlled material interaction rather than aggressive wear. The cutting edge and flank retained their sharpness, and the insert nose showed no signs of engraving or deformation, confirming negligible material loss. The present of observation corresponds with the results reported by Singh, et al^49^., and Panda, et al^11^., 

These results visually emphasize the impact of optimized machining settings: not only is tool integrity preserved, but surface interactions remain stable and controlled. The nearly imperceptible wear rate confirms that proper parameter selection is critical for extending tool life and ensuring high-quality surface finishes in industrial applications. This observation is agreement with^59–64^.

## Conclusions

Turning of shafts used in calibration applications encounters several challenges, including residual stresses, excessive tool wear, poor surface integrity, and dimensional inaccuracies. To solve these problems, a hybrid RSM–ANN prediction-based modeling and optimization framework was successfully developed for the precision turning of CK45 steel. The integrated approach effectively captured the complex nonlinear relationships between the key cutting parameters spindle speed, feed rate, depth of cut, and tool nose radius and multiple machining responses, including MRR, TWR, Ra, R_max_, OR, and H. The results underline the metrological robustness and predictive precision of the proposed models, as detailed below:

1. High model fidelity was achieved, with strong correlations among experimental and predicted responses (R² > 0.95), confirming the reliability of the developed RSM-based regression models.
2. MRR was mainly influenced by N and F, whereas TWR was affected by the quadratic and interaction effects of N and D.
3. Ra, R_max_, OR, and H exhibited complex dependencies on cutting parameters and tool geometry, reflecting the intricate interactions in precision turning.
4. Optimal cutting conditions (*N* = 3000 rpm, F = 600 mm/min, D = 0.70 mm, *R* = 0.60 mm) resulted in minimal deviations (< 6.5%), refined dendritic microstructures (10.86–17.44 μm), and low tool wear (1.23 × 10⁻⁵ g/min).
5. ANN models delivered higher predictive accuracy compared with RSM. Single-output ANN networks showed strong agreement with the experimental outcomes. Multi-output ANN and RSM were less effective than the single-output ANN in forecasting the results. ANNs operate as complex nonlinear systems of weights and biases that cannot be easily expressed in a straight forward mathematical form. On the other hand, RSM employs a quadratic polynomial equation, offering a clear and explicit mathematical model. This form directly reflects the individual influence of parameters as well as their combined interactions, making it useful for locating optimal conditions.
6. ANN models outperformed RSM in predictive accuracy, while RSM maintained interpretability, and the integrated modeling approach ensured reproducibility and reliability of measurements.
7. The developed models and metrological insights provide a validated foundation for process optimization, quality assurance, and improved measurement reliability in turning CK45.

Overall, this research advances the state-of-the-art in measurement-based process characterization by demonstrating how hybrid ANN–RSM modeling can enhance measurement accuracy, uncertainty evaluation, and surface morphology interpretation in high-precision turning applications.

## Supplementary Information

Below is the link to the electronic supplementary material.

Supplementary Material 1

## Data Availability

The datasets generated and/or analysed during the current study are available in the [Supplementary material].

## Abbreviations

ANOVA: Analysis of Variance
CNC: Computer Numerical Control
CD: Composite desirability
MRR: Metal removal rate
TWR: Tool wear rate
Ra: Arithmetic average surface roughness
R_max_: Maximum Roughness Depth
OR: Roundness error
H: Hardness
RSM: Response-surface-methodology
RS: Response surface
SR: Surface roughness
(CCD): Central Composite Design
DF: Degree of Freedom
di: Desirability for response i
V: Cutting speed
N: Rotational speed, (r, p,m)
F: Feed rate
D: Depth of cut
R: Tool nose radius
(CBN): Cubic Boron Nitride
HAZ: Heat affected zone
Hi: Value represents the highest bound for response i
(EPAHT): electric pulse-assisted hard turning
R^2^: Determination coefficient
MS: Mean square
SS: Sum square
(S/N): Signal to Noise ratios
(UVAT): ultrasonic vibration-assisted hard turning
ANN: Artificial Neural Network
LT: Layer Thickness
MAPE: Mean Absolute Percentage Error
MSE: Mean Squared Error
RMSE: Root Mean Squared Error
SR: Surface Roughness
TP: Turning process
SI: Sensitivity index
