# Correction: Hybrid intelligent RSM–ANN modeling and optimization of precision turning of CK45 steel for calibration devices

**DOI:** 10.1038/s41598-026-60842-x

**Published:** 2026-07-10

**Authors:** W. M. Farouk, Amer G. Ahmed, Mohammed Gamil, Farah Tamer Nasser, Mohamed Abu-Okail, Mamdouh I. Elamy

**Affiliations:** 1 Mechanical Engineering Department, Faculty of Engineering (Benha), Benha University, Benha, Egypt; 2 Nuclear Metallurgy Department, Atomic Energy Authority, Cairo, Egypt; 3 Department of Mechanical Engineering, Faculty of Engineering-Shoubra, Benha University, Cairo, Egypt; 4 Industrial Engineering Department, College of Engineering, Northern Border University, Arar, Saudi Arabia; 5 Department of Production Engineering and Mechanical Design, Faculty of Engineering, Menoufia University, Shebin El-Kom, Egypt; 6 Korean Egyptian Faculty for Industry and Energy Technology, Beni-Suef Technological University, Beni-Suef, 62521 Egypt; 7 Mechatronics Technology Department, Higher Technological Institut, Beni-Suef, Egypt

Correction to: *Scientific Reports* 10.1038/s41598-026-43388-w, published online 02 April 2026

In the original version of this Article, Figure  54 was omitted in the PDF but was included with the initial submission.

In addition, Mamdouh I. Elamy was incorrectly affiliated with Affiliation 5. The correct affiliation is Affiliation 4: ‘Industrial Engineering Department, College of Engineering, Northern Border University, Arar, Saudi Arabia.’

The original Article has been corrected.

