# Supplementary material for: Hybrid intelligent RSM–ANN modeling and optimization of precision turning of CK45 steel for calibration devices
Source: Sci Rep. 2026 Apr 2;16:11358. doi: 10.1038/s41598-026-43388-w (PMC13049169; doi:10.1038/s41598-026-43388-w)
Supplement: Supplementary file 1 — Supplementary Material 1 [file 41598_2026_43388_MOESM1_ESM.pdf]

Supplementary file

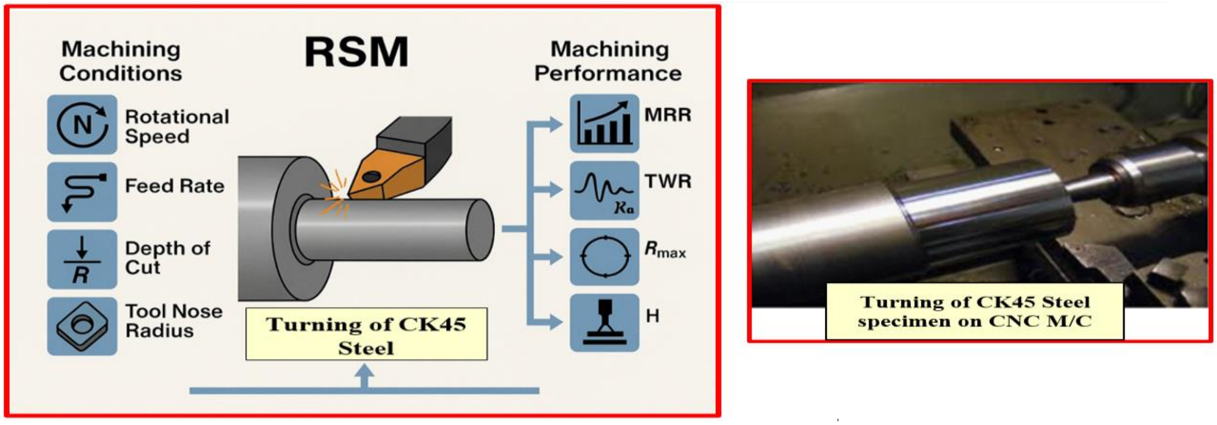

S1: Schematic diagram of turning machining conditions and performance of CK45 by using response surface methodology.

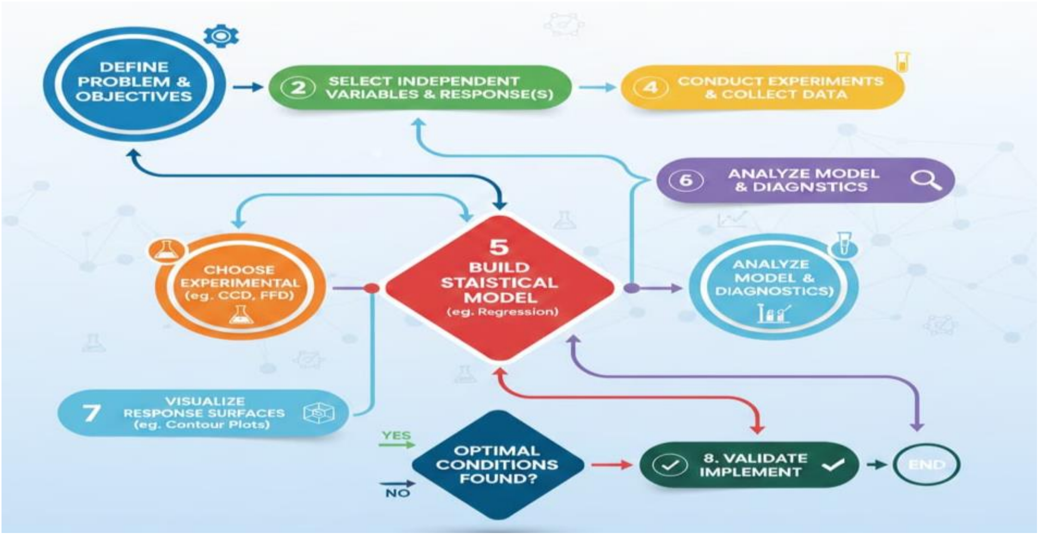

S2: Flow chart of RSM procedure.

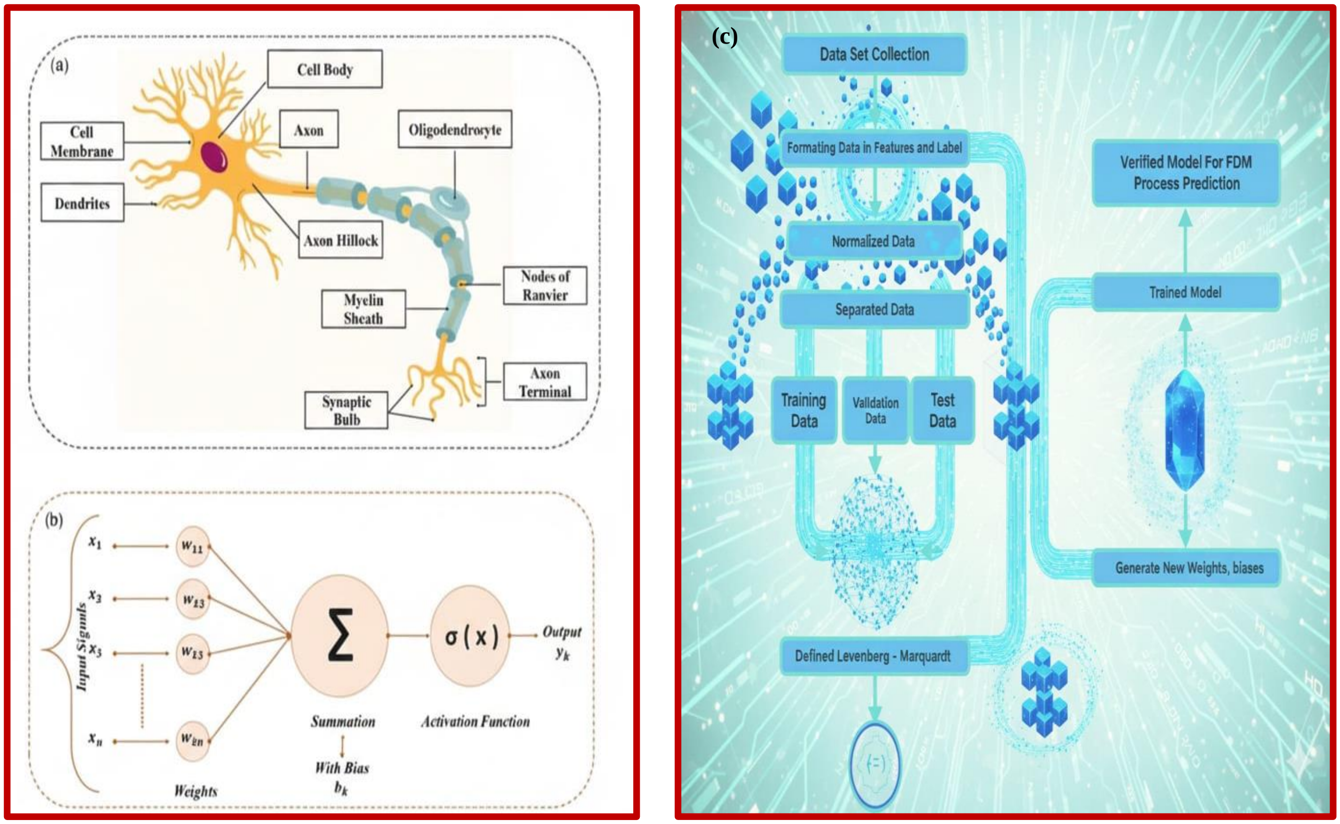

S3: Schematic illustration of (a), The fundamental components of an artificial neuron [20], (b) the mathematical representation of ANN [20], and the overall workflow of the present study for predicting responses of precision turning of CK45 using ANN algorithm.

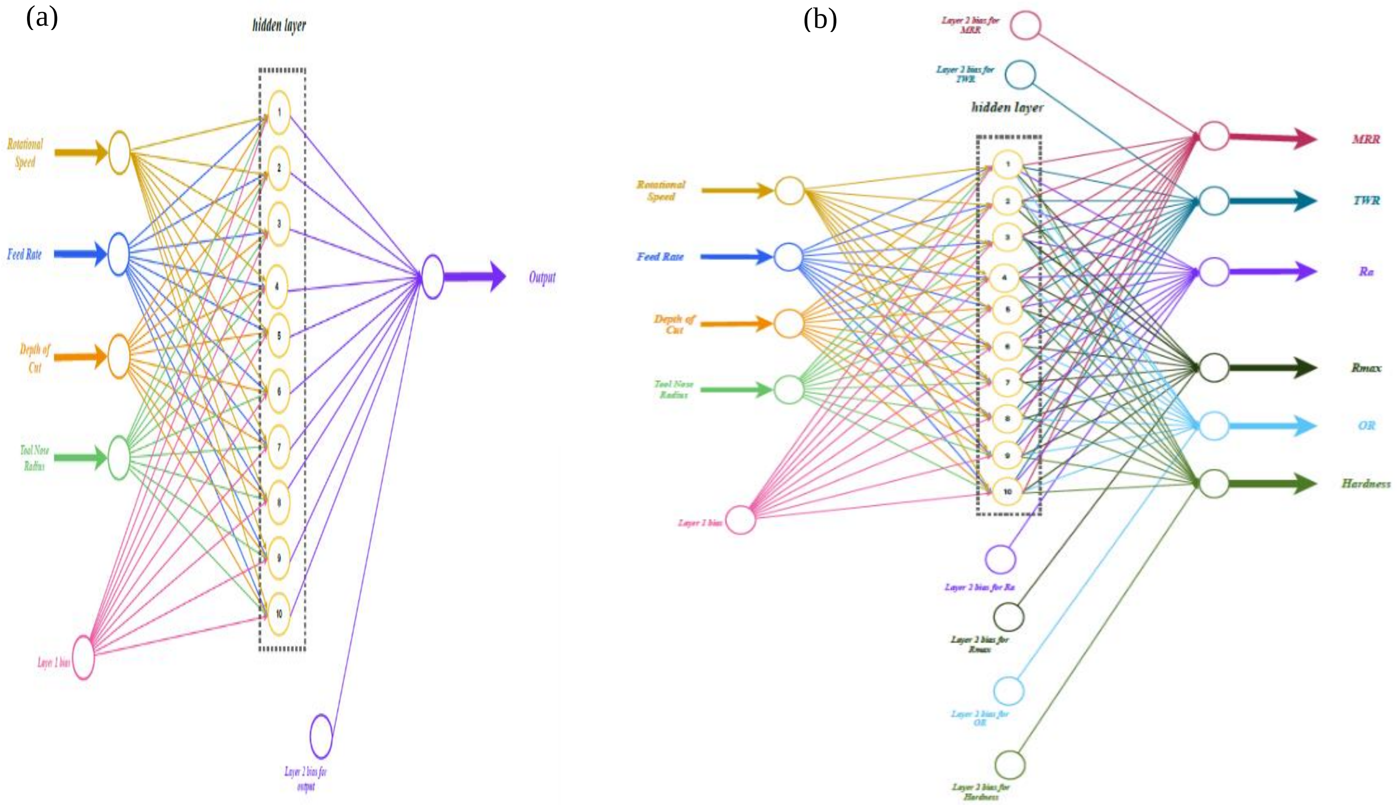

S4: (a) The 4-10-1 Single Output ANN (b) The 4-10-6 Multi Output ANN

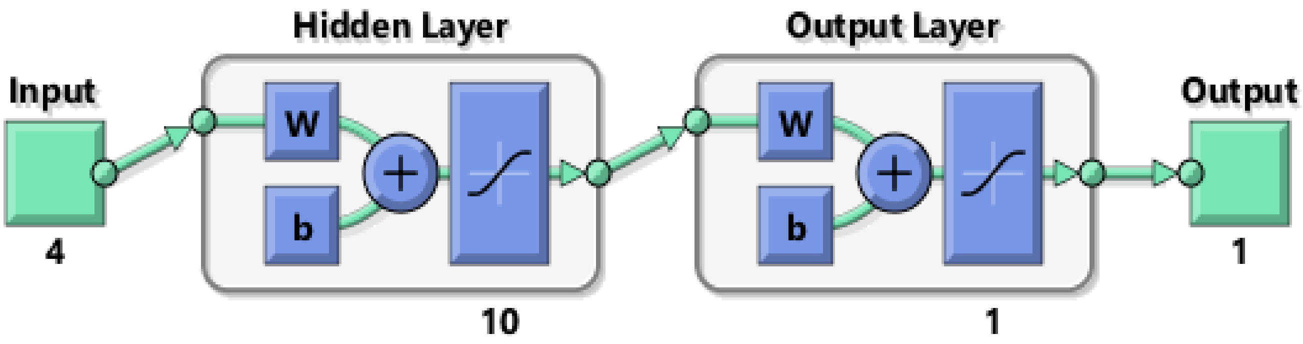

S5: Hidden Tier and Output Tier Tansig Function Representation for a Single Output.

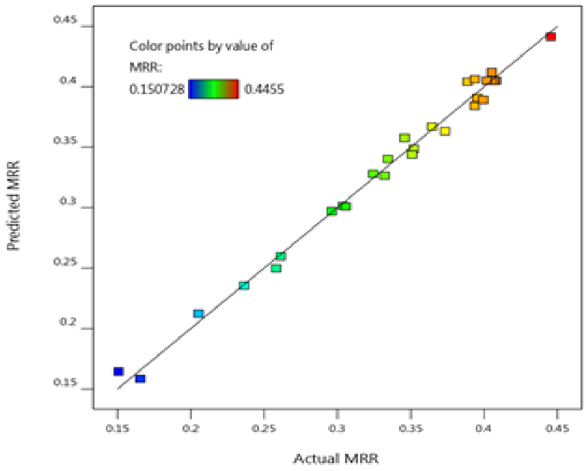

(a) Forecasted vs measured magnitudes of MRR (CK45)

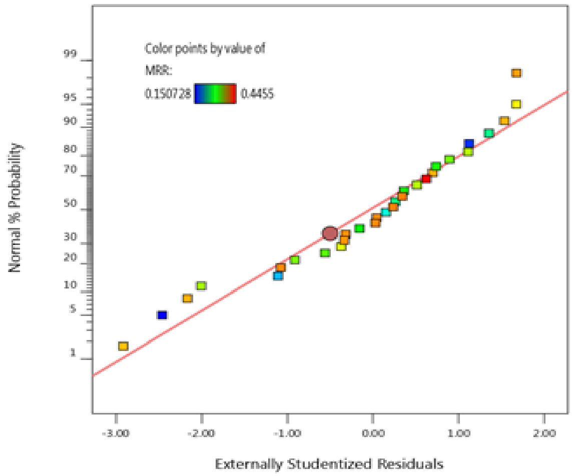

(b) Effects of residuals plot for MRR (CK45)

S6: Schematic diagnostics of MRR.

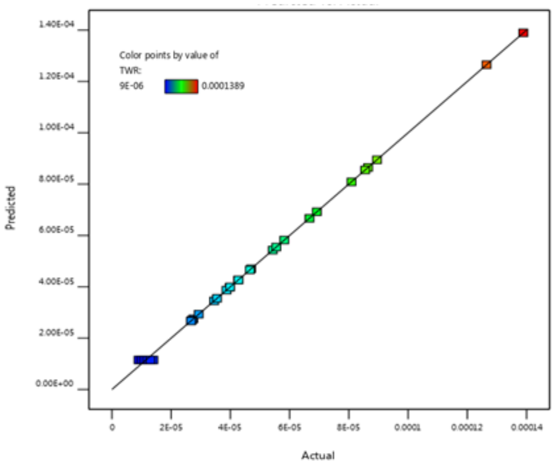

(a) Forecasted vs measured magnitudes of TWR (CK45)

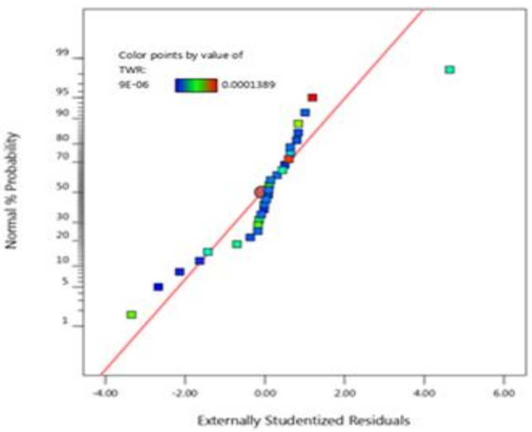

(b) Effects of residuals plot for TWR (CK45)

S7: Schematic diagnostics of TWR.

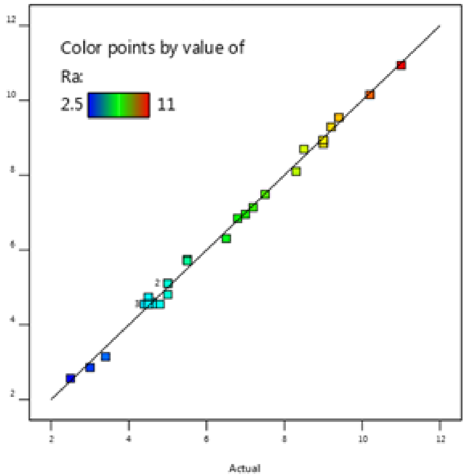

(a) Forecasted vs measured magnitudes of R_a (CK45)

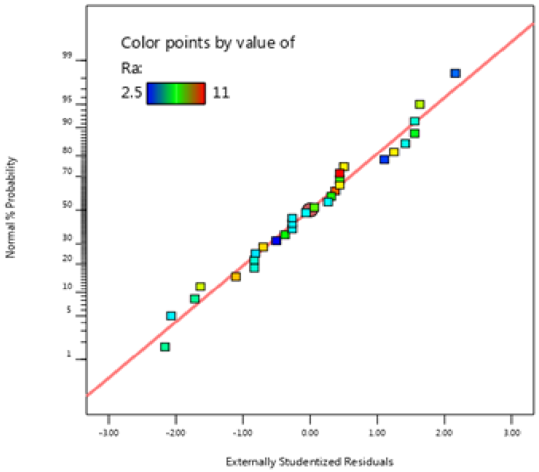

(b) Effects of residuals plot for R_a (CK45)

S8: Schematic diagnostics of R_a.

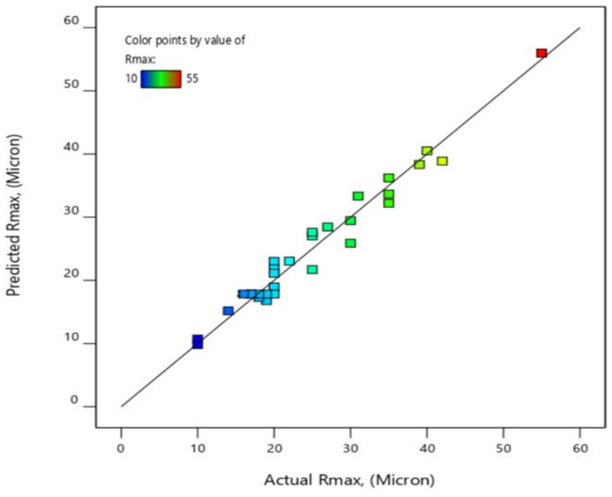

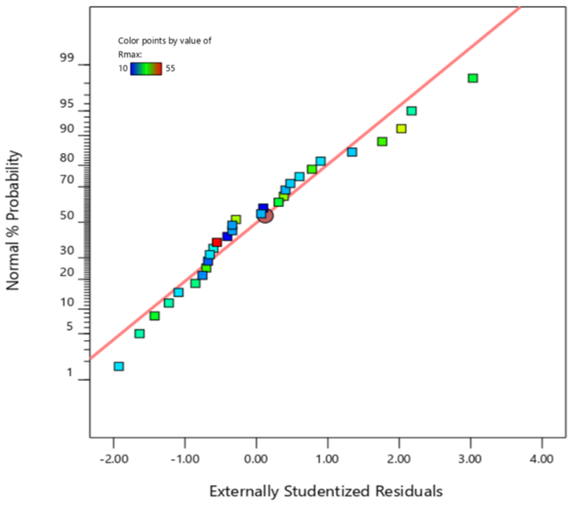

(a) Forecasted vs. measured magnitudes of R_{max} (CK45) (b)Effects of residuals plot for R_{max} (CK45)

S9: Schematic diagnostics of R_{max}.

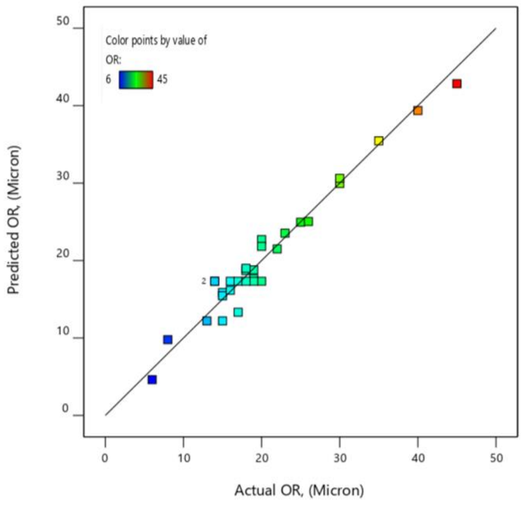

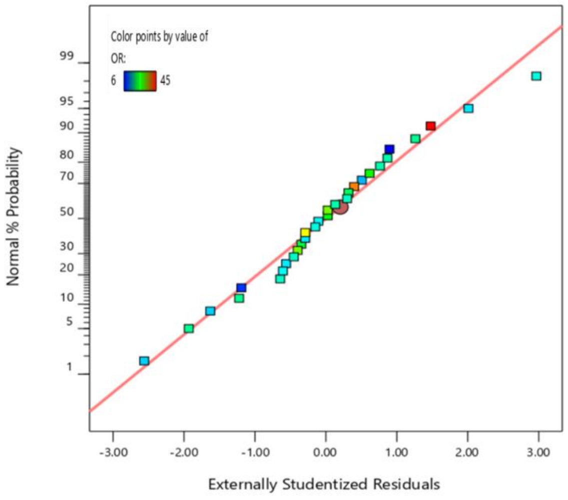

(a) Forecasted vs measured magnitudes of OR (CK45) (b) Effects of residuals plot for OR (CK45)

S10: Schematic diagnostics of OR.

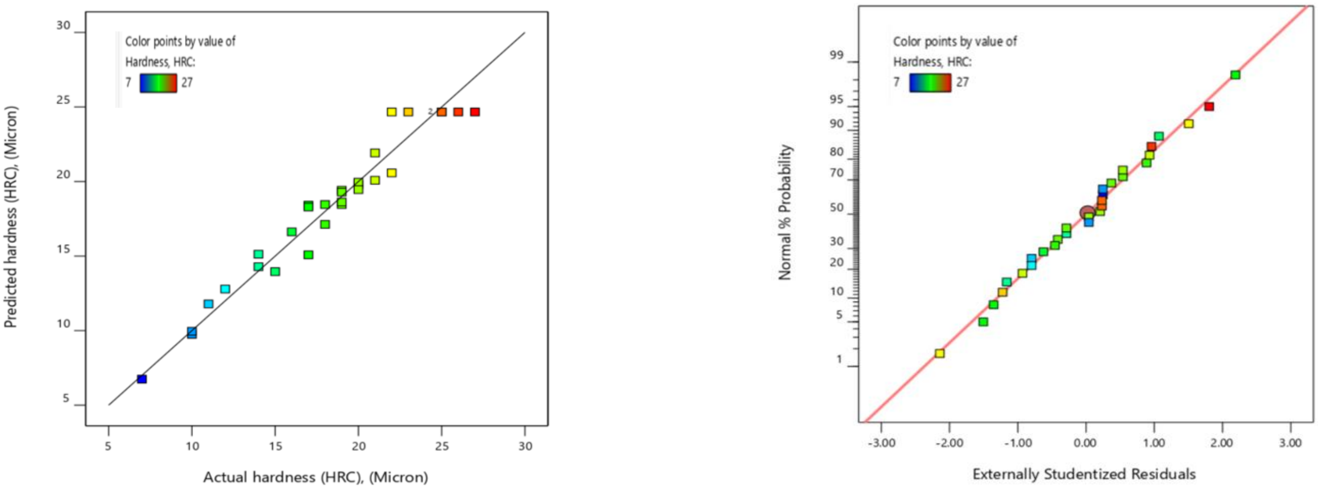

(a) Forecasted vs measured magnitudes of H (CK45)

(b) Effects of residuals plot for H (CK45)

S11: Schematic diagnostics of H.

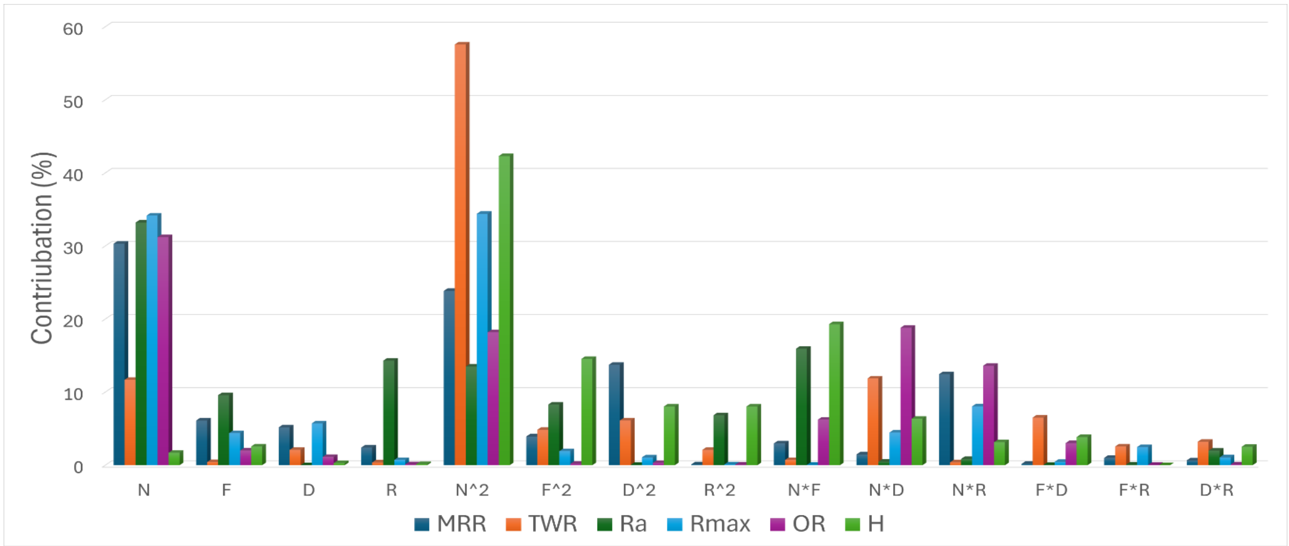

S12: SI or contribution % of machining parameters on the responses of the TP for CK45.

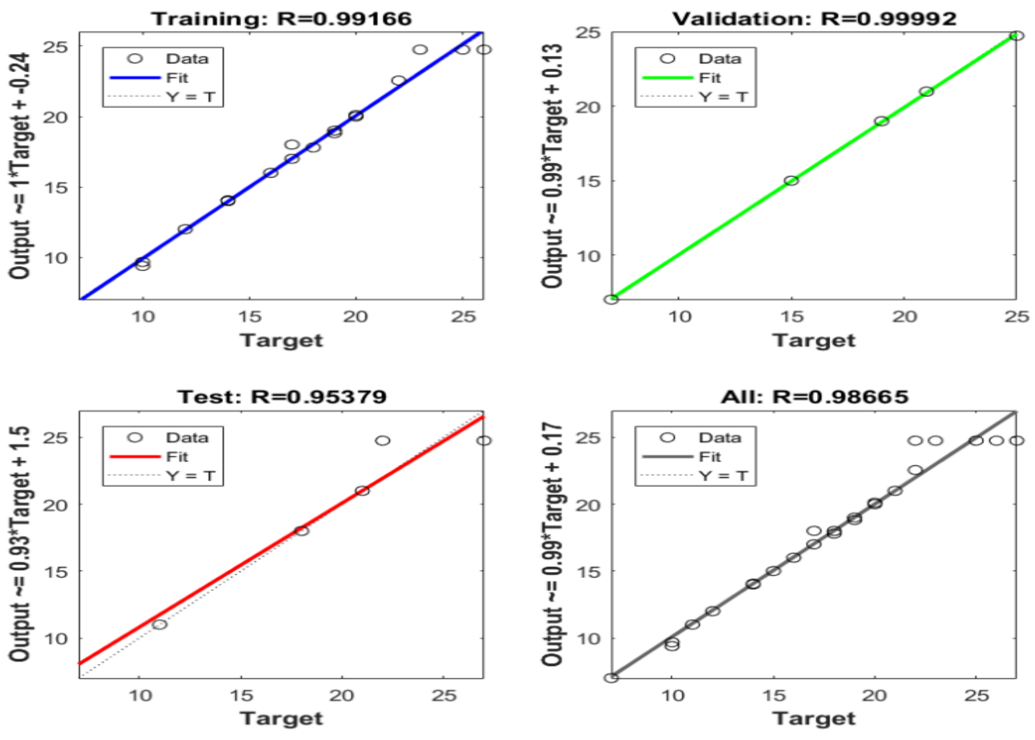

S13: Regression of Training, Verification, Evaluation data sets for H.

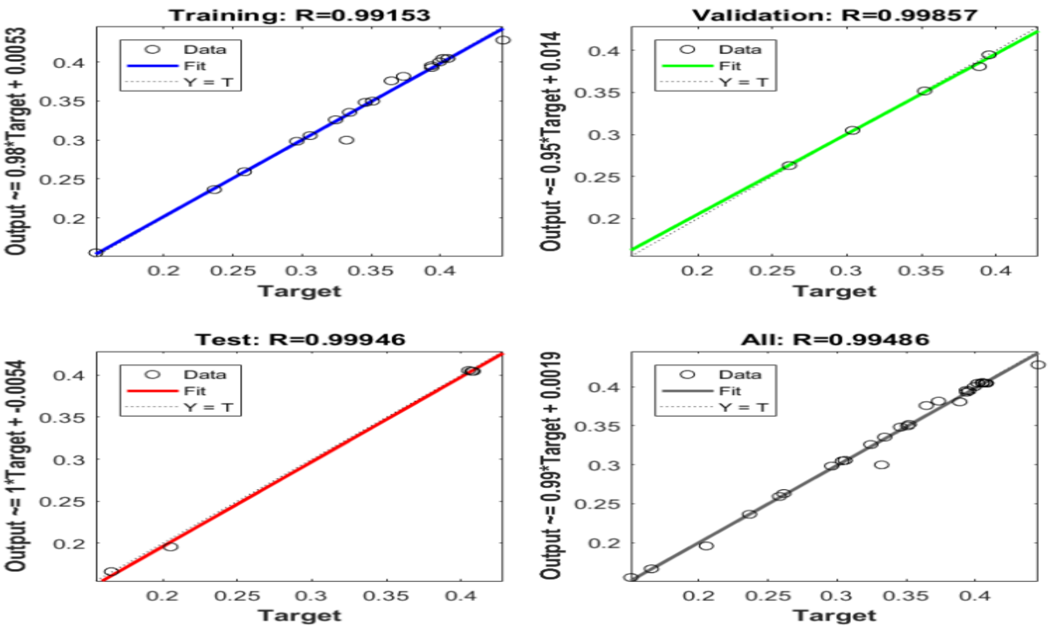

S14: Regression of Training, Verification, Evaluation data sets for MRR.

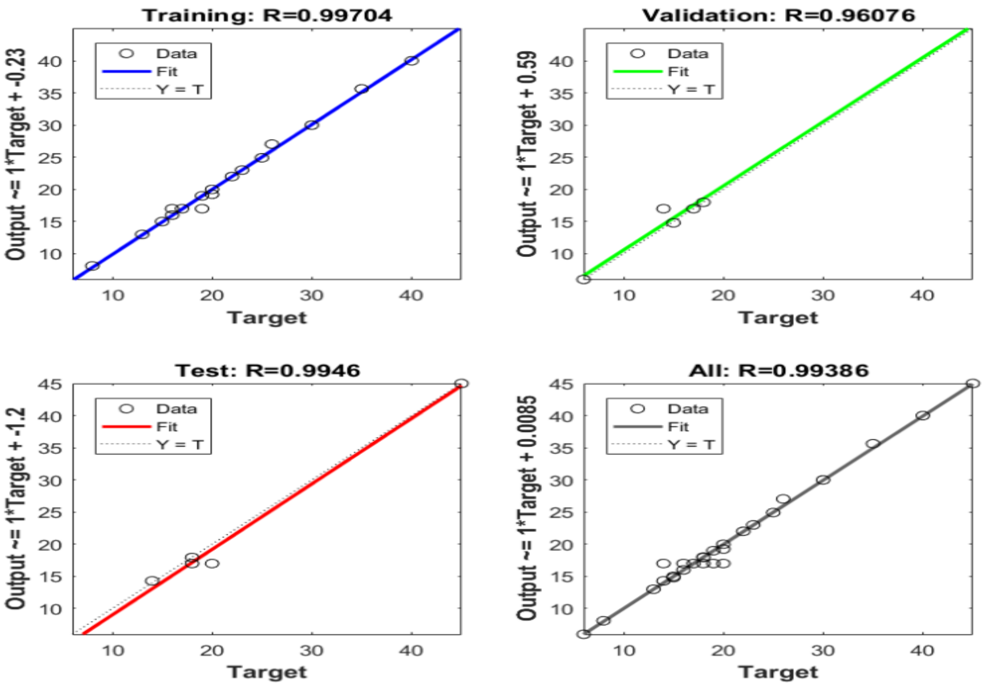

S15: Regression of Training, Verification, Evaluation data sets for OR.

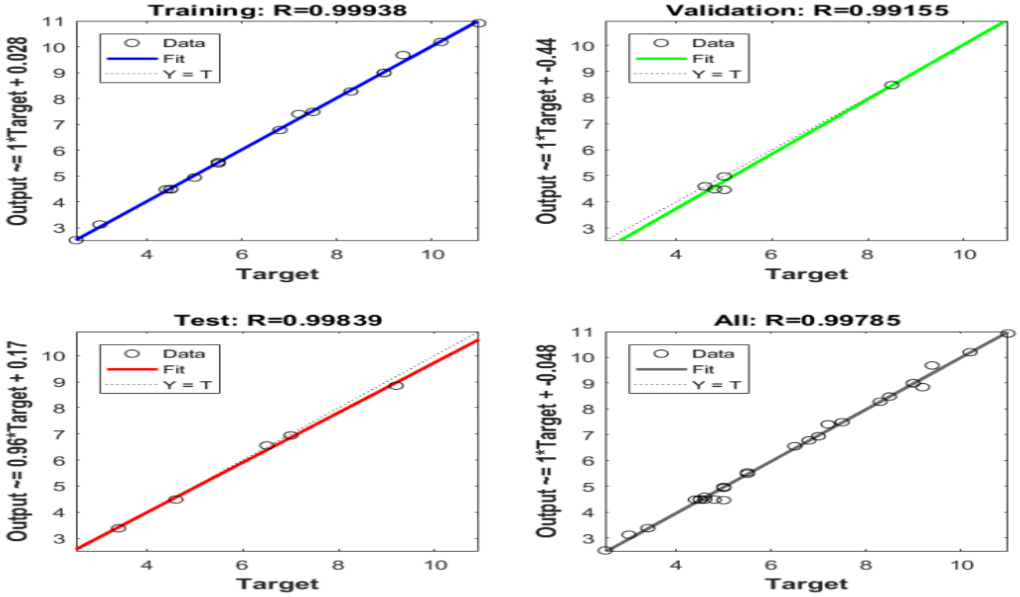

S16: Regression of Training, Verification, Evaluation data sets for Ra.

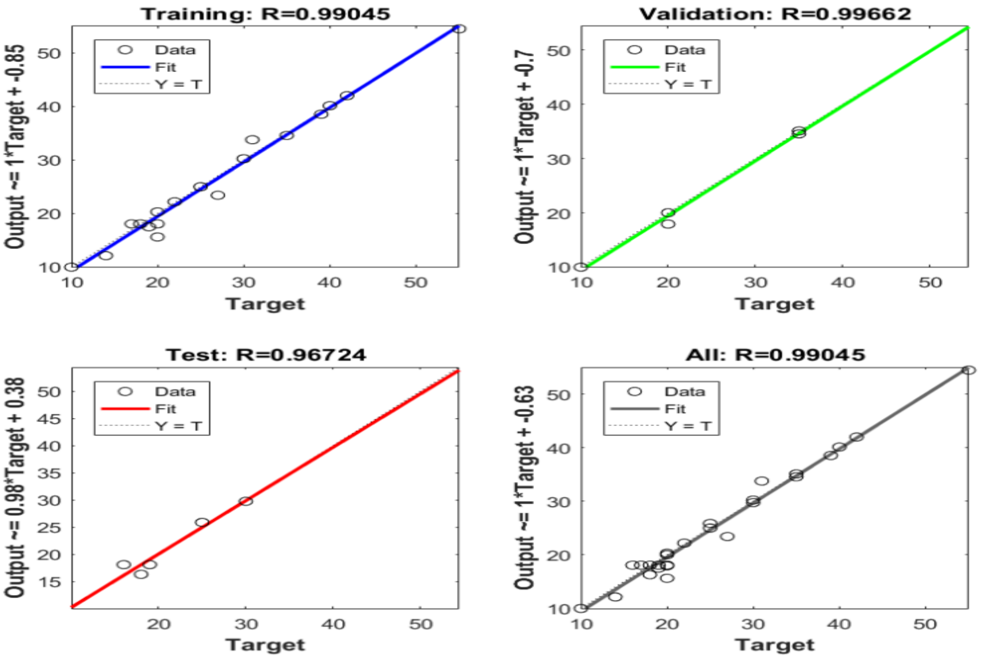

S17: Regression of Training, Verification, Evaluation data sets for $R_{max.}$

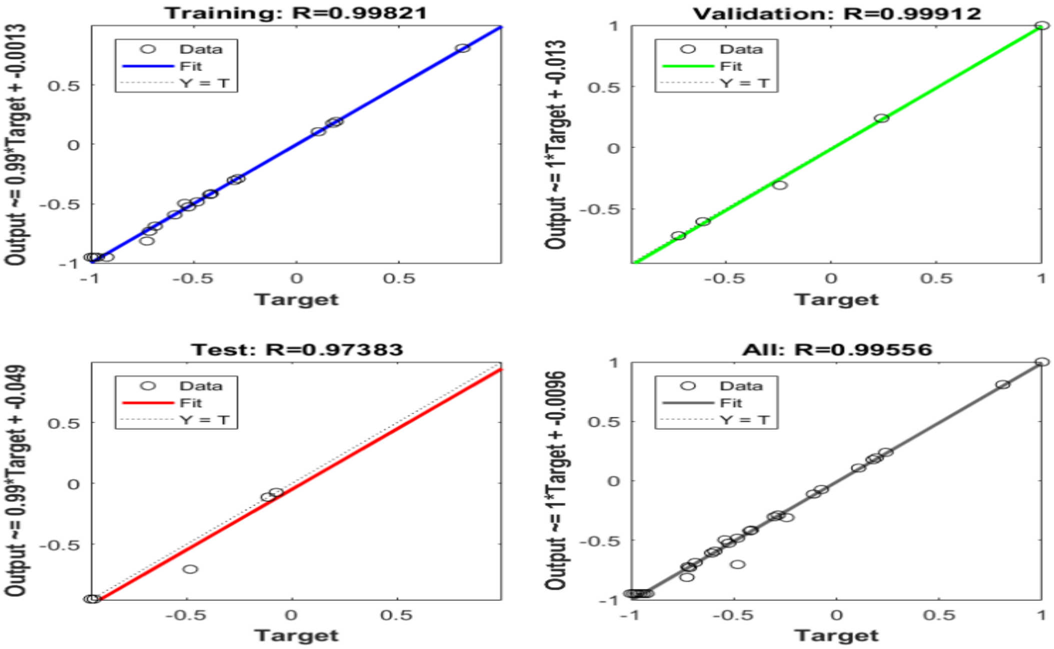

S18: Regression of Training, Verification, Evaluation data sets for TWR.

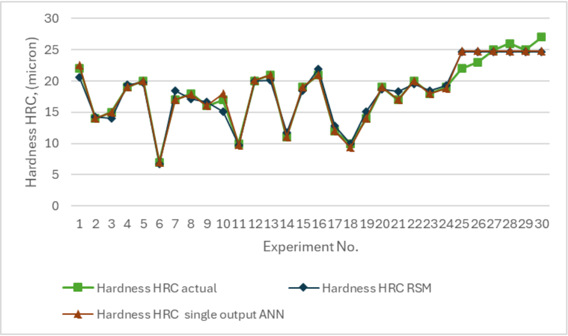

(a)

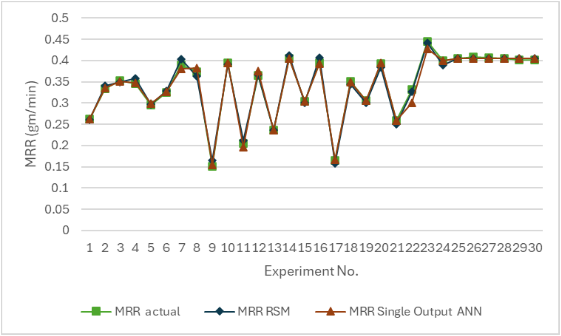

(b)

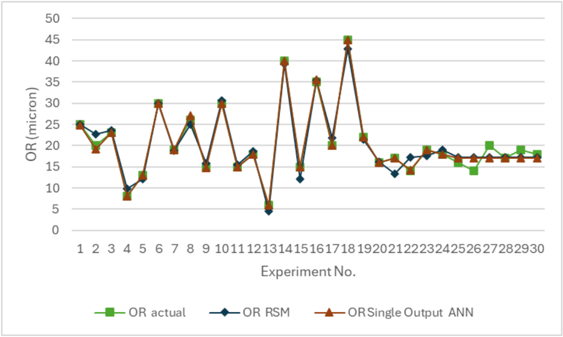

(c)

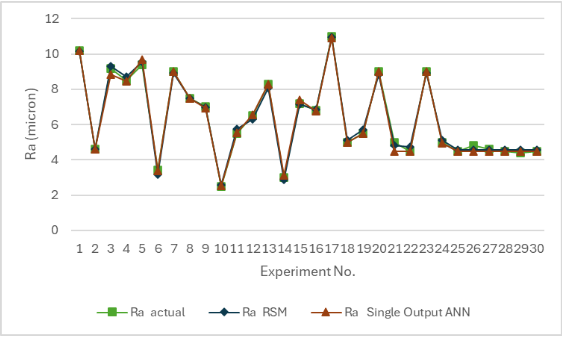

(d)

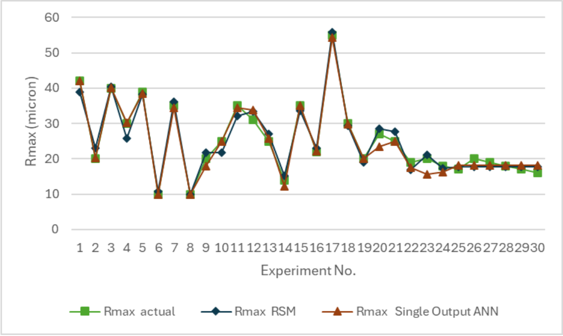

(e)

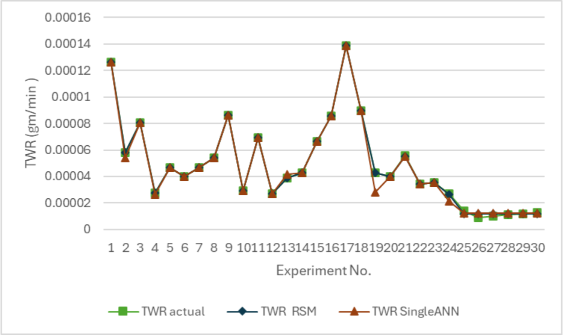

(f)

S19: Actual magnitudes Vs Predicted magnitudes by RSM and ANN for (a) H (b) MRR(c) OR (d) Ra (e) R_{max} (f) TWR.

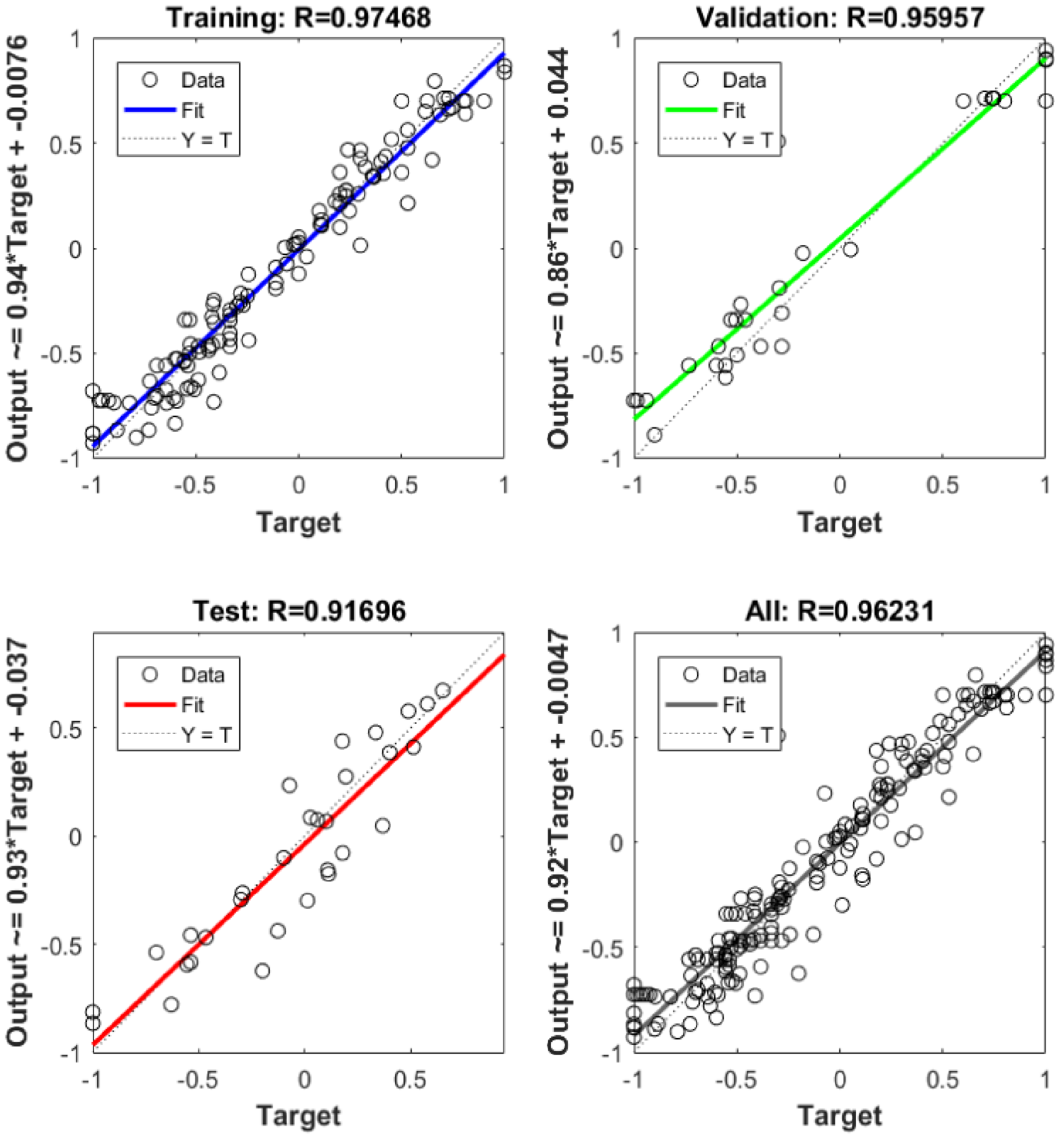

S20: Regression of Training, Validation, Testing Data Sets for Multi-Output ANN

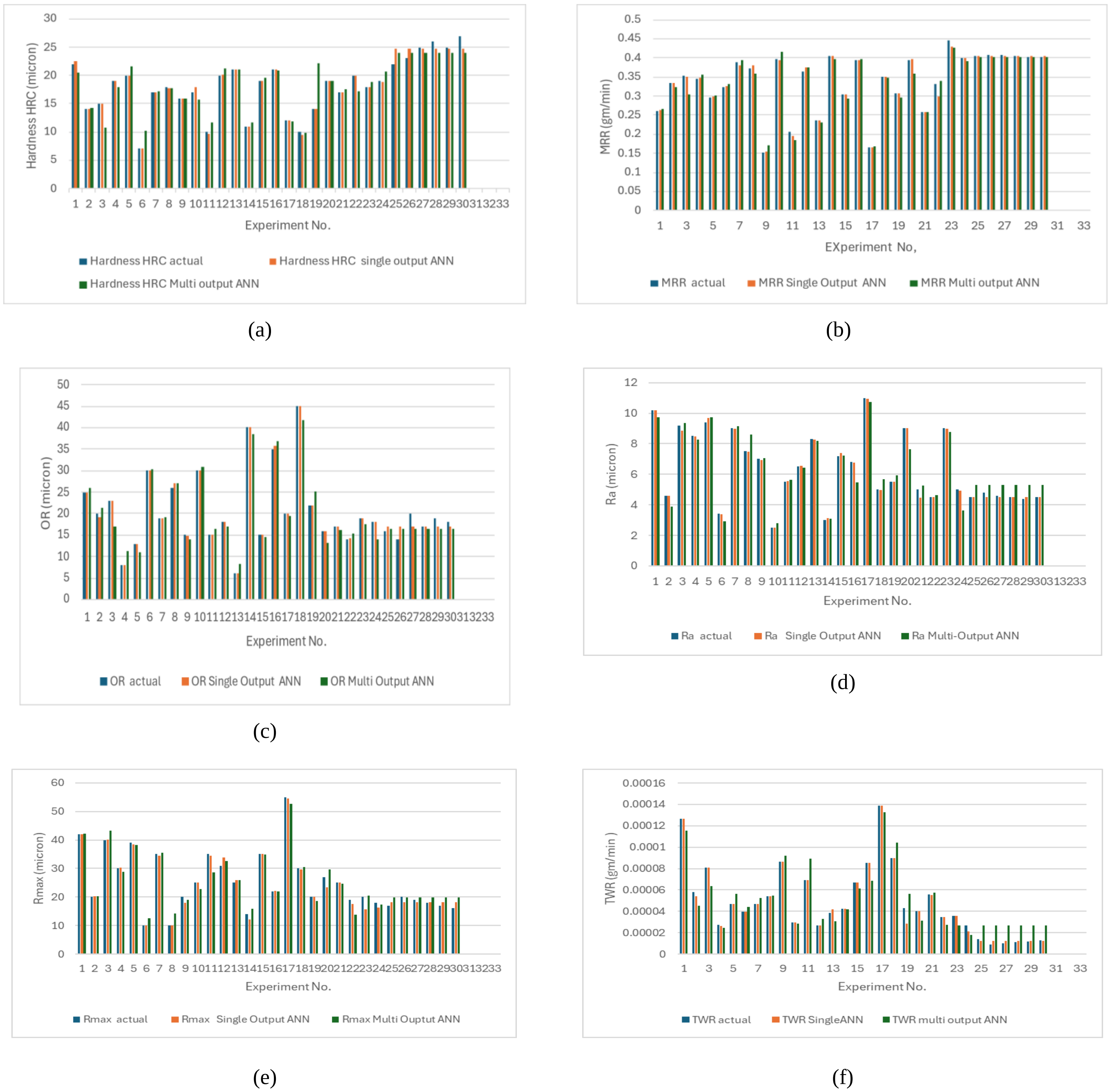

S21: Actual Values Vs Predicted Values Using Single Output ANN and Multi Output ANN (a) H (b) MRR(c) OR (d) Ra (e) R_{max} (f) TWR.

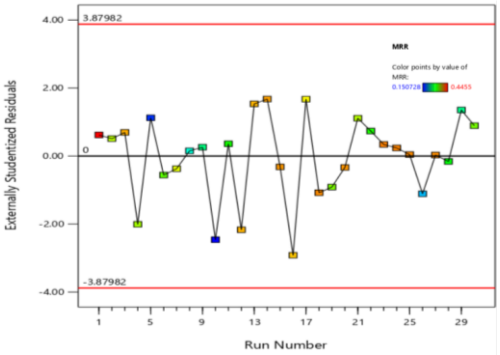

a) MRR

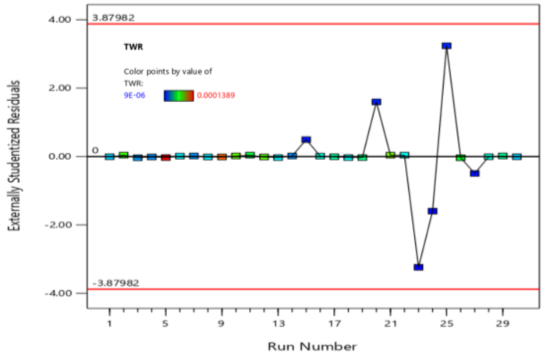

b) TWR

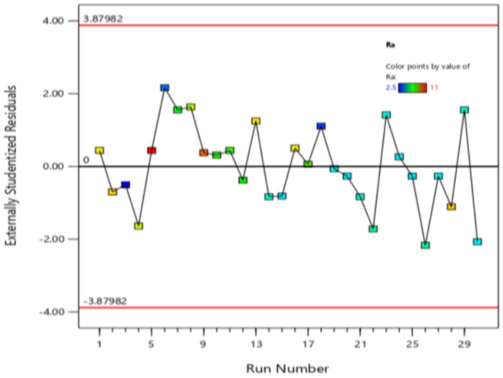

c) Ra

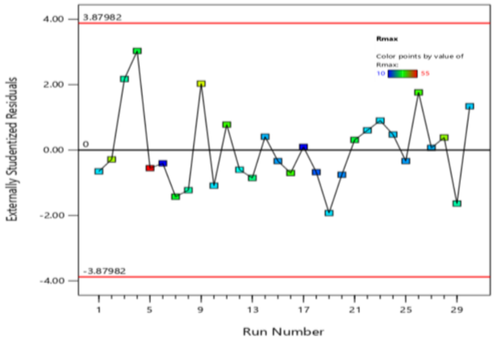

d) R_{max}

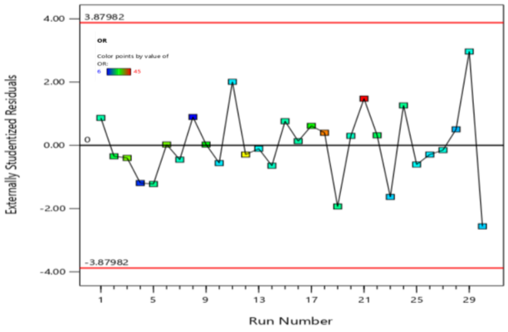

e) OR

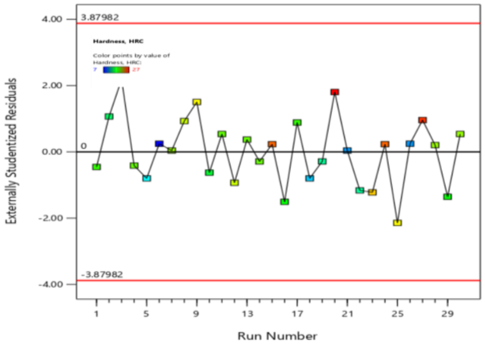

f) H

S22: (Studentized residuals versus experimental run order) values of (MRR, TWR, R_a, R_{max}, OR and H) for (CK45).

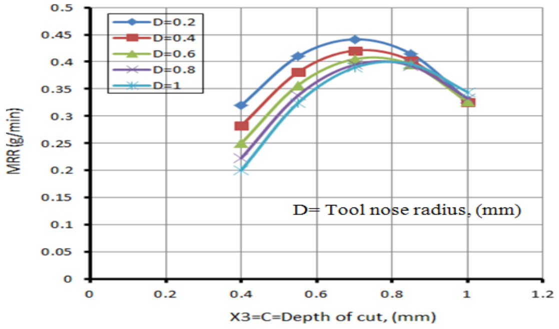

a) Effect of D on MRR at different levels of R

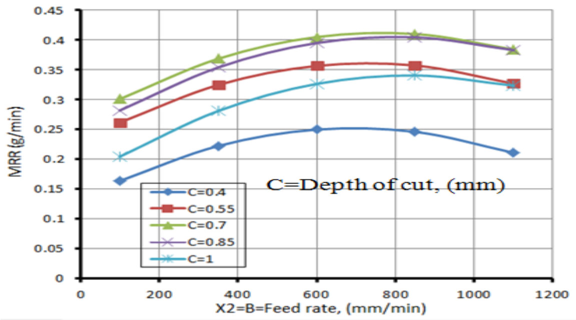

b) Effect of F on MRR at different levels of D

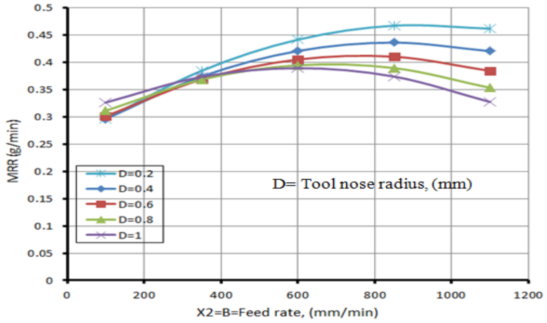

c) Effect of F on MRR at different levels of R

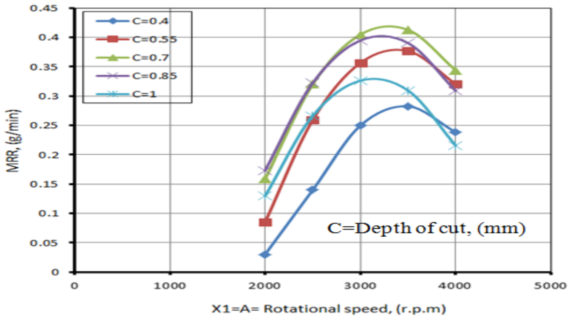

d) Effect of N on MRR at different levels of D

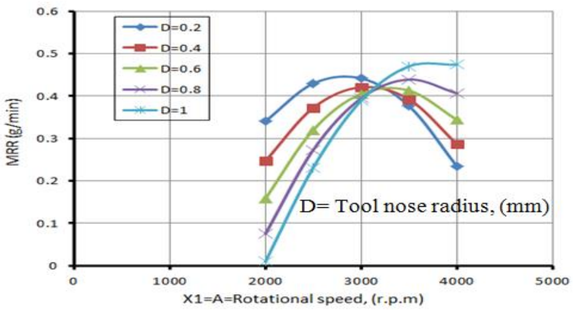

e) Effect of N on MRR at different levels of R

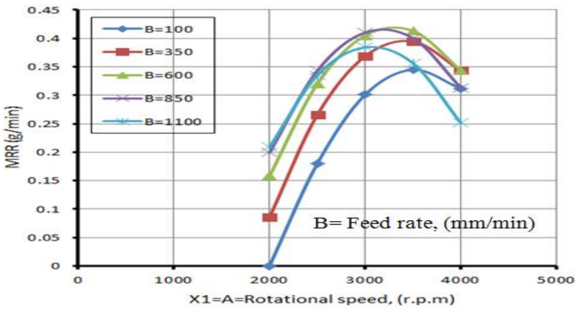

f) Effect of N on MRR at different levels of F

S23: Influence of the different parameters on MRR.

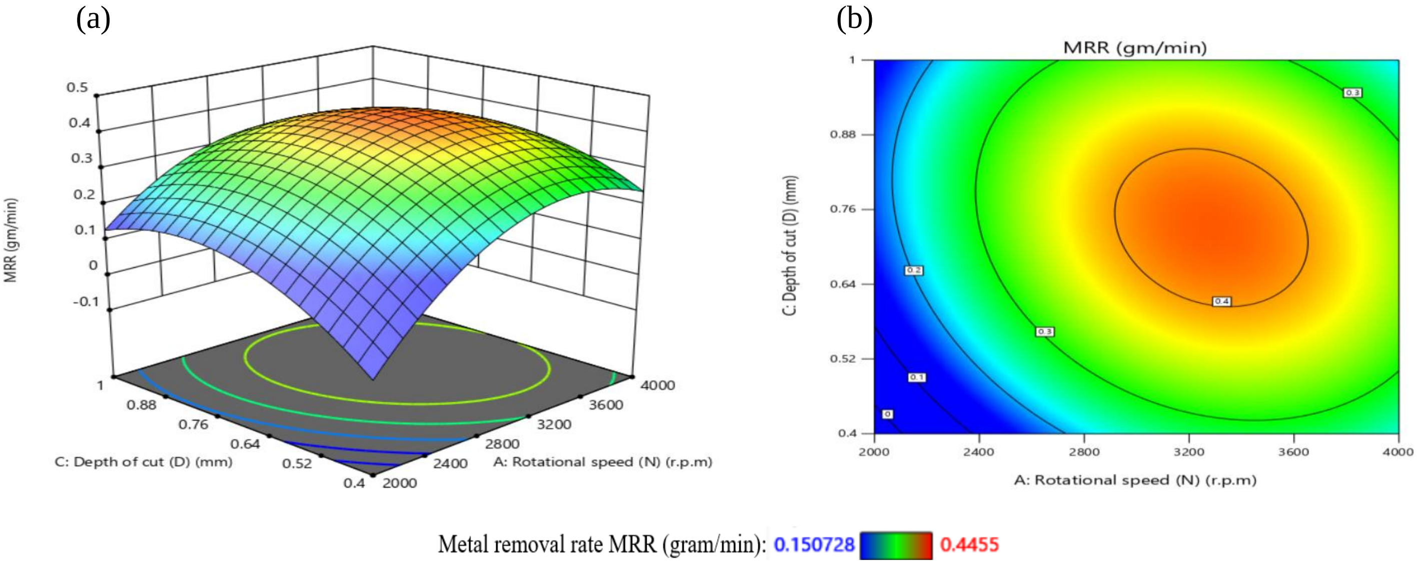

S24: Interaction of N and D on MRR: (a) 3D surface; (b) contour plot

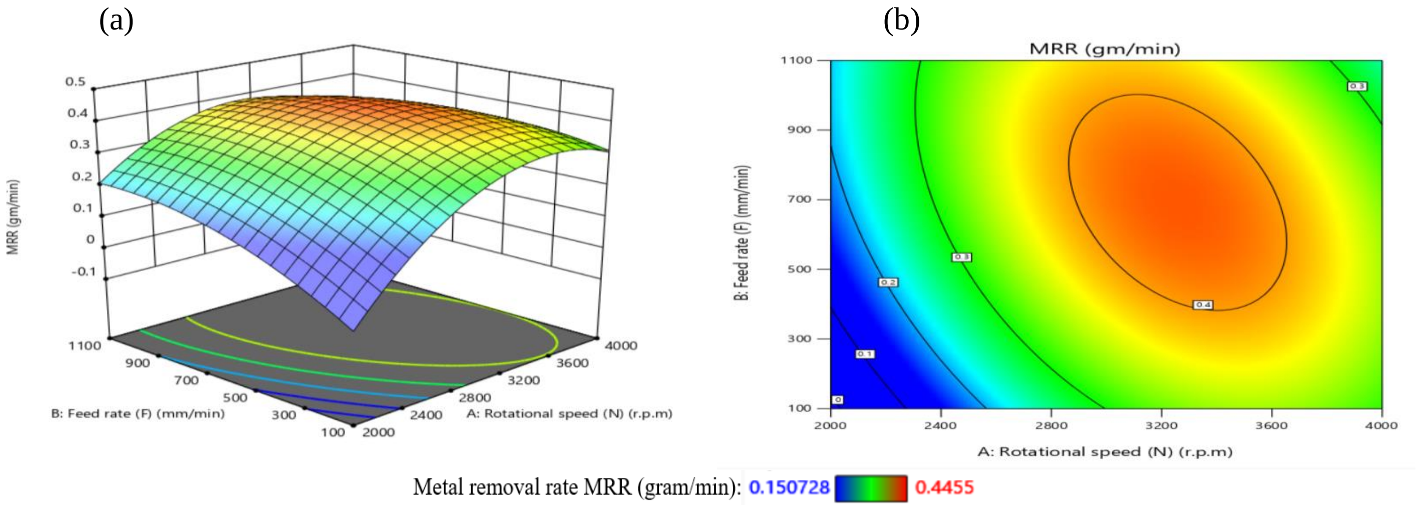

S25: Interaction of N and F on MRR: (a) 3D surface; (b) contour plot.

(a) (b)

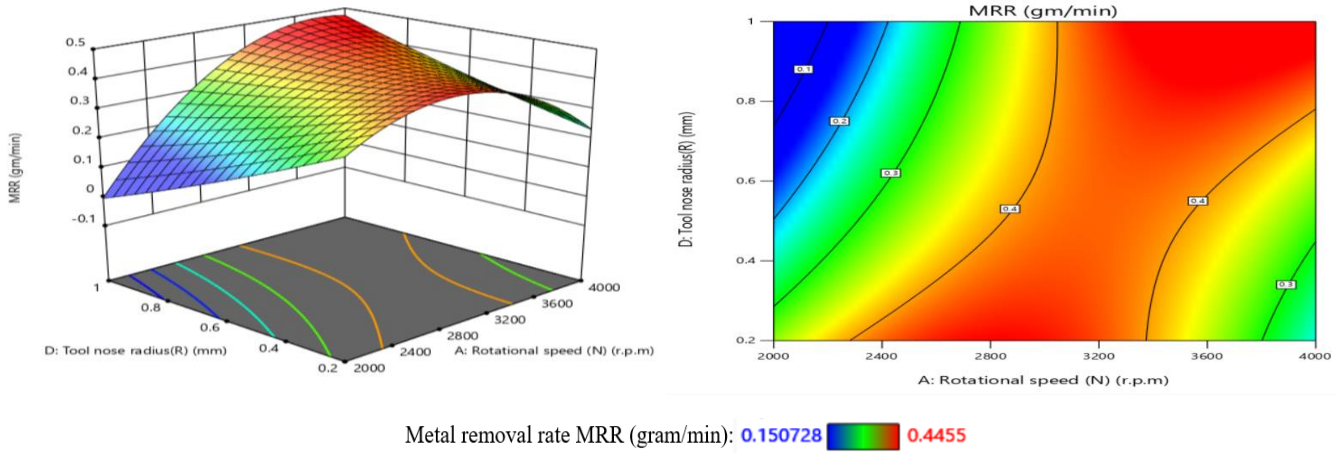

S26: Interaction of N and R on MRR: (a) 3D surface; (b) contour plot.

(a) (b)

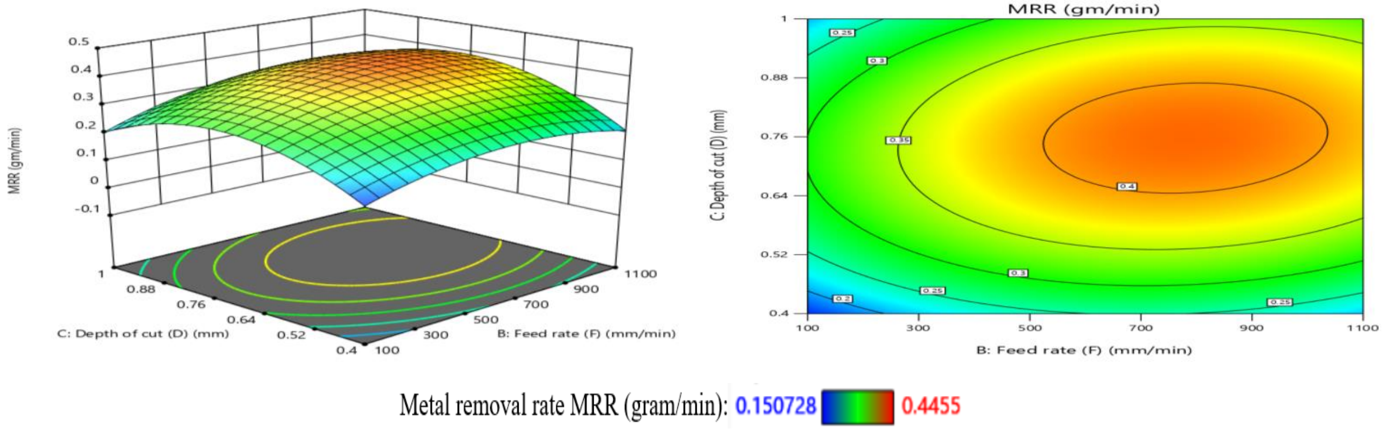

S27: Interaction of F and D on MRR: (a) 3D surface; (b) contour plot.

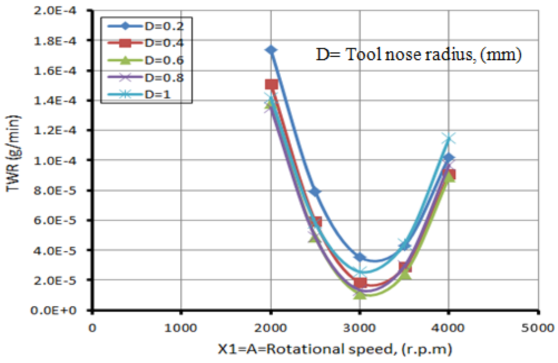

a) Behavior of N on TWR at different values of R

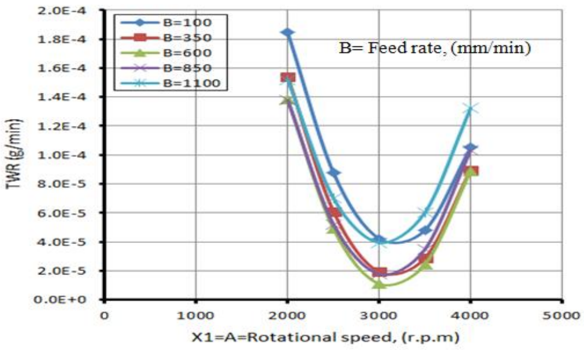

b) Behavior of N on TWR at different values of F

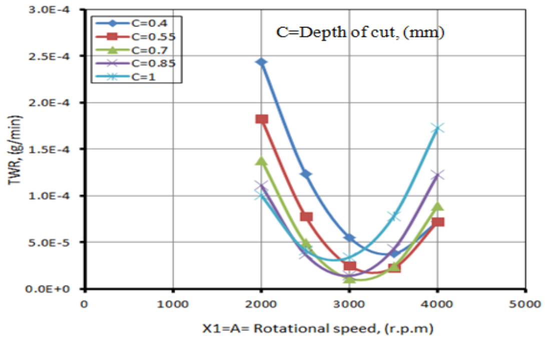

c) Behavior of N on TWR at different values of D

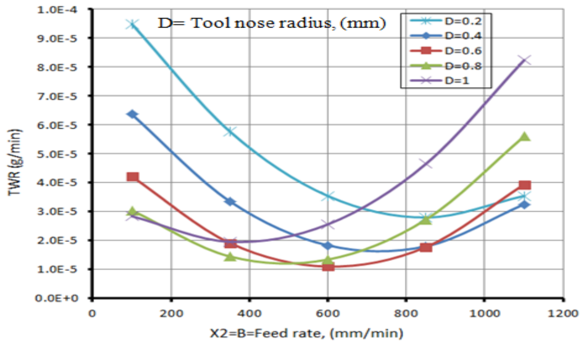

d) Behavior of F on TWR at different values of R

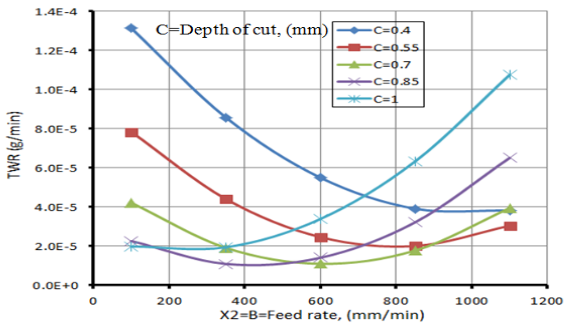

e) Behavior of F on TWR at different values of D

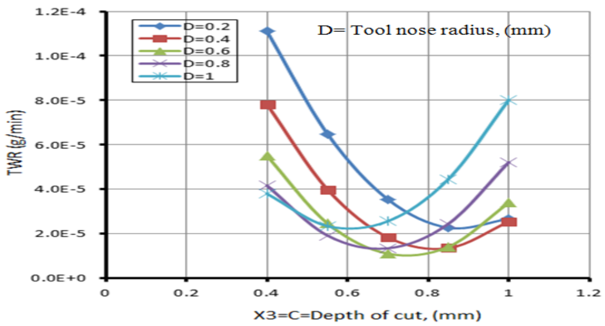

f) Behavior of D on TWR at different values of R

S28: Influence of the various parameters on TWR.

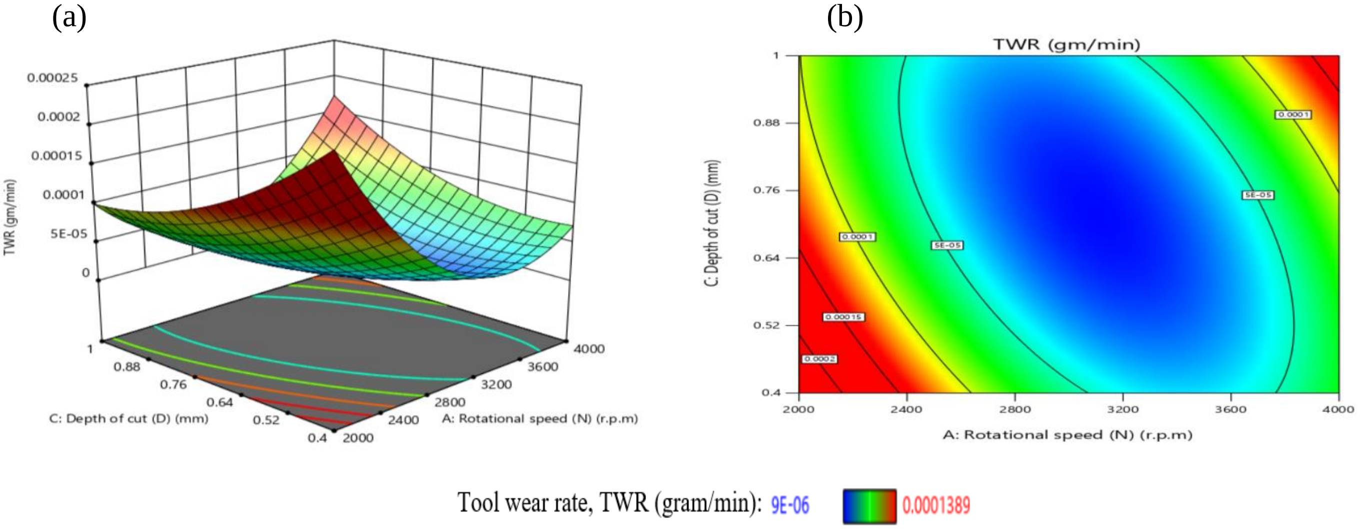

S29: Interaction of N and D on TWR: (a) 3D surface; (b) contour plot.

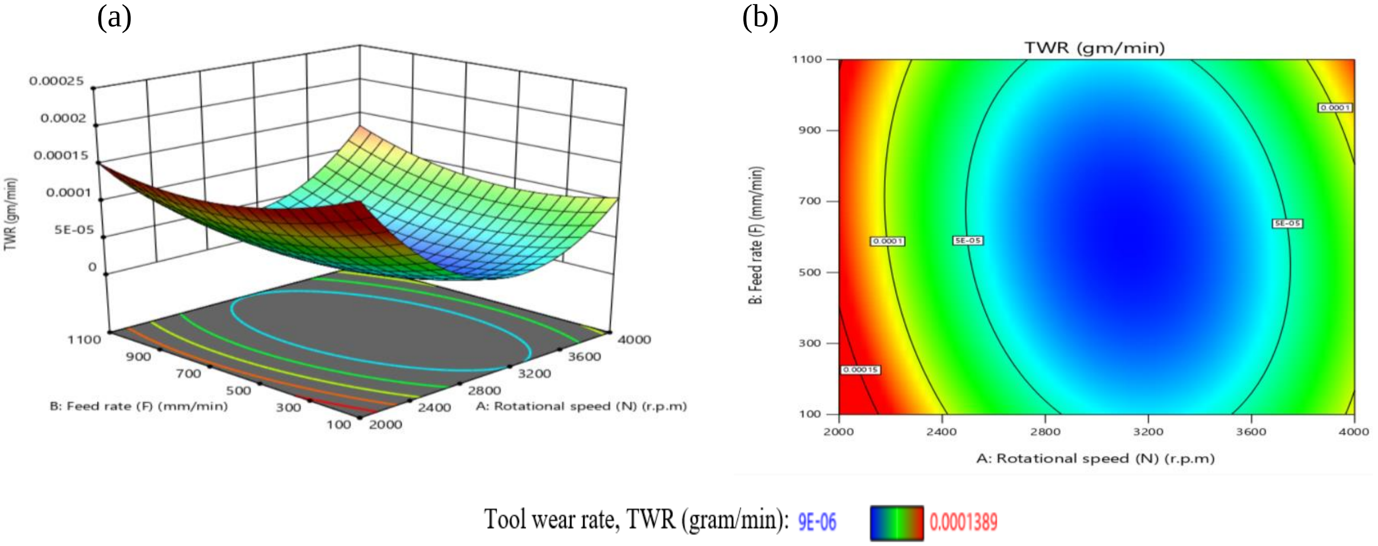

S30: Interaction of N and F on TWR: (a) 3D surface; (b) contour plot.

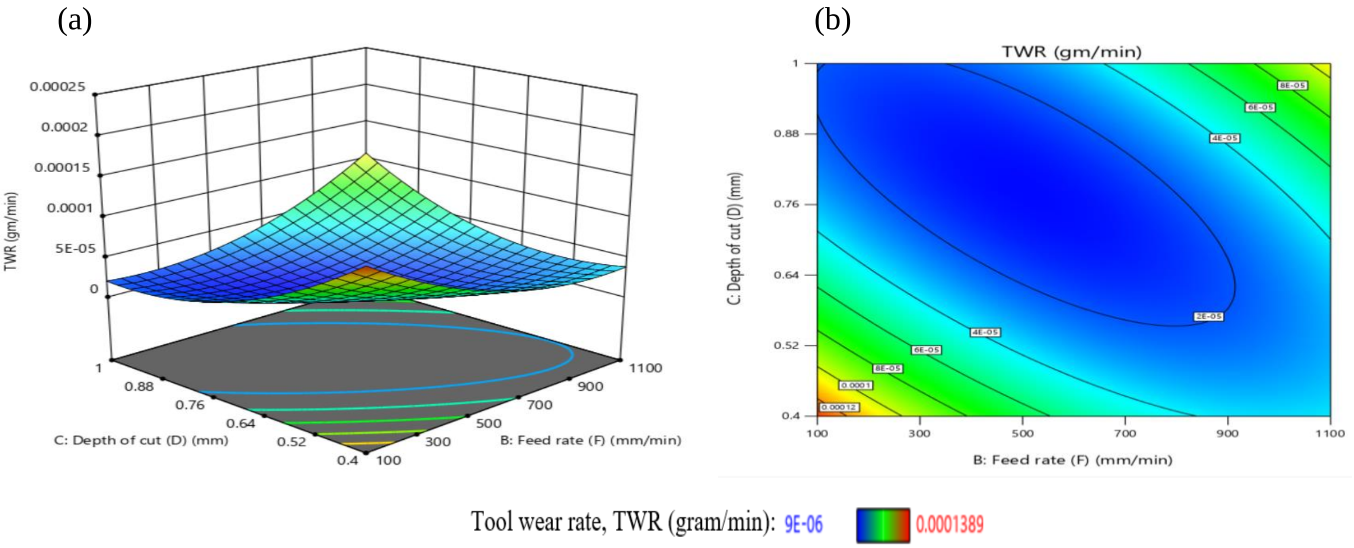

S31: Interaction of F and D on TWR: (a) 3D surface; (b) contour plot.

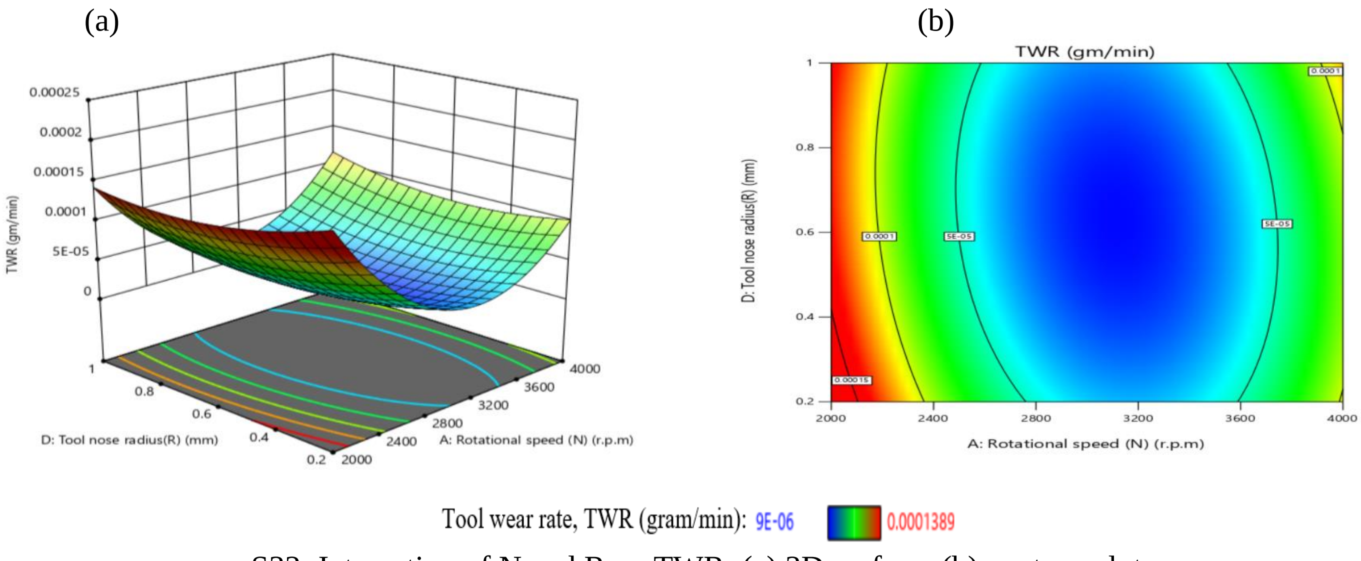

S32: Interaction of N and R on TWR: (a) 3D surface; (b) contour plot.

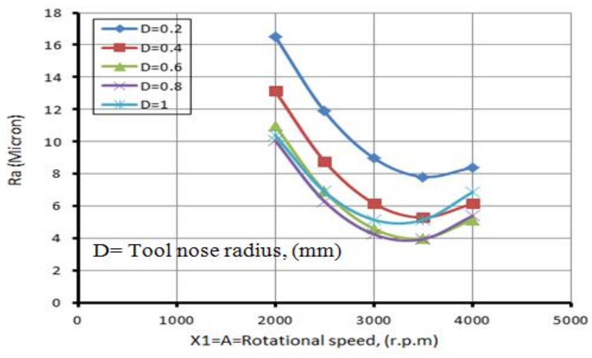

a) Behavior of N on R_a at various levels of R

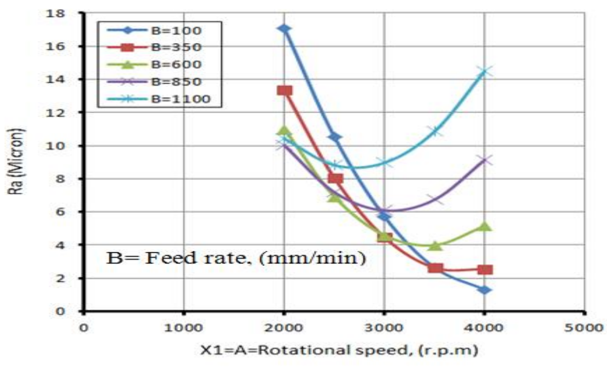

b) Behavior of N on R_a at various levels of F

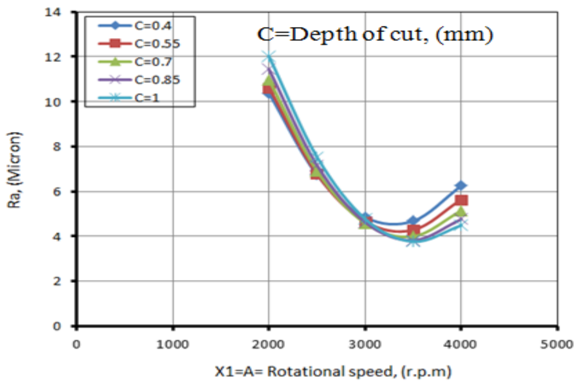

c) Behavior of N on R_a at various levels of D

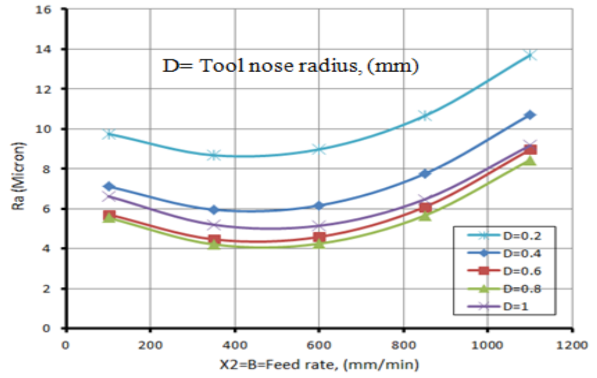

d) Behavior of F on R_a at various levels of R

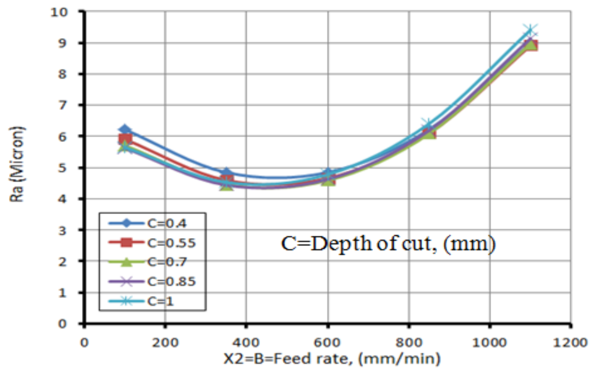

e) Behavior of F on R_a at various levels of D

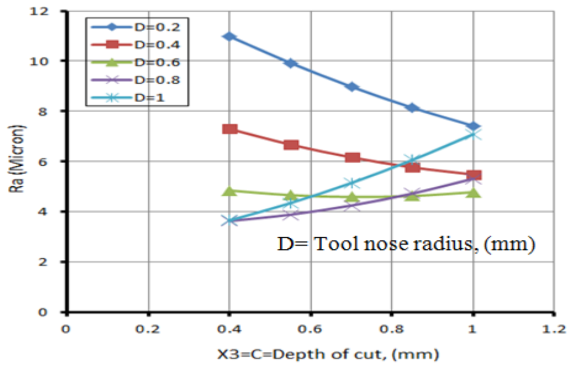

f) Behavior of D on R_a at various levels of R

S33: Influence of the various parameters on Ra.

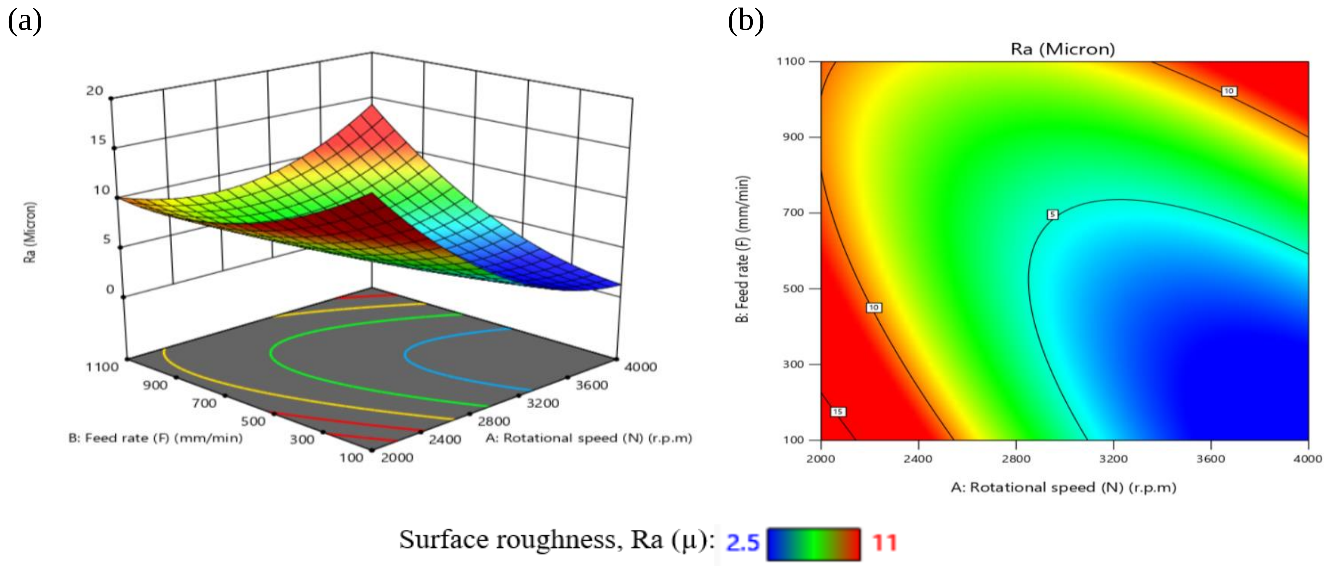

S34: Interaction of N and F on R_a: (a) 3D surface; (b) contour plot.

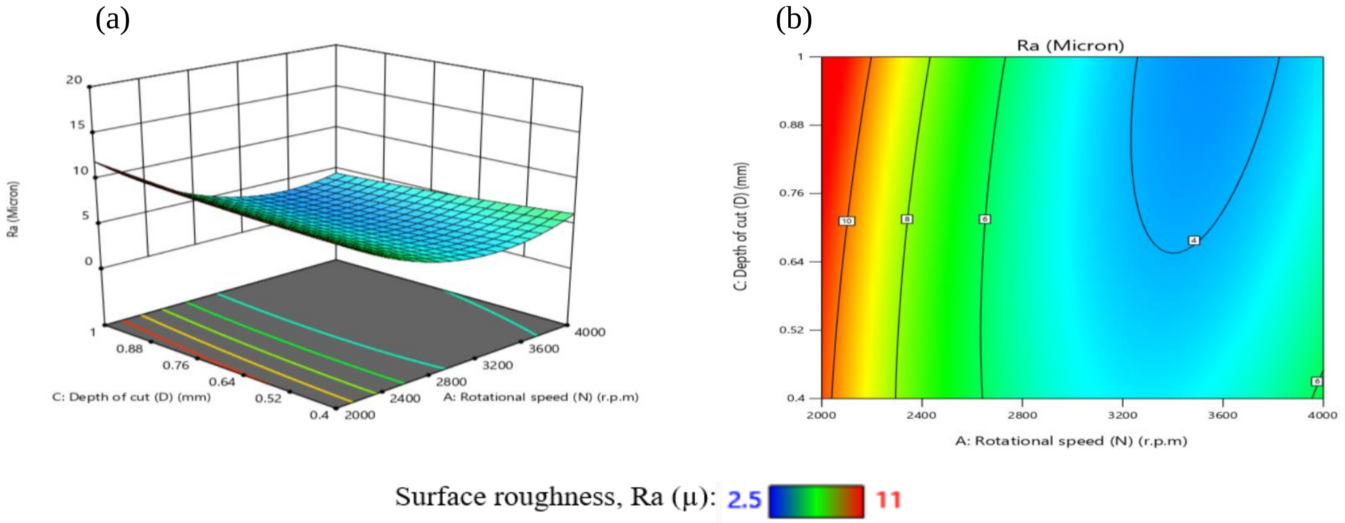

S35: Interaction of N and D on R_a: (a) 3D surface; (b) contour plot.

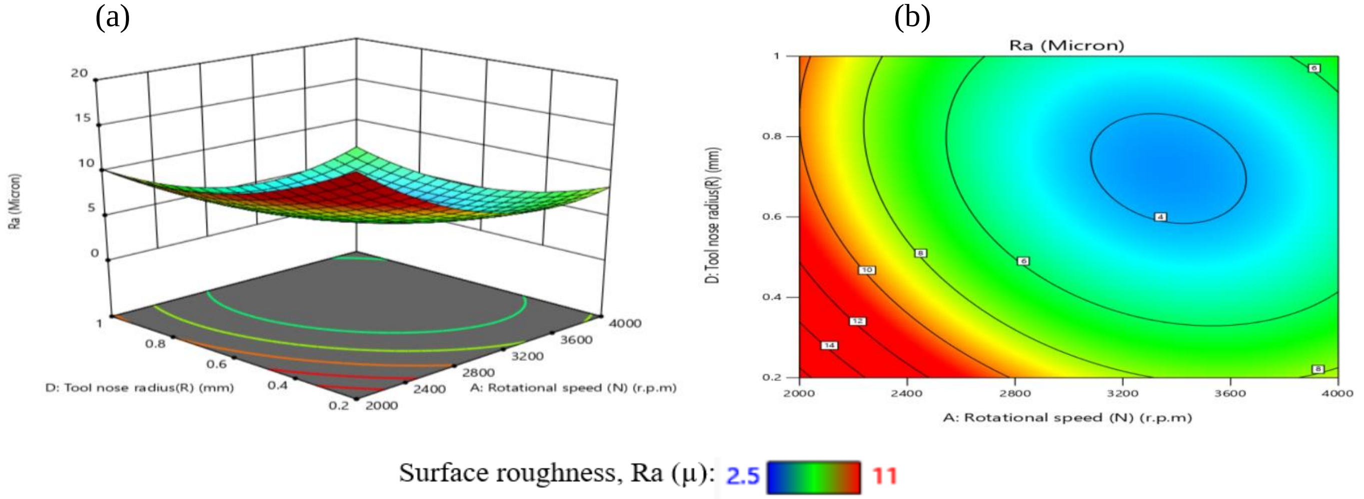

S36: Interaction of N and R on R_a: (a) 3D surface; (b) contour plot.

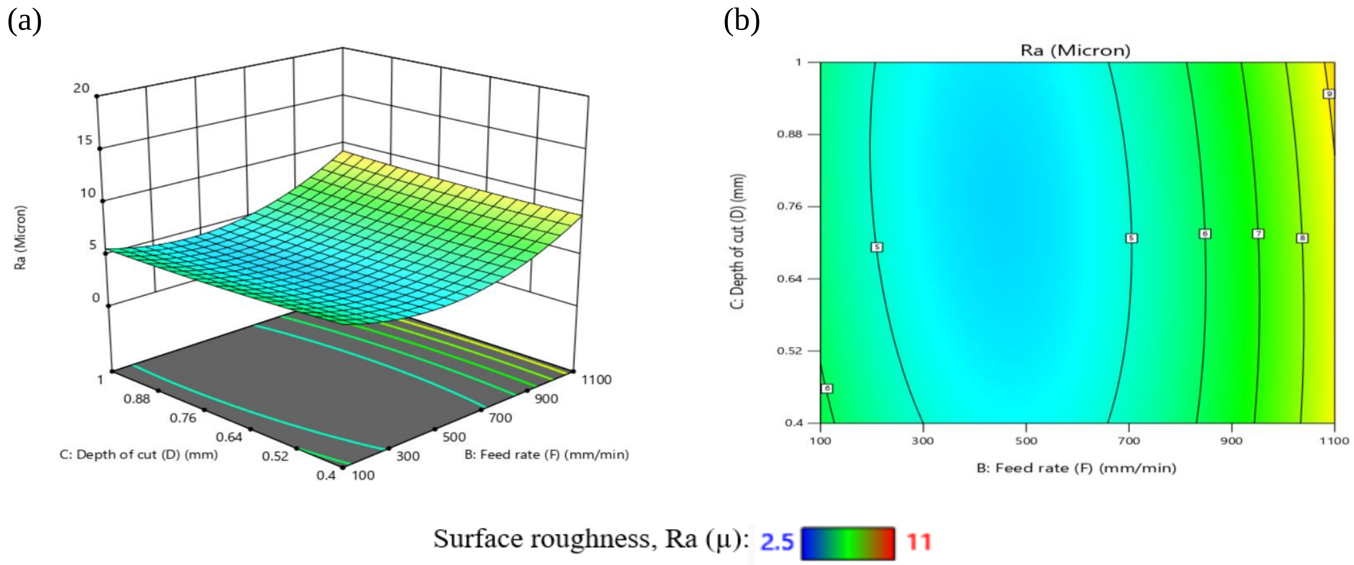

S37: Interaction of F and D on R_a: (a) 3D surface; (b) contour plot

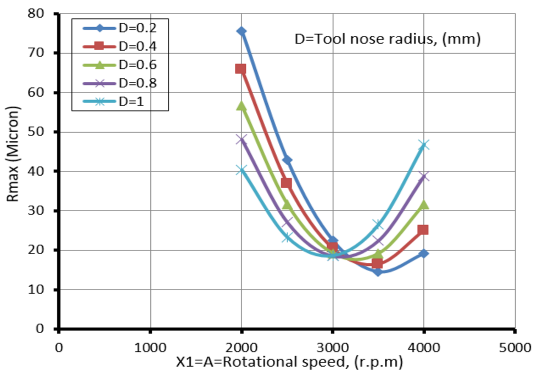

a) Behavior of N on R_{max} at various levels of R

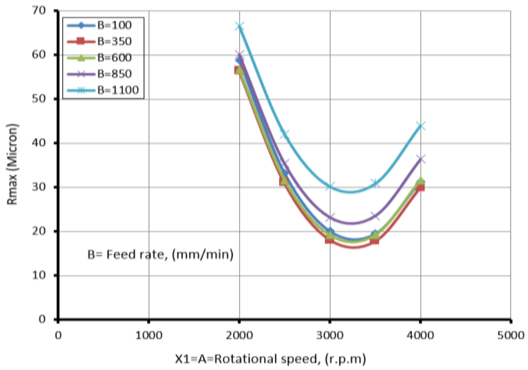

b) Behavior of N on R_{max} at various levels of F

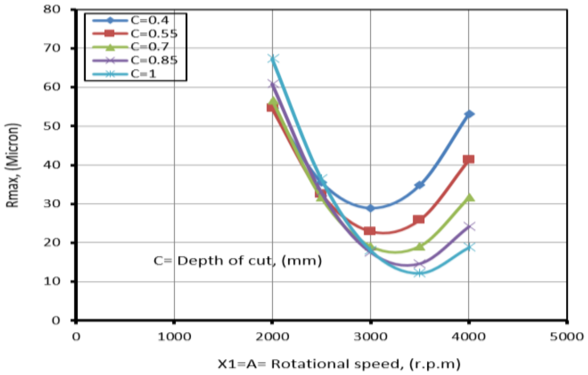

c) Behavior of N on R_{max} at various levels of D

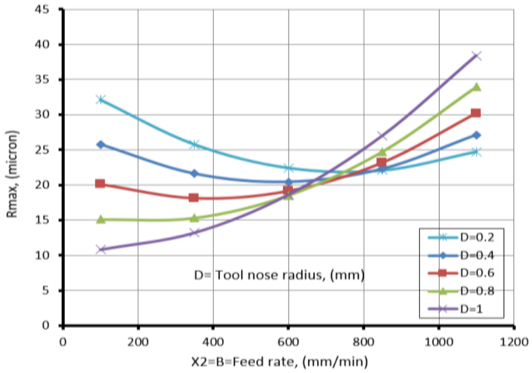

d) Behavior of F on R_{max} at various t levels of R

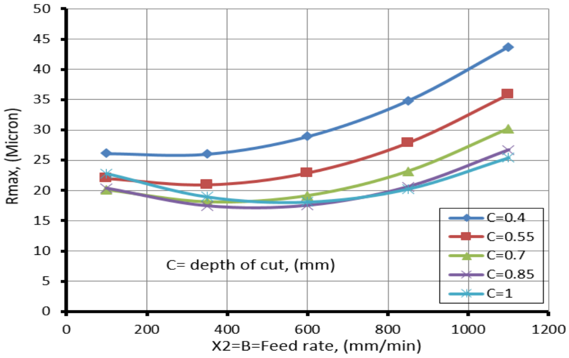

e) Behavior of F on R_{max} at various levels of D

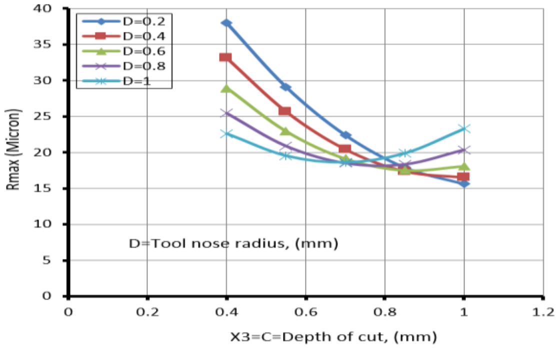

f) Behavior of D on R_{max} at various levels of R

S38: Influence of the various parameters on R_{max}.

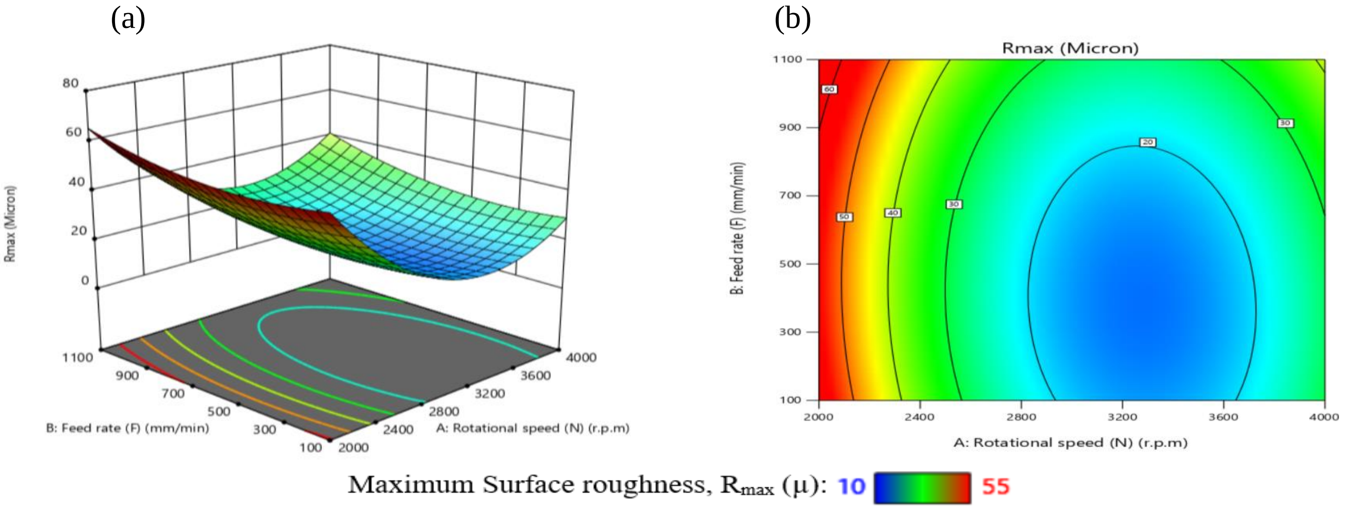

S39: Interaction of N and F on R_{max}: (a) 3D surface; (b) contour plot.

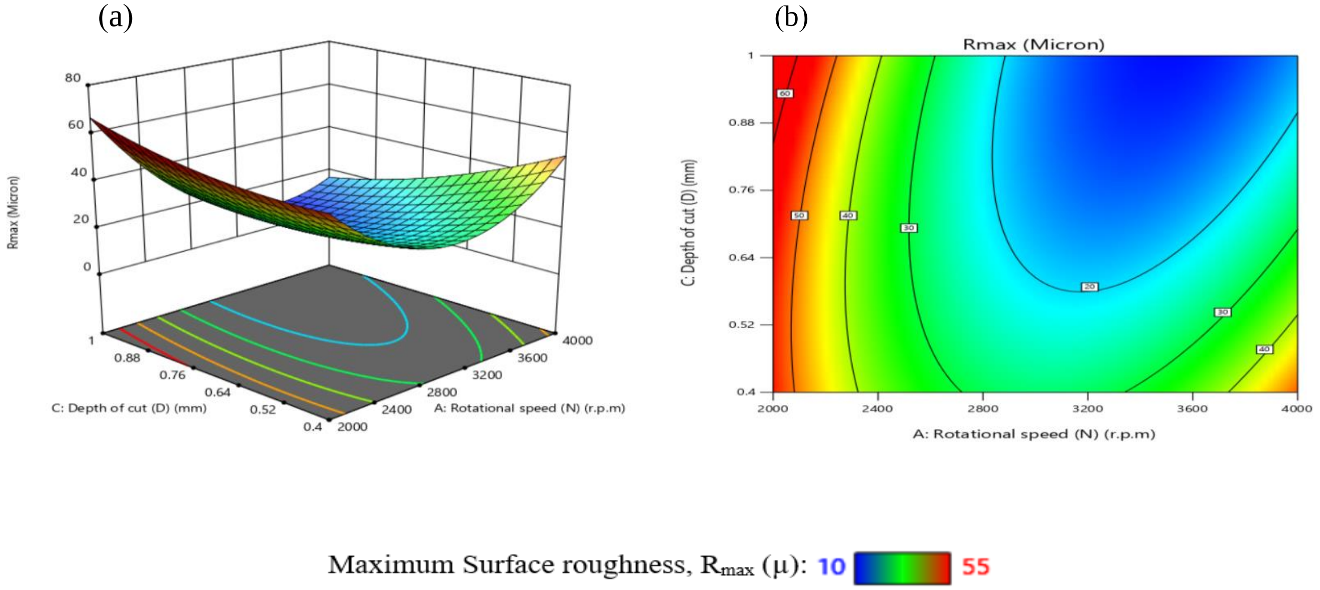

S40: Interaction of N and D on R_{max}: (a) 3D surface; (b) contour plot

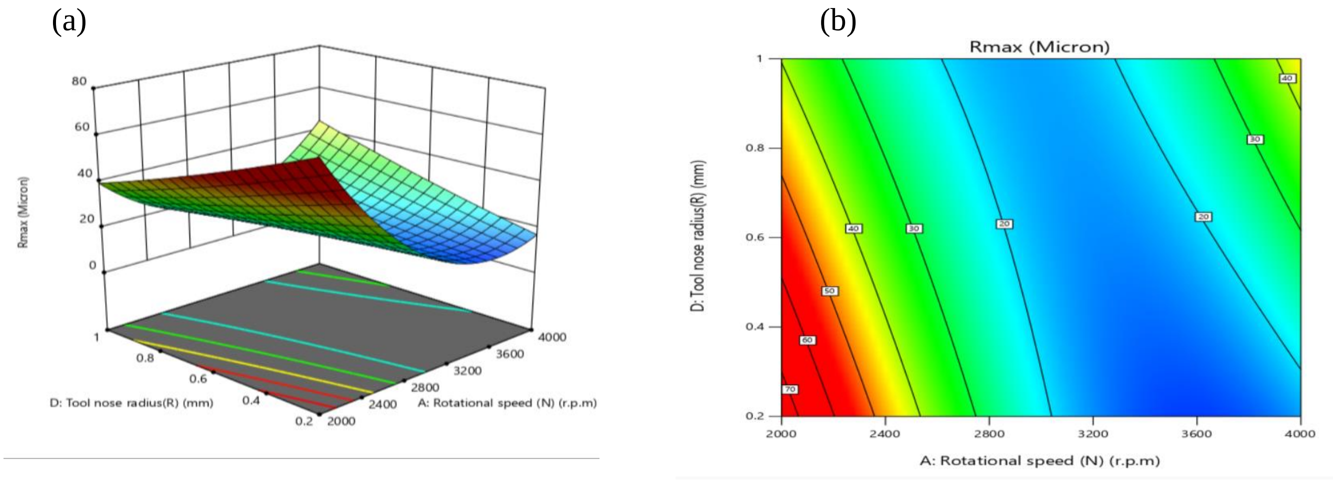

Maximum Surface roughness, R_{max} (μ): 10 55

S41: Interaction of N and R on R_{max}: (a) 3D surface; (b) contour plot.

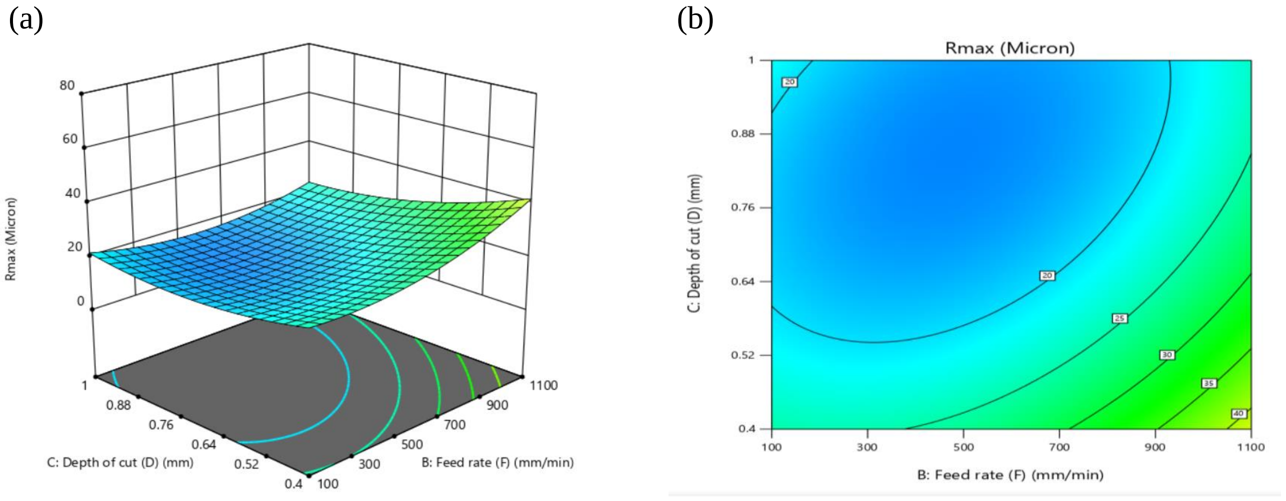

Maximum Surface roughness, R_{max} (μ): 10 55

S42: Interaction of F and D on R_{max}: (a) 3D surface; (b) contour plot.

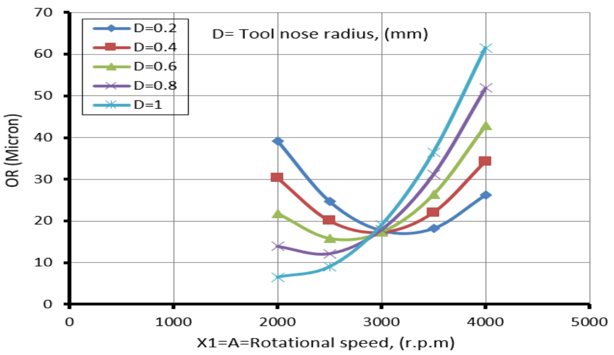

a) Behavior of N on OR at various levels of R

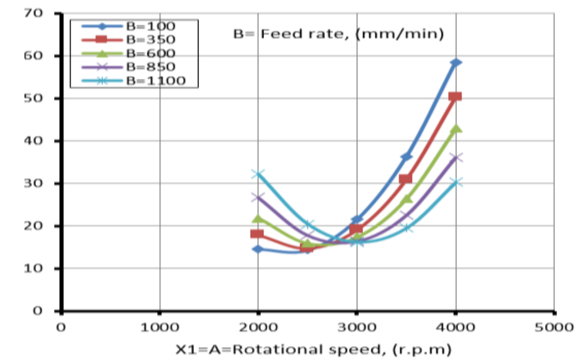

b) Behavior of N on OR at various levels of F

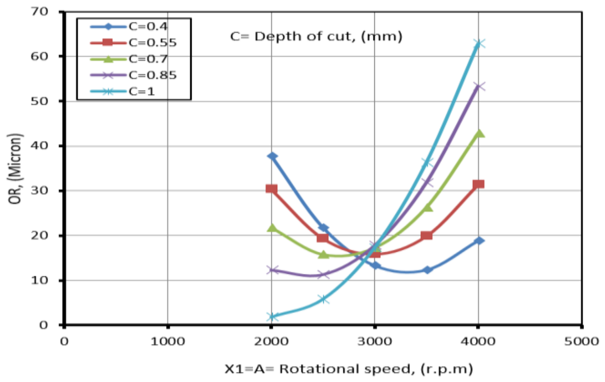

c) Behavior of N on OR at various levels of D

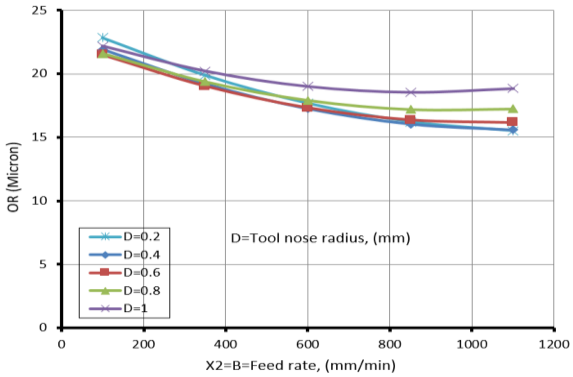

e) Behavior of F on OR at various levels of R

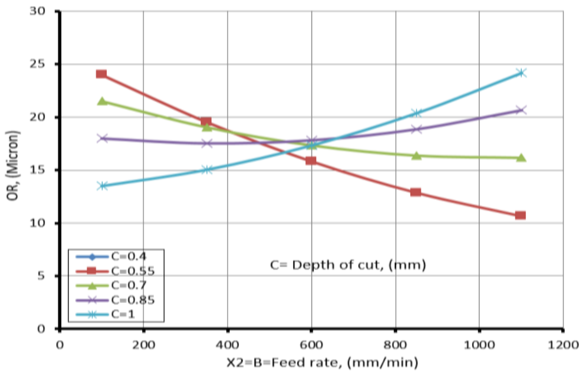

e) Behavior of F on OR at various levels of D

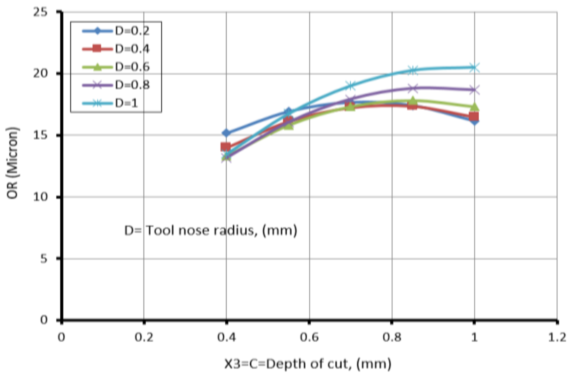

f) Behavior of D on OR at various levels of R

S43: Influence of the various parameters on OR.

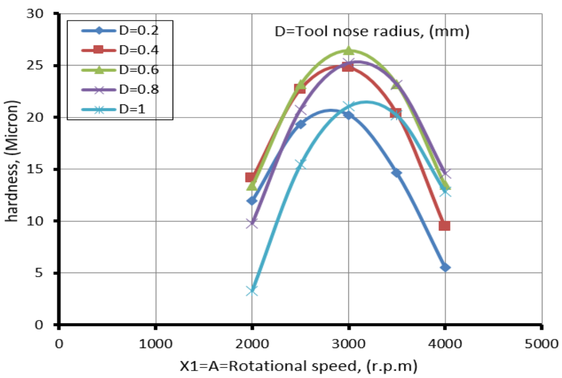

a) Behavior of N on H at various levels of R

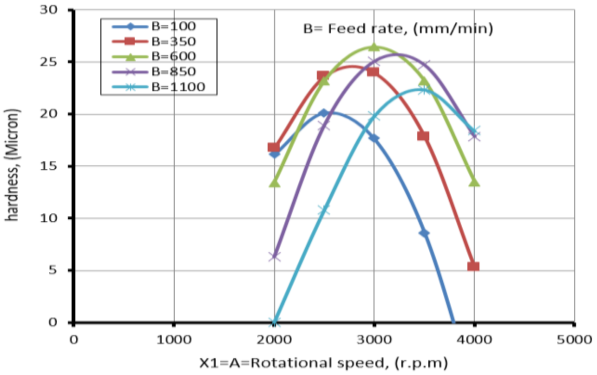

b) Behavior of N on H at various levels of F

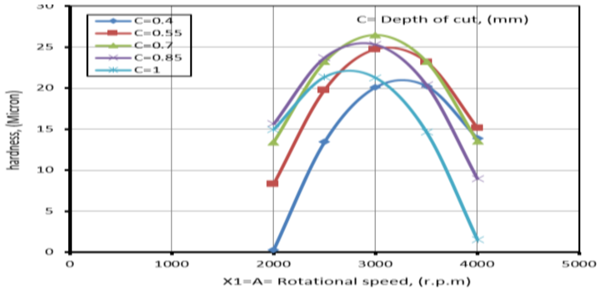

c) Behavior of N on H at various levels of D

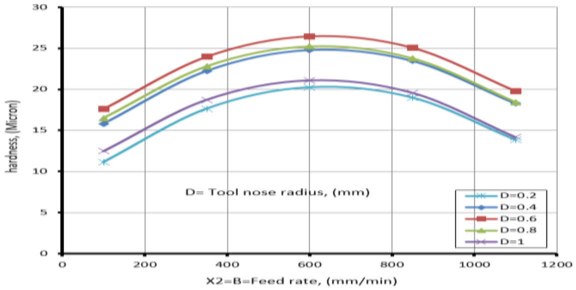

d) Behavior of F on H at various levels of R

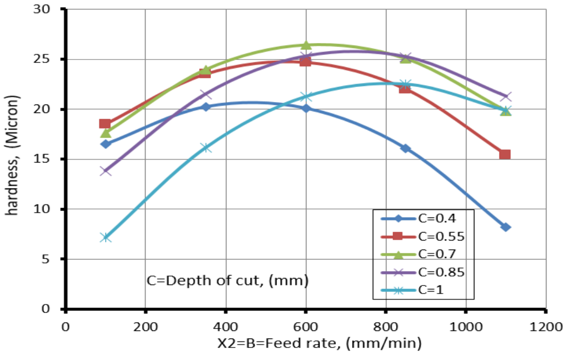

e) Behavior of F on H at various levels of D

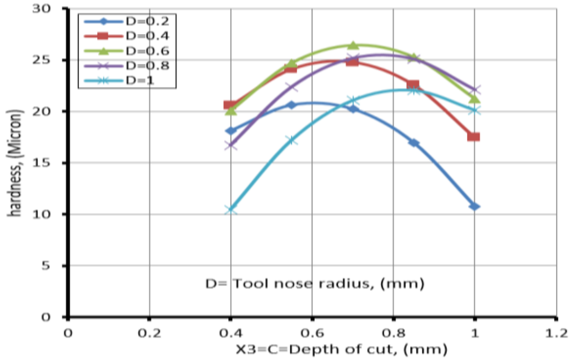

f) Behavior of D on H at various levels of R

S44: Influence of various parameters on H.

(a) (b)

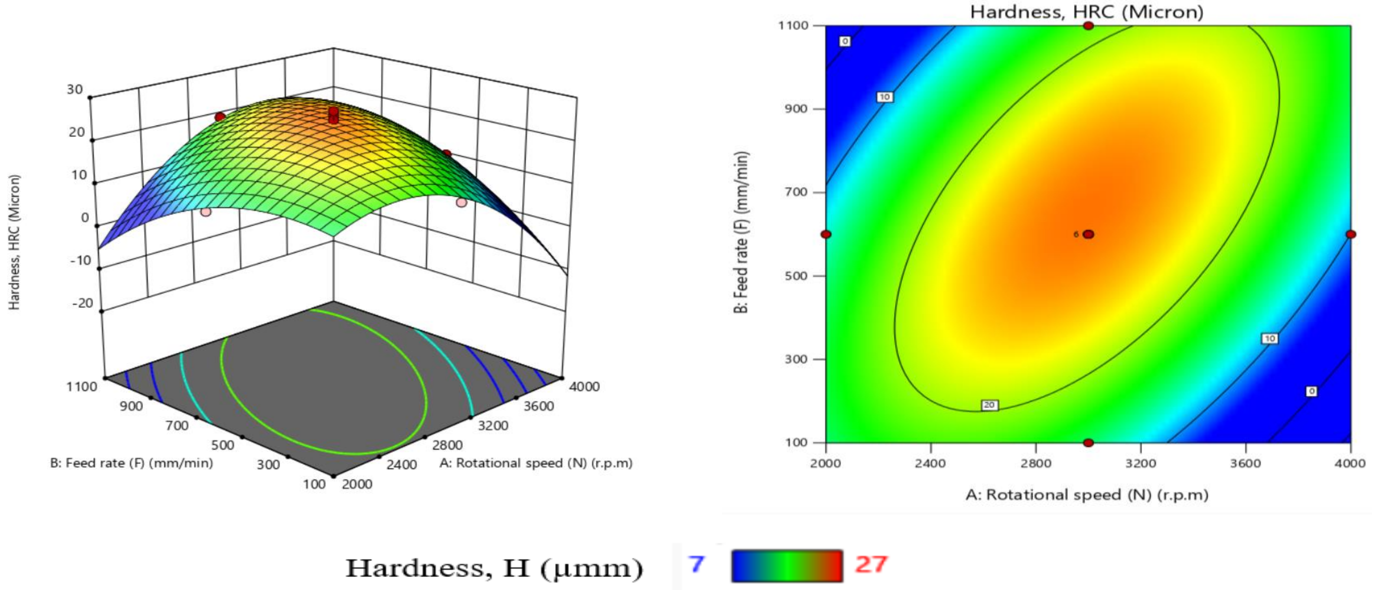

S45: Interaction of N and F on H: (a) 3D surface; (b) contour plot.

(a) (b)

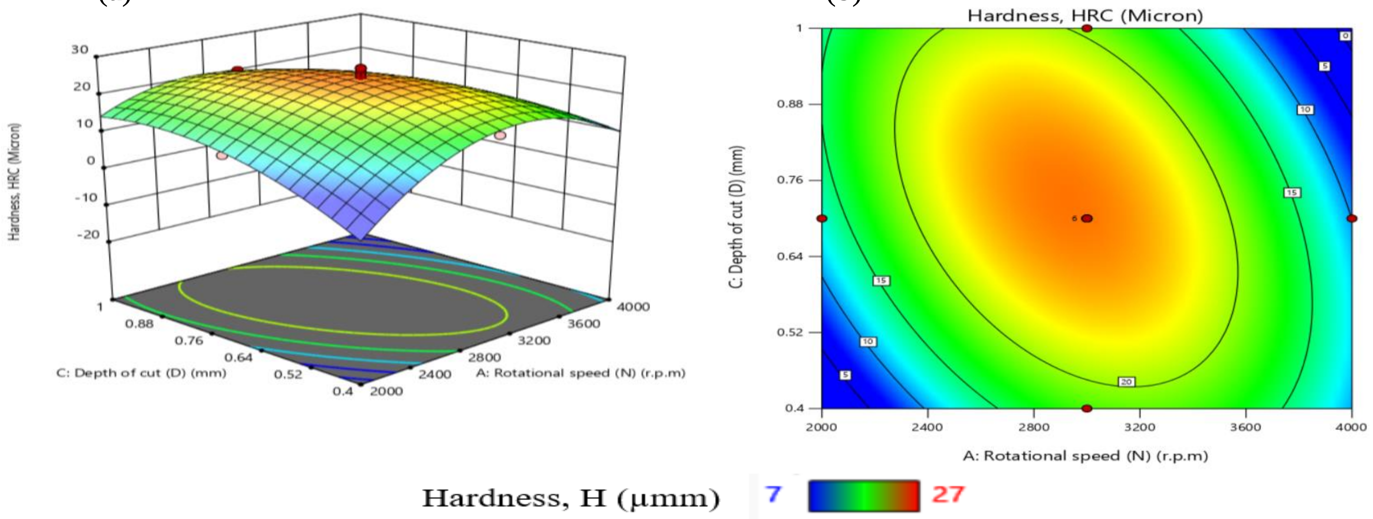

S46: Interaction of N and D on H: (a) 3D surface; (b) contour plot.

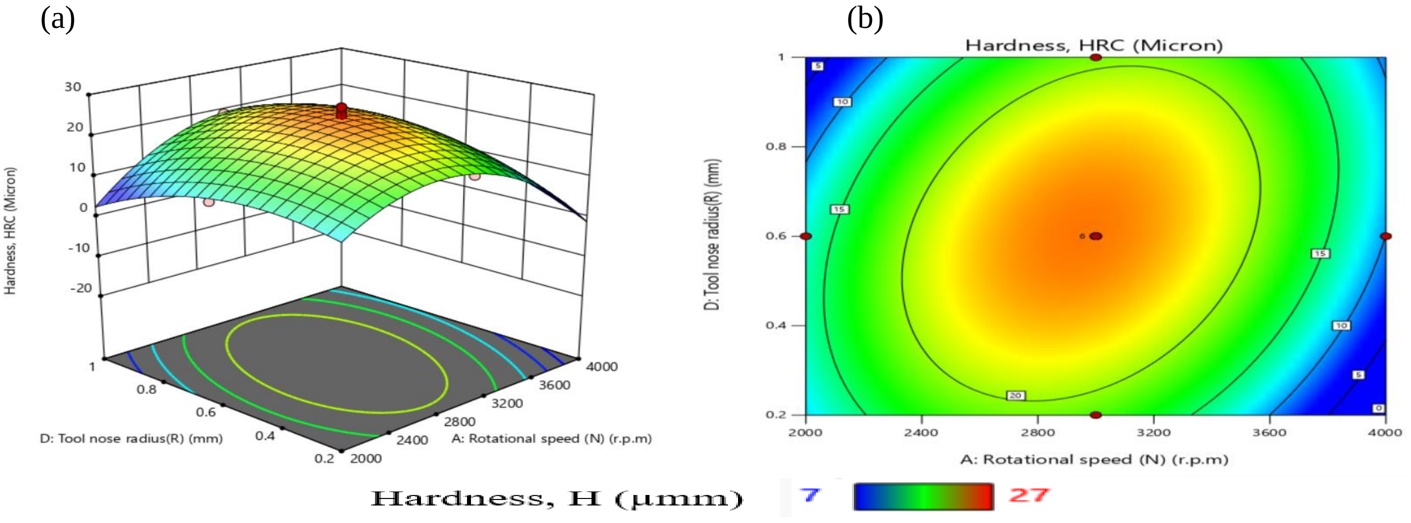

S47: Interaction of N and R on H: (a) 3D surface; (b) contour plot.

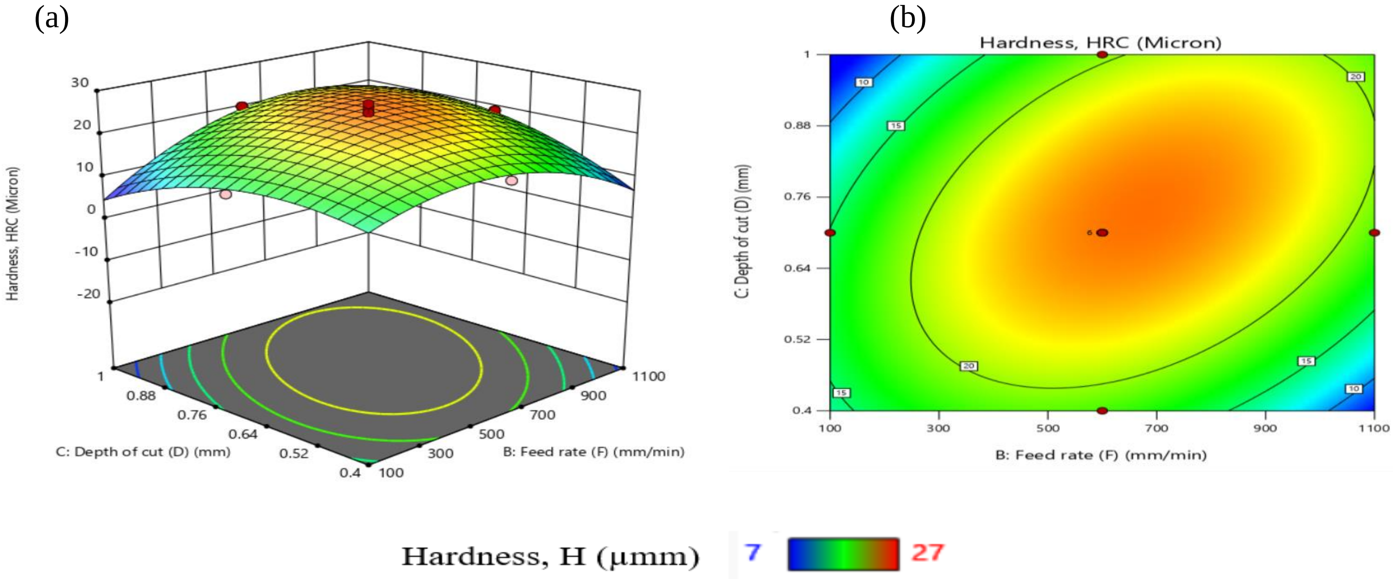

S48. Interaction of F and D on H: (a) 3D surface; (b) contour plot.

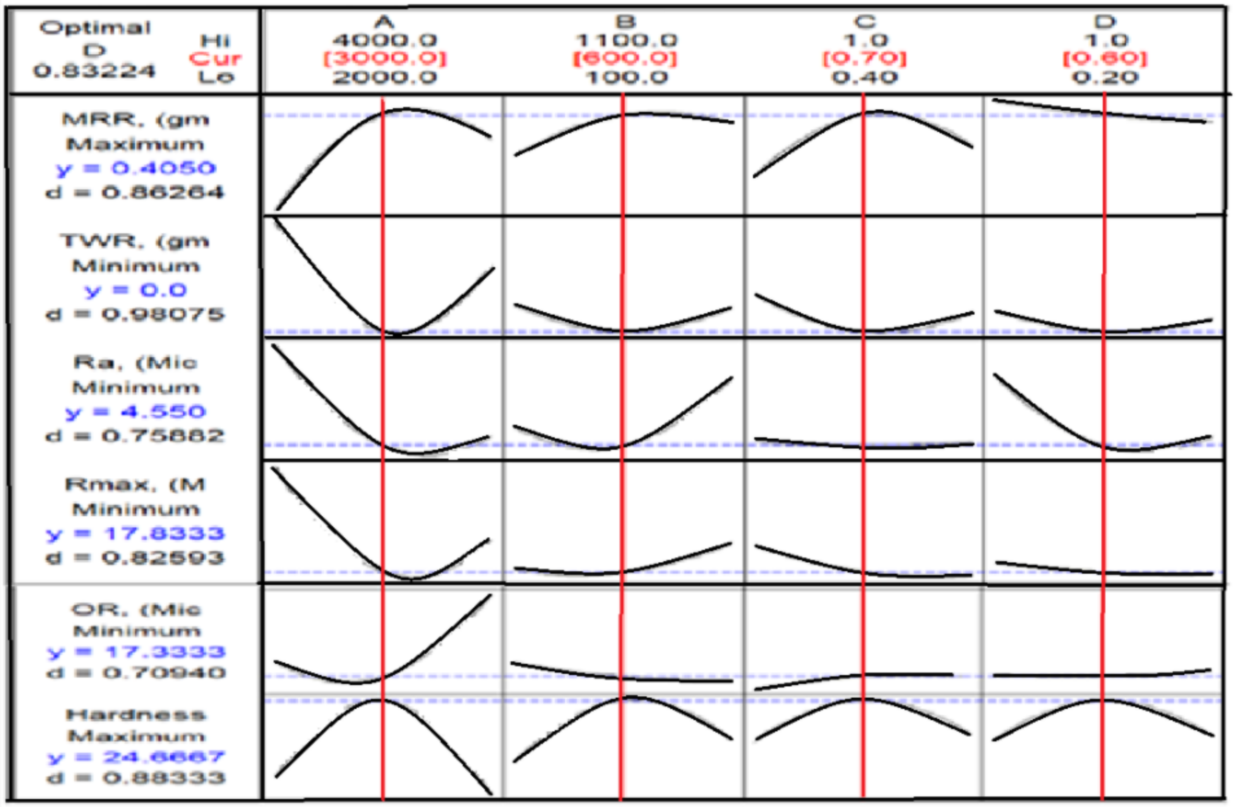

S49: Multi-objective optimization outcomes for turning CK45.

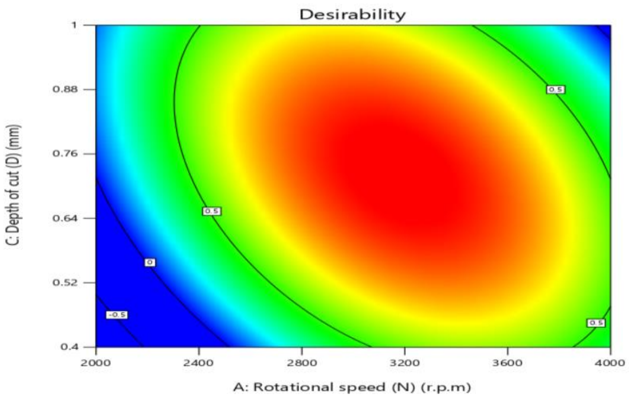

(a) Contour plots for results of D(X) vs. N and D (hold values: F=600 mm/min, R=0.6 mm)

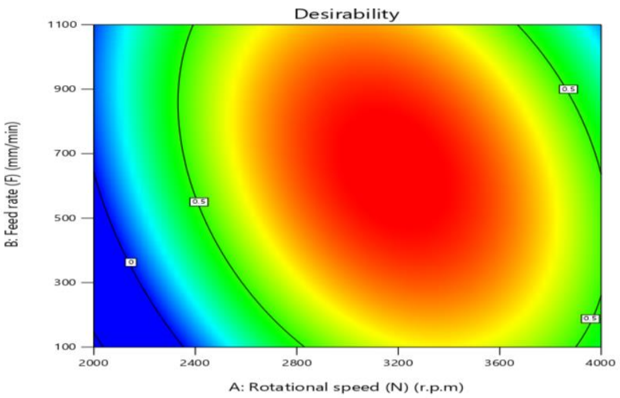

(b) Contour plots for results of D(X) vs. N and F (hold values: D=0.7 mm, R=0.6 mm)

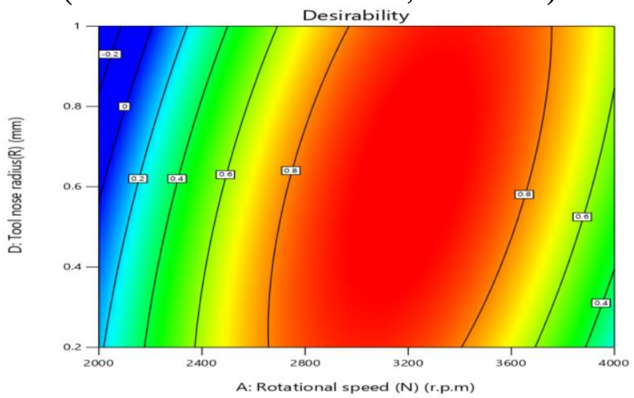

(c) Contour plots for results of D(X) vs. N and R (hold values: F=600 mm/min, D=0.7 mm)

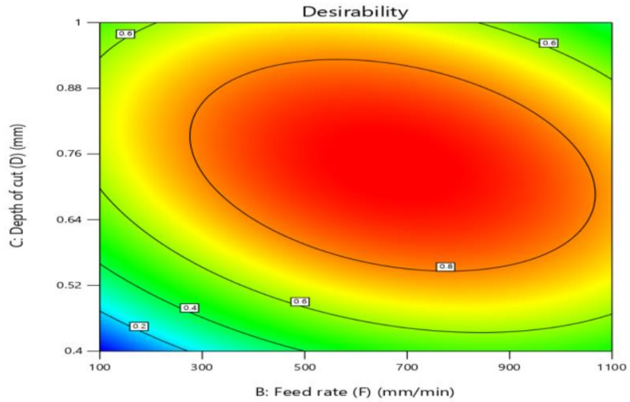

(d) Contour plots for results of D(X) vs. F and D (hold values: N=3000 r.p.m, R=0.6 mm)

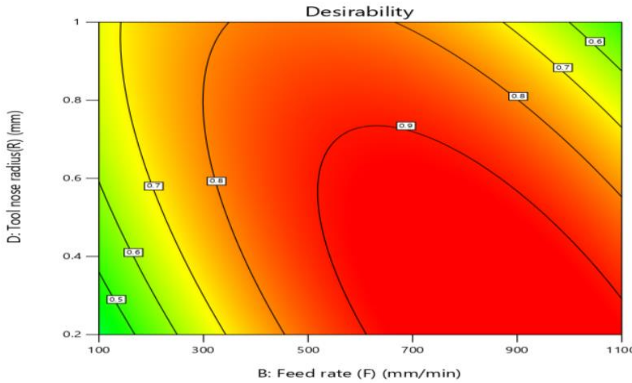

(e) Contour plots for results of D(X) vs. F and R (hold values: N=3000 r.p.m, D=0.7 mm)

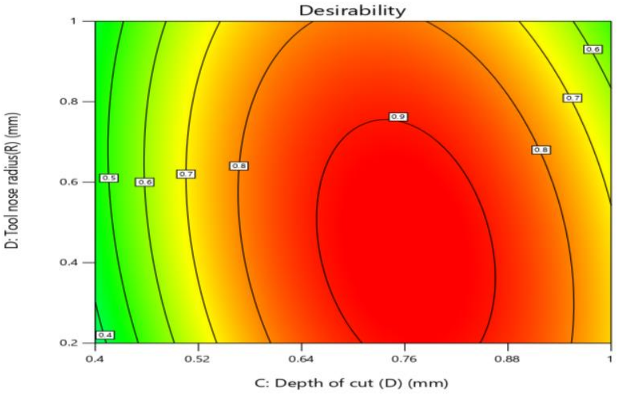

(f) Contour plots for results of D(X) vs. D and R (hold values: N=3000 r.p.m, F=600 mm/min)

S50: (a, b, c, d, e, f) Contour plots of the D(X) function for CK45 workpieces machined using TP parameters.

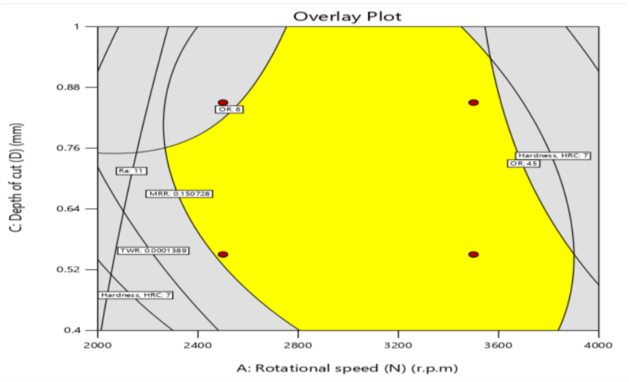

(a) Overlay Plot vs. N and D (hold values: F=350 mm/min, R=0.8 mm)

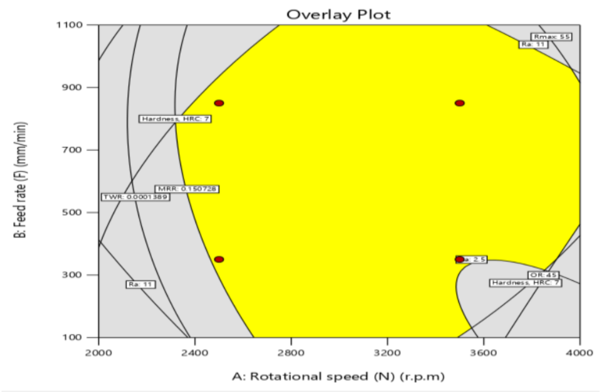

(b) Overlay Plot vs. N and F (hold values: D=0.8 mm, R=0.55 mm)

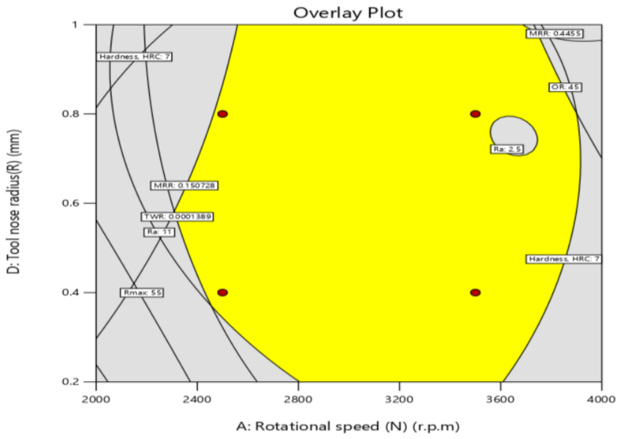

(c) Overlay Plot vs. N and R (hold values: F=350 mm/min, D=0.55 mm)

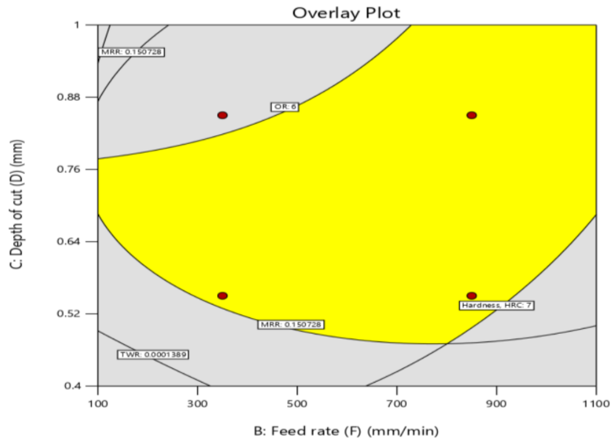

(d) Overlay Plot vs. F and D (hold values: N=2500 r.p.m, R=0.8 mm)

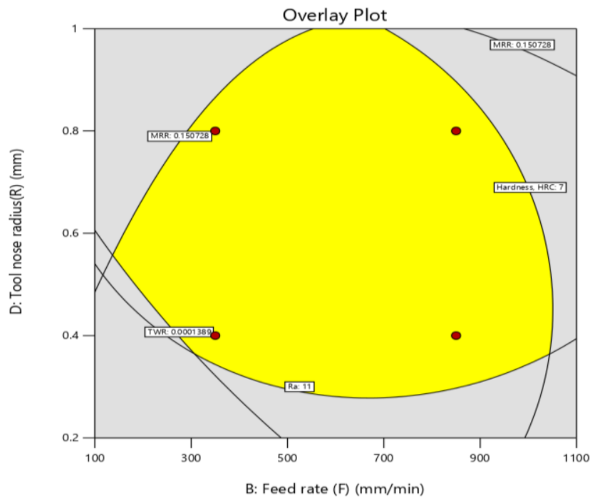

(e) Overlay Plot vs. F and R (hold values: N=2500 r.p.m, D=0.55 mm)

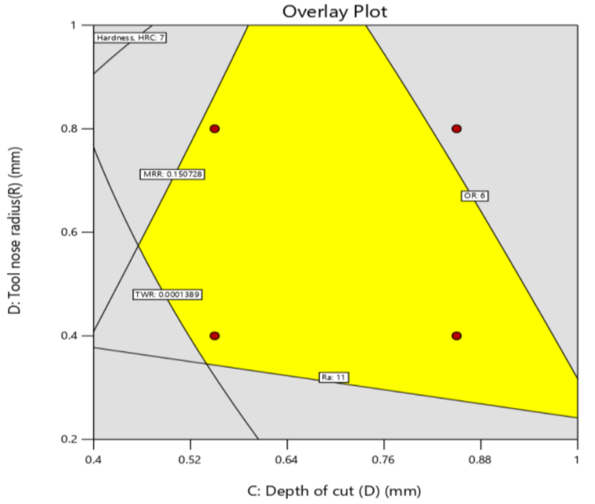

(f) Overlay Plot vs. D and R (hold values: N=2500 r.p.m, F=350 mm/min)

S51: (a, b, c, d, e, f) Overlay Plot for multi-response for results Ck45 workpieces turned by rough (TP).

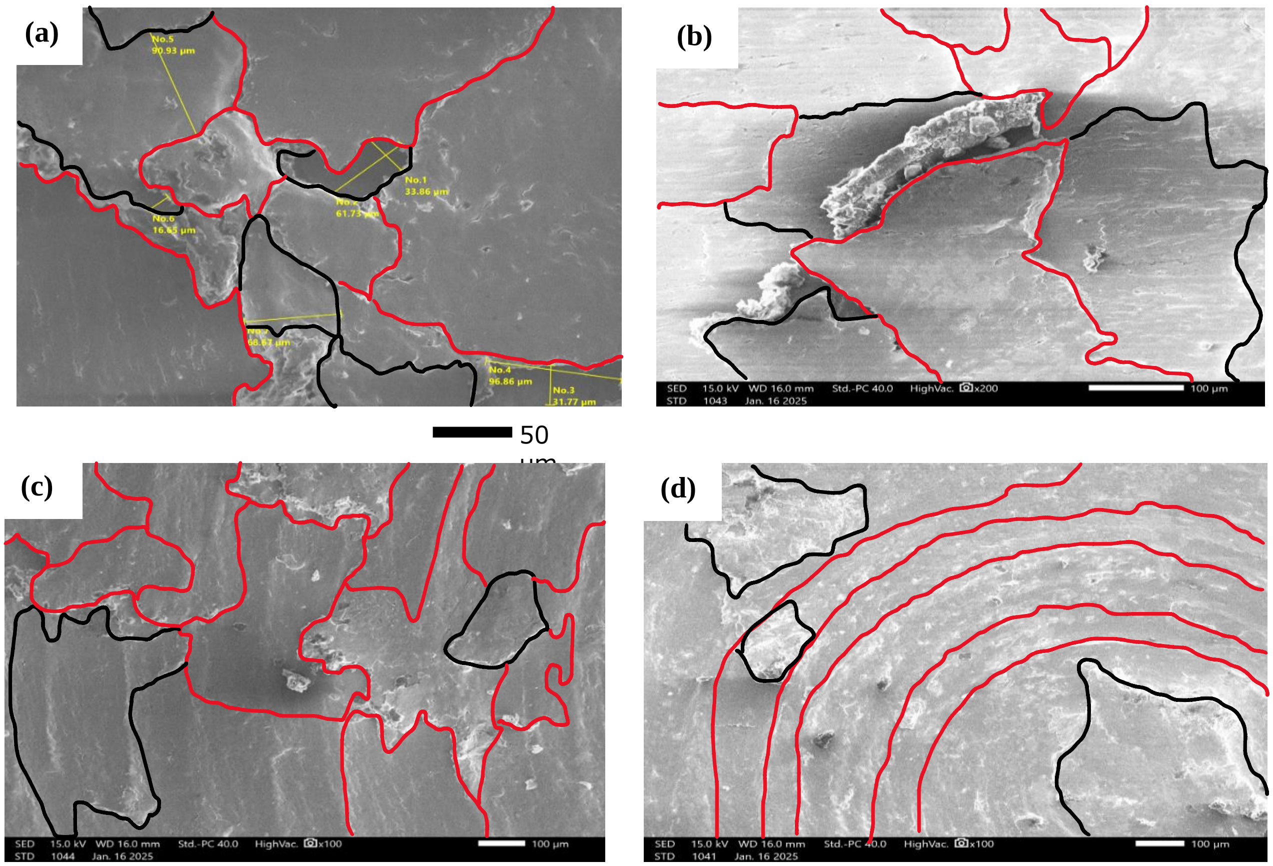

S52: SEM photos of different positions (a) first position, (b) second position, (c) third position, and (d) fourth position of CK45 workpiece at bad (TP) machining conditions: N= 2000 r.p.m, F= 600 mm/min, D= 0.70 mm and R= 0.6 mm.

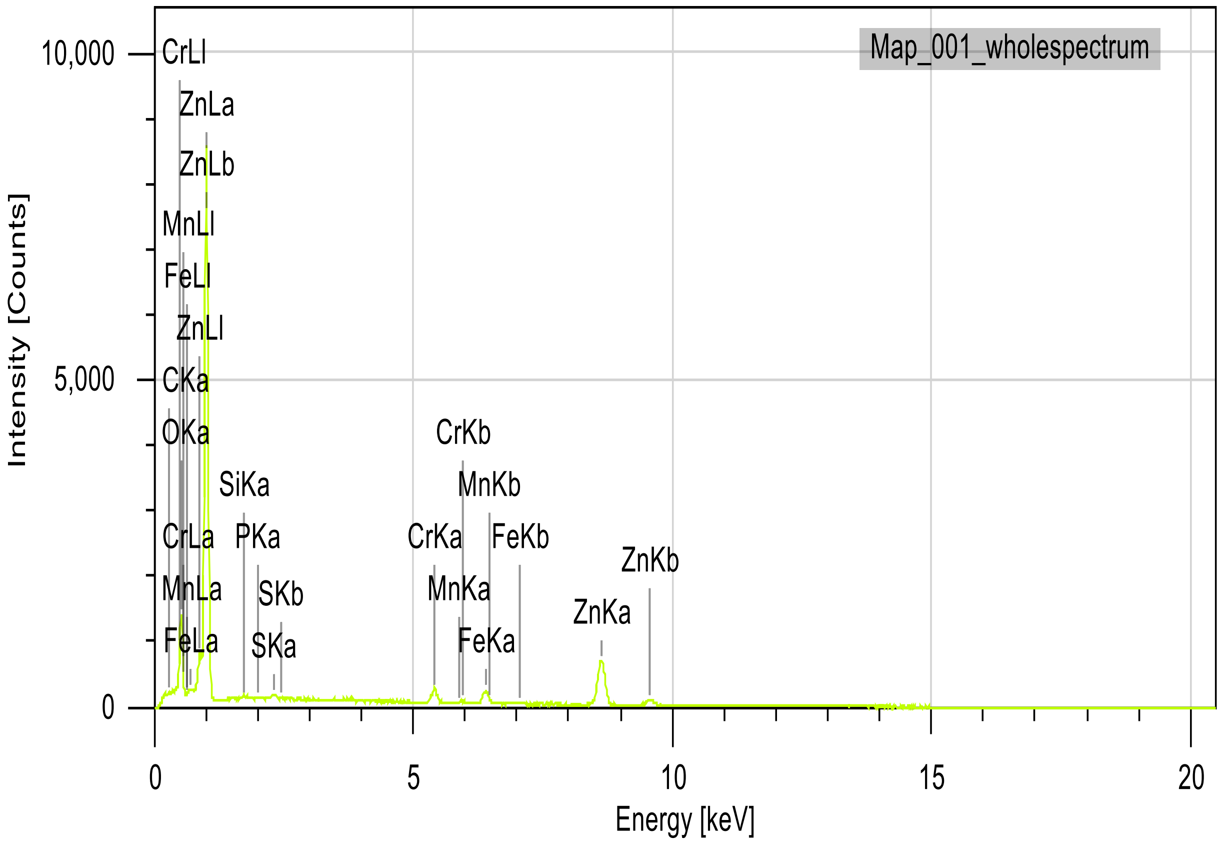

S53: EDS analysis of fourth position of CK45 workpiece at bad (TP) machining conditions: N= 2000 r.p.m, F= 600 mm/min, D= 0.70 mm and R= 0.6 mm.

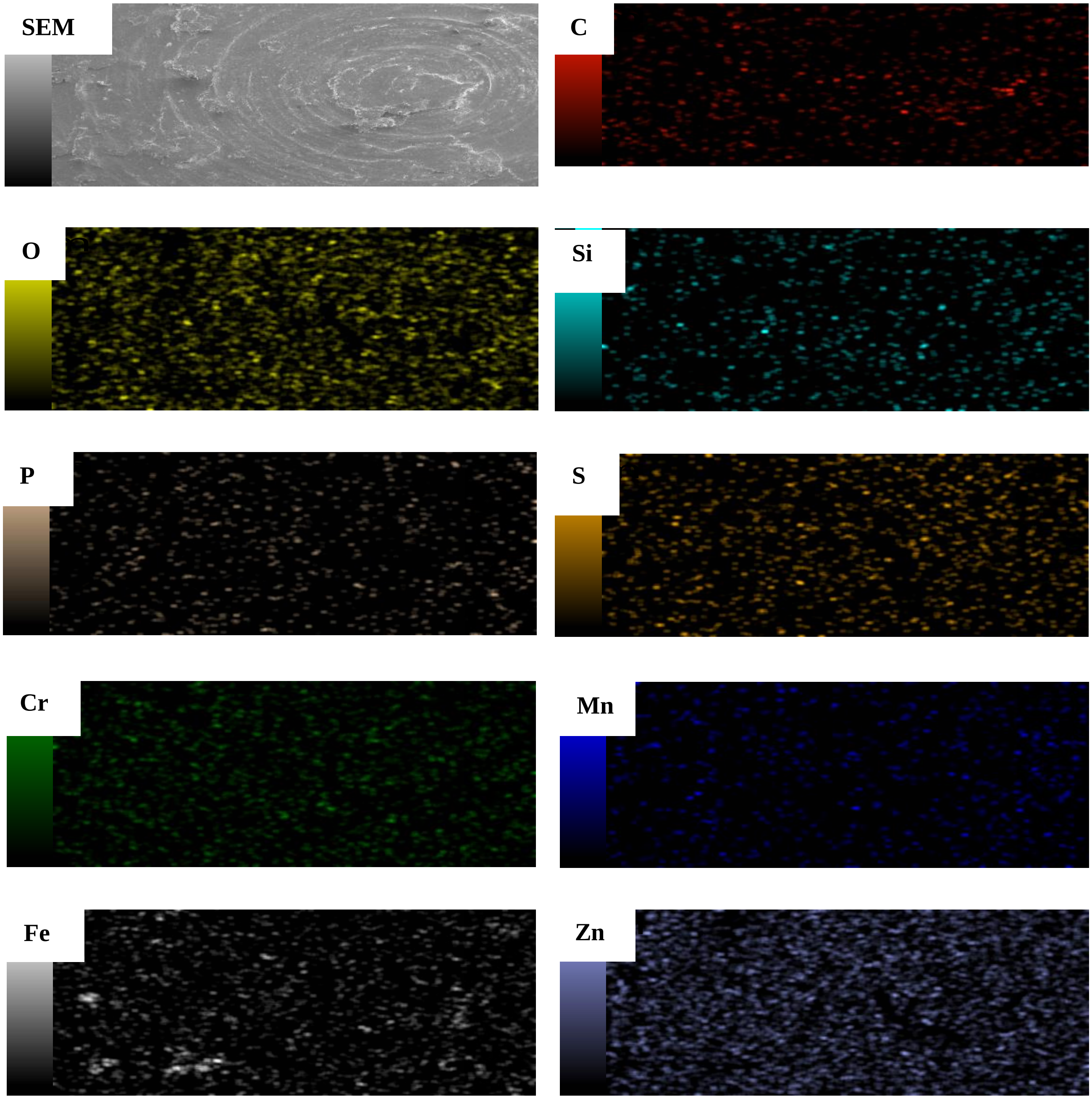

S54: Map analysis of fourth position of CK45 workpiece at bad (TP) machining conditions: N= 2000 r.p.m, F= 600 mm/min, D= 0.70 mm and R= 0.6 mm.

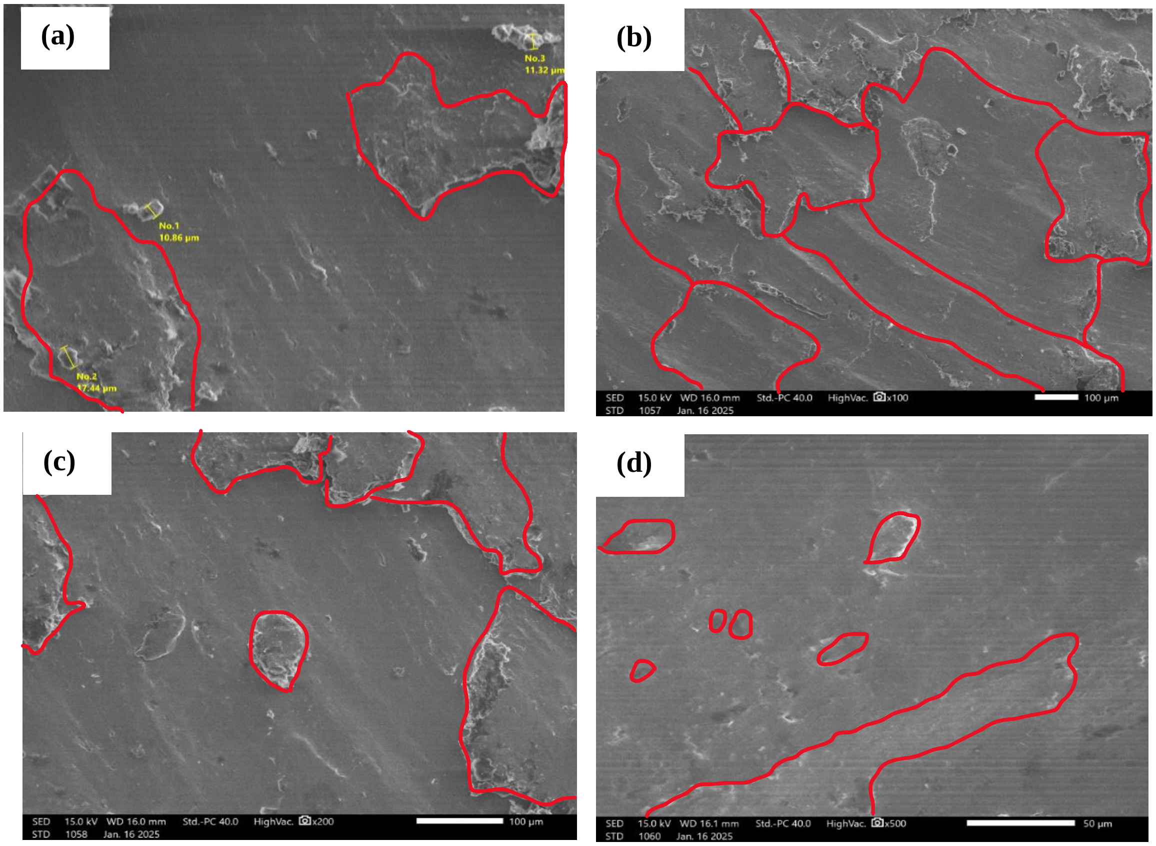

S55: SEM photos of different positions (a) first position, (b) second position, (c) third position, and (d) fourth position of CK45 workpiece at optimum (TP) machining conditions: N= 3000 r.p.m, F= 600 mm/min, D= 0.70mm and R= 0.6 mm.

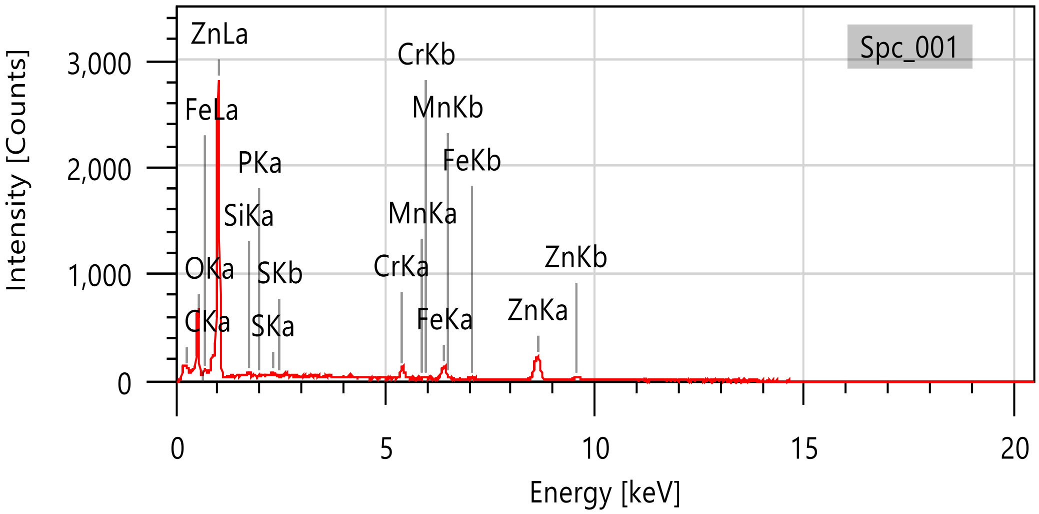

S56: EDS analysis of second position of CK45 workpiece at (TP) optimum machining conditions at N= 3000 r.p.m, F= 600 mm/min, D= 0.70 mm and R= 0.6 mm.

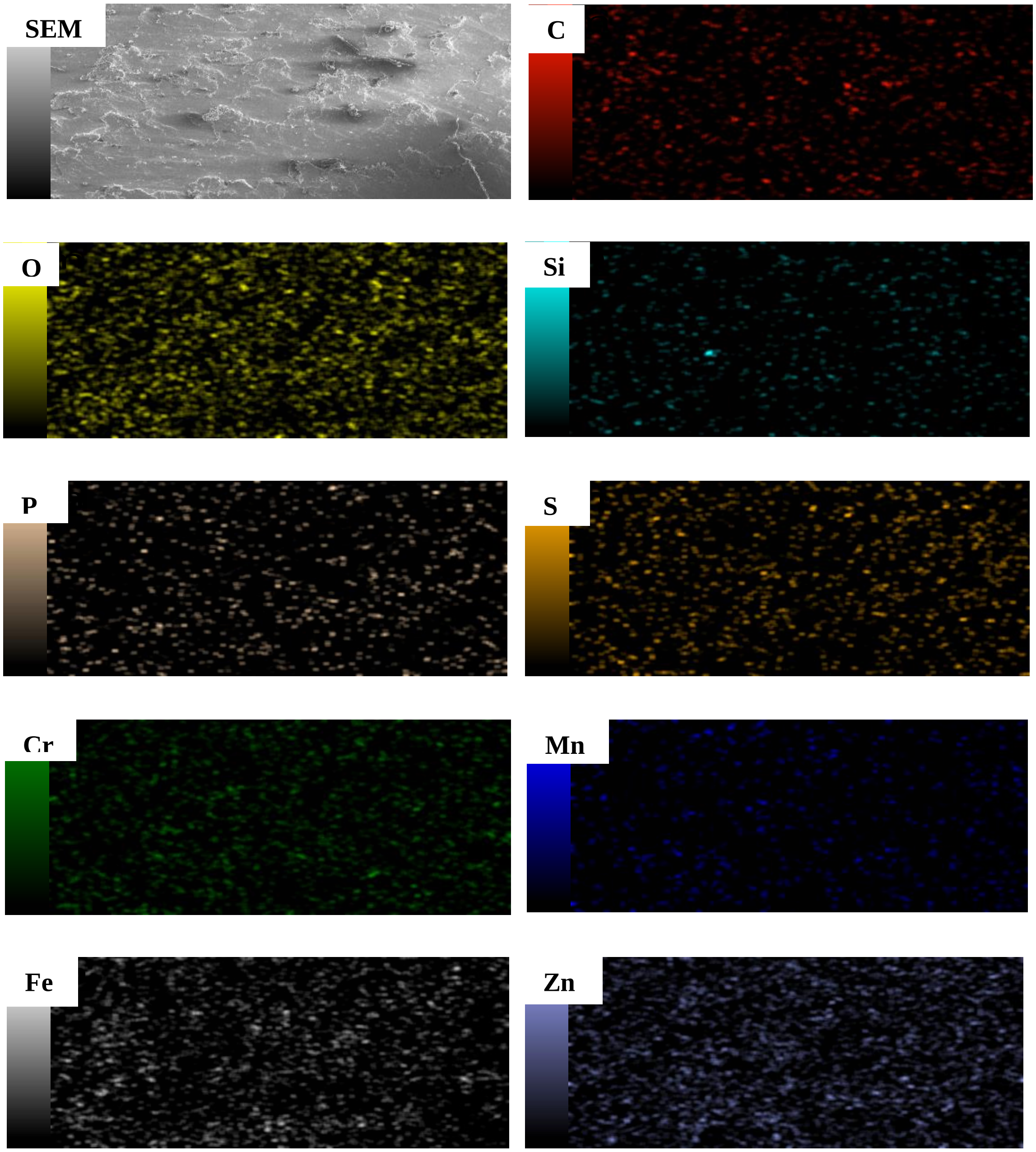

S57: Map analysis of second position of CK45 workpiece at optimum (TP) machining conditions at N= 3000 r.p.m, F= 600 mm/min, D= 0.70 mm and R= 0.6 mm.

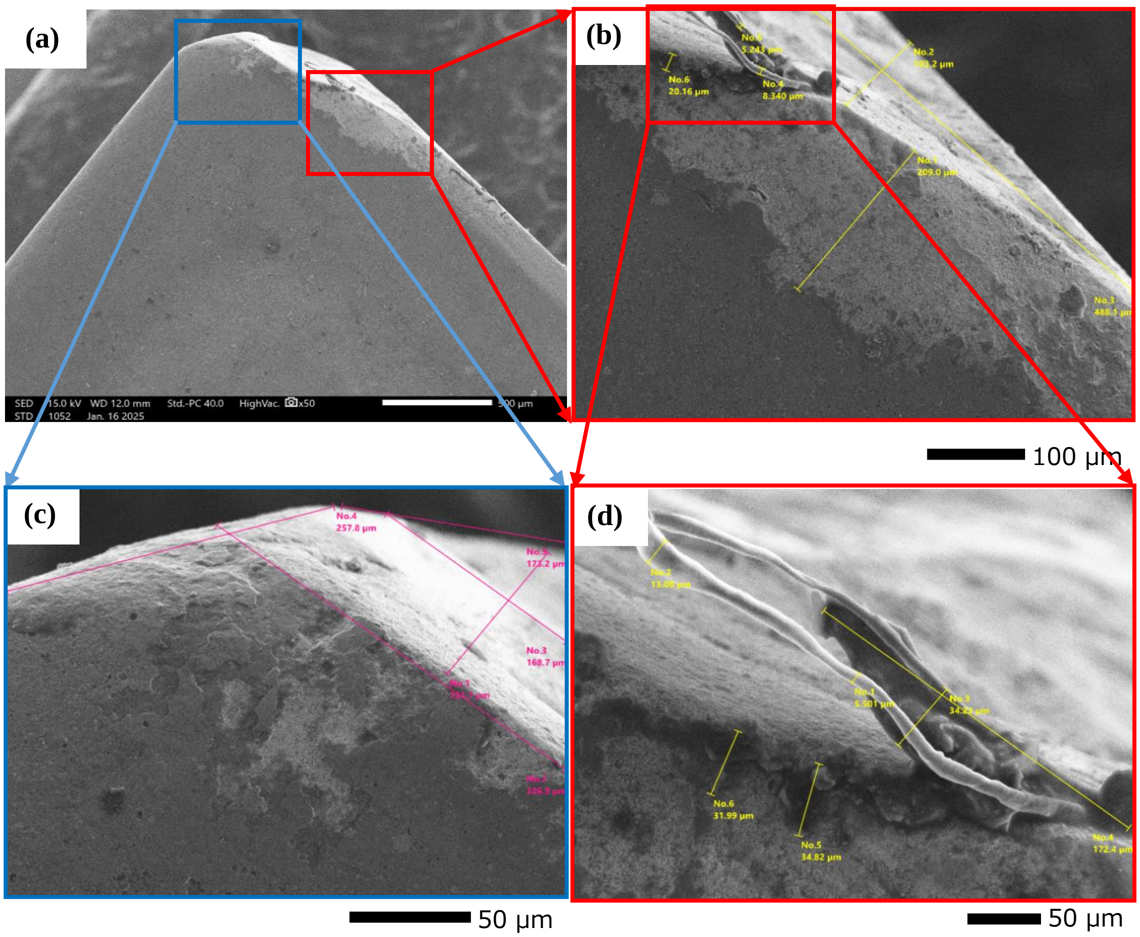

S58: SEM photos of different positions (a) first position, (b) second position, (c) third position, and (d) fourth position of (CBN) insert at bad TP machining conditions for CK45 workpiece at N= 2000 r.p.m, F= 600 mm/min, D= 0.70 mm and R= 0.6 mm.

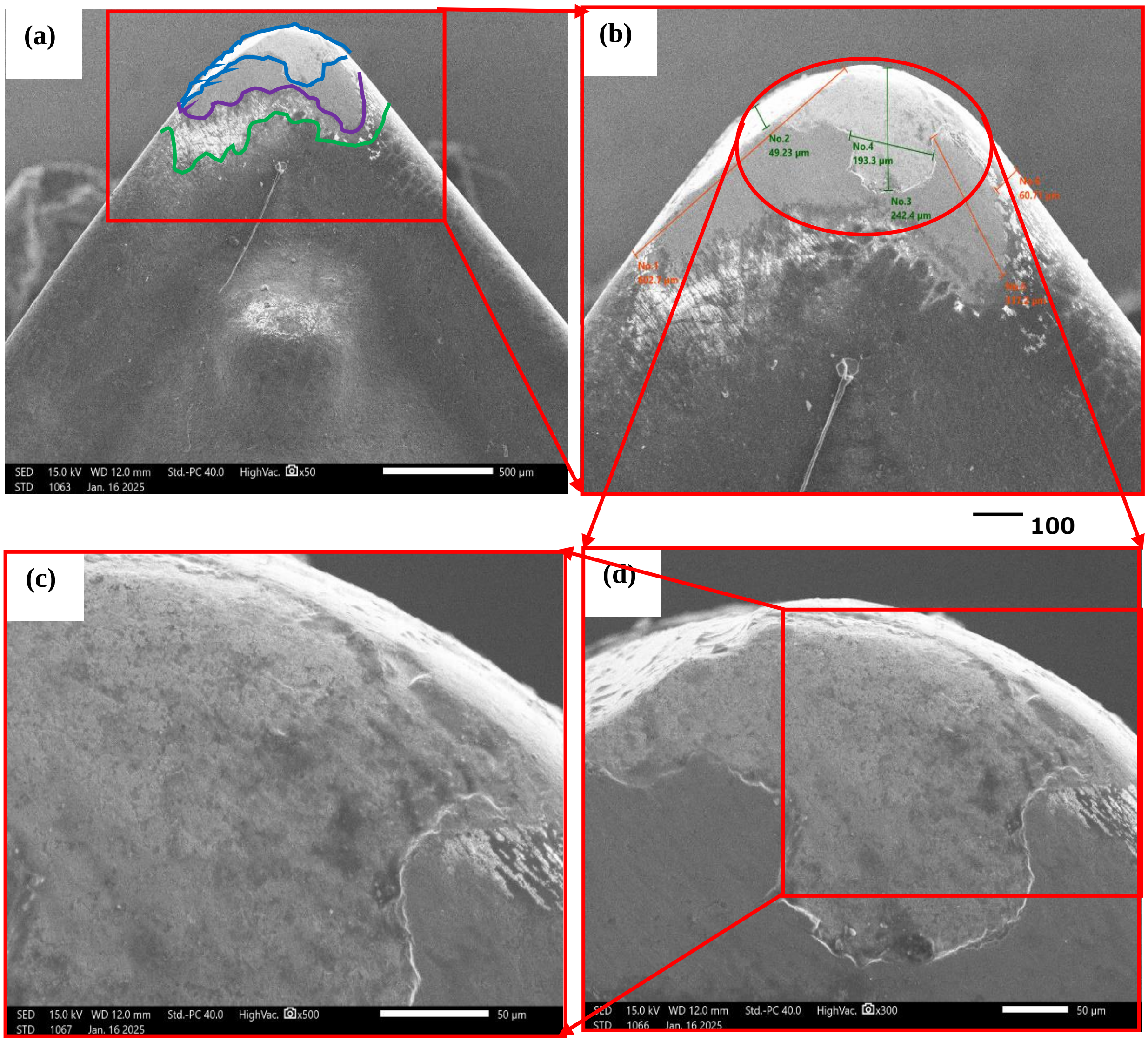

S59: SEM photos of different positions (a) first position, (b) second position, (c) third position, and (d) fourth position of (CBN) insert at optimum (TP) machining conditions for CK45 steel workpiece at N= 3000 r.p.m, F= 600 mm/min, D= 0.70 mm and R= 0.6 mm.
